# CRISPR-mediated mutations in the ABC transporter gene *ABCA2* confer pink bollworm resistance to Bt toxin Cry2Ab

**DOI:** 10.1038/s41598-021-89771-7

**Published:** 2021-05-17

**Authors:** Jeffrey A. Fabrick, Dannialle M. LeRoy, Lolita G. Mathew, Yidong Wu, Gopalan C. Unnithan, Alex J. Yelich, Yves Carrière, Xianchun Li, Bruce E. Tabashnik

**Affiliations:** 1 USDA ARS, U.S. Arid Land Agricultural Research Center, 21881 N. Cardon Lane, Maricopa, AZ 85138 USA; 2 College of Plant Protection, Nanjing Agricultural University, Nanjing, 210095 China; 3 Department of Entomology, University of Arizona, Tucson, AZ 85721 USA; 4 Present Address: Pairwise Plants, Durham, NC 27701 USA

**Keywords:** Agricultural genetics, Genetic engineering, CRISPR-Cas9 genome editing, Agricultural genetics, Molecular evolution

## Abstract

Crops genetically engineered to produce insecticidal proteins from *Bacillus thuringiensis* (Bt) have many benefits and are important globally for managing insect pests. However, the evolution of pest resistance to Bt crops reduces their benefits. Understanding the genetic basis of such resistance is needed to better monitor, manage, and counter pest resistance to Bt crops. Previous work shows that resistance to Bt toxin Cry2Ab is associated with mutations in the gene encoding the ATP-binding cassette protein ABCA2 in lab- and field-selected populations of the pink bollworm (*Pectinophora gossypiella*), one of the world’s most destructive pests of cotton. Here we used CRISPR/Cas9 gene editing to test the hypothesis that mutations in the pink bollworm gene encoding ABCA2 (*PgABCA2*) can cause resistance to Cry2Ab. Consistent with this hypothesis, introduction of disruptive mutations in *PgABCA2* in a susceptible strain of pink bollworm increased the frequency of resistance to Cry2Ab and facilitated creation of a Cry2Ab-resistant strain. All Cry2Ab-resistant individuals tested in this study had disruptive mutations in *PgABCA2.* Overall, we found 17 different disruptive mutations in *PgABCA2* gDNA and 26 in *PgABCA2* cDNA, including novel mutations corresponding precisely to single-guide (sgRNA) sites used for CRISPR/Cas9. Together with previous results, these findings provide the first case of practical resistance to Cry2Ab where evidence identifies a specific gene in which disruptive mutations can cause resistance and are associated with resistance in field-selected populations.

## Introduction

Genetically engineered crops that produce insecticidal proteins derived from *Bacillus thuringiensis* (Bt) are widely cultivated. In 2019, 109 million hectares of transgenic Bt crops were grown in 27 countries^1^. Bt crops have improved control of several major insect pests and decreased conventional insecticide use, leading to increased yield and farmer profits, as well as health and environmental benefits^2–5^. However, some pests have evolved practical resistance to Bt crops, thereby reducing their benefits^6–11^. Better understanding of the genetic basis of resistance is useful for monitoring, managing, and countering pest resistance to Bt crops.

To kill insects, the crystalline (Cry) toxins deployed widely in transgenic crops must be ingested and bind to receptor proteins in the midgut^12,13^. For Cry1A toxins, receptors include ATP-binding cassette (ABC) transporter proteins, cadherins, aminopeptidases N, and alkaline phosphatases^14–19^. Resistance to Cry2Ab in the lepidopteran pests *Helicoverpa armigera* and *Helicoverpa punctigera* is associated with reduced binding of Cry2Ab to larval midgut membranes and is linked with autosomal recessive mutations in the ABC transporter gene *ABCA2*^20,21^. Furthermore, knockout or removal of portions of the gene encoding ABCA2 using CRISPR/Cas9 or TALENs caused resistance to Cry2Ab in *H. armigera, Trichoplusia ni,* and *Bombyx mori*^22–24^.

Here we tested the hypothesis that CRISPR/Cas9-mediated disruption of the gene encoding ABCA2 can cause resistance to Cry2Ab in the pink bollworm (*Pectinophora gossypiella*), one of the world’s most damaging pests of cotton^25,26^. Sustained susceptibility to Bt cotton producing Cry1Ac and Cry2Ab was critical for eradicating pink bollworm from the cotton-growing areas of the continental United States and Mexico^27^. However, this lepidopteran remains an important pest in many other countries and has evolved practical resistance to Cry1Ac and Cry2Ab in India^26,28^. Previous work shows that mutations disrupting *PgABCA2*, the pink bollworm gene encoding ABCA2 protein, are associated with resistance to Cry2Ab in field-selected populations from India and in two lab-selected strains from the United States^29,30^. However, gene editing has not been used previously to determine if mutations in any pink bollworm gene can cause resistance to a Bt toxin.

We discovered that introducing CRISPR/Cas9-mediated mutations in the *PgABCA2* gene in a susceptible strain of pink bollworm increased the frequency of resistance to Cry2Ab and facilitated creation of a Cry2Ab-resistant strain. All Cry2Ab-resistant individuals tested in this study harbored disruptive mutations in *PgABCA2* DNA, including some novel mutations corresponding precisely to the single-guide RNA (sgRNA) target sites we used for gene editing.

## Results

### In vitro Cas9 cleavage of *PgABCA2* PCR products with guide RNAs

We tested five sgRNAs separately by combining the sgRNA/Cas9 ribonucleotide mixture for each sgRNA with *PgABCA2* PCR products amplified from gDNA. Cas9 cleavage of the *PgABCA2* amplicons in vitro occurred with sgRNAs 1, 4 and 5, but not sgRNAs 2 and 3 (Supplementary Figs. S1 and S2).

### Creation of Cry2Ab-resistant strain CRISPR-R2

Of 637 embryos (G_0_) from the susceptible APHIS-S strain injected with the ribonucleotide mixture containing all five sgRNAs, 36% hatched (231) and 19% pupated (121). To start the CRISPR-R2 strain, we pooled 10 G_0_ pupae and allowed the adults to eclose and mate. After three generations of rearing larvae on untreated diet, we screened G_4_ neonates on diet containing the diagnostic concentration of 3 μg Cry2Ab per mL diet. Survival from egg to pupa was 0.83% based on 400 pupae obtained from approximately 48,000 eggs we placed near the treated diet. As a control, we used the same method to test approximately 50,000 eggs from APHIS-S that were not injected and not descendants of insects injected as embryos. For the control, survival from egg to pupa on treated diet was 0%, which is significantly lower than survival of the G_4_ larvae (Fisher’s exact test, P < 0.00001). These results show that the mutations introduced by CRISPR significantly increased the frequency of resistance to Cry2Ab.

We used the G_4_ survivors of exposure to Cry2Ab to continue the CRISPR-R2 strain. After five additional generations of rearing larvae on untreated diet, adjusted survival was 96% (n = 32 larvae) when G_10_ neonates were tested at the diagnostic concentration of Cry2Ab.

### Mutations in sgRNA target sites in *PgABCA2* gDNA from the CRISPR-R2 strain

Consistent with the in vitro cleavage results (Supplementary Figs. S1 and S2), 15 of the 20 G_4_ survivors we analyzed from the CRISPR-R2 strain had mutations in *PgABCA2* gDNA in the target sites of sgRNAs 1, 4, and/or 5, but not sgRNAs 2 or 3 (Table 1, Supplementary Fig. S3). The numbers of survivors with mutations corresponding to the sgRNA target sites were 12 for sgRNA1, one for sgRNA4, and five for sgRNA5, which includes three individuals with mutations at two target sites (Table 1, Supplementary Fig. S3). Overall, we found 17 different mutations in the *PgABCA2* gDNA sequenced from the survivors (Supplementary Fig. S3).

**Table 1:** Mutations corresponding to sgRNA target sites in *PgABCA2* gDNA in 20 Cry2Ab-resistant pink bollworm larvae from the G_4_ of the CRISPR-R2 strain.

Individual #	sgRNA 1	sgRNA 2	sgRNA 3	sgRNA 4	sgRNA 5
1	Indel^a^	0^b^	0	0	0
2	Indel	0	0	0	0
3^c^	0	0	0	0	0
4	0	0	0	Substitution^d^	Deletion
5^c^	0	0	0	0	0
6	Indel	0	0	0	0
7	Indel	0	0	0	Deletion
8	Indel	0	0	0	0
9^c^	0	0	0	0	0
10^c^	0	0	0	0	0
11	0	0	0	0	Deletion
12	Indel	0	0	0	0
13	0	0	0	0	Indel
14	Indel	0	0	0	nd^e^
15	Indel	0	0	0	nd
16	Indel	0	0	0	0
17	Indel	0	0	0	0
18^c^	0	0	0	0	0
19	Indel	0	0	0	0
20	Indel	nd	nd	nd	Indel

^a^Insertion/deletion mutation.

^b^Wild-type, sequence identical to *PgABCA2* from APHIS-S.

^c^Lacked mutations at all five sgRNA target sites.

^d^Single base pair substitution.

^e^Not determined, unable to sequence the PCR product.

### Mutations in *PgABCA2 *cDNA from Cry2Ab-resistant larvae of the CRISPR-R2 strain that lacked *PgABCA2* gDNA mutations in the sgRNA target sites

**Figure 1:**
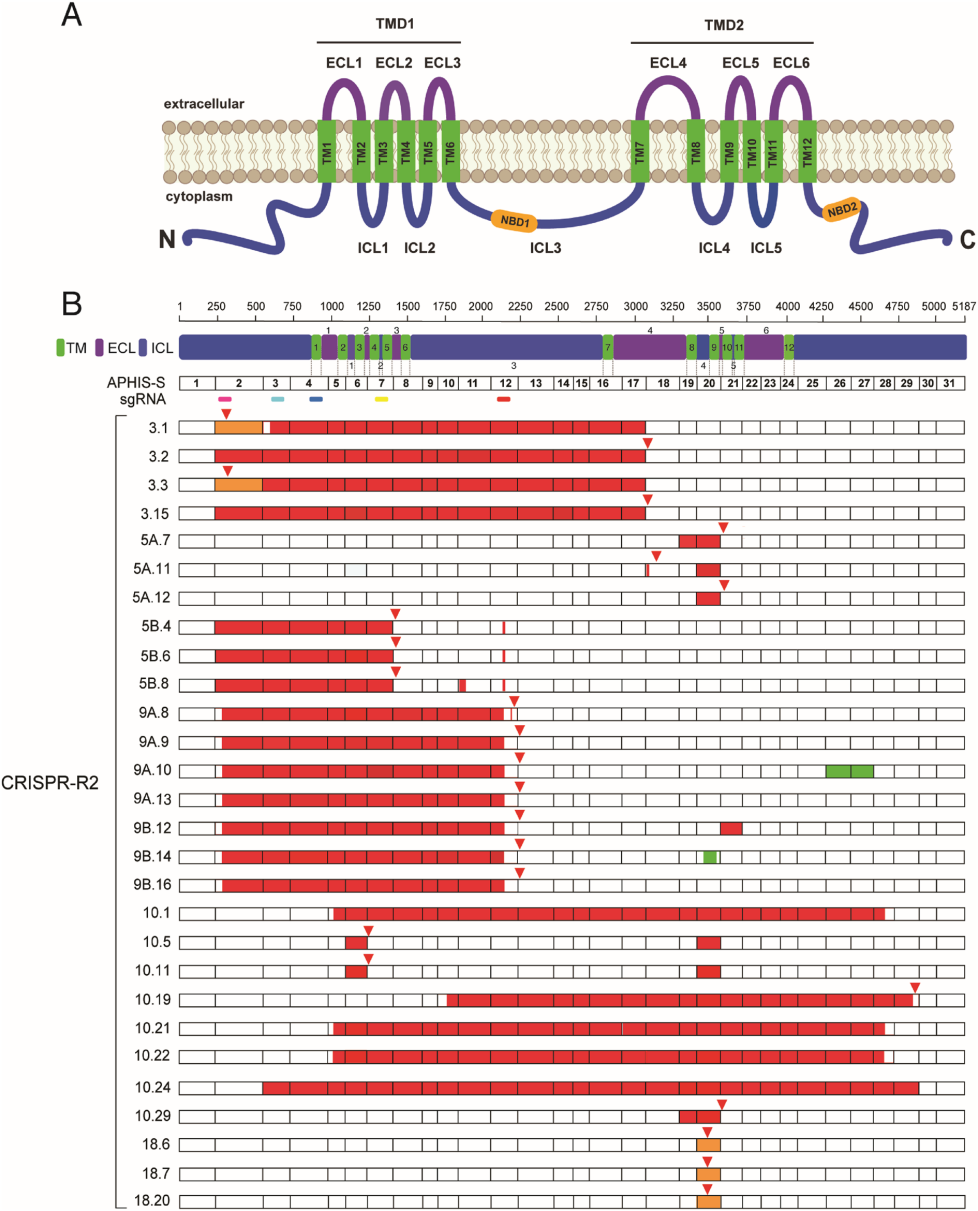
Mutations in 28 *PgABCA2* cDNA sequences from five Cry2Ab-resistant pink bollworm larvae from the CRISPR-R2 strain that lacked gDNA mutations in sgRNA target sites. (**A**) The predicted PgABCA2 protein includes amino (N) and carboxyl (C) termini and transmembrane domains (TMD1 and TMD2). Each TMD contains six transmembrane-spanning regions (TM, green), three extracellular loops (ECL, purple), and two intracellular loops (ICL, blue). The two TMDs are connected by a single intracellular loop (ICL3). ICL3 and the C-terminal domain each contain a nucleotide-binding domain (NBD, orange). (**B**) Mutations in *PgABCA2* cDNAs from CRISPR-R2 (3–8 clones from each of five individuals: 3, 5, 9, 10 and 18) relative to the susceptible strain APHIS-S (MG637361). Individuals with two distinct PCR products cloned are indicated as A or B (e.g., 5A and 5B, etc.). Numbers to the right of the decimal point for each individual indicate the clone sequenced. Exon numbers are shown for APHIS-S. Location of sgRNAs 1–5 are shown as colored bars (sgRNA1, magenta; sgRNA2, cyan; sgRNA3, blue; sgRNA4, yellow; sgRNA5, red). Red triangles above bars indicate premature stop codons, which occur in all sequences except 10.1, 10.21, 10.22, and 10.24. Colors within bars show mutations: orange for insertions and deletions (indels), red for deletions, and green for insertions.

All 28 cDNA sequences from the five G_4_ survivors that lacked mutations in the *PgABCA2* gDNA at the five sgRNA target sites harbored disruptive mutations in *PgABCA2* cDNA (Fig. 1, Table 2, Supplementary Fig. S4). Of the 28 sequences, 24 included at least one premature stop codon and the other four (all from larva 10) had one deletion affecting 24 or 27 exons (Fig. 1, Supplementary Fig. S5). Three of the five larvae (3, 5 and 9) had cDNA mutations corresponding to target sites for sgRNA1, 4, or 5 (Fig. 1B, Table 2, Supplementary Fig. S4). Collectively, four cDNA clones from larva 3 had three indels and two deletions, all affecting exons corresponding to the five sgRNAs (Fig. 1B, Table 2, Supplementary Fig. S4A). For larva 5, two distinct PCR bands were amplified and cloned (5A and 5B), with insert sizes ranging from 3.8 to 4.9 kb (Supplementary Table S3). Six clones from larva 5 revealed deletions including three with removal of exons 2–7 corresponding to sgRNAs 1–4 and a 7-bp deletion in exon 12 corresponding to sgRNA5 (Fig. 1B, Table 2, Supplementary Fig. S4B). All seven clones from larva 9 had a deletion beginning exactly in sgRNA1 in exon 2 and ending at sgRNA5 in exon 12 (Fig. 1B, Table 2, Supplementary Fig. S4C).

**Table 2:** Twenty-six *PgABCA2* cDNA mutations in 11 Cry2Ab-resistant pink bollworm larvae from the CRISPR-R2 strain (n = 5) and CRISPR-R2 X APHIS-S F_2_ progeny (n = 6).

cDNA mutation^a^	Larva.clone^b^	Codon^c^	Exon^d^	Type^e^	Effect^f^	sgRNA sites^g^	Current source^h^	Previous source^i^
c.295_559delins	3.1	99	2	fs	Stop	1	CRISPR-R2	No
c.677_3096delins	3.1	226	3–17	fs	Stop	2	CRISPR-R2	No
c.240_3100del	3.2	80	2–17	fs	Stop	No (1–5)	CRISPR-R2	No
c.295_3100delins	3.3	99	2–17	fs	Stop	1	CRISPR-R2	No
c.240_3096del	3.15	80	2–17	fs	Stop	No (1–5)	CRISPR-R2	No
c.3313_3589del	5A.7, A.1.1, A.2.2, A.2.9, 10.29	1105	19–20	fs	Stop	No	Both	Bt4-R2
c.3097_3100del	5A.11, C.4.6	1033	18	fs	Stop	No	Both	BX-R
c.3418_3589del	5A.11, 5A.12, 10.5, 10.11, C.3.4	1140	20	fs	Stop	No	Both	Bt4-R2
c.241_1409del	5B.4, 5B.6, 5B.8	80	2–7	fs	Stop	No (1–4)	CRISPR-R2	No
c.1835_1976del	5B.8	612	11	fs	Stop	No	CRISPR-R2	No
c.2125_2131del	5B.4, 5B.6, 5B.8, C.4.6, C.4.8, C.4.10	708	12	fs	Stop	5	Both	No
c.294_2128del	9A.8, 9A.9, 9A.10, 9A.13, 9B.12, 9B.14, 9B.16	98	2–12	fs	Stop	1, 5	CRISPR-R2	No
c.2220_2221delinsAG	9A.8	740	12	fs	Stop	No	CRISPR-R2	No
c.4438_4439ins	9A.10	1479	26–27	fs	Stop	No	CRISPR-R2	No
c.3589_3714del	9B.12	1196	21	InF	In-frame del	No	CRISPR-R2	No
c.3555_3590ins	9B.14	1185	20	fs	Stop	No	CRISPR-R2	No
c.1021_4668del	10.1, 10.21, 10.22	340	5–28	InF	In-frame del	No (4–5)	CRISPR-R2	No
c.1090_1234del	10.5, 10.11, A.2.9, C.3.4, C.4.6	364	6	fs	Stop	No	Both	Bt4-R2, BX-R, India
c.1755_4587del	10.19	584	10–29	fs	Stop	No (5)	CRISPR-R2	No
c.559_4884del	10.24	186	3–29	InF	In-frame del	No (2–5)	CRISPR-R2	No
c.3556_3588delins	18.6, 18.7, 18.20, C.3.6	1185	20	fs	Stop	No	CRISPR-R2	Bt4-R2
c.4598_4599ins	C.3.6	533	27	fs	Stop	No	F_2_	No
c.294_295delinsGAGA	C.4.6, C.4.8, C.4.10	98	2	fs	Stop	1	F_2_	No
c.674_681del	C.4.6, C.4.8, C.4.10	225	3	fs	Stop	2	F_2_	No
c.283_296del	J.5.2, J.5.3, J.5.6, J.6.4	95	2	fs	Stop	1	F_2_	No
c.679_2229delins	J.5.2, J.5.3, J.5.6, J.6.4	227	3–12	fs	Stop	2	F_2_	No

^a^cDNA nomenclature showing the nucleic acid sequence changes in *PgABCA2* (MG637361.1) based on the recommendations by the Human Genome Variation Society (http://www.hgvs.org/).

^b^Entries start with a number for the five larvae from the CRISPR-R2 strain (3, 5, 9, 10 and 18) and with a letter for the six larvae from F_2_ progeny of three single-pair families from CRISPR-R2 X APHIS-S crosses (A, C and J). For each entry, the first number indicates the larva and the second number indicates the clone (e.g., 3.1 indicates larva 3, clone 1). For 5A and 5B as well as 9A and 9B, two distinct PCR products (A and B) were cloned and sequenced from a single larva.

^c^Codon number beginning from the initiation codon and indicating disrupted position in the coding sequence.

^d^Disrupted exon in the coding sequence.

^e^Type of mutation (fs, frame shift; InF, in-frame mutation).

^f^Result of mutation in the coding sequence (Introduction of premature stop codon; In-frame del; In-frame deletion).

^g^Numbers 1, 2 or 5 indicate the sgRNA site directly affected; no indicates none of the five sgRNA sites were directly affected and numbers in parentheses indicate sgRNA sites indirectly affected (mutations occurred in exons containing sgRNA sites).

^h^Source of larvae in the current study: CRISPR-R2, F_2_ progeny of single-pair families from CRISPR-R2 X APHIS-S, or both.

^i^No indicates not found in previous studies; other entries indicate the same cDNA mutation was previously found in one or more of the following sets of Cry2Ab-resistant insects: lab-selected Bt4-R2 and BX-R strains from Arizona and field-selected populations from India^29,30^.

The two remaining G_4_ survivors (larvae 10 and 18) did not have cDNA mutations in target sites of sgRNAs 1–5 (Fig. 1B, Table 2, Supplementary Fig. S4D,E). Larva 10 yielded a diverse set of *PgABCA2* clones, ranging from 810 bp to approximately 4.8 kb, each with a premature stop codon, a deletion, or both (Fig. 1B, Supplementary Table S3). Larva 18 had only one mutation in all three clones sequenced: an indel in exon 20 (c.3556_3588delins) that matches the *r*_*A1*_ mutation previously reported in the lab-selected Cry2Ab-resistant strain Bt4-R2^29^. Collectively, three of the five larvae (5, 10 and 18) from CRISPR-R2 that lacked gDNA mutations in sgRNA sites 1–5 had five cDNA mutations that do not correspond to sgRNA sites and were reported previously in Cry2Ab-resistant laboratory-selected strains from Arizona (Bt4-R2 and BX-R), field-selected populations from India, or both (c.1090_1234del, c.3097_3100del, c.3313_3589del, c.3418_3589del, and c.3556_3588delins; Table 2)^29,30^.

### Mutations in *PgABCA2* cDNA from Cry2Ab-resistant F_2_ progeny of CRISPR-R2 X APHIS-S single-pair crosses

**Figure 2:**
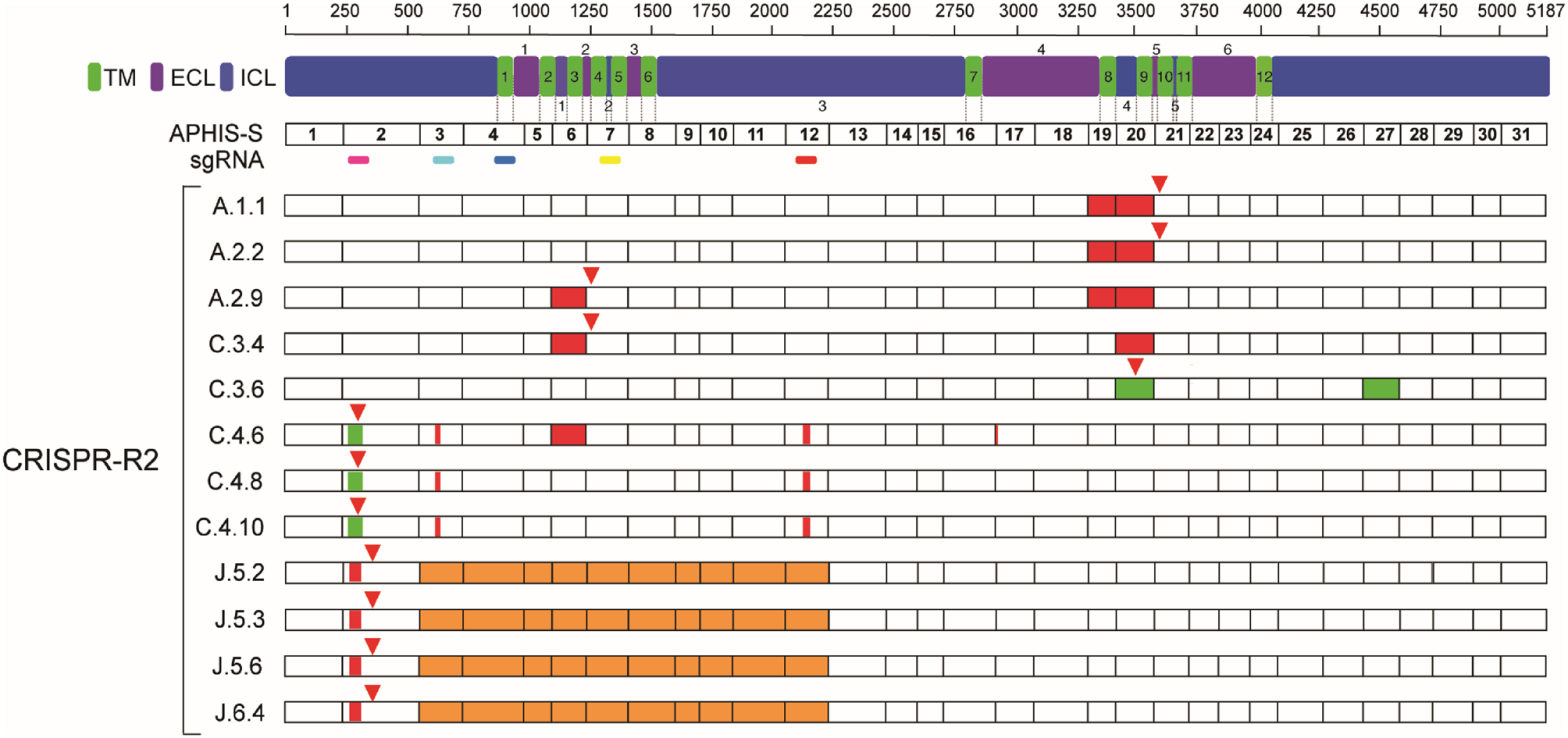
Mutations in 12 *PgABCA2* cDNA sequences from six Cry2Ab-resistant pink bollworm larvae from F_2_ progeny of CRISPR-R2 X APHIS-S single-pair families. Mutations in *PgABCA2* cDNAs from CRISPR-R2 survivors from three single-pair families (1–3 clones from each of five individuals from families A, C, and J) relative to the susceptible strain APHIS-S (MG637361). Numbers to the right of the family name indicates individual and clone number, respectively (e.g., A.1.1 indicates Family A, Individual 1, Clone 1). Exon numbers are shown for APHIS-S. Location of sgRNAs 1–5 are shown as colored bars (sgRNA1, magenta; sgRNA2, cyan; sgRNA3, blue; sgRNA4, yellow; sgRNA5, red). Red triangles above bars indicate premature stop codons. Colors within bars show mutations: orange for insertions and deletions (indels), red for deletions, and green for insertions.

We sequenced *PgABCA2* cDNA of six larvae from the F_2_ progeny of CRISPR-R2 X APHIS-S single-pair families A, C, and J that survived on diet containing the diagnostic concentration of Cry2Ab. All 12 cDNA sequences from these six Cry2Ab-resistant larvae harbored disruptive mutations in *PgABCA2* cDNA (Fig. 2, Table 2, Supplementary Fig. S6, Supplementary Table S4). Three of the six survivors (C.4, J.5 and J.6) had cDNA mutations affecting sgRNA sites 1, 2 and 5 that introduce premature stop codons (Fig. 2, Table 2, Supplementary Figs. S6, S7). The other three F_2_ survivors (A.1, A.2, and C.3) had no cDNA mutations in the five sgRNA sites, but all clones from these larvae had other disruptive mutations in the *PgABCA2* cDNA (Fig. 2, Table 2). We discovered a novel mutation (c.4598_4599ins) in clone 6 from larva C.3 (clone C.3.6) that does not correspond to sgRNA target sites 1–5 (Fig. 2) and was not previously found in Cry2Ab-resistant pink bollworm^29,30^. Collectively, four F_2_ survivors (A.1, A.2, C.3 and C.4) had the same five cDNA mutations seen in CRISPR-R2 that were previously found in Cry2Ab-resistant lab-selected strains from Arizona, field-selected populations from India, or both (Table 2).

### Summary of mutations in *PgABCA2* cDNA of CRISPR-R2 and F_2_ progeny of CRISPR-R2 X APHIS-S single-pair crosses

One or more disruptive mutations occurred in all 40 *PgABCA2* cDNA sequences we obtained from 11 Cry2Ab-resistant larvae (5 from the CRISPR-R2 strain and 6 from F_2_ survivors from single-pair crosses between CRISPR-R2 and APHIS-S; Figs. 1 and 2, Table 2). In these 40 sequences, we detected 26 different cDNA mutations and a total of 67 cDNA mutations counting the multiple occurrences for 13 of the mutations (Table 2). Of the 26 cDNA mutations, 23 are insertions, deletions, or indels causing frameshifts that introduce premature stop codons and the remaining three are in-frame deletions expected to cause loss of exons 3–29, 5–28, or 21 (Fig. 2, Table 2).

Nine of the 26 cDNA mutations occur precisely at sgRNA sites and six affect exons containing at least one sgRNA site (Table 2, Supplementary Figs. S4, S6). Of the remaining 11 cDNA mutations, six are novel and five were previously identified from lab-selected strains from Arizona, field-selected populations from India, or both (Table 2)^29,30^.

## Discussion

The results here show that CRISPR editing of the pink bollworm *ABCA2* gene (*PgABCA2*) in the susceptible APHIS-S strain significantly increased the frequency of resistance to Bt toxin Cry2Ab. These results imply that mutations in *PgABCA2* can cause resistance to Cry2Ab, which is consistent with previous results indicating that pink bollworm resistance to Cry2Ab is genetically linked with recessive mutations in *PgABCA2* in two lab-selected strains from Arizona (Bt4-R2 and BX-R) and associated with mutations in *PgABCA2* in field-selected populations from India^29,30^. The results with pink bollworm also correspond with evidence that mutations in *ABCA2* genes introduced by CRISPR or TALENs caused resistance to Cry2Ab in *H. armigera, T. ni,* and *B. mori*^22–24^ and that naturally occurring deletions at this locus were associated with resistance to Cry2Ab in *H. armigera* and *H. punctigera*^21^.

In the present study, disruptive mutations occurred in all of the *PgABCA2* cDNA sequences obtained from the 26 Cry2Ab-resistant larvae evaluated (Tables 1 and 2). In the 20 Cry2Ab-resistant larvae from which gDNA was sequenced, 15 larvae had a deletion, an indel, or both (Table 1). All of the 40 cDNA sequences derived from 11 Cry2Ab-resistant larvae (including 5 from which we also analyzed gDNA) had a deletion that introduces a premature stop codon, a large deletion, or both (Table 2). The results here are consistent with previous work showing that disruptive mutations occurred in all *PgABCA2* DNA sequences analyzed from Cry2Ab-resistant larvae from the lab-selected Bt4-R2 strain and from five field-selected populations from India^29,30^. By contrast, sequence analysis and interstrain crosses with Bt4-R2 revealed that some Cry2Ab-resistant individuals from the lab-selected BX-R strain harbored disruptive mutations in *PgABCA2* DNA, whereas others had a different mechanism of resistance that does not involve this gene^30^.

CRISPR editing probably caused the 17 mutations in *PgABCA2* gDNA and the nine mutations in *PgABCA2* cDNA that correspond precisely with one or more of the five sgRNA target sites. In principle, the 17 mutations in *PgABCA2* cDNA that do not correspond precisely with any of the five sgRNA target sites may reflect the effects of CRISPR that acted outside of the target sites, standing genetic variation in the susceptible APHIS-S strain, or both. However, alleles conferring resistance to Cry2Ab were rare in APHIS-S, as indicated by 0% survival for the larvae from approximately 50,000 eggs of APHIS-S tested here on diet containing 3 μg Cry2Ab per mL diet and in several similar previous tests with smaller sample sizes^30,32,36,37,43^.

After we injected G_0_ embryos from the susceptible strain APHIS-S with five sgRNAs targeting *PgABCA2* and reared larvae on untreated diet for three generations, G_4_ survival on diet containing a diagnostic concentration of Cry2Ab was 0.83% versus 0% for controls from APHIS-S. Assuming recessive inheritance based on our previous results^29,30^, genotype proportions based on Hardy–Weinberg equilibrium, and that all resistance to Cry2Ab in the G_4_ larvae was caused by mutations in *PgABCA2*, we can estimate the total frequency of resistance alleles at this locus was 0.091 for the G_4_ larvae (square root of 0.0083 = 0.091). We did not find substantial fitness costs in a multi-toxin resistant strain of pink bollworm (AZP-R2) that derived its resistance to Cry2Ab from Bt4-R2, which harbors naturally occurring mutations in *PgABCA2*^29,30,32^. However, if some or all of the CRISPR-induced *PgABCA2* mutations in CRISPR-R2 do cause fitness costs, this would have tended to reduce the frequency of resistance alleles during rearing of CRISPR-R2 on untreated diet from G_0_ to G_4_. Thus, the 0.091 frequency of resistance alleles estimated for G_4_ could underestimate the frequency of resistance alleles at G_0._

In comparison with the results here, 8.0% of 113 randomly chosen G_1_ pupae of *H. armigera* descended from injected G_0_ insects were homozygous for the specific indel mutation introduced by CRISPR in exon 18 of *HaABCA2*^22^. This yields an estimated frequency of 0.28 for this particular resistance allele (square root of 0.08 = 0.28). Based on the proportion of G_1_ moths of *Plutella xylostella* heterozygous for specific CRISPR-mediated mutations in the ABC transporter genes *PxABCC2*, *PxABCC3*, or both^31^, we estimated the resistance allele frequency for each specific mutation introduced was 0.021, 0.031, and 0.005, respectively. Thus, the estimated frequency of resistance alleles in our study was intermediate relative to the two previous studies mentioned above. Whereas the estimate from our study includes all resistance alleles, the other estimates include only the specific CRISPR-induced resistance alleles.

Although CRISPR gene editing can identify mutations that can cause resistance, analyzing insects from the field is needed to determine which naturally occurring mutations are actually associated with practical resistance^31,33–35^. The results here demonstrate that knocking out *PgABCA2* with CRISPR-induced mutations can cause pink bollworm resistance to Cry2Ab. Previous results show that practical resistance to Cry2Ab in pink bollworm from India is associated with many disruptive mutations in *PgABCA2*^29^. These results together provide the first case for practical resistance to Cry2Ab where evidence identifies a specific gene in which disruptive mutations can cause resistance and are associated with resistance in field-selected populations. For practical resistance to Cry2Ab in other lepidopteran pests, it will be useful to determine if mutations in *ABCA2* genes can cause resistance and if such mutations are associated with practical resistance in the field.

## Methods

### Insects

We used the APHIS-S strain of pink bollworm as the susceptible strain in all experiments. APHIS-S is susceptible to Cry2Ab^30,32,36,37^ and had been reared in the laboratory for more than 30 years without exposure to Bt toxins or insecticides^38,39^. Larvae were reared on wheat germ diet^40^. All rearing and diet bioassays were done at 26 °C, 14 h light:10 h dark.

### Bt toxin

We used purified and solubilized protoxin of Cry2A.127, an engineered variant of Cry2Ab that was prepared as described previously^41^ and provided by Corteva Agriscience. Cry2A.127 is 98.6% identical with Cry2Ab1 and Cry2Ab2 (9 substitutions out of 633 amino acids) and is referred to here as Cry2Ab.

### Design and synthesis of single guide RNA (sgRNA)

Based on the conserved DNA sequence of 5′-CCNN_18_CC-3′^42^, we designed five target sgRNAs from within exon 2, 3, 4, 7 and 12 of the *PgABCA2* gene^29^ (Supplementary Table S1). Each selected sgRNA target sequence was checked for potential off-target sites by BLAST searching of the GenBank non-redundant database. Double-stranded DNA fragments (e.g. gBlock) were synthesized by Integrated DNA Technologies (Coralville, Iowa), with each containing a T7 RNA polymerase binding site (5′-TAATACGACTCACTATA-3′), the 20-bp *PgABCA2*-specific target region (Supplementary Table S1), and the 80-bp common stem-loop tracrRNA sequence (5′-GTTTTAGAGCTAGAAATAGCAAGTTAAAATAAGGCTAGTCCGTTATCAACTTGAAAAAGTGGCACCGAGTCGGTGCTTTT-3′).

Each 125-bp synthetic gBlock DNA was used as a template for RNA synthesis by in vitro transcription using the HiScribe T7 Quick High Yield RNA Synthesis Kit (New England Biolabs, Ipswich, MA). Transcribed sgRNAs were purified using the Agencourt RNAClean XP system (Beckman Coulter Life Sciences, Indianapolis, IN) and quantified with a NanoDrop One spectrophotometer (Thermo Fisher Scientific, Waltham, MA).

### In vitro testing of Cas9 cleavage with guide RNAs

To test each sgRNA and determine if Cas9 cuts PCR-amplified *PgABCA2* gDNA in vitro, we used the Guide-it sgRNA Screening Kit (Takara Bio, Mountain View, CA). First, we extracted gDNA from APHIS-S 4th instar larvae using the Gentra Puregene Tissue Kit (Qiagen, Hilden, Germany), which served as DNA template for PCR. Primer pairs 104PgABCA2-5 + 82PgABC3, 178PgABCA2-5 + 192PgABCA2-3, 174PgABCA2-5 + 192PgABCA2-3, 83PgABC5 + 190PgABCA2-3, and 53PgABCA2-5 + 139PgABCA2-3 were used to amplify PCR products corresponding to exon 1 to exon 2, exon 3 to exon 5, exon 4 to exon 5, exon 7 to exon 8, and exon 11 to exon 12, respectively. Each sgRNA was diluted to 50 ng μL^−1^ in RNase-free water, incubated at 60 °C for 3 min, before returning to ice. Then, 250 ng of Cas9 nuclease was incubated with 1 μL of each sgRNA, mixed, and incubated at 37 °C for 5 min. Approximately 50 ng of each PCR template was combined with Cas9 Reaction Buffer, Bovine Serum Albumin, and RNase-free water and appropriate Cas9/sgRNA mixture and incubated at 37 °C for 1 h. Reactions were terminated by heating at 80 °C for 5 min. Aliquots of undigested controls and each digestion reaction were separated by 1.5% agarose gel electrophoresis and stained with SYBR Safe DNA Gel Stain (Thermo Fisher Scientific).

### Embryo microinjection

We collected freshly laid APHIS-S eggs naturally affixed to microscope glass coverslips (40 × 60 mm) that had been placed for 50 min in cages containing several hundred moths. Before injections, we used a pressurized air canister to remove loose debris from the coverslip (e.g., wing scales and loosely attached eggs).

Aliquots of sgRNA (total of 400 ng each in 3.5 μL of RNase-free water) were heat denatured at 60 °C for 3 min and immediately returned to ice. Recombinant Cas9 protein (at a final concentration of 300 ng mL^−1^ in RNase-free water) from *Streptococcus pyogenes* (PNA Bio, Newbury Park, CA) was then added to each denatured sgRNA solution (final sgRNA concentration of either 80 or 300 ng μL^−1^) and incubated on ice for 10 min. Prior to microinjection, one microliter of each of the five sgRNA + Cas9 solutions were pooled and backloaded into a pulled borosilicate needle.

Individual APHIS-S embryos (1–2 h post collection) were injected with approximately 100–200 picoliters of the sgRNA/Cas9 solution using a Narishige IM-200 microinjector (Narishige International USA, Amityville, NY) equipped with an Olympus IMT-2 inverted microscope (Olympus Corporation, Center Valley, PA). Over 12 days, we injected 637 eggs (n = 301 with 80 ng μL^−1^ sgRNAs + 300 ng μL^−1^ Cas9 and n = 336 with 300 ng μL^−1^ sgRNAs + 300 ng μL^−1^ Cas9). Following injections, glass coverslips with eggs were placed on top of a piece of aluminum foil (to prevent direct contact of glass slide with diet and thereby reducing the potential for microbial contamination) in 350 mL paper cups containing wheat germ diet. G_0_ 4^th^ instar larvae cutouts were allowed to pupate in Hexcel sheets (Hexcel Corporation, Casa Grande, AZ; PN1 commercial aramid fiber honeycomb, https://www.plascore.com/).

### Creation of the Cry2Ab-resistant strain CRISPR-R2

Of the initial 637 embryos that were injected, 121 survived to G_0_ pupae. Of these, we pooled 10 G_0_ pupae of unknown sex and allowed the adults that eclosed to mate and produce eggs. We used the remaining 111 pupae for other experiments that ultimately were not fruitful. To increase numbers to generate the CRISPR-R2 strain described here, larvae were reared on untreated wheat germ diet for three generations. Because we previously found that resistance to Cry2Ab associated with mutations in *PgABCA2* is recessive^29,30^, rearing on untreated diet to G_2_ and beyond was also important to allow matings between mutant/wild-type heterozygotes to produce progeny that were homozygous for mutations in *PgABCA2*.

On six days in 2019 (25 and 27 February and 1, 4, 6 and 8 March), a total of 8 egg sheets (pieces of printer paper approximately 25 cm^2^) containing an estimated 6000 ± 1000 (mean ± standard error) eggs per egg sheet were pinned into 350 mL paper cups each with 100 g of wheat germ diet containing 3 μg Cry2Ab per mL diet. Over 21 d for each replicate, a total of 400 survivors were collected and used to establish the CRISPR-R2 colony. As a control, to measure survival in larvae not subjected to CRISPR editing, we set up an egg sheet containing approximately 50,000 eggs of the APHIS-S strain on 100 g of diet containing the same concentration of Cry2Ab.

We tested G_10_ larvae from the CRISPR-R2 strain for susceptibility to Cry2Ab using our standard diet incorporation bioassay^43–45^. We tested one neonate per well in bioassay trays (BIO-BA-128, Pitman, NJ) containing ~ 1 g of diet per well. Trays were covered with Pull N' Peel covers (BIO-CU-16, Pitman, NJ). We scored live fourth instars, pupae, and adults as survivors after 21 days. We calculated adjusted survival (%) as the survival (%) on diet containing 3 μg Cry2Ab per mL diet (n = 32) divided by survival (%) on untreated diet (81%, n = 16). In a simultaneous test, survival of the susceptible strain APHIS-S was 87.5% on untreated diet (n = 16) and 0% on diet containing 3 μg Cry2Ab per mL diet 3 µg Cry2Ab per mL diet (n = 16).

### Cas9-induced mutations in gDNA target regions

To determine if G_4_ larvae of CRISPR-R2 that survived on diet containing 3 μg Cry2Ab per mL diet harbored mutations corresponding to sgRNA target sites, we PCR amplified and directly Sanger sequenced the relevant regions of *PgABCA2* gDNA from 20 survivors. We extracted gDNA from the head of each larva separately using the Gentra Puregene Tissue Kit (Qiagen). *PgABCA2* gDNA fragments were PCR amplified using the Phire Hot Start II DNA Polymerase from the Phire Animal Tissue Direct PCR kit (Thermo Fisher Scientific) and gene-specific oligonucleotide primers (Supplementary Table S2) corresponding to 104PgABCA2-5 + 82PgABC3 (for sgRNA1), 178PgABCA2-5 + 192PgABCA2-3 (for sgRNA2 and − 3, 83PgABC5 + 190PgABCA2-3 (for sgRNA4), and 53PgABCA2-5 + 139PgABCA2-3 (for sgRNA5). Aliquots of each PCR reaction were separated on 1.5% agarose gels and stained with SYBR Safe DNA Gel Stain (Thermo Fisher Scientific) to confirm the presence of product. PCR products were treated with ExoSAP-IT reagent (Thermo Fisher Scientific) and directly Sanger sequenced by Retrogen (San Diego, CA).

### Single-pair crosses between CRISPR-R2 and APHIS-S

We used previously described methods^29^ to perform single-pair crosses between the CRISPR-R2 (G_16_) and the susceptible APHIS-S strain. In each of 40 cups (30 mL plastic Solo cups, Dart Container Cooperation, Mason, MI), we put one female CRISPR-R2 pupa and one male APHIS-S pupa. In each of another 40 cups, we put one female APHIS-S pupa and one male CRISPR-R2 pupa. After adults eclosed, lids were replaced with new ones containing a vial of 15% sucrose and semi-circle paper sheets that fit inside the lids were provided for oviposition. Eggs from the F_1_ progeny were collected and reared to adulthood on untreated diet. The F_2_ neonates from each of 10 hybrid families (A-E, male CRISPR-R2 X female APHIS-S; F-J, female CRISPR-R2 X male APHIS-S) were separately reared on diet containing 3 μg Cry2Ab per mL diet. Nine of the 10 families produced hundreds of survivors from which nine lines were established and reared on untreated diet. One family (I) produced only six pupae and was lost. As described below, we extracted RNA and sequenced *PgABCA2* cDNA from two larvae per family of the F_2_ generation from each of three families (A, C and J).

### Sequencing *PgABCA2* cDNA of Cry2Ab-resistant larvae from CRISPR-R2 and F_2_ progeny from CRISPR-R2 X APHIS-S

To assess mutations in RNA, we PCR amplified near full-length *PgABCA2* cDNA from five 4^th^ instar larvae that survived on 3 μg Cry2Ab per mL diet and did not show mutations in gDNA corresponding to sgRNA target sites; and from five 4th instar larvae on Cry2Ab from three single-pair CRISPR-R2 X APHIS-S families (A, C and J, see above). Total RNA was extracted using TRI Reagent (Thermo Fisher Scientific) and treated with DNase I (Thermo Fisher Scientific). cDNA was prepared using 2 μg of total RNA with both random hexamer primers and oligo-dT primers with a SuperScript IV reverse transcriptase (Thermo Fisher Scientific) according to the manufacturer’s instruction. *PgABCA2* cDNA was amplified in PCR using Platinum SuperFi Green PCR Master Mix (Thermo Fisher Scientific) and 1 μM gene-specific oligonucleotide primers 163PgABCA2-5 and 166PgABCA2-3 (Supplementary Table S2) at: 98 °C for 30 s (1 cycle); 40 cycles of 98 °C for 10 s, 50.9 °C for 10 s and 72 °C for 6 min; then 72 °C for 10 min. PCR products were separated on 1% agarose gels stained with SYBR Safe (Thermo Fisher Scientific) and viewed using an LED UV Illuminator (Maestrogen, Hsinchu City, Taiwan). Bands were gel-purified using the Montage DNA Gel Extraction kit (EMD Millipore/Merck KGaA, Darmstadt, Germany) and ligated into pCR-XL-2-TOPO (Thermo Fisher Scientific). Plasmids were propagated in TOP10 OmniMax One Shot Chemically Competent *E. coli* by growing on LB agar plates containing 50 μg per mL Carbenicillin (Research Products International, Mount Prospect, IL) for up to 72 h at room temperature. Colonies were screened using PCR and plasmid DNA was purified using a QIAprep Spin MiniPrep kit with a QIAcube system (Qiagen, Hilden, Germany). Inserts were sequenced by Retrogen (San Diego, CA) with primers as previously described^29^. *PgABCA2* coding sequences obtained from the CRISPR-R2 strain are deposited in the GenBank public database (Accession numbers MW523108-MW523137). Multiple sequence alignments were performed using Clustal Omega^46^ or MUSCLE (v3.8)^47^.

### Statistical analysis

We used Fisher’s exact test to determine if survival to pupation on diet containing 3 μg Cry2Ab per mL diet differed significantly between eggs from the G_4_ insects descended from CRISPR-treated G_0_ insects of APHIS-S and control eggs from APHIS-S. We first performed this test using our best estimates of sample sizes for the treated eggs (48,000) and control eggs (50,000). In addition, to provide an extremely conservative statistical test, we also performed this analysis with the sample size for the treated eggs purposely overestimated (multiplied by 5 = 240,000) and the sample size for the control eggs purposely underestimated (divided by 5 = 10,000). In tests with our best estimates and the purposely altered estimates, with 400 survivors observed in the treated group and none in the controls, the difference was significant with P < 0.00001. These results imply that the conclusion survival was higher in the treated than control eggs is exceptionally robust.

To estimate the frequency of resistance alleles in the G_4_, we assumed all G_4_ survivors on diet containing 3 μg Cry2Ab per mL diet were homozygous for resistance based on previous data showing that recessive mutations in *PgABCA2* are associated with resistance to Cry2Ab^29,30^. Based on the assumption of Hardy–Weinberg equilibrium, we calculated the frequency of resistance alleles at *PgABCA2* as the square root of the frequency of survivors. We used an analogous approach to estimate the frequency of the specific Cry2Ab resistance allele introduced by CRISPR in *H. armigera* based on the frequency of individuals homozygous for this mutation in the G_1_^22^. We used the frequency of mutant/wild-type heterozygotes to estimate the frequency of the specific resistance alleles at *PxABCC2*, *PxABCC3*, or both in the G_1_ of *P. xylostella*^31^. For example, in 24 months, one was a heterozygote for the *PxABCC2* mutation and 23 lacked the mutation, yielding a resistance allele frequency of 0.021 (one resistant allele per 48 alleles).

## Supplementary Information

Supplementary Information.
