# Supplementary material for: CRISPR-mediated mutations in the ABC transporter gene ABCA2 confer pink bollworm resistance to Bt toxin Cry2Ab
Source: Sci Rep. 2021 May 17;11:10377. doi: 10.1038/s41598-021-89771-7 (PMC8128902; doi:10.1038/s41598-021-89771-7)
Supplement: Supplementary file 1 — Supplementary Information. [file 41598_2021_89771_MOESM1_ESM.docx]

**SUPPLEMENTARY INFORMATION**

**CRISPR-mediated mutations in the ABC transporter gene *ABCA2* confer pink bollworm resistance to Bt toxin Cry2Ab**

Jeffrey A. Fabrick^1,*^, Dannialle M. LeRoy^1^, Lolita G. Mathew^1,‡^, Yidong Wu^2^, Gopalan C. Unnithan^3^, Alex J. Yelich^3^, Yves Carrière^3^, Xianchun Li^3^, and Bruce E. Tabashnik^3^

^1^ USDA ARS, U.S. Arid Land Agricultural Research Center, Maricopa, AZ 85138 USA

^2^ College of Plant Protection, Nanjing Agricultural University, Nanjing 210095, China

^3^ Department of Entomology, University of Arizona, Tucson, AZ 85721 USA

^*^ Corresponding author:

Jeffrey A. Fabrick

USDA ARS, U.S. Arid Land Agricultural Research Center

21881 N. Cardon Lane

Maricopa, AZ 85138, USA

Phone: 520-316-6335

Email: [jeff.fabrick@usda.gov](mailto:jeff.fabrick@usda.gov)

**^‡^** Current affiliation: Pairwise Plants, Durham, NC 27701

**SUPPLEMENTARY INFORMATION includes:**

**Supplementary Tables S1-S4**

**Supplementary Figures S1-S7**

**Supplementary Table S1. *PgABCA2* single guide RNAs (sgRNAs).**

| **Oligo** | **Sequence (5' to 3')^1^** | **Position^2^** | **Exon** | **Direction** |
| --- | --- | --- | --- | --- |
| sgRNA 1 | ACATAAGCTAATACGACTCACTATA**GGATACGACGTCCCTCAATA**GTTTTAGAGCTAGAAATAGCAAGTTAAAATAAGGCTAGTCCGTTATCAACTTGAAAAAGTGGCACCGAGTCGGTGCTTTT | 293-312 | 2 | Antisense |
| sgRNA 2 | ACATAAGCTAATACGACTCACTATA**GGTCCGGGAACTTCGAACAT**GTTTTAGAGCTAGAAATAGCAAGTTAAAATAAGGCTAGTCCGTTATCAACTTGAAAAAGTGGCACCGAGTCGGTGCTTTT | 676-695 | 3 | Antisense |
| sgRNA 3 | ACATAAGCTAATACGACTCACTATA**GGATCGTATAATATTGATAG**GTTTTAGAGCTAGAAATAGCAAGTTAAAATAAGGCTAGTCCGTTATCAACTTGAAAAAGTGGCACCGAGTCGGTGCTTTT | 929-948 | 4 | Antisense |
| sgRNA 4 | ACATAAGCTAATACGACTCACTATA**GGCGGAGTTGAGCATTAGGC**GTTTTAGAGCTAGAAATAGCAAGTTAAAATAAGGCTAGTCCGTTATCAACTTGAAAAAGTGGCACCGAGTCGGTGCTTTT | 1,343-1,362 | 7 | Antisense |
| sgRNA 5 | ACATAAGCTAATACGACTCACTATA**GGAGTGGGCGTCGCGCTATG**GTTTTAGAGCTAGAAATAGCAAGTTAAAATAAGGCTAGTCCGTTATCAACTTGAAAAAGTGGCACCGAGTCGGTGCTTTT | 2,113-2,131 | 12 | Sense |

^1^ Sequence corresponding to the T7 RNA polymerase binding site (dashed underline); *PgABCA2*-specific sgRNA sequence (bold); common stem-loop tracrRNA sequence (underline).

^2^ Sequence position within *PgABCA2* cDNA (MG637361).

**Supplementary Table S2. Nucleotide primers used to amplify, genotype, and/or DNA sequence *PgABCA2*.**

| **Primer** | | **Sequence** | | **Direction** | | **Application** | |
| --- | --- | --- | --- | --- | --- | --- | --- |
| 104PgABCA2-5 | | 5' - AGGCGGGCTCATGGGTGAA - 3' | | Sense | | Exon 1 primer for amplification of gDNA for Cas9 *in vitro* screen and PCR amplicon DNA sequencing | |
| 82PgABC3 | | 5' - ATTCTATGCCAGCTATCACCCTTCTC - 3' | | Antisense | | Exon 2 primer for amplification of gDNA for Cas9 *in vitro* screen and PCR amplicon DNA sequencing | |
| 178PgABCA2-5 | | 5' - GAGCTACCACTAGACTTGTCGTATGC - 3' | | Sense | | Exon 3 primer for amplification of gDNA for Cas9 *in vitro* screen and PCR amplicon DNA sequencing | |
| 174PgABCA2-5 | | 5' - CATGGACTTGGCGAAGGAAG - 3' | | Sense | | Exon 4 primer for amplification of gDNA for Cas9 *in vitro* screen | |
| 192PgABCA2-3 | | 5' - GGGAGTCCCATGATCTTCATC - 3' | | Antisense | | Exon 5 primer for amplification of gDNA for Cas9 *in vitro* screen and PCR amplicon DNA sequencing | |
| 83PgABCA5 | | 5' - GTTGTTTGGTGGGGTGATCT - 3' | | Sense | | Exon 7 primer for amplification of gDNA for Cas9 *in vitro* screen and PCR amplicon DNA sequencing | |
| 190PgABCA2-3 | | 5' - ATTATAACGACGTGACCGAATAC - 3' | | Antisense | | Exon 8 primer for amplification of gDNA for Cas9 *in vitro* screen and PCR amplicon DNA sequencing | |
| 53PgABCA2-5 | | 5' - CTGAAAGGCTTCAGCGGTAA - 3' | | Sense | | Exon 11 primer for amplification of gDNA for Cas9 *in vitro* screen and PCR amplicon DNA sequencing | |
| 139PgABCA2-3 | | 5' - AACAAGTCCCATAGGGCAC - 3' | | Antisense | | Exon 12 primer for amplification of gDNA for Cas9 *in vitro* screen and PCR amplicon DNA sequencing | |
| 163PgABCA2-5 | | 5' - GAATTTGGATTTGAGGCG - 3' | | Sense | | 5'-UTR primer for amplifying & cloning near full-length cDNA | |
| 166PgABCA2-3 | | 5' - TCGAGGGTAGTTTGTGAT - 3' | | Antisense | | Exon 31 primer for amplification & cloning near full-length cDNA | |
|  |  | |  | |  | |

**Supplementary Table S3. *PgABCA2* cDNA from five CRISPR-R2 G_4_ survivors on 3 μg Cry2Ab per mL lacking gDNA mutations corresponding to sgRNA target sites.**

| Individual^a^ | No. Clones Sequenced | Clone Sizes (bp) | Translation of ORF (aa) | Premature stop (exon #)^b^ |
| --- | --- | --- | --- | --- |
| 3 | 4 | 2,275, 2,279, 2,427, 2,544 | 80, 94, 100(2) | 2, 18 |
| 5 | 6 | 3,817, 3,960(2), 4,859, 4,960, 4,964 | 84(3), 1,045, 1,111, 1,146 | 8, 11, 12, 18, 21 |
| 9 | 7 | 3,301(2), 3,302(2), 4,043, 3,176, 3,363 | 129, 135(6) | 12, 13, 21, 26, |
| 10 | 8 | 810, 1,488(3), 2,033, 4,820(2), 4,859 | 270, 372(2), 496(3), 587, 1,111 | 7, 21, 29 |
| 18 | 3 | 5,426, 5,427(2) | 1,219(3) | 20 |

^a^ Five larvae from the CRISPR-R2 strain (3, 5, 9, 10 and 18) survived bioassays on 3 μg Cry2A.127 per mL diet and had no mutations detected in gDNA corresponding to *PgABCA2* sgRNA target sites 1-5.

^b^ Disrupted exons in the coding sequence.

**Supplementary Table S4. *PgABCA2* cDNA from six CRISPR-R2 X APHIS-S F_2_ progeny.**

| Cross^a^ | Larvae.clone^b^ | Sex | Clone Size (bp) | Translation of ORF (aa) | Premature stop (exon #)^c^ |
| --- | --- | --- | --- | --- | --- |
| A | 1.1 | Female | 4,859 | 1,111 | 21 |
|  | 2.2  2.9 | Male  Male | 4,859  4,765 | 1,111  372 | 21  7, 21 |
| C | 3.4  3.6  4.6 | Female  Female  Male | 4,870  5,622  5,025 | 372  1,219  99 | 7, 21  20, 27  2, 3, 7, 12, 18 |
| J | 4.8  4.10  5.2  5.3  5.6 | Male  Male  Female  Female  Female | 5,123  5,122  3,720  3,720  3,720 | 99  99  107  107  107 | 2, 3, 12  2, 3, 12  2, 3  2, 3  2, 3 |
|  | 6.4 | Male | 3,720 | 107 | 2, 3 |

^a^ Twelve *PgABCA2* cDNA clones from six F_2_ progeny of three single-pair families from CRISPR-RS X APHIS-S crosses (A, C and J)

^b^ First number indicates the larva and the second number indicates the clone (e.g., 1.1 indicates larva 1, clone 1).

^c^ Disrupted exons in the coding sequence.

**Supplementary Figure S1. *In vitro* screen of sgRNAs by Cas9 cleavage of *PgABCA2* PCR gDNA amplicons.** Cas9/sgRNA ribonucleotide mixtures incubated with *PgABCA2* gDNA PCR products were separated by 1.5% agarose gel electrophoresis. Negative (-) and positive (+) symbols indicate the absence or presence of Cas9, respectively. Arrows indicate primary bands resulting from Cas9 cleavage (red) or no Cas9 cleavage (blue).


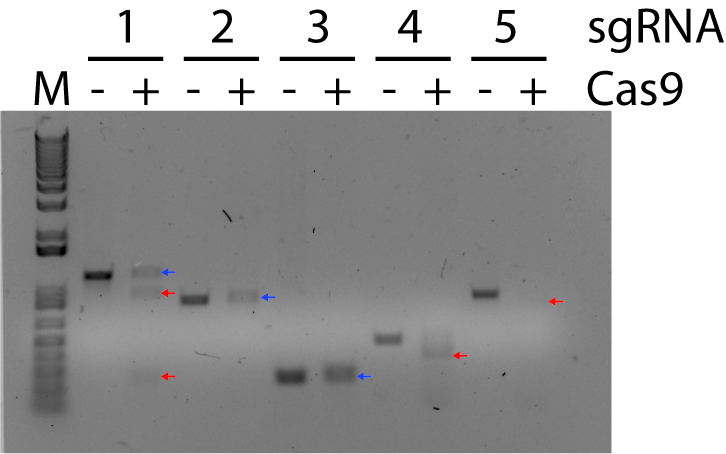


**Supplementary Figure S2. Unprocessed agarose gel showing *in vitro* screen of sgRNAs by Cas9 cleavage of *PgABCA2* PCR gDNA amplicons.** Cas9/sgRNA ribonucleotide mixtures incubated with *PgABCA2* gDNA PCR products were separated by 1.5% agarose gel electrophoresis. Negative (-) and positive (+) symbols indicate the absence or presence of Cas9, respectively.
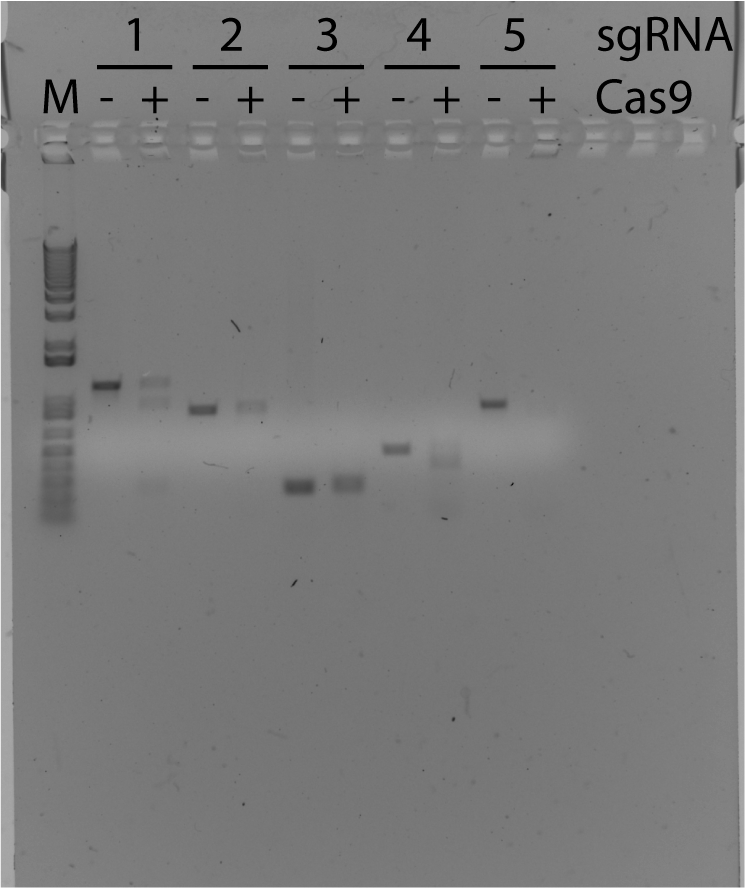


**Supplementary Figure S3. Partial genomic DNA sequencing of *PgABCA2* corresponding to sgRNA target sites from G_4_ survivors on 3 μg Cry2Ab per mL diet.** A. Twenty gDNA sequences from 104PgABCA2-5 + 82PgABC3 PCR products (the numbers to the right of the underscore refer to the individual) are aligned with the corresponding wild-type *PgABCA2* (MG637361.1) from susceptible strain APHIS-S. sgRNA1 is located in exon 2 of *PgABCA2* is highlighted in magenta. B. Nineteen gDNA sequences from 178PgABCA2-5 + 192PgABCA2-3 PCR products are aligned with the corresponding wild-type *PgABCA2*. sgRNAs 2 and -3 are located in *PgABCA2* exons 3 and 4 and are highlighted in cyan and teal, respectively. C. Nineteen gDNA sequences from 83PgABC5 + 190PgABCA2-3 PCR products are aligned with the corresponding wild-type *PgABCA2*. sgRNA4 is located in *PgABCA2* exon 7 and is highlighted in yellow. D. Eighteen gDNA sequences from 53PgABCA2-5 + 139PgABCA2-3 PCR products are aligned with corresponding wild-type *PgABCA2*. sgRNA5 is located in exon 12 of *PgABCA2* is highlighted in red. Stars show bases conserved in all sequences. Exon/intron splice sites are shown in gray and all intron sequences were removed to simplify alignments. Sequence of primers used to amplify gDNA fragments near sgRNA sites are in bold (note that in some cases primers are not shown if they are located within an adjacent exon and separated by intron). The order of sequences is based on their similarity determined by Clustal Omega (<https://www.ebi.ac.uk/Tools/msa/clustalo/>).

**A**

104+82_14 -------CAAGTATAGGCGCGGCANGATCATAAGAGGAAGGTTCGTGGCAGTCTCACGAG

104+82_12 ---GGCGCAATGTATAGGGCCGGAATGTACTTAACAATATCACCCGAGGCANTNTCANCG

104+82_19 -GGCGGAA-----------------TGTACTTAACAANNTCATCCGTGGCATTCTCACCG

104+82_7 GGGCGGACAGGAATTTCCAAGGCATTNTCNGGCGATNTCGCGTGAGAAAAGTCACAGAAG

104+82_1 GGGCCGAA--TTATTGGGGCAGGAAGGTTCATGCCAGTATCTCCAGGGCATTCTCACCTG

104+82_8 GGGACGAA--TTATAGGGGCGGGAAGAATCTAACCAGTATCTCCAGGGCATTCTCACCCG

104+82_17 GGGGGGGG--NGNNAATATCACGAAAATGCACAGCACTTTCTCACGAGAGAACGGTATAG

104+82_16 --TAGGAA--TGAATTTATTATAGAGGCGCAATGCATTTTCCCCGGAGGTCCATGTCATG

104+82_2 -GGCGGAA--GGAATTTCACAAGAATGTCCATGGCGATNTCATCGGAGAAGTCACTAGAG

104+82_6 -GGCGGAA--GGAATTTCACAGCATTGTCCATGGCGATTTCGTCGGAGAAGTCACTAGAG

104+82_15 --GCGGNA--AGAATTTAACAAGAATCTCCGCGGNNTCATCACCGGAGACTG-CAGTATG

104+82_11 -GGCGGAA--TGAATTTAACAAGAATATCCATGGCATTNTCACCCGAGAATGCGGTATTG

104+82_20 -GGCGGAA--TGAATTTAACAAGAATGTCCATGGCATTCTCACCCGAGAATGGCG---AG

104+82_13 --GCGGAA--TGAATTTAACAAGAATGTCCATGGCATTCTCACCCGAGAATGCCGTATTG

104+82_9 --GCGGAA--TGAATTTAACAAGAATGTCCATGGCATTCTCACCCGAGAATGCCGTATTG

104+82_5 -GGCGGAA--TGAATTTAACAAGAATGTCCATGGCATTCTCACCCGAGAATGCCGTATTG

APHIS-S_104+82 -GGCGGAA--TGAATTTAACAAGAATGTCCATGGCATTCTCACCCGAGAATGCCGTATTG

104+82_3 -GGCGGAA--TGAATTTAACAAGAATGTCCATGGCATTCTCACCCGAGAATGCCGTATTG

104+82_4 -GGCGGAA--TGAATTTAACAAGAATGTCCATGGCATTCTCACCCGAGAATGCCGTATTG

104+82_10 -GGCGGAA--TGAATTTAACAAGAATGTCCATGGCATTCTCACCCGAGAATGCCGTATTG

104+82_18 -GGCGGAA--TGAATTTAACAAGAATGTCCATGGCATTCTCACCCGAGAATGCCGTATTG

*

104+82_14 AGGGAAGTCGTATCCAGTGCTACAACAAAGTTACTGCTTAAAAACATGAGAGACCAAGTA

104+82_12 GGGAATGTCGTATCCAGTGCTACAACAAAGTTACTGCTTAAAAACATGAGAGACCAAGTA

104+82_19 GGGAATGTCGTATCCAGTGCTACAACAAAGTTACTGCTTAAAAACATGAGAGACCAAGTA

104+82_7 AGGGAAGTCGTATCCAGTGCTACAACAAAGTTACTGCTTAAAAACATGAGAGACCAAGTA

104+82_1 AGGGACGTCGTATCCAGTGCTACAACAAAGTTACTGCTTAAAAACATGAGAGACCAAGTA

104+82_8 AGGGACGTCGTATCCAGTGCTACAACAAAGTTACTGCTTAAAAACATGAGAGACCAAGTA

104+82_17 AGGGACGTCGTATCCAGTGCTACAACAAAGTTACTGCTTAAAAACATGAGAGACCAAGTA

104+82_16 AGGGACGTCGTATCCAGTGCTACAACAAAGTTACTGCTTAAAAACATGAGAGACCAAGTA

104+82_2 AGGGAAGTCGTATCCAGTGCTACAACAAAGTTACTGCTTAAAAACATGAGAGACCAAGTA

104+82_6 GGGGAAGTCGTATCCAGTGCTACAACAAAGTTACTGCTTAAAAACATGAGAGACCAAGTA

104+82_15 AGGGACGTCGTATCCAGTGCTACAACAAAGTTACTGCTTAAAAACATGAGAGACCAAGTA

104+82_11 AGGGACGTCGTATCCAGTGCTACAACAAAGTTACTGCTTAAAAACATGAGAGACCAAGTA

104+82_20 AGGGACGTCGTATCCAGTGCTACAACAAAGTTACTGCTTAAAAACATGAGAGACCAAGTA

104+82_13 AGGGACGTCGTATCCAGTGCTACAACAAAGTTACTGCTTAAAAACATGAGAGACCAAGTA

104+82_9 AGGGACGTCGTATCCAGTGCTACAACAAAGTTACTGCTTAAAAACATGAGAGACCAAGTA

104+82_5 AGGGACGTCGTATCCAGTGCTACAACAAAGTTACTGCTTAAAAACATGAGAGACCAAGTA

APHIS-S_104+82 AGGGACGTCGTATCCAGTGCTACAACAAAGTTACTGCTTAAAAACATGAGAGACCAAGTA

104+82_3 AGGGACGTCGTATCCAGTGCTACAACAAAGTTACTGCTTAAAAACATGAGAGACCAAGTA

104+82_4 AGGGACGTCGTATCCAGTGCTACAACAAAGTTACTGCTTAAAAACATGAGAGACCAAGTA

104+82_10 AGGGACGTCGTATCCAGTGCTACAACAAAGTTACTGCTTAAAAACATGAGAGACCAAGTA

104+82_18 AGGGACGTCGTATCCAGTGCTACAACAAAGTTACTGCTTAAAAACATGAGAGACCAAGTA

** * ******************************************************

104+82_14 CTGCCCATCATTGAGGCATTGCCAATAGAAATACCGCCGGGACTGGTAAACTCGTCACAG

104+82_12 CTGCCCATCATTGAGGCATTGCCAATAGAAATACCGCCGGGACTGGTAAACTCGTCACAG

104+82_19 CTGCCCATCATTGAGGCATTGCCAATAGAAATACCGCCGGGACTGGTAAACTCGTCACAG

104+82_7 CTGCCCATCATTGAGGCATTGCCAATAGAAATACCGCCGGGACTGGTAAACTCGTCACAG

104+82_1 CTGCCCATCATTGAGGCATTGCCAATAGAAATACCGCCGGGACTGGTAAACTCGTCACAG

104+82_8 CTGCCCATCATTGAGGCATTGCCAATAGAAATACCGCCGGGACTGGTAAACTCGTCACAG

104+82_17 CTGCCCATCATTGAGGCATTGCCAATAGAAATACCGCCGGGACTGGTAAACTCGTCACAG

104+82_16 CTGCCCATCATTGAGGCATTGCCAATAGAAATACCGCCGGGACTGGTAAACTCGTCACAG

104+82_2 CTGCCCATCATTGAGGCATTGCCAATAGAAATACCGCCGGGACTGGTAAACTCGTCACAG

104+82_6 CTGCCCATCATTGAGGCATTGCCAATAGAAATACCGCCGGGACTGGTAAACTCGTCACAG

104+82_15 CTGCCCATCATTGAGGCATTGCCAATAGAAATACCGCCGGGACTGGTAAACTCGTCACAG

104+82_11 CTGTCCATCATTGAGGCATTGCCAATAGAAATACCGCCGGGACTGGTAAACTCGTCACAG

104+82_20 CTGCCCATCATTGAGGCATTGCCAATAGAAATACCGCCGGGACTGGTAAACTCGTCACAG

104+82_13 CTGCCCATCATTGAGGCATTGCCAATAGAAATACCGCCGGGACTGGTAAACTCGTCACAG

104+82_9 CTGCCCATCATTGAGGCATTGCCAATAGAAATACCGCCGGGACTGGTAAACTCGTCACAG

104+82_5 CTGCCCATCATTGAGGCATTGCCAATAGAAATACCGCCGGGACTGGTAAACTCGTCACAG

APHIS-S_104+82 CTGCCCATCATTGAGGCATTGCCAATAGAAATACCGCCGGGACTGGTAAACTCGTCACAG

104+82_3 CTGCCCATCATTGAGGCATTGCCAATAGAAATACCGCCGGGACTGGTAAACTCGTCACAG

104+82_4 CTGCCCATCATTGAGGCATTGCCAATAGAAATACCGCCGGGACTGGTAAACTCGTCACAG

104+82_10 CTGCCCATCATTGAGGCATTGCCAATAGAAATACCGCCGGGACTGGTAAACTCGTCACAG

104+82_18 CTGCCCATCATTGAGGCATTGCCAATAGAAATACCGCCGGGACTGGTAAACTCGTCACAG

*** ********************************************************

104+82_14 GTGTACGAAATAGTTAAAT-TATTTGTCGACGAGAACGTTGTTACCGGATACAATAGCAG

104+82_12 GTGTACGAAATANNNAAAT-TATTTGTCGACGAGAACGTTGTTACCGGATACAATAGCAG

104+82_19 GTGTACGAAATAGTTAAAT-TATTTGTCGACGAGAACGTTGTTACCGGATACAATAGCAG

104+82_7 GTGTACGAAATAGTTAAAT-TATTTGTCGACGAGAACGTTGTTACCGGATACAATAGCAG

104+82_1 GTGTACGAAATAGTTAAAT-TATTTGTCGACGAGAACGTTGTTACCGGATACAATAGCAG

104+82_8 GTGTACGAAATAGTTAAAT-TATTTGTCGACGAGAACGTTGTTACCGGATACAATAGCAG

104+82_17 GTGTACGAAATAGTTAAAT-TATTTGTCGACGAGAACGTTGTTACCGGATACAATAGCAG

104+82_16 GTGTACGAAATAGTTAAAT-TATTTGTCGACGAGAACGTTGTTACCGGATACAATAGCAG

104+82_2 GTGTACGAAATAGTTAAAT-TATTTGTCGACGAGAACGTTGTTACCGGATACAATAGCAG

104+82_6 GTGTACGAAATAGTTAAAT-TATTTGTCGACGAGAACGTTGTTACCGGATACAATAGCAG

104+82_15 GTGTACGAAATAGTTAAAT-TATTTGTCGACGAGAACGTTGTTACCGGATACAATAGCAG

104+82_11 GTGTACGAAATAGTTNNAATTATTTGTCGACGAGAACGNNGTTACCGGATNCAATAAGCA

104+82_20 GTGTACGAAATAGTNAANT-TATTTGTCGACGAGAACGTTGTTACCGGATACANTAGCAG

104+82_13 GTGTACGAAATAGTTAAAT-TATTTGTCGACGAGAACGTTGTTACCGGATACAATAGCAG

104+82_9 GTGTACGAAATAGTTAAAT-TATTTGTCGACGAGAACGTTGTTACCGGATACAATAGCAG

104+82_5 GTGTACGAAATAGTTAAAT-TATTTGTCGACGAGAACGTTGTTACCGGATACAATAGCAG

APHIS-S_104+82 GTGTACGAAATAGTTAAAT-TATTTGTCGACGAGAACGTTGTTACCGGATACAATAGCAG

104+82_3 GTGTACGAAATAGTTAAAT-TATTTGTCGACGAGAACGTTGTTACCGGATACAATAGCAG

104+82_4 GTGTACGAAATAGTTAAAT-TATTTGTCGACGAGAACGTTGTTACCGGATACAATAGCAG

104+82_10 GTGTACGAAATAGTTAAAT-TATTTGTCGACGAGAACGTTGTTACCGGATACAATAGCAG

104+82_18 GTGTACGAAATAGTTAAAT-TATTTGTCGACGAGAACGTTGTTACCGGATACAATAGCAG

************ ****************** ********** ** **

104+82_14 TGCGGCAANGAGAGGA--------------------------------------------

104+82_12 TGCGGCAA----------------------------------------------------

104+82_19 TGCGGCAA----------------------------------------------------

104+82_7 NGCGGCAA----------------------------------------------------

104+82_1 TGCGGCAA----------------------------------------------------

104+82_8 TGCGGCAAGAGA------------------------------------------------

104+82_17 TGCGGCTAANGAGAG---------------------------------------------

104+82_16 TGCGGCAA----------------------------------------------------

104+82_2 TGCGGCA-----------------------------------------------------

104+82_6 TGCGGCAA----------------------------------------------------

104+82_15 TGCGGCAA----------------------------------------------------

104+82_11 GTGCGGC-----------------------------------------------------

104+82_20 TGCGG-------------------------------------------------------

104+82_13 TGCGGCTAA---------------------------------------------------

104+82_9 TGCGGCAA----------------------------------------------------

104+82_5 TGCGGCAATGAGAGGAATAA----------------------------------------

APHIS-S_104+82 TGCGGCAATGAGAGGAATATACGCAGAGGAAGAAGCCAC**GAGAAGGGTGATAGCTGGCAT**

104+82_3 TGCG--------------------------------------------------------

104+82_4 TGCGGCAA----------------------------------------------------

104+82_10 TGCGGC------------------------------------------------------

104+82_18 TGCGGCAA----------------------------------------------------

104+82_14 -----

104+82_12 -----

104+82_19 -----

104+82_7 -----

104+82_1 -----

104+82_8 -----

104+82_17 -----

104+82_16 -----

104+82_2 -----

104+82_6 -----

104+82_15 -----

104+82_11 -----

104+82_20 -----

104+82_13 -----

104+82_9 -----

104+82_5 -----

APHIS-S_104+82 **AGAAT**

104+82_3 -----

104+82_4 -----

104+82_10 -----

104+82_18 -----

**B**

178+192_10 ------------------------------------------------------------

178+192_6 ----------------------------TTCGTATTCCGGAGAGACCTCGCTTGAATTCC

APHIS-S_178+192 **GAGCTACCACTAGACTTGTCGTATGC**GCTTCGTTTTCCGGAGAGACCTCGCTTGAATTCC

178+192_2 -----------------------------------TCCGGAGAGACCTCGCTTGAATTCC

178+192_3 -----------------------------TCGTTTTCCGGAGAGACCTCGCTTGAATTCC

178+192_4 -----------------------------TCGTTTTCCGGAGAGACCTCGCTTGAATTCC

178+192_5 --------------------------------TTTTCCGGAGAGACCTCGCTTGAATTCC

178+192_1 -------------------------------------------------------ATTCC

178+192_14 -----------------------------TCGTTTTCCGGAGAGACCTCGCTTGAATTCN

178+192_18 ---------------------------TTCGTTTTCCGGGAGAGACCTCGCTTGAATTCC

178+192_16 ----------TAGACTTGTCGTATGCGNTTCGTTTTCCGGAGAGACCTCGCTTGAATTCC

178+192_7 --------------------------------TTTTCCGGAGAGACCTCGCTTGAATTCC

178+192_8 -------------------------------------------------------ATTCC

178+192_9 -----------------------------TCGTTTTCCGGAGAGACCTCGCTTGAATTCC

178+192_11 -----------------------------TCGTTTTCCGGAGAGACCTCGCTTGAATTCC

178+192_12 -----------------------------TCGTTTTCCGGAGAGACCTCGCTTGAATTCC

178+192_13 ------------------TNGTATGCGNTTCGTTTTCCGGAGAGACCTCGCTTGAATTCC

178+192_15 ----------------------------TTCGTTTTCCGGAGAGACCTCGCTTGAATTCC

178+192_17 ---AACCCCCTAGACTTGTNGTATGCGNTTCGTTTTCCGGAGAGACCTCGCTTGAATTCC

178+192_19 --------------------------------TTTTCCGGAGAGACCTCGCTTGAATTCC

178+192_10 ---------------------------------GGTGCACAGATTCCTATGTTCGAAGTT

178+192_6 TTCTTCATGACAGGCGGTCGGACTTGGCGCACAGATAACGTGTTTCCTATGTTCGAAGTT

APHIS-S_178+192 TTCTTCATGACAGGCGGTCGGACTTGGCGCACAGATAACGTGTTTCCTATGTTCGAAGTT

178+192_2 TTCTTCATGACAGGCGGTCGGACTTGGCGCACAGATAACGTGTTTCCTATGTTCGAAGTT

178+192_3 TTCTTCATGACAGGCGGTCGGACTTGGCGCACAGATAACGTGTTTCCTATGTTCGAAGTT

178+192_4 TTCTTCATGACAGGCGGTCGGACTTGGCGCACAGATAACGTGTTTCCTATGTTCGAAGTT

178+192_5 TTCTTCATGACAGGCGGTCGGACTTGGCGCACAGATAACGTGTTTCCTATGTTCGAAGTT

178+192_1 TTCTTCATGACAGGCGGTCGGACTTGGCGCACAGATAACGTGTTTCCTATGTTCGAAGTT

178+192_14 TTCTTCATGACAGGCGGTCGGACTTGGCGCACAGATAACGTGTTTCCTATGTTCGAAGTT

178+192_18 TTCTTCATGACAGGCGGTCGGACTTGGCGCACAGATAACGTGTTTCCTATGTTCGAAGTT

178+192_16 TTCTTCATGACAGGCGGTCGGACTTGGCGCACAGATAACGTGTTTCCTATGTTCGAAGTT

178+192_7 TTCTTCATGACAGGCGGTCGGACTTGGCGCACAGATAACGTGTTTCCTATGTTCGAAGTT

178+192_8 TTCTTCATGACAGGCGGTCGGACTTGGCGCACAGATAACGTGTTTCCTATGTTCGAAGTT

178+192_9 TTCTTCATGACAGGCGGTCGGACTTGGCGCACAGATAACGTGTTTCCTATGTTCGAAGTT

178+192_11 TTCTTCATGACAGGCGGTCGGACTTGGCGCACAGATAACGTGTTTCCTATGTTCGAAGTT

178+192_12 TTCTTCATGACAGGCGGTCGGACTTGGCGCACAGATAACGTGTTTCCTATGTTCGAAGTT

178+192_13 TTCTTCATGACAGGCGGTCGGACTTGGCGCACAGATAACGTGTTTCCTATGTTCGAAGTT

178+192_15 TTCTTCATGACAGGCGGTCGGACTTGGCGCACAGATAACGTGTTTCCTATGTTCGAAGTT

178+192_17 TTCTTCATGACAGGCGGTCGGACTTGGCGCACAGATAACGTGTTTCCTATGTTCGAAGTT

178+192_19 TTCTTCATGACAGGCGGTCGGACTTGGCGCACAGATAACGTGTTTCCTATGTTCGAAGTT

* * * *****************

178+192_10 CCCGGACCTCGCTTTCCGTATTCATGGGAAGGTGGAAATG-------ATACGTAAACGAG

178+192_6 CCCGGACCTCGCTTTCCGTATTCATGGGAAGGTGGAAATGATCCAGGATACGTAAACGAG

APHIS-S_178+192 CCCGGACCTCGCTTTCCGTATTCATGGGAAGGTGGAAATGATCCAGGATACGTAAACGAG

178+192_2 CCCGGACCTCGCTTTCCGTATTCATGGGAAGGTGGAAATGATCCAGGATACGTAAACGAG

178+192_3 CCCGGACCTCGCTTTCCGTATTCATGGGAAGGTGGAAATGATCCAGGATACGTAAACGAG

178+192_4 CCCGGACCTCGCTTTCCGTATTCATGGGAAGGTGGAAATGATCCAGGATACGTAAACGAG

178+192_5 CCCGGACCTCGCTTTCCGTATTCATGGGAAGGTGGAAATGATCCAGGATACGTAAACGAG

178+192_1 CCCGGACCTCGCTTTCCGTATTCATGGGAAGGTGGAAATGATCCAGGATACGTAAACGAG

178+192_14 CCCGGACCTCGCTTTCCGTATTCATGGGAAGGTGGAAATG-------ATACGTAAACGAG

178+192_18 CCCGGACCTCGCTTTCCGTATTCATGGGAAGGTGGAAATG-------ATACGTAAACGAG

178+192_16 CCCGGACCTCGCTTTCCGTATTCATGGGAAGGTGGAAATG-------ATACGTAAACGAG

178+192_7 CCCGGACCTCGCTTTCCGTATTCATGGGAAGGTGGAAATG-------ATACGTAAACGAG

178+192_8 CCCGGACCTCGCTTTCCGTATTCATGGGAAGGTGGAAATG-------ATACGTAAACGAG

178+192_9 CCCGGACCTCGCTTTCCGTATTCATGGGAAGGTGGAAATG-------ATACGTAAACGAG

178+192_11 CCCGGACCTCGCTTTCCGTATTCATGGGAAGGTGGAAATG-------ATACGTAAACGAG

178+192_12 CCCGGACCTCGCTTTCCGTATTCATGGGAAGGTGGAAATG-------ATACGTAAACGAG

178+192_13 CCCGGACCTCGCTTTCCGTATTCATGGGAAGGTGGAAATG-------ATACGTAAACGAG

178+192_15 CCCGGACCTCGCTTTCCGTATTCATGGGAAGGTGGAAATG-------ATACGTAAACGAG

178+192_17 CCCGGACCTCGCTTTCCGTATTCATGGGAAGGTGGAAATG-------ATACGTAAACGAG

178+192_19 CCCGGACCTCGCTTTCCGTATTCATGGGAAGGTGGAAATG-------ATACGTAAACGAG

*************************************** *************

178+192_10 ATGTTCATAGCCTTGCAGCACATGATATCTTCAGAACTGGTATCTAAAGTGGCGGGAGTG

178+192_6 ATGTTCATAGCCTTGCAGCACATGATATCTTCAGAACTGGTATCTAAAGTGGCGGGAGTG

APHIS-S_178+192 ATGTTCATAGCCTTGCAGCACATGATATCTTCAGAACTGGTATCTAAAGTGGCGGGAGTG

178+192_2 ATGTTCATAGCCTTGCAGCACATGATATCTTCAGAACTGGTATCTAAAGTGGCGGGAGTG

178+192_3 ATGTTCATAGCCTTGCAGCACATGATATCTTCAGAACTGGTATCTAAAGTGGCGGGAGTG

178+192_4 ATGTTCATAGCCTTGCAGCACATGATATCTTCAGAACTGGTATCTAAAGTGGCGGGAGTG

178+192_5 ATGTTCATAGCCTTGCAGCACATGATATCTTCAGAACTGGTATCTAAAGTGGCGGGAGTG

178+192_1 ATGTTCATAGCCTTGCAGCACATGATATCTTCAGAACTGGTATCTAAAGTGGCGGGAGTG

178+192_14 ATGTTCATAGCCTTGCAGCACATGATATCTTCAGAACTGGTATCTAAAGTGGCGGGAGTG

178+192_18 ATGTTCATAGCCTTGCAGCACATGATATCTTCAGAACTGGTATCTAAAGTGGCGGGAGTG

178+192_16 ATGTTCATAGCCTTGCAGCACATGATATCTTCAGAACTGGTATCTAAAGTGGCGGGAGTG

178+192_7 ATGTTCATAGCCTTGCAGCACATGATATCTTCAGAACTGGTATCTAAAGTGGCGGGAGTG

178+192_8 ATGTTCATAGCCTTGCAGCACATGATATCTTCAGAACTGGTATCTAAAGTGGCGGGAGTG

178+192_9 ATGTTCATAGCCTTGCAGCACATGATATCTTCAGAACTGGTATCTAAAGTGGCGGGAGTG

178+192_11 ATGTTCATAGCCTTGCAGCACATGATATCTTCAGAACTGGTATCTAAAGTGGCGGGAGTG

178+192_12 ATGTTCATAGCCTTGCAGCACATGATATCTTCAGAACTGGTATCTAAAGTGGCGGGAGTG

178+192_13 ATGTTCATAGCCTTGCAGCACATGATATCTTCAGAACTGGTATCTAAAGTGGCGGGAGTG

178+192_15 ATGTTCATAGCCTTGCAGCACATGATATCTTCAGAACTGGTATCTAAAGTGGCGGGAGTG

178+192_17 ATGTTCATAGCCTTGCAGCACATGATATCTTCAGAACTGGTATCTAAAGTGGCGGGAGTG

178+192_19 ATGTTCATAGCCTTGCAGCACATGATATCTTCAGAACTGGTATCTAAAGTGGCGGGAGTG

************************************************************

178+192_10 AACCTAGACTTCGATGTGCACATACAGAGGTACCCACATCCAGCATACATCATGGACTTG

178+192_6 AACCTAGACTTCGATGTGCACATACAGAGGTACCCACATCCAGCATACATCATGGACTTG

APHIS-S_178+192 AACCTAGACTTCGATGTGCACATACAGAGGTACCCACATCCAGCATACATCATGGACTTG

178+192_2 AACCTAGACTTCGATGTGCACATACAGAGGTACCCACATCCAGCATACATCATGGACTTG

178+192_3 AACCTAGACTTCGATGTGCACATACAGAGGTACCCACATCCAGCATACATCATGGACTTG

178+192_4 AACCTAGACTTCGATGTGCACATACAGAGGTACCCACATCCAGCATACATCATGGACTTG

178+192_5 AACCTAGACTTCGATGTGCACATACAGAGGTACCCACATCCAGCATACATCATGGACTTG

178+192_1 AACCTAGACTTCGATGTGCACATACAGAGGTACCCACATCCAGCATACATCATGGACTTG

178+192_14 AACCTAGACTTCGATGTGCACATACAGAGGTACCCACATCCAGCATACATCATGGACTTG

178+192_18 AACCTAGACTTCGATGTGCACATACAGAGGTACCCACATCCAGCATACATCATGGACTTG

178+192_16 AACCTAGACTTCGATGTGCACATACAGAGGTACCCACATCCAGCATACATCATGGACTTG

178+192_7 AACCTAGACTTCGATGTGCACATACAGAGGTACCCACATCCAGCATACATCATGGACTTG

178+192_8 AACCTAGACTTCGATGTGCACATACAGAGGTACCCACATCCAGCATACATCATGGACTTG

178+192_9 AACCTAGACTTCGATGTGCACATACAGAGGTACCCACATCCAGCATACATCATGGACTTG

178+192_11 AACCTAGACTTCGATGTGCACATACAGAGGTACCCACATCCAGCATACATCATGGACTTG

178+192_12 AACCTAGACTTCGATGTGCACATACAGAGGTACCCACATCCAGCATACATCATGGACTTG

178+192_13 AACCTAGACTTCGATGTGCACATACAGAGGTACCCACATCCAGCATACATCATGGACTTG

178+192_15 AACCTAGACTTCGATGTGCACATACAGAGGTACCCACATCCAGCATACATCATGGACTTG

178+192_17 AACCTAGACTTCGATGTGCACATACAGAGGTACCCACATCCAGCATACATCATGGACTTG

178+192_19 AACCTAGACTTCGATGTGCACATACAGAGGTACCCACATCCAGCATACATCATGGACTTG

************************************************************

178+192_10 GCGAAGGAAGCCCTGCAGTTCCTCTTCCCATCATTCATCATGATCAGCTTCAGTTACACC

178+192_6 GCGAAGGAAGCCCTGCAGTTCCTCTTCCCATCATTCATCATGATCAGCTTCAGTTACACC

APHIS-S_178+192 GCGAAGGAAGCCCTGCAGTTCCTCTTCCCATCATTCATCATGATCAGCTTCAGTTACACC

178+192_2 GCGAAGGAAGCCCTGCAGTTCCTCTTCCCATCATTCATCATGATCAGCTTCAGTTACACC

178+192_3 GCGAAGGAAGCCCTGCAGTTCCTCTTCCCATCATTCATCATGATCAGCTTCAGTTACACC

178+192_4 GCGAAGGAAGCCCTGCAGTTCCTCTTCCCATCATTCATCATGATCAGCTTCAGTTACACC

178+192_5 GCGAAGGAAGCCCTGCAGTTCCTCTTCCCATCATTCATCATGATCAGCTTCAGTTACACC

178+192_1 GCGAAGGAAGCCCTGCAGTTCCTCTTCCCATCATTCATCATGATCAGCTTCAGTTACACC

178+192_14 GCGAAGGAAGCCCTGCAGTTCCTCTTCCCATCATTCATCATGATCAGCTTCAGTTACACC

178+192_18 GCGAAGGAAGCCCTGCAGTTCCTCTTCCCATCATTCATCATGATCAGCTTCAGTTACACC

178+192_16 GCGAAGGAAGCCCTGCAGTTCCTCTTCCCATCATTCATCATGATCAGCTTCAGTTACACC

178+192_7 GCGAAGGAAGCCCTGCAGTTCCTCTTCCCATCATTCATCATGATCAGCTTCAGTTACACC

178+192_8 GCGAAGGAAGCCCTGCAGTTCCTCTTCCCATCATTCATCATGATCAGCTTCAGTTACACC

178+192_9 GCGAAGGAAGCCCTGCAGTTCCTCTTCCCATCATTCATCATGATCAGCTTCAGTTACACC

178+192_11 GCGAAGGAAGCCCTGCAGTTCCTCTTCCCATCATTCATCATGATCAGCTTCAGTTACACC

178+192_12 GCGAAGGAAGCCCTGCAGTTCCTCTTCCCATCATTCATCATGATCAGCTTCAGTTACACC

178+192_13 GCGAAGGAAGCCCTGCAGTTCCTCTTCCCATCATTCATCATGATCAGCTTCAGTTACACC

178+192_15 GCGAAGGAAGCCCTGCAGTTCCTCTTCCCATCATTCATCATGATCAGCTTCAGTTACACC

178+192_17 GCGAAGGAAGCCCTGCAGTTCCTCTTCCCATCATTCATCATGATCAGCTTCAGTTACACC

178+192_19 GCGAAGGAAGCCCTGCAGTTCCTCTTCCCATCATTCATCATGATCAGCTTCAGTTACACC

************************************************************

178+192_10 GCTATCAATATTATACGATCCGTGACCGTGGAAAAAGAAATGCAATTGAAG---------

178+192_6 GCTATCAATATTATACGATCCGTGACCGTGGAAAAAGAAATGCAATTGAAG---------

APHIS-S_178+192 GCTATCAATATTATACGATCCGTGACCGTGGAAAAAGAAATGCAATTGAAGGAAAC**GATG**

178+192_2 GCTATCAATATTATACGATCCGTGACCGTGGAAAAAGAAATGCAATTGAAG---------

178+192_3 GCTATCAATATTATACGATCCGTGACCGTGGAAAAAGAAATGCAATTGAAG---------

178+192_4 GCTATCAATATTATACGATCCGTGACCGTGGAAAAAGAAATGCAATTGAAG---------

178+192_5 GCTATCAATATTATACGATCCGTGACCGTGGAAAAAGAAATGCAATTGAAG---------

178+192_1 GCTATCAATATTATACGATCCGTGACCGTGGAAAAAGAAATGCAATTGAAG---------

178+192_14 GCTATCAATATTATACGATCCGTGACCGTGGAAAAAGAAATGCAATTGAAG---------

178+192_18 GCTATCAATATTATACGATCCGTGACCGTGGAAAAAGAAATGCAATTGAAG---------

178+192_16 GCTATCAATATTATACGATCCGTGACCGTGGAAAAAGAAATGCAATTGAAG---------

178+192_7 GCTATCAATATTATACGATCCGTGACCGTGGAAAAAGAAATGCAATTGAAG---------

178+192_8 GCTATCAATATTATACGATCCGTGACCGTGGAAAAAGAAATGCAATTGAAG---------

178+192_9 GCTATCAATATTATACGATCCGTGACCGTGGAAAAAGAAATGCAATTGAAG---------

178+192_11 GCTATCAATATTATACGATCCGTGACCGTGGAAAAAGAAATGCAATTGAAG---------

178+192_12 GCTATCAATATTATACGATCCGTGACCGTGGAAAAAGAAATGCAATTGAAG---------

178+192_13 GCTATCAATATTATACGATCCGTGACCGTGGAAAAAGAAATGCAATTGAAG---------

178+192_15 GCTATCAATATTATACGATCCGTGACCGTGGAAAAAGAAATGCAATTGAAG---------

178+192_17 GCTATCAATATTATACGATCCGTGACCGTGGAAAAAGAAATGCAATTGAAG---------

178+192_19 GCTATCAATATTATACGATCCGTGACCGTGGAAAAAGAAATGCAATTGAAG---------

***************************************************

178+192_10 -----------------

178+192_6 -----------------

APHIS-S_178+192 **AAGATCATGGGACTCCC**

178+192_2 -----------------

178+192_3 -----------------

178+192_4 -----------------

178+192_5 -----------------

178+192_1 -----------------

178+192_14 -----------------

178+192_18 -----------------

178+192_16 -----------------

178+192_7 -----------------

178+192_8 -----------------

178+192_9 -----------------

178+192_11 -----------------

178+192_12 -----------------

178+192_13 -----------------

178+192_15 -----------------

178+192_17 -----------------

178+192_19 -----------------

**C**

83+190_4 -------------------------------TATATCCCCGCTCNCCNCGGGNATAGGGA

83+190_19 -------------GTGATCTGGTTTCTGACGTATATCCCCGCATTCCTCCTGGCTATGGA

83+190_18 -------------GTGATCTGGTTTCTGACGTATATCCCCGCATTCCTCCTGGCTATGGA

83+190_15 --------GTGGGGTGATCTGGTTTCTGACGTATATCCCCGCATTCCTCCTGGCTATGGA

83+190_12 ----------GGGGTGATCTGGTTTCTGACGTATATCCCCGCATTCCTCCTGGCTATGGA

83+190_6 -------------GTGATCTGGTTTCTGACGTATATCCCCGCATTCCTCCTGGCTATGGA

83+190_5 GTTGTTTGNTGGGGTGATCTGGTTTCTGACGTATATCCCCGCATTCCTCCTGGCTATGGA

APHIS-S_83+190 **GTTGTTTGGTGGGGTGATCT**GGTTTCTGACGTATATCCCCGCATTCCTCCTGGCTATGGA

83+190_2 GTTGTTTGGTGGGGTGATCTGGTTTCTGACGTATATCCCCGCATTCCTCCTGGCTATGGA

83+190_3 GTTGTTTGGTGGGGTGATCTGGTTTCTGACGTATATCCCCGCATTCCTCCTGGCTATGGA

83+190_1 -------------GTGATCTGGTTTCTGACGTATATCCCCGCATTCCTCCTGGCTATGGA

83+190_7 -------------GTGATCTGGTTTCTGACGTATATCCCCGCATTCCTCCTGGCTATGGA

83+190_8 ----------GGGGTGATCTGGTTTCTGACGTATATCCCCGCATTCCTCCTGGCTATGGA

83+190_9 ----------GGGGTGATCTGGTTTCTGACGTATATCCCCGCATTCCTCCTGGCTATGGA

83+190_10 -------------GTGATCTGGTTTCTGACGTATATCCCCGCATTCCTCCTGGCTATGGA

83+190_11 --------GTGGGGTGATCTGGTTTCTGACGTATATCCCCGCATTCCTCCTGGCTATGGA

83+190_13 -------------GTGATCTGGTTTCTGACGTATATCCCCGCATTCCTCCTGGCTATGGA

83+190_14 -------------GTGATCTGGTTTCTGACGTATATCCCCGCATTCCTCCTGGCTATGGA

83+190_16 -------------GTGATCTGGTTTCTGACGTATATCCCCGCATTCCTCCTGGCTATGGA

83+190_17 -------------GTGATCTGGTTTCTGACGTATATCCCCGCATTCCTCCTGGCTATGGA

*********** ** * * ** ***

83+190_4 CGNGCACATGTNCCTCTCTACACGAGCTCTCCCGCGCGTAATGCTCAACTCCGCCATGTC

83+190_19 CGTGAACATGTCTACCTCTCTACAAGCGGTCACCTGCCTAATGCTCAACTCCGCCATGTC

83+190_18 CGTGAACATGTCTACCTCTCTACAAGCGGTCACCTGCCTAATGCTCAACTCCGCCATGTC

83+190_15 CGTGAACATGTCTACCTCTCTACAAGCGGTCACCTGCCTAATGCTCAACTCCGCCATGTC

83+190_12 CGTGAACATGTCTACCTCTCTACAAGCGGTCACCTGCCTAATGCTCAACTCCGCCATGTC

83+190_6 CGTGAACATGTCTACCTCTCTACAAGCGGTCACCTGCCTAATGCTCAACTCCGCCATGTC

83+190_5 CGTGAACATGTCTACCTCTCTACAAGCGGTCACCTGCCTAATGCTCAACTCCGCCATGTC

APHIS-S_83+190 CGTGAACATGTCTACCTCTCTACAAGCGGTCACCTGCCTAATGCTCAACTCCGCCATGTC

83+190_2 CGTGAACATGTCTACCTCTCTACAAGCGGTCACCTGCCTAATGCTCAACTCCGCCATGTC

83+190_3 CGTGAACATGTCTACCTCTCTACAAGCGGTCACCTGCCTAATGCTCAACTCCGCCATGTC

83+190_1 CGTGAACATGTCTACCTCTCTACAAGCGGTCACCTGCCTAATGCTCAACTCCGCCATGTC

83+190_7 CGTGAACATGTCTACCTCTCTACAAGCGGTCACCTGCCTAATGCTCAACTCCGCCATGTC

83+190_8 CGTGAACATGTCTACCTCTCTACAAGCGGTCACCTGCCTAATGCTCAACTCCGCCATGTC

83+190_9 CGTGAACATGTCTACCTCTCTACAAGCGGTCACCTGCCTAATGCTCAACTCCGCCATGTC

83+190_10 CGTGAACATGTCTACCTCTCTACAAGCGGTCACCTGCCTAATGCTCAACTCCGCCATGTC

83+190_11 CGTGAACATGTCTACCTCTCTACAAGCGGTCACCTGCCTAATGCTCAACTCCGCCATGTC

83+190_13 CGTGAACATGTCTACCTCTCTACAAGCGGTCACCTGCCTAATGCTCAACTCCGCCATGTC

83+190_14 CGTGAACATGTCTACCTCTCTACAAGCGGTCACCTGCCTAATGCTCAACTCCGCCATGTC

83+190_16 CGTGAACATGTCTACCTCTCTACAAGCGGTCACCTGCCTAATGCTCAACTCCGCCATGTC

83+190_17 CGTGAACATGTCTACCTCTCTACAAGCGGTCACCTGCCTAATGCTCAACTCCGCCATGTC

** * ****** **** ** *** ** * ** **********************

83+190_4 TTACGGCTTCCAGCTGTTACTGGCCCGGGAAAGTACCGGAGGAATGCAGGGGGGAGTTTT

83+190_19 TTACGGCTTCCAGCTGTTACTGGCCCGGGAAAGTACCGGAGGAATGCAGTGGGGTGATTT

83+190_18 TTACGGCTTCCAGCTGTTACTGGCCCGGGAAAGTACCGGAGGAATGCAGTGGGGTGATTT

83+190_15 TTACGGCTTCCAGCTGTTACTGGCCCGGGAAAGTACCGGAGGAATGCAGTGGGGTGATTT

83+190_12 TTACGGCTTCCAGCTGTTACTGGCCCGGGAAAGTACCGGAGGAATGCAGTGGGGTGATTT

83+190_6 TTACGGCTTCCAGCTGTTACTGGCCCGGGAAAGTACCGGAGGAATGCAGTGGGGTGATTT

83+190_5 TTACGGCTTCCAGCTGTTACTGGCCCGGGAAAGTACCGGAGGAATGCAGTGGGGTGATTT

APHIS-S_83+190 TTACGGCTTCCAGCTGTTACTGGCCCGGGAAAGTACCGGAGGAATGCAGTGGGGTGATTT

83+190_2 TTACGGCTTCCAGCTGTTACTGGCCCGGGAAAGTACCGGAGGAATGCAGTGGGGTGATTT

83+190_3 TTACGGCTTCCAGCTGTTACTGGCCCGGGAAAGTACCGGAGGAATGCAGTGGGGTGATTT

83+190_1 TTACGGCTTCCAGCTGTTACTGGCCCGGGAAAGTACCGGAGGAATGCAGTGGGGTGATTT

83+190_7 TTACGGCTTCCAGCTGTTACTGGCCCGGGAAAGTACCGGAGGAATGCAGTGGGGTGATTT

83+190_8 TTACGGCTTCCAGCTGTTACTGGCCCGGGAAAGTACCGGAGGAATGCAGTGGGGTGATTT

83+190_9 TTACGGCTTCCAGCTGTTACTGGCCCGGGAAAGTACCGGAGGAATGCAGTGGGGTGATTT

83+190_10 TTACGGCTTCCAGCTGTTACTGGCCCGGGAAAGTACCGGAGGAATGCAGTGGGGTGATTT

83+190_11 TTACGGCTTCCAGCTGTTACTGGCCCGGGAAAGTACCGGAGGAATGCAGTGGGGTGATTT

83+190_13 TTACGGCTTCCAGCTGTTACTGGCCCGGGAAAGTACCGGAGGAATGCAGTGGGGTGATTT

83+190_14 TTACGGCTTCCAGCTGTTACTGGCCCGGGAAAGTACCGGAGGAATGCAGTGGGGTGATTT

83+190_16 TTACGGCTTCCAGCTGTTACTGGCCCGGGAAAGTACCGGAGGAATGCAGTGGGGTGATTT

83+190_17 TTACGGCTTCCAGCTGTTACTGGCCCGGGAAAGTACCGGAGGAATGCAGTGGGGTGATTT

************************************************* **** * ***

83+190_4 TAACACCTCCACCACCACAGACTCCTCGAT------------------------------

83+190_19 TATGACGTCACCAGCAACGGACTCGTCACGATTCGTATTCGGTCACGTC-----------

83+190_18 TATGACGTCACCAGCAACGGACTCGTCACGATTCGTATTCGGTCACGTCNGTTATAATAT

83+190_15 TATGACGTCACCAGCAACGGACTCGTCACGATTCGTATTCGGT-----------------

83+190_12 TATGACGTCACCAGCAACGGACTCGTCACGATTCGTATTCGGT-----------------

83+190_6 TATGACGTCACCAGCAACGGACTCGTCACGATTCGTATTCGGTCACGTCGCTATAAT---

83+190_5 TATGACGTCACCAGCAACGGACTCGTCACGATTCGTATTCGGTCACGTAATGCAGT----

APHIS-S_83+190 TATGACGTCACCAGCAACGGACTCGTCACGATTC**GTATTCGGTCACGTCGTTATAAT**---

83+190_2 TATGACGTCACCAGCAACGGACTCGTCACGATTCGTATTCGGTCACGTCGTTATAAT---

83+190_3 TATGACGTCACCAGCAACGGACTCGTCACGATTCGTATTCGGTCACGTCGTTATAAT---

83+190_1 TATGACGTCACCAGCAACGGACTCGTCACGATTCGTATTCGGT-----------------

83+190_7 TATGACGTCACCAGCAACGGACTCGTCACGATTCGTATTCGGTCAC--------------

83+190_8 TATGACGTCACCAGCAACGGACTCGTCACGATTCGTATTCGGTCAC--------------

83+190_9 TATGACGTCACCAGCAACGGACTCGTCACGATTCGTATTCGGTCAC--------------

83+190_10 TATGACGTCACCAGCAACGGACTCGTCACGATTCGTATTCGGTCAC--------------

83+190_11 TATGACGTCACCAGCAACGGACTCGTCACGATTCGTATTCGGTCACG-------------

83+190_13 TATGACGTCACCAGCAACGGACTCGTCACGATTCGTATTCGGTCAC--------------

83+190_14 TATGACGTCACCAGCAACGGACTCGTCACGATTCGTATTCGGT-----------------

83+190_16 TATGACGTCACCAGCAACGGACTCGTCACGATTCGTATTCGGT-----------------

83+190_17 TATGACGTCACCAGCAACGGACTCGTCACGATTCGTATTCGGT-----------------

** ** ** * * ** ***** **

**D**

53+139_20 ---------------------------------------------------------AAA

53+139_13 ------------------------------------------------------------

53+139_11 ------------------------------------------------------------

53+139_4 -------------AGCGGTAAAGAGTTGGATGAAGAA-ATTGAGACGCTTATTGA-AAAA

53+139_7 -------------AGCGGTAAAGAGTTGGATGAAGAA-ATTGAGACGCTTATTGA-AAAA

53+139_6 -------GGCTTCAGCGGTAAAGAGTTGGATGAAGAAAATTGAGACGNNTATTGAAAAAA

53+139_5 -------------------------------------------------GCTTATTGAAA

53+139_18 ----AGGNCNTCAGCGGTNAAAGAGTTGGATGAAGAA-ATTGAGACGCTTAATTGAAAAA

53+139_17 ---------TTCAGCGGGTAAAGAGTTGGATGAAGAA-ATTGNNACGCTTATTGAAAAAA

53+139_16 -----------TCAGCGGTAAAGAGTTGGATGAAGAA-ATTGAGACGCTTATTGA-AAAA

53+139_12 ------------------------------------------------------------

53+139_8 ----------------GTAAAAGAGTNGGATGAAGAA-NTTGAGACGCTTATNGAAAAAN

53+139_2 ----------TTCAGCGGTAAAGAGTTGGATGAAGAA-ATTGAGACGCTTATTGA-A-NN

53+139_1 CTGAAAGGGCTTCAGCGGTAAAGAGTTGGATGAAGAA-ATTGAGACGNTTATTGA-AAAA

APHIS-S_53+139 -**CTGAAAGGCTTCAGCGGTAA**AGAGTTGGATGAAGAA-ATTGAGACGCTTATTGA-AAAA

53+139_3 ----------TTCAGCGGTAAAGAGTTGGATGAAGAA-ATTGAGACGCTTATTGA-AAAA

53+139_9 ----------TTCAGCGGTAAAGAGTTGGATGAAGAA-ATTGAGACGCTTATTGA-AAAA

53+139_10 --------GCTTCAGCGGTAAAGAGTTGGATGAAGAA-ATTGAGACGCTTATTGA-AAAA

53+139_19 ------------------------------------------------------------

53+139_20 GTTGGGATTGCAAGAAAAGAGGGATTACCAATCAGCGGGATTATCAGGGGGACAGAAGCG

53+139_13 ---GGAATTGCAAGAAAAGAGGGATTACCAATCAGCGGGGTTATCAGGGGGACAGAAGCG

53+139_11 -TTGGANTTGCAAGAAAAGAGGGATTACCAATCAGCGGGGTTATCAGGGGGACAGAAGCG

53+139_4 TTGGAATT-GCAAGAAAAGAGGGATTACCAATCAGCGGGGTTATCAGGGGGACAGAAGCG

53+139_7 TTGGAATT-GCAAGAAAAGAGGGATTACCAATCAGCGGGGTTATCAGGGGGACAGAAGCG

53+139_6 TTGGAATTGCAAGAAAAAGAGGGATTACCAATCAGCGGGGTTATCAGGGGGACAGAAGCG

53+139_5 TTGGAATT-GCAAGAAAAGAGGGATTACCAATCAGCGGGGTTATCAGGGGGACAGAAGCG

53+139_18 TTGGAATT-GCAAGAAAAGAGGGATTACCAATCAGCGGGGTTATCAGGGGGACAGAAGCG

53+139_17 TTGGAATT-GCAAGAAAAGAGGGATTACCAATCAGCGGGGTTATCAGGGGGACAGAAGCG

53+139_16 TTGGAATT-GCAAGAAAAGAGGGATTACCAATCAGCGGGGTTATCAGGGGGACAGAAGCG

53+139_12 TTGGAATT-GCAAGAAAAGAGGGATTACCAATCAGCGGGGTTATCAGGGGGACAGAAGCG

53+139_8 TTGGAATT-GCAAGAAAAGAGGGATTACCAATCAGCGGGGTTATCAGGGGGACAGAAGCG

53+139_2 TTGGAATT-GCAAGAAAAGAGGGATTACCAATCAGCGGGGTTATCAGGGGGACAGAAGCG

53+139_1 TTGGAATT-GCAAGAAAAGAGGGATTACCAATCAGCGGGGTTATCAGGGGGACAGAAGCG

APHIS-S_53+139 TTGGAATT-GCAAGAAAAGAGGGATTACCAATCAGCGGGGTTATCAGGGGGACAGAAGCG

53+139_3 TTGGAATT-GCAAGAAAAGAGGGATTACCAATCAGCGGGGTTATCAGGGGGACAGAAGCG

53+139_9 TTGGAATT-GCAAGAAAAGAGGGATTACCAATCAGCGGGGTTATCAGGGGGACAGAAGCG

53+139_10 TTGGAATT-GCAAGAAAAGAGGGATTACCAATCAGCGGGGTTATCAGGGGGACAGAAGCG

53+139_19 --GGAATT-GCAAGAAAAGAGGGATTACCAATCAGCGGGGTTATCAGGGGGACAGAAGCG

* * * ************************* ********************

53+139_20 AGGA--------------CG-CCCATGCGGNGCCGCTAAAGTGGATCTACTGNACGAGCC

53+139_13 ACGATTAGGAGTGNNNNTCTGGCTATGCTGGACGACTACAGTGCTG--------------

53+139_11 ACGATTAGGAGTGGGCGTCG-------CGGGGCGGCTAAAGTGGNTNTANTGGACGAGGN

53+139_4 ACGATTAGGAGTGGGCGTCG-------CGGGGCGGCTAAAGTGGTTCTACTGGACGAGCC

53+139_7 ACGATTATGAGTGGGCGTCG-------CGGGGCGGCTAAAGTGGTTCTACTGGACGAGCC

53+139_6 ACGATTAGGAGTGGGCGTCGCGCTATGCGGGGCGGCTAAAGTGGTTCTACTGGACGAGCC

53+139_5 ACGATTAGGAGTGGGCGTCGCGCTATGCNNAGCGTGTAACGANNGGACANNNCACCATCT

53+139_18 ACGATTAGGAGTGGGCGTCGCGCTATGCGGGGCGG-------------------------

53+139_17 ACGATTAGGAGTGGGCGTCGCGCTATGCGGGGCGGCTAAAGTGGTTCTACTGGACGAGCC

53+139_16 ACGATTAGGAGTGGGCGTCGCGCTATGCGGGGCGGCTAAAGTGGTTNTNNTGGACGAGCC

53+139_12 ACGATTAGGAGTGGGCGTCGCGCTATGCGGGGCGGCTAAAAGTGGTTCTACTGGAC----

53+139_8 ACGATTAGGAGTGGGCGTCGCGCTATGCGGGGCGGCTAAAGTGGTTCTACTGGACGAGCC

53+139_2 ACGATTAGGAGTGGGCGTCGCGCTATGCGGGGCGGCTAAAGTGGTTCTANNGGACGAGCC

53+139_1 ACGATTAGGAGTGGGCGTCGCGCTATGCGGGGCGGCTAAAGTGGTTCTACTGGACGAGCC

APHIS-S_53+139 ACGATTAGGAGTGGGCGTCGCGCTATGCGGGGCGGCTAAAGTGGTTCTACTGGACGAGCC

53+139_3 ACGATTAGGAGTGGGCGTCGCGCTATGCGGGGCGGCTAAAGTGGTTCTACTGGACGAGCC

53+139_9 ACGATTAGGAGTGGGCGTCGCGCTATGCGGGGCGGCTAAAGTGGTTCTACTGGACGAGCC

53+139_10 ACGATTAGGAGTGGGCGTCGCGCTATGCGGGGCGGCTAAAGTGGTTCTACTGGACGAGCC

53+139_19 ACGATTAGGAGTGGGCGTCGCGCTATGCGGNGCGG-------------------------

* ** * *

53+139_20 CACTNCTGGCATGGACTCGG----------------------------

53+139_13 ------------------------------------------------

53+139_11 NNCTTCTGGCATGGANCCGGCCTCACGTCGTGCCCTANGGG-------

53+139_4 CACTTCTGGCATGGACCCGGCCTCACGTCGTG----------------

53+139_7 CACTTCTGGCATGGACCCGGCCTCACGTCGTGCCCT------------

53+139_6 CACTTCTGGCATGGACCCGGCCTCACGTCGTGC---------------

53+139_5 GACTTGTAGCATGGATCAGGTCTCGCGTCTGGCCATTGGTGACTTGTT

53+139_18 ------------------------------------------------

53+139_17 CACTTCTGGCATGGANCCGGCCTCACGTCGTGCCCT------------

53+139_16 CACTTCTGGCATGGACCCGGCCTCACGTCGTGCCCT------------

53+139_12 ------------------------------------------------

53+139_8 CACTTCTGGCATGGACCCGGCCTCACGTCGTG----------------

53+139_2 CACTTCTGGCATGGACCCGGCCTCACGTCGTGC---------------

53+139_1 CACTTCTGGCATGGACCCGGCCTCACGTCGTG----------------

APHIS-S_53+139 CACTTCTGGCATGGACCCGGCCTCACGTC**GTGCCCTATGGGACTTGTT**

53+139_3 CACTTCTGGCATGGACCCGGCCTCACGTCGTG----------------

53+139_9 CACTTCTGGCATGGACCCGGCCTCACGTCGTG----------------

53+139_10 CACTTCTGGCATGGACCCGGCCTCACGTCGTGCC--------------

53+139_19 ------------------------------------------------

**Supplementary Figure S4. Alignment of *PgABCA2* cDNA sequences from CRISPR-R2 G_4_ survivors on 3 μg Cry2Ab per mL lacking gDNA mutations corresponding to sgRNA target sites.** A. Four *PgABCA2* cDNA clones from CRISPR-R2 Larva 3 are aligned with the full-length *PgABCA2* (MG637361.1) from susceptible strain APHIS-S (the numbers to the right of the decimal point refer to the clone). B. Six cDNA clones from Larva 5 are aligned with wild-type *PgABCA2* cDNA. Two distinct PCR products resolved by agarose electrophoresis from Larva 5 and were cloned (indicated as 5A and 5B). C. Seven cDNA clones from Larva 9 are aligned with *PgABCA2* cDNA from APHIS-S. Two distinct PCR products resolved by agarose electrophoresis from Larva 9 and were cloned (indicated as 9A and 9B). D. Eight cDNA clones from Larva 10 are aligned with APHIS-S *PgABCA2* cDNA. E. Three cDNA clones from Larva 18 are aligned with wild-type *PgABCA2* cDNA. sgRNA target sites 1-5 are highlighted in magenta, cyan, teal, yellow, and red, respectively. Predicted exon/intron splice sites are shown in gray. The order of sequences is based on their similarity determined by Clustal Omega (<https://www.ebi.ac.uk/Tools/msa/clustalo/>). Premature stop codons are highlighted in black with white text. Stars show nucleotide bases conserved in all of the sequences.

**A**

3.1 ATGCGGGCGCGTGGAGAGCGGAAGGAGGCGGGCTCATGGGTGAAGTTTAGGCTGTTGATG

APHIS-S ATGCGGGCGCGTGGAGAGCGGAAGGAGGCGGGCTCATGGGTGAAGTTTAGGCTGTTGATG

3.3 ATGCGGGCGCGTGGAGAGCGGAAGGAGGCGGGCTCATGGGTGAAGTTTAGGCTGTTGATG

3.15 ATGCGGGCGCGTGGAGAGCGGAAGGAGGCGGGCTCATGGGTGAAGTTTAGGCTGTTGATG

3.2 ATGCGGGCGCGTGGAGAGCGGAAGGAGGCGGGCTCATGGGTGAAGTTTAGGCTGTTGATG

************************************************************

3.1 TGGAAGAACTTCGTGCAGCAGTTGAGGCACCCAGTGCAGACGGCGGCTGAGCTGCTGCTA

APHIS-S TGGAAGAACTTCGTGCAGCAGTTGAGGCACCCAGTGCAGACGGCGGCTGAGCTGCTGCTA

3.3 TGGAAGAACTTCGTGCAGCAGTTGAGGCACCCAGTGCAGACGGCGGCTGAGCTGCTGCTA

3.15 TGGAAGAACTTCGTGCAGCAGTTGAGGCACCCAGTGCAGACGGCGGCTGAGCTGCTGCTA

3.2 TGGAAGAACTTCGTGCAGCAGTTGAGGCACCCAGTGCAGACGGCGGCTGAGCTGCTGCTA

************************************************************

3.1 CCAGTCCTAACCATGAGCCTGGTCCTGGTGCTACGGTCACAGATCGACCCCGAAGTCTTG

APHIS-S CCAGTCCTAACCATGAGCCTGGTCCTGGTGCTACGGTCACAGATCGACCCCGAAGTCTTG

3.3 CCAGTCCTAACCATGAGCCTGGTCCTGGTGCTACGGTCACAGATCGACCCCGAAGTCTTG

3.15 CCAGTCCTAACCATGAGCCTGGTCCTGGTGCTACGGTCACAGATCGACCCCGAAGTCTTG

3.2 CCAGTCCTAACCATGAGCCTGGTCCTGGTGCTACGGTCACAGATCGACCCCGAAGTCTTG

************************************************************

3.1 GAAACCAGAACCTACCTGCCAATACCAGCCCACACTTTAAACTATTCCGTGACTGTTTTG

APHIS-S GAAACCAGAACCTACCCGCCAATACCAGCCCACACTTTAAACTATTCCGTGACTGTTTTG

3.3 GAAACCAGAACCTACCCGCCAATACCAGCCCACACTTTAAACTATTCCGTGACTGTTTTG

3.15 GAAACCAGAACCTACCCGCCAATACCAGCCCACACTTTAAACTATTCCGTGACTGTTTT-

3.2 GAAACCAGAACCTACCCGCCAATACCAGCCCACACTTTAAACTATTCCGTGACTGTTTT-

**************** ******************************************

3.1 GGCGGAATGAATTTAACAAGAATGTCCATGGCATTCTCACTCGAGAATGCCGTAGCGGTG

APHIS-S GGCGGAATGAATTTAACAAGAATGTCCATGGCATTCTCACCCGAGAATGCCGTATTGAGG

3.3 GGCGGAATGAATTTAACAAGAATGTCCATGGCATTCTCACTCGAGAATGCCGTAGCGGTG

3.15 ------------------------------------------------------------

3.2 ------------------------------------------------------------

3.1 ---------------------------------------------------TAACTGAAG

APHIS-S GACGTCGTATCCAGTGCTACAACAAAGTTACTGCTTAAAAACATGAGAGACCAAGTACTG

3.3 ---------------------------------------------------TAACTGAAG

3.15 ------------------------------------------------------------

3.2 ------------------------------------------------------------

3.1 CTGATCA-TGATGAATGATGGGAAGAGGAACTGCAGGGCTTCCTTCGC------CAAGTC

APHIS-S CCCATCATTGAGGCATTGCCAATAGAAATACCGCCGGGACTGGTAAACTCGTCACAGGTG

3.3 CTGATCA-TGATGAATGATGGGAAGAGGAACTGCAGGGCTTCCTTCGC------CAAGTC

3.15 ------------------------------------------------------------

3.2 ------------------------------------------------------------

3.1 CATGATGTA-----------------------------TGCTGGAT--------------

APHIS-S TACGAAATAGTTAAATTATTTGTCGACGAGAACGTTGTTACCGGATACAATAGCAGTGCG

3.3 CATGATGTA-----------------------------TGCTGGAT--------------

3.15 ------------------------------------------------------------

3.2 ------------------------------------------------------------

3.1 ------------------------------------------------------------

APHIS-S GCAATGAGAGGAATATACGCAGAGGAAGAAGCCACGAGAAGGGTGATAGCTGGCATAGAA

3.3 ------------------------------------------------------------

3.15 ------------------------------------------------------------

3.2 ------------------------------------------------------------

3.1 -------------------AAATAACGGAGCTACCACTAGACTTGTCGTATGCGCTTCGT

APHIS-S TTCGATGACTCATTGCGTGAAATAACGGAGCTACCACTAGACTTGTCGTATGCGCTTCGT

3.3 --------------------------GTGGGTACCTCT----------------------

3.15 ------------------------------------------------------------

3.2 ------------------------------------------------------------

3.1 TTTCCGGAGAGACCTCGCTTGAATTCCTTCTTCATGACAGGCGGTCGGACTTGGCGCACA

APHIS-S TTTCCGGAGAGACCTCGCTTGAATTCCTTCTTCATGACAGGCGGTCGGACTTGGCGCACA

3.3 ------------------------------------------------------------

3.15 ------------------------------------------------------------

3.2 ------------------------------------------------------------

3.1 GATAACGTGTTTCCTA--------------------------------------------

APHIS-S GATAACGTGTTTCCTATGTTCGAAGTTCCCGGACCTCGCTTTCCGTATTCATGGGAAGGT

3.3 ------------------------------------------------------------

3.15 ------------------------------------------------------------

3.2 ------------------------------------------------------------

3.1 ------------------------------------------------------------

APHIS-S GGAAATGATCCAGGATACGTAAACGAGATGTTCATAGCCTTGCAGCACATGATATCTTCA

3.3 ------------------------------------------------------------

3.15 ------------------------------------------------------------

3.2 ------------------------------------------------------------

3.1 ------------------------------------------------------------

APHIS-S GAACTGGTATCTAAAGTGGCGGGAGTGAACCTAGACTTCGATGTGCACATACAGAGGTAC

3.3 ------------------------------------------------------------

3.15 ------------------------------------------------------------

3.2 ------------------------------------------------------------

3.1 ------------------------------------------------------------

APHIS-S CCACATCCAGCATACATCATGGACTTGGCGAAGGAAGCCCTGCAGTTCCTCTTCCCATCA

3.3 ------------------------------------------------------------

3.15 ------------------------------------------------------------

3.2 ------------------------------------------------------------

3.1 ------------------------------------------------------------

APHIS-S TTCATCATGATCAGCTTCAGTTACACCGCTATCAATATTATACGATCCGTGACCGTGGAA

3.3 ------------------------------------------------------------

3.15 ------------------------------------------------------------

3.2 ------------------------------------------------------------

3.1 ------------------------------------------------------------

APHIS-S AAAGAAATGCAATTGAAGGAAACGATGAAGATCATGGGACTCCCAACGTGGCTGCATTGG

3.3 ------------------------------------------------------------

3.15 ------------------------------------------------------------

3.2 ------------------------------------------------------------

3.1 ------------------------------------------------------------

APHIS-S ATGGCATGGTTTTTTAAACAATTTATTTATTTGCTGATTGCTTCGGTTTTGATACTTGTT

3.3 ------------------------------------------------------------

3.15 ------------------------------------------------------------

3.2 ------------------------------------------------------------

3.1 ------------------------------------------------------------

APHIS-S ATATTAAAGGTAAATTGGTTTACTACAGAAGAAGGCTTTAGCGACTATGCCGTATTCACT

3.3 ------------------------------------------------------------

3.15 ------------------------------------------------------------

3.2 ------------------------------------------------------------

3.1 ------------------------------------------------------------

APHIS-S AATACACCTTGGACCGTCCTCTTCTTCTTCCTAACACTGTATCTTACGTGTACCATATTT

3.3 ------------------------------------------------------------

3.15 ------------------------------------------------------------

3.2 ------------------------------------------------------------

3.1 ------------------------------------------------------------

APHIS-S TTCTGTTTCATGATAAGTGGTTTCTTTTCAAAAGCCAGTACAGCGGCGTTGTTTGGTGGG

3.3 ------------------------------------------------------------

3.15 ------------------------------------------------------------

3.2 ------------------------------------------------------------

3.1 ------------------------------------------------------------

APHIS-S GTGATCTGGTTTCTGACGTATATCCCCGCATTCCTCCTGGCTATGGACGTGAACATGTCT

3.3 ------------------------------------------------------------

3.15 ------------------------------------------------------------

3.2 ------------------------------------------------------------

3.1 ------------------------------------------------------------

APHIS-S ACCTCTCTACAAGCGGTCACCTGCCTAATGCTCAACTCCGCCATGTCTTACGGCTTCCAG

3.3 ------------------------------------------------------------

3.15 ------------------------------------------------------------

3.2 ------------------------------------------------------------

3.1 ------------------------------------------------------------

APHIS-S CTGTTACTGGCCCGGGAAAGTACCGGAGGAATGCAGTGGGGTGATTTTATGACGTCACCA

3.3 ------------------------------------------------------------

3.15 ------------------------------------------------------------

3.2 ------------------------------------------------------------

3.1 ------------------------------------------------------------

APHIS-S GCAACGGACTCGTCACGATTCGTATTCGGTCACGTCGTTATAATGATGGCTTTGAACTGT

3.3 ------------------------------------------------------------

3.15 ------------------------------------------------------------

3.2 ------------------------------------------------------------

3.1 ------------------------------------------------------------

APHIS-S GTGCTCTACATGTTGATTGCCCTATATCTAGAGCAAGTACTACCCGGGCCGTATGGCACA

3.3 ------------------------------------------------------------

3.15 ------------------------------------------------------------

3.2 ------------------------------------------------------------

3.1 ------------------------------------------------------------

APHIS-S CCGAAGCCCTGGTATTTCTTCGTCCAAAGACAGTTCTGGTGTAGCAGCAAAACTACTCAT

3.3 ------------------------------------------------------------

3.15 ------------------------------------------------------------

3.2 ------------------------------------------------------------

3.1 ------------------------------------------------------------

APHIS-S GATATCGGTACAGACAACAGCGACACATCAAGTTTAACAAAAGAAAGCGACCCTACAGAC

3.3 ------------------------------------------------------------

3.15 ------------------------------------------------------------

3.2 ------------------------------------------------------------

3.1 ------------------------------------------------------------

APHIS-S CTTCCGATTGGAGTTAAAATACAAAACCTTAAAAAGGTTTACGGGAGCAACGTTGCGGTA

3.3 ------------------------------------------------------------

3.15 ------------------------------------------------------------

3.2 ------------------------------------------------------------

3.1 ------------------------------------------------------------

APHIS-S AACAATTTATCCCTCAACATTTACGACGACCAAATCACGGTTCTACTTGGACACAACGGA

3.3 ------------------------------------------------------------

3.15 ------------------------------------------------------------

3.2 ------------------------------------------------------------

3.1 ------------------------------------------------------------

APHIS-S GCGGGAAAATCCACAACCATTTCAATGCTCACAGGTAACGTGGACATAACCAGCGGGTCG

3.3 ------------------------------------------------------------

3.15 ------------------------------------------------------------

3.2 ------------------------------------------------------------

3.1 ------------------------------------------------------------

APHIS-S GTGACGGTGGCTGGCTACGACATAGAAAAACAAACAAGTTCAGCACGCTCACACATTGGA

3.3 ------------------------------------------------------------

3.15 ------------------------------------------------------------

3.2 ------------------------------------------------------------

3.1 ------------------------------------------------------------

APHIS-S CTCTGCCCTCAACATAACGTACTCTTCAACGAACTCACAGTCAAAGAACATTTACAGTTC

3.3 ------------------------------------------------------------

3.15 ------------------------------------------------------------

3.2 ------------------------------------------------------------

3.1 ------------------------------------------------------------

APHIS-S TTCTCTCGTCTGAAAGGCTTCAGCGGTAAAGAGTTGGATGAAGAAATTGAGACGCTTATT

3.3 ------------------------------------------------------------

3.15 ------------------------------------------------------------

3.2 ------------------------------------------------------------

3.1 ------------------------------------------------------------

APHIS-S GAAAAATTGGAATTGCAAGAAAAGAGGGATTACCAATCAGCGGGGTTATCAGGGGGACAG

3.3 ------------------------------------------------------------

3.15 ------------------------------------------------------------

3.2 ------------------------------------------------------------

3.1 ------------------------------------------------------------

APHIS-S AAGCGACGATTAGGAGTGGGCGTCGCGCTATGCGGGGCGGCTAAAGTGGTTCTACTGGAC

3.3 ------------------------------------------------------------

3.15 ------------------------------------------------------------

3.2 ------------------------------------------------------------

3.1 ------------------------------------------------------------

APHIS-S GAGCCCACTTCTGGCATGGACCCGGCCTCACGTCGTGCCCTATGGGACTTGTTGCAGAGA

3.3 ------------------------------------------------------------

3.15 ------------------------------------------------------------

3.2 ------------------------------------------------------------

3.1 ------------------------------------------------------------

APHIS-S GAGAAGAAAGGTCGATCGATGATCCTGACGACACACTTCATGGACGAAGCGGACATATTA

3.3 ------------------------------------------------------------

3.15 ------------------------------------------------------------

3.2 ------------------------------------------------------------

3.1 ------------------------------------------------------------

APHIS-S GGGGATAGAGTTGCCATTATGGCGGACGGTCGTCTCCAGTGCGTGGGCTCACCTTACTTC

3.3 ------------------------------------------------------------

3.15 ------------------------------------------------------------

3.2 ------------------------------------------------------------

3.1 ------------------------------------------------------------

APHIS-S CTCAAGAGACACTATGGAGTCGGCTACACGCTAGTTGTGGTCAAGAAGGAAGATTTCCGA

3.3 ------------------------------------------------------------

3.15 ------------------------------------------------------------

3.2 ------------------------------------------------------------

3.1 ------------------------------------------------------------

APHIS-S CTGGACACCTGCACAGAGCTGATCAATAGATACATCCCTGGAACTGTTGTGAAGGAAGAC

3.3 ------------------------------------------------------------

3.15 ------------------------------------------------------------

3.2 ------------------------------------------------------------

3.1 ------------------------------------------------------------

APHIS-S CGAGGCACTGAAGTGACATATAGCATGACTAATGAGTATTCGCACGTGTTTGAATCTATG

3.3 ------------------------------------------------------------

3.15 ------------------------------------------------------------

3.2 ------------------------------------------------------------

3.1 ------------------------------------------------------------

APHIS-S CTGCGCGATTTGGAGGCAAAGGCCGATGAGATAAACTTTAAAAACTACGGCCTACTGGCT

3.3 ------------------------------------------------------------

3.15 ------------------------------------------------------------

3.2 ------------------------------------------------------------

3.1 ------------------------------------------------------------

APHIS-S ACTACATTAGAAGATGTGTTCATGTCCGTGGGCACAGATGTGGTCGCAACTTCAGATGTG

3.3 ------------------------------------------------------------

3.15 ------------------------------------------------------------

3.2 ------------------------------------------------------------

3.1 ------------------------------------------------------------

APHIS-S GACGACAATACAACCGTTTCATCTAGTGCTGATACTCTAGCATTTGAATATGATTCTTTA

3.3 ------------------------------------------------------------

3.15 ------------------------------------------------------------

3.2 ------------------------------------------------------------

3.1 ------------------------------------------------------------

APHIS-S GAAAAATTGGACGGGACTGGCTATGGGGATGAAAAAGGGATCCGATTAATTTGCCAACAC

3.3 ------------------------------------------------------------

3.15 ------------------------------------------------------------

3.2 ------------------------------------------------------------

3.1 ------------------------------------------------------------

APHIS-S GTGGTAGCAATATGGATGAAACTGTTTCTGGTGCTGACAAGGTCTTGGCTTATCCTGTTG

3.3 ------------------------------------------------------------

3.15 ------------------------------------------------------------

3.2 ------------------------------------------------------------

3.1 ------------------------------------------------------------

APHIS-S CTCCAAGTATTGGTGTCCTTGGTACAAATCATTGCCACACTCGGAGTCATGCAGTATGTC

3.3 ------------------------------------------------------------

3.15 ------------------------------------------------------------

3.2 ------------------------------------------------------------

3.1 ------------------------------------------------------------

APHIS-S ATCTCTATGACCGAGCATATACAAAGAAGAGAACTTTCATTGGCTGAAGGTTTCGCAGGC

3.3 ------------------------------------------------------------

3.15 ------------------------------------------------------------

3.2 ------------------------------------------------------------

3.1 ------------------------------------------------------------

APHIS-S ACAGAAACATTAGTTAGTTTCAAAGGGTTGTCCCCTACATCGACAGGTTCGCTAGCGAAG

3.3 ------------------------------------------------------------

3.15 ------------------------------------------------------------

3.2 ------------------------------------------------------------

3.1 ------------------------------------------------------------

APHIS-S GCTGCCTACGAGTCGATATTTGTAACCGCCAATAATCCCACAATGGAAATCACTGTTGTT

3.3 ------------------------------------------------------------

3.15 ------------------------------------------------------------

3.2 ------------------------------------------------------------

3.1 ----------------------------TGGGGCAGACAGATGACGTATCAGCGATGGCG

APHIS-S GATAATACACCTATAGATGAATATTATTTGGAAAGAACAGATGACGTATCAGCGATGGCG

3.3 ----------------------------------------ATGACGTATCAGCGATGGCG

3.15 ------------------------------------ACAGATGACGTATCAGCGATGGCG

3.2 ----------------------------------------ATGACGTATCAGCGATGGCG

********************

3.1 GTGCTCCGGCACAGTCTGTTGATCGGCGCGACGTTCGACGACCACTCCGCGACCGCGTGG

APHIS-S GTGCTCCGGCACAGTCTGTTGATCGGCGCGACGTTCGACGACCACTCCGCGACCGCGTGG

3.3 GTGCTCCGGCACAGTCTGTTGATCGGCGCGACGTTCGACGACCACTCCGCGACCGCGTGG

3.15 GTGCTCCGGCACAGTCTGTTGATCGGCGCGACGTTCGACGACCACTCCGCGACCGCGTGG

3.2 GTGCTCCGGCACAGTCTGTTGATCGGCGCGACGTTCGACGACCACTCCGCGACCGCGTGG

************************************************************

3.1 TTCAGCAACTTCGGTTACCACGACGTGGCCATGTCACTGGCTGCTGTGCACGCCGCCTTG

APHIS-S TTCAGCAACTTCGGTTACCACGACGTGGCCATGTCACTGGCTGCTGTGCACGCCGCCTTG

3.3 TTCAGCAACTTCGGTTACCACGACGTGGCCATGTCACTGGCTGCTGTGCACGCCGCCTTG

3.15 TTCAGCAACTTCGGTTACCACGACGTGGCCATGTCACTGGCTGCTGTGCACGCCGCCTTG

3.2 TTCAGCAACTTCGGTTACCACGACGTGGCCATGTCACTGGCTGCTGTGCACGCCGCCTTG

************************************************************

3.1 CTCAGAGCTGTCAATCCTGCAGCCAACTTGACTGTTTACAACCACCCACTTGAGGCCAAT

APHIS-S CTCAGAGCTGTCAATCCTGCAGCCAACTTGACTGTTTACAACCACCCACTTGAGGCCAAT

3.3 CTCAGAGCTGTCAATCCTGCAGCCAACTTGACTGTTTACAACCACCCACTTGAGGCCAAT

3.15 CTCAGAGCTGTCAATCCTGCAGCCAACTTGACTGTTTACAACCACCCACTTGAGGCCAAT

3.2 CTCAGAGCTGTCAATCCTGCAGCCAACTTGACTGTTTACAACCACCCACTTGAGGCCAAT

************************************************************

3.1 TATGTCAACCAGAACGACATGCAAACAATGGTAGCGTTCCTCTCGATGCAACTTGCGTCG

APHIS-S TATGTCAACCAGAACGACATGCAAACAATGGTAGCGTTCCTCTCGATGCAACTTGCGTCG

3.3 TATGTCAACCAGAACGACATGCAAACAATGGTAGCGTTCCTCTCGATGCAACTTGCGTCG

3.15 TATGTCAACCAGAACGACATGCAAACAATGGTAGCGTTCCTCTCGATGCAACTTGCGTCG

3.2 TATGTCAACCAGAACGACATGCAAACAATGGTAGCGTTCCTCTCGATGCAACTTGCGTCG

************************************************************

3.1 GGCATCGGCAGCAGTCTGTCAATTGTCAGTGCTGTGTTCATCATGTTCTATATCAAGGAG

APHIS-S GGCATCGGCAGCAGTCTGTCAATTGTCAGTGCTGTGTTCATCATGTTCTATATCAAGGAG

3.3 GGCATCGGCAGCAGTCTGTCAATTGTCAGTGCTGTGTTCATCATGTTCTATATCAAGGAG

3.15 GGCATCGGCAGCAGTCTGTCAATTGTCAGTGCTGTGTTCATCATGTTCTATATCAAGGAG

3.2 GGCATCGGCAGCAGTCTGTCAATTGTCAGTGCTGTGTTCATCATGTTCTATATCAAGGAG

************************************************************

3.1 TGAGTATCTCGCGCCAAGCTGCTGCAGAAGGCGGCAGGCATCCAGCCGTTAGTGATGTGG

APHIS-S CGAGTATCTCGCGCCAAGCTGCTGCAGAAGGCGGCAGGCATCCAGCCGTTAGTGATGTGG

3.3 CGAGTATCTCGCGCCAAGCTGCTGCAGAAGGCGGCAGGCATCCAGCCGTTAGTGATGTGG

3.15 CGAGTATCTCGCGCCAAGCTGCTGCAGAAGGCGGCAGGCATCCAGCCGTTAGTGATGTGG

3.2 CGAGTATCTCGCGCCAAGCTGCTGCAGAAGGCGGCAGGCATCCAGCCGTTAGTGATGTGG

***********************************************************

3.1 CTCAGCGCCGCCGTGTTCGACTGGATCTGGTTCTGCGTCATCGCCGTCGGCATCGTTATC

APHIS-S CTCAGCGCCGCCGTGTTCGACTGGATCTGGTTCTGCGTCATCGCCGTCGGCATCGTTATC

3.3 CTCAGCGCCGCCGTGTTCGACTGGATCTGGTTCTGCGTCATCGCCGTCGGCATCGTTATC

3.15 CTCAGCGCCGCCGTGTTCGACTGGATCTGGTTCTGCGTCATCGCCGTCGGCATCGTTATC

3.2 CTCAGCGCCGCCGTGTTCGACTGGATCTGGTTCTGCGTCATCGCCGTCGGCATCGTTATC

************************************************************

3.1 GCCTGCGCCGCTTTTAACGTCATTGGGCTCTCTTCTGTCGATGAACTGGGTCGGATGTAC

APHIS-S GCCTGCGCCGCTTTTAACGTCATTGGGCTCTCTTCTGTCGATGAACTGGGTCGGATGTAC

3.3 GCCTGCGCCGCTTTTAACGTCATTGGGCTCTCTTCTGTCGATGAACTGGGTCGGATGTAC

3.15 GCCTGCGCCGCTTTTAACGTCATTGGGCTCTCTTCTGTCGATGAACTGGGTCGGATGTAC

3.2 GCCTGCGCCGCTTTTAACGTCATTGGGCTCTCTTCTGTCGATGAACTGGGTCGGATGTAC

************************************************************

3.1 TTGTGCATCATAGTGTATGGCGCCGCCAGTCTACCGATAGGCTACGTGTTCTCCTATTTC

APHIS-S TTGTGCATCATAGTGTATGGCGCCGCCAGTCTACCGATAGGCTACGTGTTCTCCTATTTC

3.3 TTGTGCATCATAGTGTATGGCGCCGCCAGTCTACCGATAGGCTACGTGTTCTCCTATTTC

3.15 TTGTGCATCATAGTGTATGGCGCCGCCAGTCTACCGATAGGCTACGTGTTCTCCTATTTC

3.2 TTGTGCATCATAGTGTATGGCGCCGCCAGTCTACCGATAGGCTACGTGTTCTCCTATTTC

************************************************************

3.1 TTCAAAGGCCCTGCCGTCGGTTTTGTCACCATGTTCTTTATCAACATTCTCTTTGGTATG

APHIS-S TTCAAAGGCCCTGCCGTCGGTTTTGTCACCATGTTCTTTATCAACATTCTCTTTGGTATG

3.3 TTCAAAGGCCCTGCCGTCGGTTTTGTCACCATGTTCTTTATCAACATTCTCTTTGGTATG

3.15 TTCAAAGGCCCTGCCGTCGGTTTTGTCACCATGTTCTTTATCAACATTCTCTTTGGTATG

3.2 TTCAAAGGCCCTGCCGTCGGTTTTGTCACCATGTTCTTTATCAACATTCTCTTTGGTATG

************************************************************

3.1 ATGGGGGCGCAGATTGTGGAGGCCTTGTTGTCACCGCAGCTTGATACTGAAAATGTCGCT

APHIS-S ATGGGGGCGCAGATTGTGGAGGCCTTGTTGTCACCGCAGCTTGATACTGAAAATGTCGCT

3.3 ATGGGGGCGCAGATTGTGGAGGCCTTGTTGTCACCGCAGCTTGATACTGAAAATGTCGCT

3.15 ATGGGGGCGCAGATTGTGGAGGCCTTGTTGTCACCGCAGCTTGATACTGAAAATGTCGCT

3.2 ATGGGGGCGCAGATTGTGGAGGCCTTGTTGTCACCGCAGCTTGATACTGAAAATGTCGCT

************************************************************

3.1 AATATACTTGACTCCATCTTGCAATTCTTCCCACTCTATAGTCTTGTCACATCTGCCAGA

APHIS-S AATATACTTGACTCCATCTTGCAATTCTTCCCACTCTATAGTCTTGTCACATCTGCCAGA

3.3 AATATACTTGACTCCATCTTGCAATTCTTCCCACTCTATAGTCTTGTCACATCTGCCAGA

3.15 AATATACTTGACTCCATCTTGCAATTCTTCCCACTCTATAGTCTTGTCACATCTGCCAGA

3.2 AATATACTTGACTCCATCTTGCAATTCTTCCCACTCTATAGTCTTGTCACATCTGCCAGA

************************************************************

3.1 CTGTTGAATCAGGTGGGACTGCTGGAGTGGTCGTGCCTGCAGAACTGCGAGTACCTGTCC

APHIS-S CTGTTGAATCAGGTGGGACTGCTGGAGTGGTCGTGCCTGCAGAACTGCGAGTACCTGTCC

3.3 CTGTTGAATCAGGTGGGACTGCTGGAGTGGTCGTGCCTGCAGAACTGCGAGTACCTGTCC

3.15 CTGTTGAATCAGGTGGGACTGCTGGAGTGGTCGTGCCTGCAGAACTGCGAGTACCTGTCC

3.2 CTGTTGAATCAGGTGGGACTGCTGGAGTGGTCGTGCCTGCAGAACTGCGAGTACCTGTCC

************************************************************

3.1 GCAGTGATGCCCAACTTGACCGAATGCTCCATGGACGTTATGTGCCAGACGTTCTCACAA

APHIS-S GCAGTGATGCCCAACTTGACCGAATGCTCCATGGACGTTATGTGCCAGACGTTCTCACAA

3.3 GCAGTGATGCCCAACTTGACCGAATGCTCCATGGACGTTATGTGCCAGACGTTCTCACAA

3.15 GCAGTGATGCCCAACTTGACCGAATGCTCCATGGACGTTATGTGCCAGACGTTCTCACAA

3.2 GCAGTGATGCCCAACTTGACCGAATGCTCCATGGACGTTATGTGCCAGACGTTCTCACAA

************************************************************

3.1 TGTTGCATCCCAGACGATCCTTGGTTCATGTGGGATCACCCTGGAGTACTCCGCTACATA

APHIS-S TGTTGCATCCCAGACGATCCTTGGTTCATGTGGGATCACCCTGGAGTACTCCGCTACATA

3.3 TGTTGCATCCCAGACGATCCTTGGTTCATGTGGGATCACCCTGGAGTACTCCGCTACATA

3.15 TGTTGCATCCCAGACGATCCTTGGTTCATGTGGGATCACCCTGGAGTACTCCGCTACATA

3.2 TGTTGCATCCCAGACGATCCTTGGTTCATGTGGGATCACCCTGGAGTACTCCGCTACATA

************************************************************

3.1 GTATGCATGATCGTCAGTGGAGTTGTCATGTGGTTCGTACTCTTGATCGCCGAGTATCGA

APHIS-S GTATGCATGATCGTCAGTGGAGTTGTCATGTGGTTCGTACTCTTGATCGCCGAGTATCGA

3.3 GTATGCATGATCGTCAGTGGAGTTGTCATGTGGTTCGTACTCTTGATCGCCGAGTATCGA

3.15 GTATGCATGATCGTCAGTGGAGTTGTCATGTGGTTCGTACTCTTGATCGCCGAGTATCGA

3.2 GTATGCATGATCGTCAGTGGAGTTGTCATGTGGTTCGTACTCTTGATCGCCGAGTATCGA

************************************************************

3.1 TTGTTCCAGAAGGTGATCTACCGGGAAAAGAAAGCTCCTCCAGTTGATGAGAGCGCACTG

APHIS-S TTGTTCCAGAAGGTGATCTACCGGGAAAAGAAAGCTCCTCCAGTTGATGAGAGCGCACTG

3.3 TTGTTCCAGAAGGTGATCTACCGGGAAAAGAAAGCTCCTCCAGTTGATGAGAGCGCACTG

3.15 TTGTTCCAGAAGGTGATCTACCGGGAAAAGAAAGCTCCTCCAGTTGATGAGAGCGCACTG

3.2 TTGTTCCAGAAGGTGATCTACCGGGAAAAGAAAGCTCCTCCAGTTGATGAGAGCGCACTG

************************************************************

3.1 GACAATGACGTGGCGGACGAGGCCAGACACGTGGCGCGAGTTGGAGCAGGAGCAATCCTC

APHIS-S GACAATGACGTGGCGGACGAGGCCAGACACGTGGCGCGAGTTGGAGCAGGAGCAATCCTC

3.3 GACAATGACGTGGCGGACGAGGCCAGACACGTGGCGCGAGTTGGAGCAGGAGCAATCCTC

3.15 GACAATGACGTGGCGGACGAGGCCAGACACGTGGCGCGAGTTGGAGCAGGAGCAATCCTC

3.2 GACAATGACGTGGCGGACGAGGCCAGACACGTGGCGCGAGTTGGAGCAGGAGCAATCCTC

************************************************************

3.1 GGGCAGCACAGCCTAGTAGCAAATGGCCTCACCAAGTATTATGGGAAACACCTTGCAGTC

APHIS-S GGGCAGCACAGCCTAGTAGCAAATGGCCTCACCAAGTATTATGGGAAACACCTTGCAGTC

3.3 GGGCAGCACAGCCTAGTAGCAAATGGCCTCACCAAGTATTATGGGAAACACCTTGCAGTC

3.15 GGGCAGCACAGCCTAGTAGCAAATGGCCTCACCAAGTATTATGGGAAACACCTTGCAGTC

3.2 GGGCAGCACAGCCTAGTAGCAAATGGCCTCACCAAGTATTATGGGAAACACCTTGCAGTC

************************************************************

3.1 AATCAAGTGTCATTCACCGTGGGCGACACGGAATGCTTTGGTCTTCTGGGTGTGAACGGC

APHIS-S AATCAAGTGTCATTCACCGTGGGCGACACGGAATGCTTTGGTCTTCTGGGTGTGAACGGC

3.3 AATCAAGTGTCATTCACCGTGGGCGACACGGAATGCTTTGGTCTTCTGGGTGTGAACGGC

3.15 AATCAAGTGTCATTCACCGTGGGCGACACGGAATGCTTTGGTCTTCTGGGTGTGAACGGC

3.2 AATCAAGTGTCATTCACCGTGGGCGACACGGAATGCTTTGGTCTTCTGGGTGTGAACGGC

************************************************************

3.1 GCCGGTAAGACGACCACCTTCAAGATGTTGATGGGAGATGAGACCGTCTCCAGCGGAGAT

APHIS-S GCCGGTAAGACGACCACCTTCAAGATGTTGATGGGAGATGAGACCGTCTCCAGCGGAGAT

3.3 GCCGGTAAGACGACCACCTTCAAGATGTTGATGGGAGATGAGACCGTCTCCAGCGGAGAT

3.15 GCCGGTAAGACGACCACCTTCAAGATGTTGATGGGAGATGAGACCGTCTCCAGCGGAGAT

3.2 GCCGGTAAGACGACCACCTTCAAGATGTTGATGGGAGATGAGACCGTCTCCAGCGGAGAT

************************************************************

3.1 GCCTTCGTGAGTGGCCATTCTGTCAAGACTAATATCACTCAAGTTTACAAAAATATTGGT

APHIS-S GCCTTCGTGAGTGGCCATTCTGTCAAGACTAATATCACTCAAGTTTACAAAAATATTGGT

3.3 GCCTTCGTGAGTGGCCATTCTGTCAAGACTAATATCACTCAAGTTTACAAAAATATTGGT

3.15 GCCTTCGTGAGTGGCCATTCTGTCAAGACTAATATCACTCAAGTTTACAAAAATATTGGT

3.2 GCCTTCGTGAGTGGCCATTCTGTCAAGACTAATATCACTCAAGTTTACAAAAATATTGGT

************************************************************

3.1 TACTGTCCGCAATTCGAAGCGACATTCGGCGAGCTGACGGGACGCGAGACACTACGGCTG

APHIS-S TACTGTCCGCAATTCGAAGCGACATTCGGCGAGCTGACGGGACGCGAGACACTACGGCTG

3.3 TACTGTCCGCAATTCGAAGCGACATTCGGCGAGCTGACGGGACGCGAGACACTACGGCTG

3.15 TACTGTCCGCAATTCGAAGCGACATTCGGCGAGCTGACGGGACGCGAGACACTACGGCTG

3.2 TACTGTCCGCAATTCGAAGCGACATTCGGCGAGCTGACGGGACGCGAGACACTACGGCTG

************************************************************

3.1 TTCTCGGCGCTGCGAGGGTTGCCAGTGCGAGGCGCCACGCTCCACGCGGAGGCCTTAGCA

APHIS-S TTCTCGGCGCTGCGAGGGTTGCCAGTGCGAGGCGCCACGCTCCACGCGGAGGCCTTAGCA

3.3 TTCTCGGCGCTGCGAGGGTTGCCAGTGCGAGGCGCCACGCTCCACGCGGAGGCCTTAGCA

3.15 TTCTCGGCGCTGCGAGGGTTGCCAGTGCGAGGCGCCACGCTCCACGCGGAGGCCTTAGCA

3.2 TTCTCGGCGCTGCGAGGGTTGCCAGTGCGAGGCGCCACGCTCCACGCGGAGGCCTTAGCA

************************************************************

3.1 CATGCTCTTGGTTTCTATAAGCATCTTGATAAAAGGGTGGACCACTACTCTGGTGGCAAC

APHIS-S CATGCTCTTGGTTTCTATAAGCATCTTGATAAAAGGGTGGACCACTACTCTGGTGGCAAC

3.3 CATGCTCTTGGTTTCTATAAGCATCTTGATAAAAGGGTGGACCACTACTCTGGTGGCAAC

3.15 CATGCTCTTGGTTTCTATAAGCATCTTGATAAAAGGGTGGACCACTACTCTGGTGGCAAC

3.2 CATGCTCTTGGTTTCTATAAGCATCTTGATAAAAGGGTGGACCACTACTCTGGTGGCAAC

************************************************************

3.1 AAGCGCAAGTTGAGCACGGCTGTGGCGTTGCTGGGGCGCACGCGGCTTATATTCGTCGAC

APHIS-S AAGCGCAAGTTGAGCACGGCTGTGGCGTTGCTGGGGCGCACGCGGCTTATATTCGTCGAC

3.3 AAGCGCAAGTTGAGCACGGCTGTGGCGTTGCTGGGGCGCACGCGGCTTATATTCGTCGAC

3.15 AAGCGCAAGTTGAGCACGGCTGTGGCGTTGCTGGGGCGCACGCGGCTTATATTCGTCGAC

3.2 AAGCGCAAGTTGAGCACGGCTGTGGCGTTGCTGGGGCGCACGCGGCTTATATTCGTCGAC

************************************************************

3.1 GAACCCACTACTGGAGTCGATCCTGCTGCTAAGAGACAGATGTGGAACGCGGTTCGAGAA

APHIS-S GAACCCACTACTGGAGTCGATCCTGCTGCTAAGAGACAGATGTGGAACGCGGTTCGAGAA

3.3 GAACCCACTACTGGAGTCGATCCTGCTGCTAAGAGACAGATGTGGAACGCGGTTCGAGAA

3.15 GAACCCACTACTGGAGTCGATCCTGCTGCTAAGAGACAGATGTGGAACGCGGTTCGAGAA

3.2 GAACCCACTACTGGAGTCGATCCTGCTGCTAAGAGACAGATGTGGAACGCGGTTCGAGAA

************************************************************

3.1 GCTCGCCGGTCGGGTCGTGGTGTGGTGCTGACATCACACAGCATGGAGGAGTGTGAGGCT

APHIS-S GCTCGCCGGTCGGGTCGTGGTGTGGTGCTGACATCACACAGCATGGAGGAGTGTGAGGCT

3.3 GCTCGCCGGTCGGGTCGTGGTGTGGTGCTGACATCACACAGCATGGAGGAGTGTGAGGCT

3.15 GCTCGCCGGTCGGGTCGTGGTGTGGTGCTGACATCACACAGCATGGAGGAGTGTGAGGCT

3.2 GCTCGCCGGTCGGGTCGTGGTGTGGTGCTGACATCACACAGCATGGAGGAGTGTGAGGCT

************************************************************

3.1 CTGTGCTCGCGGCTCACAATCATGGTCAACGGACAGTTCCAGTGCCTCGGCACGCCGCAA

APHIS-S CTGTGCTCGCGGCTCACAATCATGGTCAACGGACAGTTCCAGTGCCTCGGCACGCCGCAA

3.3 CTGTGCTCGCGGCTCACAATCATGGTCAACGGACAGTTCCAGTGCCTCGGCACGCCGCAA

3.15 CTGTGCTCGCGGCTCACAATCATGGTCAACGGACAGTTCCAGTGCCTCGGCACGCCGCAA

3.2 CTGTGCTCGCGGCTCACAATCATGGTCAACGGACAGTTCCAGTGCCTCGGCACGCCGCAA

************************************************************

3.1 CATTTAAAGAATAAGTTCTCTGAAGGTTTCACATTGACAATTAAAATTAAAGTGGACGAC

APHIS-S CATTTAAAGAATAAGTTCTCTGAAGGTTTCACATTGACAATTAAAATTAAAGTGGACGAC

3.3 CATTTAAAGAATAAGTTCTCTGAAGGTTTCACATTGACAATTAAAATTAAAGTGGACGAC

3.15 CATTTAAAGAATAAGTTCTCTGAAGGTTTCACATTGACAATTAAAATTAAAGTGGACGAC

3.2 CATTTAAAGAATAAGTTCTCTGAAGGTTTCACATTGACAATTAAAATTAAAGTGGACGAC

************************************************************

3.1 GAGACGAAGACTGTACGGCCTGAAGTCTGCGATGCTGTGAAGCATTACGTCAGTACCAAC

APHIS-S GAGACGAAGACTGTACGGCCTGAAGTCTGCGATGCTGTGAAGCATTACGTCAGTACCAAC

3.3 GAGACGAAGACTGTACGGCCTGAAGTCTGCGATGCTGTGAAGCATTACGTCAGTACCAAC

3.15 GAGACGAAGACTGTACGGCCTGAAGTCTGCGATGCTGTGAAGCATTACGTCAGTACCAAC

3.2 GAGACGAAGACTGTACGGCCTGAAGTCTGCGATGCTGTGAAGCATTACGTCAGTACCAAC

************************************************************

3.1 TTCAGAGAGCCGAAGATTATGGAGGAGTACCAGGGTCTGTTAACATACTATTTGCCAGAC

APHIS-S TTCAGAGAGCCGAAGATTATGGAGGAGTACCAGGGTCTGTTAACATACTATTTGCCAGAC

3.3 TTCAGAGAGCCGAAGATTATGGAGGAGTACCAGGGTCTGTTAACATACTATTTGCCAGAC

3.15 TTCAGAGAGCCGAAGATTATGGAGGAGTACCAGGGTCTGTTAACATACTATTTGCCAGAC

3.2 TTCAGAGAGCCGAAGATTATGGAGGAGTACCAGGGTCTGTTAACATACTATTTGCCAGAC

************************************************************

3.1 AAGTCGGTGGCGTGGTCCAGAATGTTCGGCATAATGGAGGCGGCCAAACGCGACCTCCCC

APHIS-S AAGTCGGTGGCGTGGTCCAGAATGTTCGGCATAATGGAGGCGGCCAAACGCGACCTCCCC

3.3 AAGTCGGTGGCGTGGTCCAGAATGTTCGGCATAATGGAGGCGGCCAAACGCGACCTCCCC

3.15 AAGTCGGTGGCGTGGTCCAGAATGTTCGGCATAATGGAGGCGGCCAAACGCGACCTCCCC

3.2 AAGTCGGTGGCGTGGTCCAGAATGTTCGGCATAATGGAGGCGGCCAAACGCGACCTCCCC

************************************************************

3.1 GTCGAAGACTACAGCATATCACAAACTACCCTCGAG------------------------

APHIS-S GTCGAAGACTACAGCATATCACAAACTACCCTCGAGCAGATATTCCTACAGTTCACAAAG

3.3 GTCGAAGACTACAGCATATCACAAACTACCCTCGAG------------------------

3.15 GTCGAAGACTACAGCATATCACAAACTACCCTCGAG------------------------

3.2 GTCGAAGACTACAGCATATCACAAACTACCCTCGAG------------------------

************************************

3.1 ---------------------------

APHIS-S TATCAACATGAAGCACAACAGACATAA

3.3 ---------------------------

3.15 ---------------------------

3.2 ---------------------------

**B**

5B.8 ATGCGGGCGCGTGGAGAGCGGAARGAGGCGGGCTCATGGGTGAAGTTTAGGCTGTTGATG

5A.7 ATGCGGGCGCGTGGAGAGCGGAAGGAGGCGGGCTCATGGGTGAAGTTTAGGCTGTTGATG

APHIS-S ATGCGGGCGCGTGGAGAGCGGAAGGAGGCGGGCTCATGGGTGAAGTTTAGGCTGTTGATG

5A.11 ATGCGGGCGCGTGGAGAGCGGAAGGAGGCGGGCTCATGGGTGAAGTTTAGGCTGTTGATG

5A.12 ATGCGGGCGCGTGGAGAGCGGAAGGAGGCGGGCTCATGGGTGAAGTTTAGGCTGTTGATG

5B.4 ATGCGGGCGCGTGGAGAGCGGAAGGAGGCGGGCTCATGGGTGAAGTTTAGGCTGTTGATG

5B.6 ATGCGGGCGCGTGGAGAGCGGAAGGAGGCGGGCTCATGGGTGAAGTTTAGGCTGTTGATG

*********************** ************************************

5B.8 TGGAAGAACTTCGTGCAGCAGTTGAGGCACCCAGTGCAGACGGCGGCTGAGCTGCTGCTA

5A.7 TGGAAGAACTTCGTGCAGCAGTTGAGGCACCCAGTGCAGACGGCGGCTGAGCTGCTGCTA

APHIS-S TGGAAGAACTTCGTGCAGCAGTTGAGGCACCCAGTGCAGACGGCGGCTGAGCTGCTGCTA

5A.11 TGGAAGAACTTCGTGCAGCAGTTGAGGCACCCAGTGCAGACGGCGGCTGAGCTGCTGCTA

5A.12 TGGAAGAACTTCGTGCAGCAGTTGAGGCACCCAGTGCAGACGGCGGCTGAGCTGCTGCTA

5B.4 TGGAAGAACTTCGTGCAGCAGTTGAGGCACCCAGTGCAGACGGCGGCTGAGCTGCTGCTA

5B.6 TGGAAGAACTTCGTGCAGCAGTTGAGGCACCCAGTGCAGACGGCGGCTGAGCTGCTGCTA

************************************************************

5B.8 CCAGTCCTAACCATGAGCCTGGTCCTGGTGCTACGGTCACAGATCGACCCCGAAGTCTTG

5A.7 CCAGTCCTAACCATGAGCCTGGTCCTGGTGCTACGGTCACAGATCGACCCCGAAGTCTTG

APHIS-S CCAGTCCTAACCATGAGCCTGGTCCTGGTGCTACGGTCACAGATCGACCCCGAAGTCTTG

5A.11 CCAGTCCTAACCATGAGCCTGGTCCTGGTGCTACGGTCACAGATCGACCCCGAAGTCTTG

5A.12 CCAGTCCTAACCATGAGCCTGGTCCTGGTGCTACGGTCACAGATCGACCCCGAAGTCTTG

5B.4 CCAGTCCTAACCATGAGCCTGGTCCTGGTGCTACGGTCACAGATCGACCCCGAAGTCTTG

5B.6 CCAGTCCTAACCATGAGCCTGGTCCTGGTGCTACGGTCACAGATCGACCCCGAAGTCTTG

************************************************************

5B.8 GAAACCAGAACCTACCCGCCAATACCAGCCCACACTTTAAACTATTCCGTGACTGTTTTG

5A.7 GAAACCAGAACCTACCCGCCAATACCAGCCCACACTTTAAACTATTCCGTGACTGTTTTG

APHIS-S GAAACCAGAACCTACCCGCCAATACCAGCCCACACTTTAAACTATTCCGTGACTGTTTTG

5A.11 GAAACCAGAACCTACCCGCCAATACCAGCCCACACTTTAAACTATTCCGTGACTGTTTTG

5A.12 GAAACCAGAACCTACCCGCCAATACCAGCCCACACTTTAAACTATTCCGTGACTGTTTTG

5B.4 GAAACCAGAACCTACCCGCCAATACCAGCCCACACTTTAAACTATTCCGTGACTGTTTTG

5B.6 GAAACCAGAACCTACCCGCCAATACCAGCCCACACTTTAAACTATTCCGTGACTGTTTTG

************************************************************

5B.8 ------------------------------------------------------------

5A.7 GGCGGAATGAATTTAACAAGAATGTCCATGGCATTCTCACCCGAGAATGCCGTATTGAGG

APHIS-S GGCGGAATGAATTTAACAAGAATGTCCATGGCATTCTCACCCGAGAATGCCGTATTGAGG

5A.11 GGCGGAATGAATTTAACAAGAATGTCCATGGCATTCTCACCCGAGAATGCCGTATTGAGG

5A.12 GGCGGAATGAATTTAACAAGAATGTCCATGGCATTCTCACCCGAGAATGCCGTATTGAGG

5B.4 ------------------------------------------------------------

5B.6 ------------------------------------------------------------

5B.8 ------------------------------------------------------------

5A.7 GACGTCGTATCCAGTGCTACAACAAAGTTACTGCTTAAAAACATGAGAGACCAAGTACTG

APHIS-S GACGTCGTATCCAGTGCTACAACAAAGTTACTGCTTAAAAACATGAGAGACCAAGTACTG

5A.11 GACGTCGTATCCAGTGCTACAACAAAGTTACTGCTTAAAAACATGAGAGACCAAGTACTG

5A.12 GACGTCGTATCCAGTGCTACAACAAAGTTACTGCTTAAAAACATGAGAGACCAAGTACTG

5B.4 ------------------------------------------------------------

5B.6 ------------------------------------------------------------

5B.8 ------------------------------------------------------------

5A.7 CCCATCATTGAGGCATTGCCAATAGAAATACCGCCGGGACTGGTAAACTCGTCACAGGTG

APHIS-S CCCATCATTGAGGCATTGCCAATAGAAATACCGCCGGGACTGGTAAACTCGTCACAGGTG

5A.11 CCCATCATTGAGGCATTGCCAATAGAAATACCGCCGGGACTGGTAAACTCGTCACAGGTG

5A.12 CCCATCATTGAGGCATTGCCAATAGAAATACCGCCGGGACTGGTAAACTCGTCACAGGTG

5B.4 ------------------------------------------------------------

5B.6 ------------------------------------------------------------

5B.8 ------------------------------------------------------------

5A.7 TACGAAATAGTTAAATTATTTGTCGACGAGAACGTTGTTACCGGATACAATAGCAGTGCG

APHIS-S TACGAAATAGTTAAATTATTTGTCGACGAGAACGTTGTTACCGGATACAATAGCAGTGCG

5A.11 TACGAAATAGTTAAATTATTTGTCGACGAGAACGTTGTTACCGGATACAATAGCAGTGCG

5A.12 TACGAAATAGTTAAATTATTTGTCGACGAGAACGTTGTTACCGGATACAATAGCAGTGCG

5B.4 ------------------------------------------------------------

5B.6 ------------------------------------------------------------

5B.8 ------------------------------------------------------------

5A.7 GCAATGAGAGGAATATACGCAGAGGAAGAAGCCACGAGAAGGGTGATAGCTGGCATAGAA

APHIS-S GCAATGAGAGGAATATACGCAGAGGAAGAAGCCACGAGAAGGGTGATAGCTGGCATAGAA

5A.11 GCAATGAGAGGAATATACGCAGAGGAAGAAGCCACGAGAAGGGTGATAGCTGGCATAGAA

5A.12 GCAATGAGAGGAATATACGCAGAGGAAGAAGCCACGAGAAGGGTGATAGCTGGCATAGAA

5B.4 ------------------------------------------------------------

5B.6 ------------------------------------------------------------

5B.8 ------------------------------------------------------------

5A.7 TTCGATGACTCATTGCGTGAAATAACGGAGCTACCACTAGACTTGTCGTATGCGCTTCGT

APHIS-S TTCGATGACTCATTGCGTGAAATAACGGAGCTACCACTAGACTTGTCGTATGCGCTTCGT

5A.11 TTCGATGACTCATTGCGTGAAATAACGGAGCTACCACTAGACTTGTCGTATGCGCTTCGT

5A.12 TTCGATGACTCATTGCGTGAAATAACGGAGCTACCACTAGACTTGTCGTATGCGCTTCGT

5B.4 ------------------------------------------------------------

5B.6 ------------------------------------------------------------

5B.8 ------------------------------------------------------------

5A.7 TTTCCGGAGAGACCTCGCTTGAATTCCTTCTTCATGACAGGCGGTCGGACTTGGCGCACA

APHIS-S TTTCCGGAGAGACCTCGCTTGAATTCCTTCTTCATGACAGGCGGTCGGACTTGGCGCACA

5A.11 TTTCCGGAGAGACCTCGCTTGAATTCCTTCTTCATGACAGGCGGTCGGACTTGGCGCACA

5A.12 TTTCCGGAGAGACCTCGCTTGAATTCCTTCTTCATGACAGGCGGTCGGACTTGGCGCACA

5B.4 ------------------------------------------------------------

5B.6 ------------------------------------------------------------

5B.8 ------------------------------------------------------------

5A.7 GATAACGTGTTTCCTATGTTCGAAGTTCCCGGACCTCGCTTTCCGTATTCATGGGAAGGT

APHIS-S GATAACGTGTTTCCTATGTTCGAAGTTCCCGGACCTCGCTTTCCGTATTCATGGGAAGGT

5A.11 GATAACGTGTTTCCTATGTTCGAAGTTCCCGGACCTCGCTTTCCGTATTCATGGGAAGGT

5A.12 GATAACGTGTTTCCTATGTTCGAAGTTCCCGGACCTCGCTTTCCGTATTCATGGGAAGGT

5B.4 ------------------------------------------------------------

5B.6 ------------------------------------------------------------

5B.8 ------------------------------------------------------------

5A.7 GGAAATGATCCAGGATACGTAAACGAGATGTTCATAGCCTTGCAGCACATGATATCTTCA

APHIS-S GGAAATGATCCAGGATACGTAAACGAGATGTTCATAGCCTTGCAGCACATGATATCTTCA

5A.11 GGAAATGATCCAGGATACGTAAACGAGATGTTCATAGCCTTGCAGCACATGATATCTTCA

5A.12 GGAAATGATCCAGGATACGTAAACGAGATGTTCATAGCCTTGCAGCACATGATATCTTCA

5B.4 ------------------------------------------------------------

5B.6 ------------------------------------------------------------

5B.8 ------------------------------------------------------------

5A.7 GAACTGGTATCTAAAGTGGCGGGAGTGAACCTAGACTTCGATGTGCACATACAGAGGTAC

APHIS-S GAACTGGTATCTAAAGTGGCGGGAGTGAACCTAGACTTCGATGTGCACATACAGAGGTAC

5A.11 GAACTGGTATCTAAAGTGGCGGGAGTGAACCTAGACTTCGATGTGCACATACAGAGGTAC

5A.12 GAACTGGTATCTGAAGTGGCGGGAGTGAACCTAGACTTCGATGTGCACATACAGAGGTAC

5B.4 ------------------------------------------------------------

5B.6 ------------------------------------------------------------

5B.8 ------------------------------------------------------------

5A.7 CCACATCCAGCATACATCATGGACTTGGCGAAGGAAGCCCTGCAGTTCCTCTTCCCATCA

APHIS-S CCACATCCAGCATACATCATGGACTTGGCGAAGGAAGCCCTGCAGTTCCTCTTCCCATCA

5A.11 CCACATCCAGCATACATCATGGACTTGGCGAAGGAAGCCCTGCAGTTCCTCTTCCCATCA

5A.12 CCACATCCAGCATACATCATGGACTTGGCGAAGGAAGCCCTGCAGTTCCTCTTCCCATCA

5B.4 ------------------------------------------------------------

5B.6 ------------------------------------------------------------

5B.8 ------------------------------------------------------------

5A.7 TTCATCATGATCAGCTTCAGTTACACCGCTATCAATATTATACGATCCGTGACCGTGGAA

APHIS-S TTCATCATGATCAGCTTCAGTTACACCGCTATCAATATTATACGATCCGTGACCGTGGAA

5A.11 TTCATCATGATCAGCTTCAGTTACACCGCTATCAATATTATACGATCCGTGACCGTGGAA

5A.12 TTCATCATGATCAGCTTCAGTTACACCGCTATCAATATTATACGATCCGTGACCGTGGAA

5B.4 ------------------------------------------------------------

5B.6 ------------------------------------------------------------

5B.8 ------------------------------------------------------------

5A.7 AAAGAAATGCAATTGAAGGAAACGATGAAGATCATGGGACTCCCAACGTGGCTGCATTGG

APHIS-S AAAGAAATGCAATTGAAGGAAACGATGAAGATCATGGGACTCCCAACGTGGCTGCATTGG

5A.11 AAAGAAATGCAATTGAAGGAAACGATGAAGATCATGGGACTCCCAACGTGGCTGCATTGG

5A.12 AAAGAAATGCAATTGAAGGAAACGATGAAGATCATGGGACTCCCAACGTGGCTGCATTGG

5B.4 ------------------------------------------------------------

5B.6 ------------------------------------------------------------

5B.8 ------------------------------------------------------------

5A.7 ATGGCATGGTTTTTTAAACAATTTATTTATTTGCTGATTGCTTCGGTTTTGATACTTGTT

APHIS-S ATGGCATGGTTTTTTAAACAATTTATTTATTTGCTGATTGCTTCGGTTTTGATACTTGTT

5A.11 ATGGCATGGTTTTTTAAACAATTTATTTATTTGCTGATTGCTTCGGTTTTGATACTTGTT

5A.12 ATGGCATGGTTTTTTAAACAATTTATTTATTTGCTGATTGCTTCGGTTTTGATACTTGTT

5B.4 ------------------------------------------------------------

5B.6 ------------------------------------------------------------

5B.8 ------------------------------------------------------------

5A.7 ATATTAAAGGTAAATTGGTTTACTACAGAAGAAGGCTTTAGCGACTATGCCGTATTCACT

APHIS-S ATATTAAAGGTAAATTGGTTTACTACAGAAGAAGGCTTTAGCGACTATGCCGTATTCACT

5A.11 ATATTAAAGGTAAATTGGTTTACTACAGAAGAAGGCTTTAGCGACTATGCCGTATTCACT

5A.12 ATATTAAAGGTAAATTGGTTTACTACAGAAGAAGGCTTTAGCGACTATGCCGTATTCACT

5B.4 ------------------------------------------------------------

5B.6 ------------------------------------------------------------

5B.8 ------------------------------------------------------------

5A.7 AATACACCTTGGACCGTCCTCTTCTTCTTCCTAACACTGTATCTTACGTGTACCATATTT

APHIS-S AATACACCTTGGACCGTCCTCTTCTTCTTCCTAACACTGTATCTTACGTGTACCATATTT

5A.11 AATACACCTTGGACCGTCCTCTTCTTCTTCCTAACACTGTATCTTACGTGTACCATATTT

5A.12 AATACACCTTGGACCGTCCTCTTCTTCTTCCTAACACTGTATCTTACGTGTACCATATTT

5B.4 ------------------------------------------------------------

5B.6 ------------------------------------------------------------

5B.8 ------------------------------------------------------------

5A.7 TTCTGTTTCATGATAAGTGGTTTCTTTTCAAAAGCCAGTACAGCGGCGTTGTTTGGTGGG

APHIS-S TTCTGTTTCATGATAAGTGGTTTCTTTTCAAAAGCCAGTACAGCGGCGTTGTTTGGTGGG

5A.11 TTCTGTTTCATGATAAGTGGTTTCTTTTCAAAAGCCAGTACAGCGGCGTTGTTTGGTGGG

5A.12 TTCTGTTTCATGATAAGTGGTTTCTTTTCAAAAGCCAGTACAGCGGCGTTGTTTGGTGGG

5B.4 ------------------------------------------------------------

5B.6 ------------------------------------------------------------

5B.8 ------------------------------------------------------------

5A.7 GTGATCTGGTTTCTGACGTATATCCCCGCATTCCTCCTGGCTATGGACGTGAACATGTCT

APHIS-S GTGATCTGGTTTCTGACGTATATCCCCGCATTCCTCCTGGCTATGGACGTGAACATGTCT

5A.11 GTGATCTGGTTTCTGACGTATATCCCCGCATTCCTCCTGGCTATGGACGTGAACATGTCT

5A.12 GTGATCTGGTTTCTGACGTATATCCCCGCATTCCTCCTGGCTATGGACGTGAACATGTCT

5B.4 ------------------------------------------------------------

5B.6 ------------------------------------------------------------

5B.8 ------------------------------------------------------------

5A.7 ACCTCTCTACAAGCGGTCACCTGCCTAATGCTCAACTCCGCCATGTCTTACGGCTTCCAG

APHIS-S ACCTCTCTACAAGCGGTCACCTGCCTAATGCTCAACTCCGCCATGTCTTACGGCTTCCAG

5A.11 ACCTCTCTACAAGCGGTCACCTGCCTAATGCTCAACTCCGCCATGTCTTACGGCTTCCAG

5A.12 ACCTCTCTACAAGCGGTCACCTGCCTAATGCTCAACTCCGCCATGTCTTACGGCTTCCAG

5B.4 ------------------------------------------------------------

5B.6 ------------------------------------------------------------

5B.8 -----------------------------AATGCAGTGGGGTGATTTTATGACGTCACCA

5A.7 CTGTTACTGGCCCGGGAAAGTACCGGAGGAATGCAGTGGGGTGATTTTATGACGTCACCA

APHIS-S CTGTTACTGGCCCGGGAAAGTACCGGAGGAATGCAGTGGGGTGATTTTATGACGTCACCA

5A.11 CTGTTACTGGCCCGGGAAAGTACCGGAGGAATGCAGTGGGGTGATTTTATGACGTCACCA

5A.12 CTGTTACTGGCCCGGGAAAGTACCGGAGGAATGCAGTGGGGTGATTTTATGACGTCACCA

5B.4 -----------------------------AATGCAGTGGGGTGATTTTATGACGTCACCA

5B.6 -----------------------------AATGCAGTGGGGTGATTTTATGACGTCACCA

*******************************

5B.8 GCAACGGACTCGTCACGATTCGTATTCGGTCACGTCGTTATAATGATGGCTTTGAACTGT

5A.7 GCAACGGACTCGTCACGATTCGTATTCGGTCACGTCGTTATAATGATGGCTTTGAACTGT

APHIS-S GCAACGGACTCGTCACGATTCGTATTCGGTCACGTCGTTATAATGATGGCTTTGAACTGT

5A.11 GCAACGGACTCGTCACGATTCGTATTCGGTCACGTCGTTATAATGATGGCTTTGAACTGT

5A.12 GCAACGGACTCGTCACGATTCGTATTCGGTCACGTCGTTATAATGATGGCTTTGAACTGT

5B.4 GCAACGGACTCGTCACGATTCGTATTCGGTCACGTCGTTATAATGATGGCTTTGAACTGT

5B.6 GCAACGGACTCGTCACGATTCGTATTCGGTCACGTCGTTATAATGATGGCTTTGAACTGT

************************************************************

5B.8 GTGCTCTACATGTTGATTGCCCTATATCTAGAGCAAGTACTACCCGGGCCGTATGGCACA

5A.7 GTGCTCTACATGTTGATTGCCCTATATCTAGAGCAAGTACTACCCGGGCCGTATGGCACA

APHIS-S GTGCTCTACATGTTGATTGCCCTATATCTAGAGCAAGTACTACCCGGGCCGTATGGCACA

5A.11 GTGCTCTACATGTTGATTGCCCTATATCTAGAGCAAGTACTACCCGGGCCGTATGGCACA

5A.12 GTGCTCTACATGTTGATTGCCCTATATCTAGAGCAAGTACTACCCGGGCCGTATGGCACA

5B.4 GTGCTCTACATGTTGATTGCCCTATATCTAGAGCAAGTACTACCCGGGCCGTATGGCACA

5B.6 GTGCTCTACATGTTGATTGCCCTATATCTAGAGCAAGTACTACCCGGGCCGTATGGCACA

************************************************************

5B.8 CCGAAGCCCTGGTATTTCTTCGTCCAAAGACAGTTCTGGTGTAGCAGCAAAACTACTCAT

5A.7 CCGAAGCCCTGGTATTTCTTCGTCCAAAGACAGTTCTGGTGTAGCAGCAAAACTACTCAT

APHIS-S CCGAAGCCCTGGTATTTCTTCGTCCAAAGACAGTTCTGGTGTAGCAGCAAAACTACTCAT

5A.11 CCGAAGCCCTGGTATTTCTTCGTCCAAAGACAGTTCTGGTGTAGCAGCAAAACTACTCAT

5A.12 CCGAAGCCCTGGTATTTCTTCGTCCAAAGACAGTTCTGGTGTAGCAGCAAAACTACTCAT

5B.4 CCGAAGCCCTGGTATTTCTTCGTCCAAAGACAGTTCTGGTGTAGCAGCAAAACTACTCAT

5B.6 CCGAAGCCCTGGTATTTCTTCGTCCAAAGACAGTTCTGGTGTAGCAGCAAAACTACTCAT

************************************************************

5B.8 GATATCGGTACAGACAACAGCGACACATCAAGTTTAACAAAAGAAAGCGACCCTACAGAC

5A.7 GATATCGGTACAGACAACAGCGACACATCAAGTTTAACAAAAGAAAGCGACCCTACAGAC

APHIS-S GATATCGGTACAGACAACAGCGACACATCAAGTTTAACAAAAGAAAGCGACCCTACAGAC

5A.11 GATATCGGTACAGACAACAGCGACACATCAAGTTTAACAAAAGAAAGCGACCCTACAGAC

5A.12 GATATCGGTACAGACAACAGCGACACATCAAGTTTAACAAAAGAAAGCGACCCTACAGAC

5B.4 GATATCGGTACAGACAACAGCGACACATCAAGTTTAACAAAAGAAAGCGACCCTACAGAC

5B.6 GATATCGGTACAGACAACAGCGACACATCAAGTTTAACAAAAGAAAGCGACCCTACAGAC

************************************************************

5B.8 CTTCCGATTGGAGTTAAAATACAAAACCTTAAAAAGGTTTACGGGAGCAACGTTGCGGTA

5A.7 CTTCCGATTGGAGTTAAAATACAAAACCTTAAAAAGGTTTACGGGAGCAACGTTGCGGTA

APHIS-S CTTCCGATTGGAGTTAAAATACAAAACCTTAAAAAGGTTTACGGGAGCAACGTTGCGGTA

5A.11 CTTCCGATTGGAGTTAAAATACAAAACCTTAAAAAGGTTTACGGGAGCAACGTTGCGGTA

5A.12 CTTCCGATTGGAGTTAAAATACAAAACCTTAAAAAGGTTTACGGGAGCAACGTTGCGGTA

5B.4 CTTCCGATTGGAGTTAAAATACAAAACCTTAAAAAGGTTTACGGGAGCAACGTTGCGGTA

5B.6 CTTCCGATTGGAGTTAAAATACAAAACCTTAAAAAGGTTTACGGGAGCAACGTTGCGGTA

************************************************************

5B.8 AACAATTTATCCCTCAACATTTACGACGACCAAATCACGGTTCTACTTGGACACAACGGA

5A.7 AACAATTTATCCCTCAACATTTACGACGACCAAATCACGGTTCTACTTGGACACAACGGA

APHIS-S AACAATTTATCCCTCAACATTTACGACGACCAAATCACGGTTCTACTTGGACACAACGGA

5A.11 AACAATTTATCCCTCAACATTTACGACGACCAAATCACGGTTCTACTTGGACACAACGGA

5A.12 AACAATTTATCCCTCAACATTTACGACGACCAAATCACGGTTCTACTTGGACACAACGGA

5B.4 AACAATTTATCCCTCAACATTTACGACGACCAAATCACGGTTCTACTTGGACACAACGGA

5B.6 AACAATTTATCCCTCAACATTTACGACGACCAAATCACGGTTCTACTTGGACACAACGGA

************************************************************

5B.8 GCGGGAAAATCCACAACCATTTCAATGCTCACAG--------------------------

5A.7 GCGGGAAAATCCACAACCATTTCAATGCTCACAGGTAACGTGGACATAACCAGCGGGTCG

APHIS-S GCGGGAAAATCCACAACCATTTCAATGCTCACAGGTAACGTGGACATAACCAGCGGGTCG

5A.11 GCGGGAAAATCCACAACCATTTCAATGCTCACAGGTAACGTGGACATAACCAGCGGGTCG

5A.12 GCGGGAAAATCCACAACCATTTCAATGCTCACAGGTAACGTGGACATAACCAGCGGGTCG

5B.4 GCGGGAAAATCCACAACCATTTCAATGCTCACAGGTAACGTGGACATAACCAGCGGGTCG

5B.6 GCGGGAAAATCCACAACCATTTCAATGCTCACAGGTAACGTGGACATAACCAGCGGGTCG

**********************************

5B.8 ------------------------------------------------------------

5A.7 GTGACGGTGGCTGGCTACGACATAGAAAAACAAACAAGTTCAGCACGCTCACACATTGGA

APHIS-S GTGACGGTGGCTGGCTACGACATAGAAAAACAAACAAGTTCAGCACGCTCACACATTGGA

5A.11 GTGACGGTGGCTGGCTACGACATAGAAAAACAAACAAGTTCAGCACGCTCACACATTGGA

5A.12 GTGACGGTGGCTGGCTACGACATAGAAAAACAAACAAGTTCAGCACGCTCACACATTGGA

5B.4 GTGACGGTGGCTGGCTACGACATAGAAAAACAAACAAGTTCAGCACGCTCACACATTGGA

5B.6 GTGACGGTGGCTGGCTACGACATAGAAAAACAAACAAGTTCAGCACGCTCACACATTGGA

5B.8 ---------------------------------------------------------TTC

5A.7 CTCTGCCCTCAACATAACGTACTCTTCAACGAACTCACAGTCAAAGAACATTTACAGTTC

APHIS-S CTCTGCCCTCAACATAACGTACTCTTCAACGAACTCACAGTCAAAGAACATTTACAGTTC

5A.11 CTCTGCCCTCAACATAACGTACTCTTCAACGAACTCACAGTCAAAGAACATTTACAGTTC

5A.12 CTCTGCCCTCAACATAACGTACTCTTCAACGAACTCACAGTCAAAGAACATTTACAGTTC

5B.4 CTCTGCCCTCAACATAACGTACTCTTCAACGAACTCACAGTCAAAGAACATTTACAGTTC

5B.6 CTCTGCCCTCAACATAACGTACTCTTCAACGAACTCACAGTCAAAGAACATTTACAGTTC

***

5B.8 TTCTCTCGTCTGAAAGGCTTCAGCGGTAAAGAGTTGGATGAAGAAATTGAGACGCTTATT

5A.7 TTCTCTCGTCTGAAAGGCTTCAGCGGTAAAGAGTTGGATGAAGAAATTGAGACGCTTATT

APHIS-S TTCTCTCGTCTGAAAGGCTTCAGCGGTAAAGAGTTGGATGAAGAAATTGAGACGCTTATT

5A.11 TTCTCTCGTCTGAAAGGCTTCAGCGGTAAAGAGTTGGATGAAGAAATTGAGACGCTTATT

5A.12 TTCTCTCGTCTGAAAGGCTTCAGCGGTAAAGAGTTGGATGAAGAAATTGAGACGCTTATT

5B.4 TTCTCTCGTCTGAAAGGCTTCAGCGGTAAAGAGTTGGATGAAGAAATTGAGACGCTTATT

5B.6 TTCTCTCGTCTGAAAGGCTTCAGCGGTAAAGAGTTGGATGAAGAAATTGAGACGCTTATT

************************************************************

5B.8 GAAAAATTGGAATTGCAAGAAAAGAGGGATTACCAATCAGCGGGGTTATCAGGGGGACAG

5A.7 GAAAAATTGGAATTGCAAGAAAAGAGGGATTACCAATCAGCGGGGTTATCAGGGGGACAG

APHIS-S GAAAAATTGGAATTGCAAGAAAAGAGGGATTACCAATCAGCGGGGTTATCAGGGGGACAG

5A.11 GAAAAATTGGAATTGCAAGAAAAGAGGGATTACCAATCAGCGGGGTTATCAGGGGGACAG

5A.12 GAAAAATTGGAATTGCAAGAAAAGAGGGATTACCAATCAGCGGGGTTATCAGGGGGACAG

5B.4 GAAAAATTGGAATTGCAAGAAAAGAGGGATTACCAATCAGCGGGGTTATCAGGGGGACAG

5B.6 GAAAAATTGGAATTGCAAGAAAAGAGGGATTACCAATCAGCGGGGTTATCAGGGGGACAG

************************************************************

5B.8 AAGCGACGATTAGGAGTGGGCGTC-------GCGGGGCGGCTAAAGTGGTTCTACTGGAC

5A.7 AAGCGACGATTAGGAGTGGGCGTCGCGCTATGCGGGGCGGCTAAAGTGGTTCTACTGGAC

APHIS-S AAGCGACGATTAGGAGTGGGCGTCGCGCTATGCGGGGCGGCTAAAGTGGTTCTACTGGAC

5A.11 AAGCGACGATTAGGAGTGGGCGTCGCGCTATGCGGGGCGGCTAAAGTGGTTCTACTGGAC

5A.12 AAGCGACGATTAGGAGTGGGCGTCGCGCTATGCGGGGCGGCTAAAGTGGTTCTACTGGAC

5B.4 AAGCGACGATTAGGAGTGGGCGTC-------GCGGGGCGGCTAAAGTGGTTCTACTGGAC

5B.6 AAGCGACGATTAGGAGTGGGCGTC-------GCGGGGCGGCTAAAGTGGTTCTACTGGAC

************************ *****************************

5B.8 GAGCCCACTTCTGGCATGGACCCGGCCTCACGTCGTGCCCTATGGGACTTGTTGCAGAGA

5A.7 GAGCCCACTTCTGGCATGGACCCGGCCTCACGTCGTGCCCTATGGGACTTGTTGCAGAGA

APHIS-S GAGCCCACTTCTGGCATGGACCCGGCCTCACGTCGTGCCCTATGGGACTTGTTGCAGAGA

5A.11 GAGCCCACTTCTGGCATGGACCCGGCCTCACGTCGTGCCCTATGGGACTTGTTGCAGAGA

5A.12 GAGCCCACTTCTGGCATGGACCCGGCCTCACGTCGTGCCCTATGGGACTTGTTGCAGAGA

5B.4 GAGCCCACTTCTGGCATGGACCCGGCCTCACGTCGTGCCCTATGGGACTTGTTGCAGAGA

5B.6 GAGCCCACTTCTGGCATGGACCCGGCCTCACGTCGTGCCCTATGGGACTTGTTGCAGAGA

************************************************************

5B.8 GAGAAGAAAGGTCGATCGATGATCCTGACGACACACTTCATGGACGAAGCGGACATATTA

5A.7 GAGAAGAAAGGTCGATCGATGATCCTGACGACACACTTCATGGACGAAGCGGACATATTA

APHIS-S GAGAAGAAAGGTCGATCGATGATCCTGACGACACACTTCATGGACGAAGCGGACATATTA

5A.11 GAGAAGAAAGGTCGATCGATGATCCTGACGACACACTTCATGGACGAAGCGGACATATTA

5A.12 GAGAAGAAAGGTCGATCGATGATCCTGACGACACACTTCATGGACGAAGCGGACATATTA

5B.4 GAGAAGAAAGGTCGATCGATGATCCTGACGACACACTTCATGGACGAAGCGGACATATTA

5B.6 GAGAAGAAAGGTCGATCGATGATCCTGACGACACACTTCATGGACGAAGCGGACATATTA

************************************************************

5B.8 GGGGATAGAGTTGCCATTATGGCGGACGGTCGTCTCCAGTGCGTGGGCTCACCTTACTTC

5A.7 GGGGATAGAGTTGCCATTATGGCGGACGGTCGTCTCCAGTGCGTGGGCTCACCTTACTTC

APHIS-S GGGGATAGAGTTGCCATTATGGCGGACGGTCGTCTCCAGTGCGTGGGCTCACCTTACTTC

5A.11 GGGGATAGAGTTGCCATTATGGCGGACGGTCGTCTCCAGTGCGTGGGCTCACCTTACTTC

5A.12 GGGGATAGAGTTGCCATTATGGCGGACGGTCGTCTCCAGTGCGTGGGCTCACCTTACTTC

5B.4 GGGGATAGAGTTGCCATTATGGCGGACGGTCGTCTCCAGTGCGTGGGCTCACCTTACTTC

5B.6 GGGGATAGAGTTGCCATTATGGCGGACGGTCGTCTCCAGTGCGTGGGCTCACCTTACTTC

************************************************************

5B.8 CTCAAGAGACACTATGGAGTCGGCTACACGCTAGTTGTGGTCAAGAAGGAAGATTTCCGA

5A.7 CTCAAGAGACACTATGGAGTCGGCTACACGCTAGTTGTGGTCAAGAAGGAAGATTTCCGA

APHIS-S CTCAAGAGACACTATGGAGTCGGCTACACGCTAGTTGTGGTCAAGAAGGAAGATTTCCGA

5A.11 CTCAAGAGACACTATGGAGTCGGCTACACGCTAGTTGTGGTCAAGAAGGAAGATTTCCGA

5A.12 CTCAAGAGACACTATGGAGTCGGCTACACGCTAGTTGTGGTCAAGAAGGAAGATTTCCGA

5B.4 CTCAAGAGACACTATGGAGTCGGCTACACGCTAGTTGTGGTCAAGAAGGAAGATTTCCGA

5B.6 CTCAAGAGACACTATGGAGTCGGCTACACGCTAGTTGTGGTCAAGAAGGAAGATTTCCGA

************************************************************

5B.8 CTGGACACCTGCACAGAGCTGATCAATAGATACATCCCTGGAACTGTTGTGAAGGAAGAC

5A.7 CTGGACACCTGCACAGAGCTGATCAATAGATACATCCCTGGAACTGTTGTGAAGGAAGAC

APHIS-S CTGGACACCTGCACAGAGCTGATCAATAGATACATCCCTGGAACTGTTGTGAAGGAAGAC

5A.11 CTGGACACCTGCACAGAGCTGATCAATAGATACATCCCTGGAACTGTTGTGAAGGAAGAC

5A.12 CTGGACACCTGCACAGAGCTGATCAATAGATACATCCCTGGAACTGTTGTGAAGGAAGAC

5B.4 CTGGACACCTGCACAGAGCTGATCAATAGATACATCCCTGGAACTGTTGTGAAGGAAGAC

5B.6 CTGGACACCTGCACAGAGCTGATCAATAGATACATCCCTGGAACTGTTGTGAAGGAAGAC

************************************************************

5B.8 CGAGGCACTGAAGTGACATATAGCATGACTAATGAGTATTCGCACGTGTTTGAATCTATG

5A.7 CGAGGCACTGAAGTGACATATAGCATGACTAATGAGTATTCGCACGTGTTTGAATCTATG

APHIS-S CGAGGCACTGAAGTGACATATAGCATGACTAATGAGTATTCGCACGTGTTTGAATCTATG

5A.11 CGAGGCACTGAAGTGACATATAGCATGACTAATGAGTATTCGCACGTGTTTGAATCTATG

5A.12 CGAGGCACTGAAGTGACATATAGCATGACTAATGAGTATTCGCACGTGTTTGAATCTATG

5B.4 CGAGGCACTGAAGTGACATATAGCATGACTAATGAGTATTCGCACGTGTTTGAATCTATG

5B.6 CGAGGCACTGAAGTGACATATAGCATGACTAATGAGTATTCGCACGTGTTTGAATCTATG

************************************************************

5B.8 CTGCGCGATTTGGAGGCAAAGGCCGATGAGATAAACTTTAAAAACTACGGCCTACTGGCT

5A.7 CTGCGCGATTTGGAGGCAAAGGCCGATGAGATAAACTTTAAAAACTACGGCCTACTGGCT

APHIS-S CTGCGCGATTTGGAGGCAAAGGCCGATGAGATAAACTTTAAAAACTACGGCCTACTGGCT

5A.11 CTGCGCGATTTGGAGGCAAAGGCCGATGAGATAAACTTTAAAAACTACGGCCTACTGGCT

5A.12 CTGCGCGATTTGGAGGCAAAGGCCGATGAGATAAACTTTAAAAACTACGGCCTACTGGCT

5B.4 CTGCGCGATTTGGAGGCAAAGGCCGATGAGATAAACTTTAAAAACTACGGCCTACTGGCT

5B.6 CTGCGCGATTTGGAGGCAAAGGCCGATGAGATAAACTTTAAAAACTACGGCCTACTGGCT

************************************************************

5B.8 ACTACATTAGAAGATGTGTTCATGTCCGTGGGCACAGATGTGGTCGCAACTTCAGATGTG

5A.7 ACTACATTAGAAGATGTGTTCATGTCCGTGGGCACAGATGTGGTCGCAACTTCAGATGTG

APHIS-S ACTACATTAGAAGATGTGTTCATGTCCGTGGGCACAGATGTGGTCGCAACTTCAGATGTG

5A.11 ACTACATTAGAAGATGTGTTCATGTCCGTGGGCACAGATGTGGTCGCAACTTCAGATGTG

5A.12 ACTACATTAGAAGATGTGTTCATGTCCGTGGGCACAGATGTGGTCGCAACTTCAGATGTG

5B.4 ACTACATTAGAAGATGTGTTCATGTCCGTGGGCACAGATGTGGTCGCAACTTCAGATGTG

5B.6 ACTACATTAGAAGATGTGTTCATGTCCGTGGGCACAGATGTGGTCGCAACTTCAGATGTG

************************************************************

5B.8 GACGACAATACAACCGTTTCATCTAGTGCTGATACTCTAGCATTTGAATATGATTCTTTA

5A.7 GACGACAATACAACCGTTTCATCTAGTGCTGATACTCTAGCATTTGAATATGATTCTTTA

APHIS-S GACGACAATACAACCGTTTCATCTAGTGCTGATACTCTAGCATTTGAATATGATTCTTTA

5A.11 GACGACAATACAACCGTTTCATCTAGTGCTGATACTCTAGCATTTGAATATGATTCTTTA

5A.12 GACGACAATACAACCGTTTCATCTAGTGCTGATACTCTAGCATTTGAATATGATTCTTTA

5B.4 GACGACAATACAACCGTTTCATCTAGTGCTGATACTCTAGCATTTGAATATGATTCTTTA

5B.6 GACGACAATACAACCGTTTCATCTAGTGCTGATACTCTAGCATTTGAATATGATTCTTTA

************************************************************

5B.8 GAAAAATTGGACGGGACTGGCTATGGGGATGAAAAAGGGATCCGATTAATTTGCCAACAC

5A.7 GAAAAATTGGACGGGACTGGCTATGGGGATGAAAAAGGGATCCGATTAATTTGCCAACAC

APHIS-S GAAAAATTGGACGGGACTGGCTATGGGGATGAAAAAGGGATCCGATTAATTTGCCAACAC

5A.11 GAAAAATTGGACGGGACTGGCTATGGGGATGAAAAAGGGATCCGATTAATTTGCCAACAC

5A.12 GAAAAATTGGACGGGACTGGCTATGGGGATGAAAAAGGGATCCGATTAATTTGCCAACAC

5B.4 GAAAAATTGGACGGGACTGGCTATGGGGATGAAAAAGGGATCCGATTAATTTGCCAACAC

5B.6 GAAAAATTGGACGGGACTGGCTATGGGGATGAAAAAGGGATCCGATTAATTTGCCAACAC

************************************************************

5B.8 GTGGTAGCAATATGGATGAAACTGTTTCTGGTGCTGACAAGGTCTTGGCTTATCCTGTTG

5A.7 GTGGTAGCAATATGGATGAAACTGTTTCTGGTGCTGACAAGGTCTTGGCTTATCCTGTTG

APHIS-S GTGGTAGCAATATGGATGAAACTGTTTCTGGTGCTGACAAGGTCTTGGCTTATCCTGTTG

5A.11 GTGGTAGCAATATGGATGAAACTGTTTCTGGTGCTGACAAGGTCTTGGCTTATCCTGTTG

5A.12 GTGGTAGCAATATGGATGAAACTGTTTCTGGTGCTGACAAGGTCTTGGCTTATCCTGTTG

5B.4 GTGGTAGCAATATGGATGAAACTGTTTCTGGTGCTGACAAGGTCTTGGCTTATCCTGTTG

5B.6 GTGGTAGCAATATGGATGAAACTGTTTCTGGTGCTGACAAGGTCTTGGCTTATCCTGTTG

************************************************************

5B.8 CTCCAAGTATTGGTGTCCTTGGTACAAATCATTGCCACACTCGGAGTCATGCAGTATGTC

5A.7 CTCCAAGTATTGGTGTCCTTGGTACAAATCATTGCCACACTCGGAGTCATGCAGTATGTC

APHIS-S CTCCAAGTATTGGTGTCCTTGGTACAAATCATTGCCACACTCGGAGTCATGCAGTATGTC

5A.11 CTCCAAGTATTGGTGTCCTTGGTACAAATCATTGCCACACTCGGAGTCATGCAGTATGTC

5A.12 CTCCAAGTATTGGTGTCCTTGGTACAAATCATTGCCACACTCGGAGTCATGCAGTATGTC

5B.4 CTCCAAGTATTGGTGTCCTTGGTACAAATCATTGCCACACTCGGAGTCATGCAGTATGTC

5B.6 CTCCAAGTATTGGTGTCCTTGGTACAAATCATTGCCACACTCGGAGTCATGCAGTATGTC

************************************************************

5B.8 ATCTCTATGACCGAGCATATACAAAGAAGAGAACTTTCATTGGCTGAAGGTTTCGCAGGC

5A.7 ATCTCTATGACCGAGCATATACAAAGAAGAGAACTTTCATTGGCTGAAGGTTTCGCAGGC

APHIS-S ATCTCTATGACCGAGCATATACAAAGAAGAGAACTTTCATTGGCTGAAGGTTTCGCAGGC

5A.11 ATCTCTATGACCGAGCATATACAAAGAAGAGAACTTTCATTGGCTGAAGGTTTCGCAGGC

5A.12 ATCTCTATGACCGAGCATATACAAAGAAGAGAACTTTCATTGGCTGAAGGTTTCGCAGGC

5B.4 ATCTCTATGACCGAGCATATACAAAGAAGAGAACTTTCATTGGCTGAAGGTTTCGCAGGC

5B.6 ATCTCTATGACCGAGCATATACAAAGAAGAGAACTTTCATTGGCTGAAGGTTTCGCAGGC

************************************************************

5B.8 ACAGAAACATTAGTTAGTTTCAAAGGGTTGTCCCCTACATCGACAGGTTCGCTAGCGAAG

5A.7 ACAGAAACATTAGTTAGTTTCAAAGGGTTGTCCCCTACATCGACAGGTTCGCTAGCGAAG

APHIS-S ACAGAAACATTAGTTAGTTTCAAAGGGTTGTCCCCTACATCGACAGGTTCGCTAGCGAAG

5A.11 ACAGAAACATTAGTTAGTTTCAAAGGGTTGTCCCCTACATCGACAGGTTCGCTAGCGAAG

5A.12 ACAGAAACATTAGTTAGTTTCAAAGGGTTGTCCCCTACATCGACAGGTTCGCTAGCGAAG

5B.4 ACAGAAACATTAGTTAGTTTCAAAGGGTTGTCCCCTACATCGACAGGTTCGCTAGCGAAG

5B.6 ACAGAAACATTAGTTAGTTTCAAAGGGTTGTCCCCTACATCGACAGGTTCGCTAGCGAAG

************************************************************

5B.8 GCTGCCTACGAGTCGATATTTGTAACCGCCAATAATCCCACAATGGAAATCACTGTTGTT

5A.7 GCTGCCTACGAGTCGATATTTGTAACCGCCAATAATCCCACAATGGAAATCACTGTTGTT

APHIS-S GCTGCCTACGAGTCGATATTTGTAACCGCCAATAATCCCACAATGGAAATCACTGTTGTT

5A.11 GCTGCCTACGAGTCGATATTTGTAACCGCCAATAATCCCACAATGGAAATCACTGTTGTT

5A.12 GCTGCCTACGAGTCGATATTTGTAACCGCCAATAATCCCACAATGGAAATCACTGTTGTT

5B.4 GCTGCCTACGAGTCGATATTTGTAACCGCCAATAATCCCACAATGGAAATCACTGTTGTT

5B.6 GCTGCCTACGAGTCGATATTTGTAACCGCCAATAATCCCACAATGGAAATCACTGTTGTT

************************************************************

5B.8 GATAATACACCTATAGATGAATATTATTTGGAAAGAACAGATGACGTATCAGCGATGGCG

5A.7 GATAATACACCTATAGATGAATATTATTTGGAAAGAACAGATGACGTATCAGCGATGGCG

APHIS-S GATAATACACCTATAGATGAATATTATTTGGAAAGAACAGATGACGTATCAGCGATGGCG

5A.11 GATAATACACCTATAGATGAATATTATTTGGAAAGA----ATGACGTATCAGCGATGGCG

5A.12 GATAATACACCTATAGATGAATATTATTTGGAAAGAACAGATGACGTATCAGCGATGGCG

5B.4 GATAATACACCTATAGATGAATATTATTTGGAAAGAACAGATGACGTATCAGCGATGGCG

5B.6 GATAATACACCTATAGATGAATATTATTTGGAAAGAACAGATGACGTATCAGCGATGGCG

************************************ ********************

5B.8 GTGCTCCGGCACAGTCTGTTGATCGGCGCGACGTTCGACGACCACTCCGCGACCGCGTGG

5A.7 GTGCTCCGGCACAGTCTGTTGATCGGCGCGACGTTCGACGACCACTCCGCGACCGCGTGG

APHIS-S GTGCTCCGGCACAGTCTGTTGATCGGCGCGACGTTCGACGACCACTCCGCGACCGCGTGG

5A.11 GTGCTCCGGCACAGTCTGTTGATCGGCGCGACGTTCGACGACCACTCCGCGACCGCGTGG

5A.12 GTGCTCCGGCACAGTCTGTTGATCGGCGCGACGTTCGACGACCACTCCGCGACCGCGTGG

5B.4 GTGCTCCGGCACAGTCTGTTGATCGGCGCGACGTTCGACGACCACTCCGCGACCGCGTGG

5B.6 GTGCTCCGGCACAGTCTGTTGATCGGCGCGACGTTCGACGACCACTCCGCGACCGCGTGG

************************************************************

5B.8 TTCAGCAACTTCGGTTACCACGACGTGGCCATGTCACTGGCTGCTGTGCACGCCGCCTTG

5A.7 TTCAGCAACTTCGGTTACCACGACGTGGCCATGTCACTGGCTGCTGTGCACGCCGCCTTG

APHIS-S TTCAGCAACTTCGGTTACCACGACGTGGCCATGTCACTGGCTGCTGTGCACGCCGCCTTG

5A.11 TTCAGCAACTTCGGTTACCACGACGTGGCCATGTCACTGGCTGCTGTGCACGCCGCCTTG

5A.12 TTCAGCAACTTCGGTTACCACGACGTGGCCATGTCACTGGCTGCTGTGCACGCCGCCTTG

5B.4 TTCAGCAACTTCGGTTACCACGACGTGGCCATGTCACTGGCTGCTGTGCACGCCGCCTTG

5B.6 TTCAGCAACTTCGGTTACCACGACGTGGCCATGTCACTGGCTGCTGTGCACGCCGCCTTG

************************************************************

5B.8 CTCAGAGCTGTCAATCCTGCAGCCAACTTGACTGTTTACAACCACCCACTTGAGGCCAAT

5A.7 CTCAGAGCTGTCAATCCTGCAGCCAACTTGACTGTTTACAACCACCCACTTGAGGCCAAT

APHIS-S CTCAGAGCTGTCAATCCTGCAGCCAACTTGACTGTTTACAACCACCCACTTGAGGCCAAT

5A.11 CTCAGAGCTGTCAATCCTGCAGCCAACTTGACTGTTTACAACCACCCACTTGAGGCCAAT

5A.12 CTCAGAGCTGTCAATCCTGCAGCCAACTTGACTGTTTACAACCACCCACTTGAGGCCAAT

5B.4 CTCAGAGCTGTCAATCCTGCAGCCAACTTGACTGTTTACAACCACCCACTTGAGGCCAAT

5B.6 CTCAGAGCTGTCAATCCTGCAGCCAACTTGACTGTTTACAACCACCCACTTGAGGCCAAT

************************************************************

5B.8 TATGTCAACCAGAACGACATGCAAACAATGGTAGCGTTCCTCTCGATGCAACTTGCGTCG

5A.7 TATGTCAACCAG------------------------------------------------

APHIS-S TATGTCAACCAGAACGACATGCAAACAATGGTAGCGTTCCTCTCGATGCAACTTGCGTCG

5A.11 TATGTCAACCAGAACGACATGCAAACAATGGTAGCGTTCCTCTCGATGCAACTTGCGTCG

5A.12 TATGTCAACCAGAACGACATGCAAACAATGGTAGCGTTCCTCTCGATGCAACTTGCGTCG

5B.4 TATGTCAACCAGAACGACATGCAAACAATGGTAGCGTTCCTCTCGATGCAACTTGCGTCG

5B.6 TATGTCAACCAGAACGACATGCAAACAATGGTAGCGTTCCTCTCGATGCAACTTGCGTCG

************

5B.8 GGCATCGGCAGCAGTCTGTCAATTGTCAGTGCTGTGTTCATCATGTTCTATATCAAGGAG

5A.7 ------------------------------------------------------------

APHIS-S GGCATCGGCAGCAGTCTGTCAATTGTCAGTGCTGTGTTCATCATGTTCTATATCAAGGAG

5A.11 GGCATCGGCAGCAGTCTGTCAATTGTCAGTGCTGTGTTCATCATGTTCTATATCAAGG--

5A.12 GGCATCGGCAGCAGTCTGTCAATTGTCAGTGCTGTGTTCATCATGTTCTATATCAAGG--

5B.4 GGCATCGGCAGCAGTCTGTCAATTGTCAGTGCTGTGTTCATCATGTTCTATATCAAGGAG

5B.6 GGCATCGGCAGCAGTCTGTCAATTGTCAGTGCTGTGTTCATCATGTTCTATATCAAGGAG

5B.8 CGAGTATCTCGCGCCAAGCTGCTGCAGAAGGCGGCAGGCATCCAGCCGTTAGTGATGTGG

5A.7 ------------------------------------------------------------

APHIS-S CGAGTATCTCGCGCCAAGCTGCTGCAGAAGGCGGCAGGCATCCAGCCGTTAGTGATGTGG

5A.11 ------------------------------------------------------------

5A.12 ------------------------------------------------------------

5B.4 CGAGTATCTCGCGCCAAGCTGCTGCAGAAGGCGGCAGGCATCCAGCCGTTAGTGATGTGG

5B.6 CGAGTATCTCGCGCCAAGCTGCTGCAGAAGGCGGCAGGCATCCAGCCGTTAGTGATGTGG

5B.8 CTCAGCGCCGCCGTGTTCGACTGGATCTGGTTCTGCGTCATCGCCGTCGGCATCGTTATC

5A.7 ------------------------------------------------------------

APHIS-S CTCAGCGCCGCCGTGTTCGACTGGATCTGGTTCTGCGTCATCGCCGTCGGCATCGTTATC

5A.11 ------------------------------------------------------------

5A.12 ------------------------------------------------------------

5B.4 CTCAGCGCCGCCGTGTTCGACTGGATCTGGTTCTGCGTCATCGCCGTCGGCATCGTTATC

5B.6 CTCAGCGCCGCCGTGTTCGACTGGATCTGGTTCTGCGTCATCGCCGTCGGCATCGTTATC

5B.8 GCCTGCGCCGCTTTTAACGTCATTGGGCTCTCTTCTGTCGATGAACTGGGTCGGATGTAC

5A.7 -------------------------------------------------GTCGGATGTAC

APHIS-S GCCTGCGCCGCTTTTAACGTCATTGGGCTCTCTTCTGTCGATGAACTGGGTCGGATGTAC

5A.11 --------------------------------------------------TCGGATGTAC

5A.12 --------------------------------------------------TCGGATGTAC

5B.4 GCCTGCGCCGCTTTTAACGTCATTGGGCTCTCTTCTGTCGATGAACTGGGTCGGATGTAC

5B.6 GCCTGCGCCGCTTTTAACGTCATTGGGCTCTCTTCTGTCGATGAACTGGGTCGGATGTAC

**********

5B.8 TTGTGCATCATAGTGTATGGCGCCGCCAGTCTACCGATAGGCTACGTGTTCTCCTATTTC

5A.7 TTGTGCATCATAGTGTATGGCGCCGCCAGTCTACCGATAGGCTACGTGTTCTCCTATTTC

APHIS-S TTGTGCATCATAGTGTATGGCGCCGCCAGTCTACCGATAGGCTACGTGTTCTCCTATTTC

5A.11 TTGTGCATCATAGTGTATGGCGCCGCCAGTCTACCGATAGGCTACGTGTTCTCCTATTTC

5A.12 TTGTGCATCATAGTGTATGGCGCCGCCAGTCTACCGATAGGCTACGTGTTCTCCTATTTC

5B.4 TTGTGCATCATAGTGTATGGCGCCGCCAGTCTACCGATAGGCTACGTGTTCTCCTATTTC

5B.6 TTGTGCATCATAGTGTATGGCGCCGCCAGTCTACCGATAGGCTACGTGTTCTCCTATTTC

************************************************************

5B.8 TTCAAAGGCCCTGCCGTCGGTTTTGTCACCATGTTCTTTATCAACATTCTCTTTGGTATG

5A.7 TTCAAAGGCCCTGCCGTCGGTTTTGTCACCATGTTCTTTATCAACATTCTCTTTGGTATG

APHIS-S TTCAAAGGCCCTGCCGTCGGTTTTGTCACCATGTTCTTTATCAACATTCTCTTTGGTATG

5A.11 TTCAAAGGCCCTGCCGTCGGTTTTGTCACCATGTTCTTTATCAACATTCTCTTTGGTATG

5A.12 TTCAAAGGCCCTGCCGTCGGTTTTGTCACCATGTTCTTTATCAACATTCTCTTTGGTATG

5B.4 TTCAAAGGCCCTGCCGTCGGTTTTGTCACCATGTTCTTTATCAACATTCTCTTTGGTATG

5B.6 TTCAAAGGCCCTGCCGTCGGTTTTGTCACCATGTTCTTTATCAACATTCTCTTTGGTATG

************************************************************

5B.8 ATGGGGGCGCAGATTGTGGAGGCCTTGTTGTCACCGCAGCTTGATACTGAAAATGTCGCT

5A.7 ATGGGGGCGCAGATTGTGGAGGCCTTGTTGTCACCGCAGCTTGATACTGAAAATGTCGCT

APHIS-S ATGGGGGCGCAGATTGTGGAGGCCTTGTTGTCACCGCAGCTTGATACTGAAAATGTCGCT

5A.11 ATGGGGGCGCAGATTGTGGAGGCCTTGTTGTCACCGCAGCTTGATACTGAAAATGTCGCT

5A.12 ATGGGGGCGCAGATTGTGGAGGCCTTGTTGTCACCGCAGCTTGATACTGAAAATGTCGCT

5B.4 ATGGGGGCGCAGATTGTGGAGGCCTTGTTGTCACCGCAGCTTGATACTGAAAATGTCGCT

5B.6 ATGGGGGCGCAGATTGTGGAGGCCTTGTTGTCACCGCAGCTTGATACTGAAAATGTCGCT

************************************************************

5B.8 AATATACTTGACTCCATCTTGCAATTCTTCCCACTCTATAGTCTTGTCACATCTGCCAGA

5A.7 AATATACTTGACTCCATCTTGCAATTCTTCCCACTCTATAGTCTTGTCACATCTGCCAGA

APHIS-S AATATACTTGACTCCATCTTGCAATTCTTCCCACTCTATAGTCTTGTCACATCTGCCAGA

5A.11 AATATACTTGACTCCATCTTGCAATTCTTCCCACTCTATAGTCTTGTCACATCTGCCAGA

5A.12 AATATACTTGACTCCATCTTGCAATTCTTCCCACTCTATAGTCTTGTCACATCTGCCAGA

5B.4 AATATACTTGACTCCATCTTGCAATTCTTCCCACTCTATAGTCTTGTCACATCTGCCAGA

5B.6 AATATACTTGACTCCATCTTGCAATTCTTCCCACTCTATAGTCTTGTCACATCTGCCAGA

************************************************************

5B.8 CTGTTGAATCAGGTGGGACTGCTGGAGTGGTCGTGCCTGCAGAACTGCGAGTACCTGTCC

5A.7 CTGTTGAATCAGGTGGGACTGCTGGAGTGGTCGTGCCTGCAGAACTGCGAGTACCTGTCC

APHIS-S CTGTTGAATCAGGTGGGACTGCTGGAGTGGTCGTGCCTGCAGAACTGCGAGTACCTGTCC

5A.11 CTGTTGAATCAGGTGGGACTGCTGGAGTGGTCGTGCCTGCAGAACTGCGAGTACCTGTCC

5A.12 CTGTTGAATCAGGTGGGACTGCTGGAGTGGTCGTGCCTGCAGAACTGCGAGTACCTGTCC

5B.4 CTGTTGAATCAGGTGGGACTGCTGGAGTGGTCGTGCCTGCAGAACTGCGAGTACCTGTCC

5B.6 CTGTTGAATCAGGTGGGACTGCTGGAGTGGTCGTGCCTGCAGAACTGCGAGTACCTGTCC

************************************************************

5B.8 GCAGTGATGCCCAACTTGACCGAATGCTCCATGGACGTTATGTGCCAGACGTTCTCACAA

5A.7 GCAGTGATGCCCAACTTGACCGAATGCTCCATGGACGTTATGTGCCAGACGTTCTCACAA

APHIS-S GCAGTGATGCCCAACTTGACCGAATGCTCCATGGACGTTATGTGCCAGACGTTCTCACAA

5A.11 GCAGTGATGCCCAACTTGACCGAATGCTCCATGGACGTTATGTGCCAGACGTTCTCACAA

5A.12 GCAGTGATGCCCAACTTGACCGAATGCTCCATGGACGTTATGTGCCAGACGTTCTCACAA

5B.4 GCAGTGATGCCCAACTTGACCGAATGCTCCATGGACGTTATGTGCCAGACGTTCTCACAA

5B.6 GCAGTGATGCCCAACTTGACCGAATGCTCCATGGACGTTATGTGCCAGACGTTCTCACAA

************************************************************

5B.8 TGTTGCATCCCAGACGATCCTTGGTTCATGTGGGATCACCCTGGAGTACTCCGCTACATA

5A.7 TGTTGCATCCCAGACGATCCTTGGTTCATGTGGGATCACCCTGGAGTACTCCGCTACATA

APHIS-S TGTTGCATCCCAGACGATCCTTGGTTCATGTGGGATCACCCTGGAGTACTCCGCTACATA

5A.11 TGTTGCATCCCAGACGATCCTTGGTTCATGTGGGATCACCCTGGAGTACTCCGCTACATA

5A.12 TGTTGCATCCCAGACGATCCTTGGTTCATGTGGGATCACCCTGGAGTACTCCGCTACATA

5B.4 TGTTGCATCCCAGACGATCCTTGGTTCATGTGGGATCACCCTGGAGTACTCCGCTACATA

5B.6 TGTTGCATCCCAGACGATCCTTGGTTCATGTGGGATCACCCTGGAGTACTCCGCTACATA

************************************************************

5B.8 GTATGCATGATCGTCAGTGGAGTTGTCATGTGGTTCGTACTCTTGATCGCCGAGTATCGA

5A.7 GTATGCATGATCGTCAGTGGAGTTGTCATGTGGTTCGTACTCTTGATCGCCGAGTATCGA

APHIS-S GTATGCATGATCGTCAGTGGAGTTGTCATGTGGTTCGTACTCTTGATCGCCGAGTATCGA

5A.11 GTATGCATGATCGTCAGTGGAGTTGTCATGTGGTTCGTACTCTTGATCGCCGAGTATCGA

5A.12 GTATGCATGATCGTCAGTGGAGTTGTCATGTGGTTCGTACTCTTGATCGCCGAGTATCGA

5B.4 GTATGCATGATCGTCAGTGGAGTTGTCATGTGGTTCGTACTCTTGATCGCCGAGTATCGA

5B.6 GTATGCATGATCGTCAGTGGAGTTGTCATGTGGTTCGTACTCTTGATCGCCGAGTATCGA

************************************************************

5B.8 TTGTTCCAGAAGGTGATCTACCGGGAAAAGAAAGCTCCTCCAGTTGATGAGAGCGCACTG

5A.7 TTGTTCCAGAAGGTGATCTACCGGGAAAAGAAAGCTCCTCCAGTTGATGAGAGCGCACTG

APHIS-S TTGTTCCAGAAGGTGATCTACCGGGAAAAGAAAGCTCCTCCAGTTGATGAGAGCGCACTG

5A.11 TTGTTCCAGAAGGTGATCTACCGGGAAAAGAAAGCTCCTCCAGTTGATGAGAGCGCACTG

5A.12 TTGTTCCAGAAGGTGATCTACCGGGAAAAGAAAGCTCCTCCAGTTGATGAGAGCGCACTG

5B.4 TTGTTCCAGAAGGTGATCTACCGGGAAAAGAAAGCTCCTCCAGTTGATGAGAGCGCACTG

5B.6 TTGTTCCAGAAGGTGATCTACCGGGAAAAGAAAGCTCCTCCAGTTGATGAGAGCGCACTG

************************************************************

5B.8 GACAATGACGTGGCGGACGAGGCCAGACACGTGGCGCGAGTTGGAGCAGGAGCAATCCTC

5A.7 GACAATGACGTGGCGGACGAGGCCAGACACGTGGCGCGAGTTGGAGCAGGAGCAATCCTC

APHIS-S GACAATGACGTGGCGGACGAGGCCAGACACGTGGCGCGAGTTGGAGCAGGAGCAATCCTC

5A.11 GACAATGACGTGGCGGACGAGGCCAGACACGTGGCGCGAGTTGGAGCAGGAGCAATCCTC

5A.12 GACAATGACGTGGCGGACGAGGCCAGACACGTGGCGCGAGTTGGAGCAGGAGCAATCCTC

5B.4 GACAATGACGTGGCGGACGAGGCCAGACACGTGGCGCGAGTTGGAGCAGGAGCAATCCTC

5B.6 GACAATGACGTGGCGGACGAGGCCAGACACGTGGCGCGAGTTGGAGCAGGAGCAATCCTC

************************************************************

5B.8 GGGCAGCACAGCCTAGTAGCAAATGGCCTCACCAAGTATTATGGGAAACACCTTGCAGTC

5A.7 GGGCAGCACAGCCTAGTAGCAAATGGCCTCACCAAGTATTATGGGAAACACCTTGCAGTC

APHIS-S GGGCAGCACAGCCTAGTAGCAAATGGCCTCACCAAGTATTATGGGAAACACCTTGCAGTC

5A.11 GGGCAGCACAGCCTAGTAGCAAATGGCCTCACCAAGTATTATGGGAAACACCTTGCAGTC

5A.12 GGGCAGCACAGCCTAGTAGCAAATGGCCTCACCAAGTATTATGGGAAACACCTTGCAGTC

5B.4 GGGCAGCACAGCCTAGTAGCAAATGGCCTCACCAAGTATTATGGGAAACACCTTGCAGTC

5B.6 GGGCAGCACAGCCTAGTAGCAAATGGCCTCACCAAGTATTATGGGAAACACCTTGCAGTC

************************************************************

5B.8 AATCAAGTGTCATTCACCGTGGGCGACACGGAATGCTTTGGTCTTCTGGGTGTGAACGGC

5A.7 AATCAAGTGTCATTCACCGTGGGCGACACGGAATGCTTTGGTCTTCTGGGTGTGAACGGC

APHIS-S AATCAAGTGTCATTCACCGTGGGCGACACGGAATGCTTTGGTCTTCTGGGTGTGAACGGC

5A.11 AATCAAGTGTCATTCACCGTGGGCGACACGGAATGCTTTGGTCTTCTGGGTGTGAACGGC

5A.12 AATCAAGTGTCATTCACCGTGGGCGACACGGAATGCTTTGGTCTTCTGGGTGTGAACGGC

5B.4 AATCAAGTGTCATTCACCGTGGGCGACACGGAATGCTTTGGTCTTCTGGGTGTGAACGGC

5B.6 AATCAAGTGTCATTCACCGTGGGCGACACGGAATGCTTTGGTCTTCTGGGTGTGAACGGC

************************************************************

5B.8 GCCGGTAAGACGACCACCTTCAAGATGTTGATGGGAGATGAGACCGTCTCCAGCGGAGAT

5A.7 GCCGGTAAGACGACCACCTTCAAGATGTTGATGGGAGATGAGACCGTCTCCAGCGGAGAT

APHIS-S GCCGGTAAGACGACCACCTTCAAGATGTTGATGGGAGATGAGACCGTCTCCAGCGGAGAT

5A.11 GCCGGTAAGACGACCACCTTCAAGATGTTGATGGGAGATGAGACCGTCTCCAGCGGAGAT

5A.12 GCCGGTAAGACGACCACCTTCAAGATGTTGATGGGAGATGAGACCGTCTCCAGCGGAGAT

5B.4 GCCGGTAAGACGACCACCTTCAAGATGTTGATGGGAGATGAGACCGTCTCCAGCGGAGAT

5B.6 GCCGGTAAGACGACCACCTTCAAGATGTTGATGGGAGATGAGACCGTCTCCAGCGGAGAT

************************************************************

5B.8 GCCTTCGTGAGTGGCCATTCTGTCAAGACTAATATCACTCAAGTTTACAAAAATATTGGT

5A.7 GCCTTCGTGAGTGGCCATTCTGTCAAGACTAATATCACTCAAGTTTACAAAAATATTGGT

APHIS-S GCCTTCGTGAGTGGCCATTCTGTCAAGACTAATATCACTCAAGTTTACAAAAATATTGGT

5A.11 GCCTTCGTGAGTGGCCATTCTGTCAAGACTAATATCACTCAAGTTTACAAAAATATTGGT

5A.12 GCCTTCGTGAGTGGCCATTCTGTCAAGACTAATATCACTCAAGTTTACAAAAATATTGGT

5B.4 GCCTTCGTGAGTGGCCATTCTGTCAAGACTAATATCACTCAAGTTTACAAAAATATTGGT

5B.6 GCCTTCGTGAGTGGCCATTCTGTCAAGACTAATATCACTCAAGTTTACAAAAATATTGGT

************************************************************

5B.8 TACTGTCCGCAATTCGAAGCGACATTCGGCGAGCTGACGGGACGCGAGACACTACGGCTG

5A.7 TACTGTCCGCAATTCGAAGCGACATTCGGCGAGCTGACGGGACGCGAGACACTACGGCTG

APHIS-S TACTGTCCGCAATTCGAAGCGACATTCGGCGAGCTGACGGGACGCGAGACACTACGGCTG

5A.11 TACTGTCCGCAATTCGAAGCGACATTCGGCGAGCTGACGGGACGCGAGACACTACGGCTG

5A.12 TACTGTCCGCAATTCGAAGCGACATTCGGCGAGCTGACGGGACGCGAGACACTACGGCTG

5B.4 TACTGTCCGCAATTCGAAGCGACATTCGGCGAGCTGACGGGACGCGAGACACTACGGCTG

5B.6 TACTGTCCGCAATTCGAAGCGACATTCGGCGAGCTGACGGGACGCGAGACACTACGGCTG

************************************************************

5B.8 TTCTCGGCGCTGCGAGGGTTGCCAGTGCGAGGCGCCACGCTCCACGCGGAGGCCTTAGCA

5A.7 TTCTCGGCGCTGCGAGGGTTGCCAGTGCGAGGCGCCACGCTCCACGCGGAGGCCTTAGCA

APHIS-S TTCTCGGCGCTGCGAGGGTTGCCAGTGCGAGGCGCCACGCTCCACGCGGAGGCCTTAGCA

5A.11 TTCTCGGCGCTGCGAGGGTTGCCAGTGCGAGGCGCCACGCTCCACGCGGAGGCCTTAGCA

5A.12 TTCTCGGCGCTGCGAGGGTTGCCAGTGCGAGGCGCCACGCTCCACGCGGAGGCCTTAGCA

5B.4 TTCTCGGCGCTGCGAGGGTTGCCAGTGCGAGGCGCCACGCTCCACGCGGAGGCCTTAGCA

5B.6 TTCTCGGCGCTGCGAGGGTTGCCAGTGCGAGGCGCCACGCTCCACGCGGAGGCCTTAGCA

************************************************************

5B.8 CATGCTCTTGGTTTCTATAAGCATCTTGATAAAAGGGTGGACCACTACTCTGGTGGCAAC

5A.7 CATGCTCTTGGTTTCTATAAGCATCTTGATAAAAGGGTGGACCACTACTCTGGTGGCAAC

APHIS-S CATGCTCTTGGTTTCTATAAGCATCTTGATAAAAGGGTGGACCACTACTCTGGTGGCAAC

5A.11 CATGCTCTTGGTTTCTATAAGCATCTTGATAAAAGGGTGGACCACTACTCTGGTGGCAAC

5A.12 CATGCTCTTGGTTTCTATAAGCATCTTGATAAAAGGGTGGACCACTACTCTGGTGGCAAC

5B.4 CATGCTCTTGGTTTCTATAAGCATCTTGATAAAAGGGTGGACCACTACTCTGGTGGCAAC

5B.6 CATGCTCTTGGTTTCTATAAGCATCTTGATAAAAGGGTGGACCACTACTCTGGTGGCAAC

************************************************************

5B.8 AAGCGCAAGTTGAGCACGGCTGTGGCGTTGCTGGGGCGCACGCGGCTTATATTCGTCGAC

5A.7 AAGCGCAAGTTGAGCACGGCTGTGGCGTTGCTGGGGCGCACGCGGCTTATATTCGTCGAC

APHIS-S AAGCGCAAGTTGAGCACGGCTGTGGCGTTGCTGGGGCGCACGCGGCTTATATTCGTCGAC

5A.11 AAGCGCAAGTTGAGCACGGCTGTGGCGTTGCTGGGGCGCACGCGGCTTATATTCGTCGAC

5A.12 AAGCGCAAGTTGAGCACGGCTGTGGCGTTGCTGGGGCGCACGCGGCTTATATTCGTCGAC

5B.4 AAGCGCAAGTTGAGCACGGCTGTGGCGTTGCTGGGGCGCACGCGGCTTATATTCGTCGAC

5B.6 AAGCGCAAGTTGAGCACGGCTGTGGCGTTGCTGGGGCGCACGCGGCTTATATTCGTCGAC

************************************************************

5B.8 GAACCCACTACTGGAGTCGATCCTGCTGCTAAGAGACAGATGTGGAACGCGGTTCGAGAA

5A.7 GAACCCACTACTGGAGTCGATCCTGCTGCTAAGAGACAGATGTGGAACGCGGTTCGAGAA

APHIS-S GAACCCACTACTGGAGTCGATCCTGCTGCTAAGAGACAGATGTGGAACGCGGTTCGAGAA

5A.11 GAACCCACTACTGGAGTCGATCCTGCTGCTAAGAGACAGATGTGGAACGCGGTTCGAGAA

5A.12 GAACCCACTACTGGAGTCGATCCTGCTGCTAAGAGACAGATGTGGAACGCGGTTCGAGAA

5B.4 GAACCCACTACTGGAGTCGATCCTGCTGCTAAGAGACAGATGTGGAACGCGGTTCGAGAA

5B.6 GAACCCACTACTGGAGTCGATCCTGCTGCTAAGAGACAGATGTGGAACGCGGTTCGAGAA

************************************************************

5B.8 GCTCGCCGGTCGGGTCGTGGTGTGGTGCTGACATCACACAGCATGGAGGAGTGTGAGGCT

5A.7 GCTCGCCGGTCGGGTCGTGGTGTGGTGCTGACATCACACAGCATGGAGGAGTGTGAGGCT

APHIS-S GCTCGCCGGTCGGGTCGTGGTGTGGTGCTGACATCACACAGCATGGAGGAGTGTGAGGCT

5A.11 GCTCGCCGGTCGGGTCGTGGTGTGGTGCTGACATCACACAGCATGGAGGAGTGTGAGGCT

5A.12 GCTCGCCGGTCGGGTCGTGGTGTGGTGCTGACATCACACAGCATGGAGGAGTGTGAGGCT

5B.4 GCTCGCCGGTCGGGTCGTGGTGTGGTGCTGACATCACACAGCATGGAGGAGTGTGAGGCT

5B.6 GCTCGCCGGTCGGGTCGTGGTGTGGTGCTGACATCACACAGCATGGAGGAGTGTGAGGCT

************************************************************

5B.8 CTGTGCTCGCGGCTCACAATCATGGTCAACGGACAGTTCCAGTGCCTCGGCACGCCGCAA

5A.7 CTGTGCTCGCGGCTCACAATCATGGTCAACGGACAGTTCCAGTGCCTCGGCACGCCGCAA

APHIS-S CTGTGCTCGCGGCTCACAATCATGGTCAACGGACAGTTCCAGTGCCTCGGCACGCCGCAA

5A.11 CTGTGCTCGCGGCTCACAATCATGGTCAACGGACAGTTCCAGTGCCTCGGCACGCCGCAA

5A.12 CTGTGCTCGCGGCTCACAATCATGGTCAACGGACAGTTCCAGTGCCTCGGCACGCCGCAA

5B.4 CTGTGCTCGCGGCTCACAATCATGGTCAACGGACAGTTCCAGTGCCTCGGCACGCCGCAA

5B.6 CTGTGCTCGCGGCTCACAATCATGGTCAACGGACAGTTCCAGTGCCTCGGCACGCCGCAA

************************************************************

5B.8 CATTTAAAGAATAAGTTCTCTGAAGGTTTCACATTGACAATTAAAATTAAAGTGGACGAC

5A.7 CATTTAAAGAATAAGTTCTCTGAAGGTTTCACATTGACAATTAAAATTAAAGTGGACGAC

APHIS-S CATTTAAAGAATAAGTTCTCTGAAGGTTTCACATTGACAATTAAAATTAAAGTGGACGAC

5A.11 CATTTAAAGAATAAGTTCTCTGAAGGTTTCACATTGACAATTAAAATTAAAGTGGACGAC

5A.12 CATTTAAAGAATAAGTTCTCTGAAGGTTTCACATTGACAATTAAAATTAAAGTGGACGAC

5B.4 CATTTAAAGAATAAGTTCTCTGAAGGTTTCACATTGACAATTAAAATTAAAGTGGACGAC

5B.6 CATTTAAAGAATAAGTTCTCTGAAGGTTTCACATTGACAATTAAAATTAAAGTGGACGAC

************************************************************

5B.8 GAGACGAAGACTGTACGGCCTGAAGTCTGCGATGCTGTGAAGCATTACGTCAGTACCAAC

5A.7 GAGACGAAGACTGTACGGCCTGAAGTCTGCGATGCTGTGAAGCATTACGTCAGTACCAAC

APHIS-S GAGACGAAGACTGTACGGCCTGAAGTCTGCGATGCTGTGAAGCATTACGTCAGTACCAAC

5A.11 GAGACGAAGACTGTACGGCCTGAAGTCTGCGATGCTGTGAAGCATTACGTCAGTACCAAC

5A.12 GAGACGAAGACTGTACGGCCTGAAGTCTGCGATGCTGTGAAGCATTACGTCAGTACCAAC

5B.4 GAGACGAAGACTGTACGGCCTGAAGTCTGCGATGCTGTGAAGCATTACGTCAGTACCAAC

5B.6 GAGACGAAGACTGTACGGCCTGAAGTCTGCGATGCTGTGAAGCATTACGTCAGTACCAAC

************************************************************

5B.8 TTCAGAGAGCCGAAGATTATGGAGGAGTACCAGGGTCTGTTAACATACTATTTGCCAGAC

5A.7 TTCAGAGAGCCGAAGATTATGGAGGAGTACCAGGGTCTGTTAACATACTATTTGCCAGAC

APHIS-S TTCAGAGAGCCGAAGATTATGGAGGAGTACCAGGGTCTGTTAACATACTATTTGCCAGAC

5A.11 TTCAGAGAGCCGAAGATTATGGAGGAGTACCAGGGTCTGTTAACATACTATTTGCCAGAC

5A.12 TTCAGAGAGCCGAAGATTATGGAGGAGTACCAGGGTCTGTTAACATACTATTTGCCAGAC

5B.4 TTCAGAGAGCCGAAGATTATGGAGGAGTACCAGGGTCTGTTAACATACTATTTGCCAGAC

5B.6 TTCAGAGAGCCGAAGATTATGGAGGAGTACCAGGGTCTGTTAACATACTATTTGCCAGAC

************************************************************

5B.8 AAGTCGGTGGCGTGGTCCAGAATGTTCGGCATAATGGAGGCGGCCAAACGCGACCTCCCC

5A.7 AAGTCGGTGGCGTGGTCCAGAATGTTCGGCATAATGGAGGCGGCCAAACGCGACCTCCCC

APHIS-S AAGTCGGTGGCGTGGTCCAGAATGTTCGGCATAATGGAGGCGGCCAAACGCGACCTCCCC

5A.11 AAGTCGGTGGCGTGGTCCAGAATGTTCGGCATAATGGAGGCGGCCAAACGCGACCTCCCC

5A.12 AAGTCGGTGGCGTGGTCCAGAATGTTCGGCATAATGGAGGCGGCCAAACGCGACCTCCCC

5B.4 AAGTCGGTGGCGTGGTCCAGAATGTTCGGCATAATGGAGGCGGCCAAACGCGACCTCCCC

5B.6 AAGTCGGTGGCGTGGTCCAGAATGTTCGGCATAATGGAGGCGGCCAAACGCGACCTCCCC

************************************************************

5B.8 GTCGAAGACTACAGCATATCACAAACTACCCTCGAG------------------------

5A.7 GTCGAAGACTACAGCATATCACAAACTACCCTCGAG------------------------

APHIS-S GTCGAAGACTACAGCATATCACAAACTACCCTCGAGCAGATATTCCTACAGTTCACAAAG

5A.11 GTCGAAGACTACAGCATATCACAAACTACCCTCGAG------------------------

5A.12 GTCGAAGACTACAGCATATCACAAACTACCCTCGAG------------------------

5B.4 GTCGAAGACTACAGCATATCACAAACTACCCTCGAG------------------------

5B.6 GTCGAAGACTACAGCATATCACAAACTACCCTCGAG------------------------

************************************

5B.8 ---------------------------

5A.7 ---------------------------

APHIS-S TATCAACATGAAGCACAACAGACATAA

5A.11 ---------------------------

5A.12 ---------------------------

5B.4 ---------------------------

5B.6 ---------------------------

**C**

9A.10 ATGCGGGCGCGTGGAGAGCGGAAGGAGGCGGGCTCATGGGTGAAGTTTAGGCTGTTGATG

9A.8 ATGCGGGCGCGTGGAGAGCGGAAGGAGGCGGGCTCATGGGTGAAGTTTAGGCTGTTGATG

9A.13 ATGCGGGCGCGTGGAGAGCGGAAGGAGGCGGGCTCATGGGTGAAGTTTAGGCTGTTGATG

9B.12 ATGCGGGCGCGTGGAGAGCGGAAGGAGGCGGGCTCATGGGTGAAGTTTAGGCTGTTGATG

9A.9 ATGCGGGCGCGTGGAGAGCGGAAGGAGGCGGGCTCATGGGTGAAGTTTAGGCTGTTGATG

9B.16 ATGCGGGCGCGTGGAGAGCGGAAGGAGGCGGGCTCATGGGTGAAGTTTAGGCTGTTGATG

9B.14 ATGCGGGCGCGTGGAGAGCGGAAGGAGGCGGGCTCATGGGTGAAGTTTAGGCTGTTGATG

APHIS-S ATGCGGGCGCGTGGAGAGCGGAAGGAGGCGGGCTCATGGGTGAAGTTTAGGCTGTTGATG

************************************************************

9A.10 TGGAAGAACTTCGTGCAGCAGTTGAGGCACCCAGTGCAGACGGCGGCTGAGCTGCTGCTA

9A.8 TGGAAGAACTTCGTGCAGCAGTTGAGGCACCCAGTGCAGACGGCGGCTGAGCTGCTGCTA

9A.13 TGGAAGAACTTCGTGCAGCAGTTGAGGCACCCAGTGCAGACGGCGGCTGAGCTGCTGCTA

9B.12 TGGAAGAACTTCGTGCAGCAGTTGAGGCACCCAGTGCAGACGGCGGCTGAGCTGCTGCTA

9A.9 TGGAAGAACTTCGTGCAGCAGTTGAGGCACCCAGTGCAGACGGCGGCTGAGCTGCTGCTA

9B.16 TGGAAGAACTTCGTGCAGCAGTTGAGGCACCCAGTGCAGACGGCGGCTGAGCTGCTGCTA

9B.14 TGGAAGAACTTCGTGCAGCAGTTGAGGCACCCAGTGCAGACGGCGGCTGAGCTGCTGCTA

APHIS-S TGGAAGAACTTCGTGCAGCAGTTGAGGCACCCAGTGCAGACGGCGGCTGAGCTGCTGCTA

************************************************************

9A.10 CCAGTCCTAACCATGAGCCTGGTCCTGGTGCTACGGTCACAGATCGACCCCGAAGTCTTG

9A.8 CCAGTCCTAACCATGAGCCTGGTCCTGGTGCTACGGTCACAGATCGACCCCGAAGTCTTG

9A.13 CCAGTCCTAACCATGAGCCTGGTCCTGGTGCTACGGTCACAGATCGACCCCGAAGTCTTG

9B.12 CCAGTCCTAACCATGAGCCTGGTCCTGGTGCTACGGTCACAGATCGACCCCGAAGTCTTG

9A.9 CCAGTCCTAACCATGAGCCTGGTCCTGGTGCTACGGTCACAGATCGACCCCGAAGTCTTG

9B.16 CCAGTCCTAACCATGAGCCTGGTCCTGGTGCTACGGTCACAGATCGACCCCGAAGTCTTG

9B.14 CCAGTCCTAACCATGAGCCTGGTCCTGGTGCTACGGTCACAGATCGACCCCGAAGTCTTG

APHIS-S CCAGTCCTAACCATGAGCCTGGTCCTGGTGCTACGGTCACAGATCGACCCCGAAGTCTTG

************************************************************

9A.10 GAAACCAGAACCTACCCGCCAATACCAGCCCACACTTTAAACTATTCCGTGACTGTTTTG

9A.8 GAAACCAGAACCTACCCGCCAATACCAGCCCACACTTTAAACTATTCCGTGACTGTTTTG

9A.13 GAAACCAGAACCTACCCGCCAATACCAGCCCACACTTTAAACTATTCCGTGACTGTTTTG

9B.12 GAAACCAGAACCTACCCGCCAATACCAGCCCACACTTTAAACTATTCCGTGACTGTTTTG

9A.9 GAAACCAGAACCTACCCGCCAATACCAGCCCACACTTTAAACTATTCCGTGACTGTTTTG

9B.16 GAAACCAGAACCTACCCGCCAATACCAGCCCACACTTTAAACTATTCCGTGACTGTTTTG

9B.14 GAAACCAGAACCTACCCGCCAATACCAGCCCACACTTTAAACTATTCCGTGACTGTTTTG

APHIS-S GAAACCAGAACCTACCCGCCAATACCAGCCCACACTTTAAACTATTCCGTGACTGTTTTG

************************************************************

9A.10 GGCGGAATGAATTTAACAAGAATGTCCATGGCATTCTCACCCGAGAATGCCGTG------

9A.8 GGCGGAATGAATTTAACAAGAATGTCCATGGCATTCTCACCCGAGAATGCCGTG------

9A.13 GGCGGAATGAATTTAACAAGAATGTCCATGGCATTCTCACCCGAGAATGCCGTG------

9B.12 GGCGGAATGAATTTAACAAGAATGTCCATGGCATTCTCACCCGAGAATGCCGTG------

9A.9 GGCGGAATGAATTTAACAAGAATGTCCATGGCATTCTCACCCGAGAATGCCGTG------

9B.16 GGCGGAATGAATTTAACAAGAATGTCCATGGCATTCTCACCCGAGAATGCCGTG------

9B.14 GGCGGAATGAATTTAACAAGAATGTCCATGGCATTCTCACCCGAGAATGCCGTG------

APHIS-S GGCGGAATGAATTTAACAAGAATGTCCATGGCATTCTCACCCGAGAATGCCGTATTGAGG

*****************************************************

9A.10 ------------------------------------------------------------

9A.8 ------------------------------------------------------------

9A.13 ------------------------------------------------------------

9B.12 ------------------------------------------------------------

9A.9 ------------------------------------------------------------

9B.16 ------------------------------------------------------------

9B.14 ------------------------------------------------------------

APHIS-S GACGTCGTATCCAGTGCTACAACAAAGTTACTGCTTAAAAACATGAGAGACCAAGTACTG

9A.10 ------------------------------------------------------------

9A.8 ------------------------------------------------------------

9A.13 ------------------------------------------------------------

9B.12 ------------------------------------------------------------

9A.9 ------------------------------------------------------------

9B.16 ------------------------------------------------------------

9B.14 ------------------------------------------------------------

APHIS-S CCCATCATTGAGGCATTGCCAATAGAAATACCGCCGGGACTGGTAAACTCGTCACAGGTG

9A.10 ------------------------------------------------------------

9A.8 ------------------------------------------------------------

9A.13 ------------------------------------------------------------

9B.12 ------------------------------------------------------------

9A.9 ------------------------------------------------------------

9B.16 ------------------------------------------------------------

9B.14 ------------------------------------------------------------

APHIS-S TACGAAATAGTTAAATTATTTGTCGACGAGAACGTTGTTACCGGATACAATAGCAGTGCG

9A.10 ------------------------------------------------------------

9A.8 ------------------------------------------------------------

9A.13 ------------------------------------------------------------

9B.12 ------------------------------------------------------------

9A.9 ------------------------------------------------------------

9B.16 ------------------------------------------------------------

9B.14 ------------------------------------------------------------

APHIS-S GCAATGAGAGGAATATACGCAGAGGAAGAAGCCACGAGAAGGGTGATAGCTGGCATAGAA

9A.10 ------------------------------------------------------------

9A.8 ------------------------------------------------------------

9A.13 ------------------------------------------------------------

9B.12 ------------------------------------------------------------

9A.9 ------------------------------------------------------------

9B.16 ------------------------------------------------------------

9B.14 ------------------------------------------------------------

APHIS-S TTCGATGACTCATTGCGTGAAATAACGGAGCTACCACTAGACTTGTCGTATGCGCTTCGT

9A.10 ------------------------------------------------------------

9A.8 ------------------------------------------------------------

9A.13 ------------------------------------------------------------

9B.12 ------------------------------------------------------------

9A.9 ------------------------------------------------------------

9B.16 ------------------------------------------------------------

9B.14 ------------------------------------------------------------

APHIS-S TTTCCGGAGAGACCTCGCTTGAATTCCTTCTTCATGACAGGCGGTCGGACTTGGCGCACA

9A.10 ------------------------------------------------------------

9A.8 ------------------------------------------------------------

9A.13 ------------------------------------------------------------

9B.12 ------------------------------------------------------------

9A.9 ------------------------------------------------------------

9B.16 ------------------------------------------------------------

9B.14 ------------------------------------------------------------

APHIS-S GATAACGTGTTTCCTATGTTCGAAGTTCCCGGACCTCGCTTTCCGTATTCATGGGAAGGT

9A.10 ------------------------------------------------------------

9A.8 ------------------------------------------------------------

9A.13 ------------------------------------------------------------

9B.12 ------------------------------------------------------------

9A.9 ------------------------------------------------------------

9B.16 ------------------------------------------------------------

9B.14 ------------------------------------------------------------

APHIS-S GGAAATGATCCAGGATACGTAAACGAGATGTTCATAGCCTTGCAGCACATGATATCTTCA

9A.10 ------------------------------------------------------------

9A.8 ------------------------------------------------------------

9A.13 ------------------------------------------------------------

9B.12 ------------------------------------------------------------

9A.9 ------------------------------------------------------------

9B.16 ------------------------------------------------------------

9B.14 ------------------------------------------------------------

APHIS-S GAACTGGTATCTAAAGTGGCGGGAGTGAACCTAGACTTCGATGTGCACATACAGAGGTAC

9A.10 ------------------------------------------------------------

9A.8 ------------------------------------------------------------

9A.13 ------------------------------------------------------------

9B.12 ------------------------------------------------------------

9A.9 ------------------------------------------------------------

9B.16 ------------------------------------------------------------

9B.14 ------------------------------------------------------------

APHIS-S CCACATCCAGCATACATCATGGACTTGGCGAAGGAAGCCCTGCAGTTCCTCTTCCCATCA

9A.10 ------------------------------------------------------------

9A.8 ------------------------------------------------------------

9A.13 ------------------------------------------------------------

9B.12 ------------------------------------------------------------

9A.9 ------------------------------------------------------------

9B.16 ------------------------------------------------------------

9B.14 ------------------------------------------------------------

APHIS-S TTCATCATGATCAGCTTCAGTTACACCGCTATCAATATTATACGATCCGTGACCGTGGAA

9A.10 ------------------------------------------------------------

9A.8 ------------------------------------------------------------

9A.13 ------------------------------------------------------------

9B.12 ------------------------------------------------------------

9A.9 ------------------------------------------------------------

9B.16 ------------------------------------------------------------

9B.14 ------------------------------------------------------------

APHIS-S AAAGAAATGCAATTGAAGGAAACGATGAAGATCATGGGACTCCCAACGTGGCTGCATTGG

9A.10 ------------------------------------------------------------

9A.8 ------------------------------------------------------------

9A.13 ------------------------------------------------------------

9B.12 ------------------------------------------------------------

9A.9 ------------------------------------------------------------

9B.16 ------------------------------------------------------------

9B.14 ------------------------------------------------------------

APHIS-S ATGGCATGGTTTTTTAAACAATTTATTTATTTGCTGATTGCTTCGGTTTTGATACTTGTT

9A.10 ------------------------------------------------------------

9A.8 ------------------------------------------------------------

9A.13 ------------------------------------------------------------

9B.12 ------------------------------------------------------------

9A.9 ------------------------------------------------------------

9B.16 ------------------------------------------------------------

9B.14 ------------------------------------------------------------

APHIS-S ATATTAAAGGTAAATTGGTTTACTACAGAAGAAGGCTTTAGCGACTATGCCGTATTCACT

9A.10 ------------------------------------------------------------

9A.8 ------------------------------------------------------------

9A.13 ------------------------------------------------------------

9B.12 ------------------------------------------------------------

9A.9 ------------------------------------------------------------

9B.16 ------------------------------------------------------------

9B.14 ------------------------------------------------------------

APHIS-S AATACACCTTGGACCGTCCTCTTCTTCTTCCTAACACTGTATCTTACGTGTACCATATTT

9A.10 ------------------------------------------------------------

9A.8 ------------------------------------------------------------

9A.13 ------------------------------------------------------------

9B.12 ------------------------------------------------------------

9A.9 ------------------------------------------------------------

9B.16 ------------------------------------------------------------

9B.14 ------------------------------------------------------------

APHIS-S TTCTGTTTCATGATAAGTGGTTTCTTTTCAAAAGCCAGTACAGCGGCGTTGTTTGGTGGG

9A.10 ------------------------------------------------------------

9A.8 ------------------------------------------------------------

9A.13 ------------------------------------------------------------

9B.12 ------------------------------------------------------------

9A.9 ------------------------------------------------------------

9B.16 ------------------------------------------------------------

9B.14 ------------------------------------------------------------

APHIS-S GTGATCTGGTTTCTGACGTATATCCCCGCATTCCTCCTGGCTATGGACGTGAACATGTCT

9A.10 ------------------------------------------------------------

9A.8 ------------------------------------------------------------

9A.13 ------------------------------------------------------------

9B.12 ------------------------------------------------------------

9A.9 ------------------------------------------------------------

9B.16 ------------------------------------------------------------

9B.14 ------------------------------------------------------------

APHIS-S ACCTCTCTACAAGCGGTCACCTGCCTAATGCTCAACTCCGCCATGTCTTACGGCTTCCAG

9A.10 ------------------------------------------------------------

9A.8 ------------------------------------------------------------

9A.13 ------------------------------------------------------------

9B.12 ------------------------------------------------------------

9A.9 ------------------------------------------------------------

9B.16 ------------------------------------------------------------

9B.14 ------------------------------------------------------------

APHIS-S CTGTTACTGGCCCGGGAAAGTACCGGAGGAATGCAGTGGGGTGATTTTATGACGTCACCA

9A.10 ------------------------------------------------------------

9A.8 ------------------------------------------------------------

9A.13 ------------------------------------------------------------

9B.12 ------------------------------------------------------------

9A.9 ------------------------------------------------------------

9B.16 ------------------------------------------------------------

9B.14 ------------------------------------------------------------

APHIS-S GCAACGGACTCGTCACGATTCGTATTCGGTCACGTCGTTATAATGATGGCTTTGAACTGT

9A.10 ------------------------------------------------------------

9A.8 ------------------------------------------------------------

9A.13 ------------------------------------------------------------

9B.12 ------------------------------------------------------------

9A.9 ------------------------------------------------------------

9B.16 ------------------------------------------------------------

9B.14 ------------------------------------------------------------

APHIS-S GTGCTCTACATGTTGATTGCCCTATATCTAGAGCAAGTACTACCCGGGCCGTATGGCACA

9A.10 ------------------------------------------------------------

9A.8 ------------------------------------------------------------

9A.13 ------------------------------------------------------------

9B.12 ------------------------------------------------------------

9A.9 ------------------------------------------------------------

9B.16 ------------------------------------------------------------

9B.14 ------------------------------------------------------------

APHIS-S CCGAAGCCCTGGTATTTCTTCGTCCAAAGACAGTTCTGGTGTAGCAGCAAAACTACTCAT

9A.10 ------------------------------------------------------------

9A.8 ------------------------------------------------------------

9A.13 ------------------------------------------------------------

9B.12 ------------------------------------------------------------

9A.9 ------------------------------------------------------------

9B.16 ------------------------------------------------------------

9B.14 ------------------------------------------------------------

APHIS-S GATATCGGTACAGACAACAGCGACACATCAAGTTTAACAAAAGAAAGCGACCCTACAGAC

9A.10 ------------------------------------------------------------

9A.8 ------------------------------------------------------------

9A.13 ------------------------------------------------------------

9B.12 ------------------------------------------------------------

9A.9 ------------------------------------------------------------

9B.16 ------------------------------------------------------------

9B.14 ------------------------------------------------------------

APHIS-S CTTCCGATTGGAGTTAAAATACAAAACCTTAAAAAGGTTTACGGGAGCAACGTTGCGGTA

9A.10 ------------------------------------------------------------

9A.8 ------------------------------------------------------------

9A.13 ------------------------------------------------------------

9B.12 ------------------------------------------------------------

9A.9 ------------------------------------------------------------

9B.16 ------------------------------------------------------------

9B.14 ------------------------------------------------------------

APHIS-S AACAATTTATCCCTCAACATTTACGACGACCAAATCACGGTTCTACTTGGACACAACGGA

9A.10 ------------------------------------------------------------

9A.8 ------------------------------------------------------------

9A.13 ------------------------------------------------------------

9B.12 ------------------------------------------------------------

9A.9 ------------------------------------------------------------

9B.16 ------------------------------------------------------------

9B.14 ------------------------------------------------------------

APHIS-S GCGGGAAAATCCACAACCATTTCAATGCTCACAGGTAACGTGGACATAACCAGCGGGTCG

9A.10 ------------------------------------------------------------

9A.8 ------------------------------------------------------------

9A.13 ------------------------------------------------------------

9B.12 ------------------------------------------------------------

9A.9 ------------------------------------------------------------

9B.16 ------------------------------------------------------------

9B.14 ------------------------------------------------------------

APHIS-S GTGACGGTGGCTGGCTACGACATAGAAAAACAAACAAGTTCAGCACGCTCACACATTGGA

9A.10 ------------------------------------------------------------

9A.8 ------------------------------------------------------------

9A.13 ------------------------------------------------------------

9B.12 ------------------------------------------------------------

9A.9 ------------------------------------------------------------

9B.16 ------------------------------------------------------------

9B.14 ------------------------------------------------------------

APHIS-S CTCTGCCCTCAACATAACGTACTCTTCAACGAACTCACAGTCAAAGAACATTTACAGTTC

9A.10 ------------------------------------------------------------

9A.8 ------------------------------------------------------------

9A.13 ------------------------------------------------------------

9B.12 ------------------------------------------------------------

9A.9 ------------------------------------------------------------

9B.16 ------------------------------------------------------------

9B.14 ------------------------------------------------------------

APHIS-S TTCTCTCGTCTGAAAGGCTTCAGCGGTAAAGAGTTGGATGAAGAAATTGAGACGCTTATT

9A.10 ------------------------------------------------------------

9A.8 ------------------------------------------------------------

9A.13 ------------------------------------------------------------

9B.12 ------------------------------------------------------------

9A.9 ------------------------------------------------------------

9B.16 ------------------------------------------------------------

9B.14 ------------------------------------------------------------

APHIS-S GAAAAATTGGAATTGCAAGAAAAGAGGGATTACCAATCAGCGGGGTTATCAGGGGGACAG

9A.10 ----------------------------TATGCGGGGCGGCTAAAGTGGTTCTACTGGAC

9A.8 ----------------------------TATGCGGGGCGGCTAAAGTGGTTCTACTGGAC

9A.13 ----------------------------TATGCGGGGCGGCTAAAGTGGTTCTACTGGAC

9B.12 ----------------------------TATGCGGGGCGGCTAAAGTGGTTCTACTGGAC

9A.9 ----------------------------TATGCGGGGCGGCTAAAGTGGTTCTACTGGAC

9B.16 ----------------------------TATGCGGGGCGGCTAAAGTGGTTCTACTGGAC

9B.14 ----------------------------TATGCGGGGCGGCTAAAGTGGTTCTACTGGAC

APHIS-S AAGCGACGATTAGGAGTGGGCGTCGCGCTATGCGGGGCGGCTAAAGTGGTTCTACTGGAC

********************************

9A.10 GAGCCCACTTCTGGCATGGACCCGGCCTCACGTCGTGCCCTGTGGGACTTGTTGCAGAGA

9A.8 GAGCCCACTTCTGGCATGGACCCGGCCTCACGTCGTGCCCTGTGGGACTTGTTGCAGAGA

9A.13 GAGCCCACTTCTGGCATGGACCCGGCCTCACGTCGTGCCCTGTGGGACTTGTTGCAGAGA

9B.12 GAGCCCACTTCTGGCATGGACCCGGCCTCACGTCGTGCCCTGTGGGACTTGTTGCAGAGA

9A.9 GAGCCCACTTCTGGCATGGACCCGGCCTCACGTCGTGCCCTGTGGGACTTGTTGCAGAGA

9B.16 GAGCCCACTTCTGGCATGGACCCGGCCTCACGTCGTGCCCTGTGGGACTTGTTGCAGAGA

9B.14 GAGCCCACTTCTGGCATGGACCCGGCCTCACGTCGTGCCCTGTGGGACTTGTTGCAGAGA

APHIS-S GAGCCCACTTCTGGCATGGACCCGGCCTCACGTCGTGCCCTATGGGACTTGTTGCAGAGA

***************************************** ******************

9A.10 GAGAAGAAAGGTCGATCGATGATCCTGACGACACACTTCATGGACGAAGCGGACATATTA

9A.8 G-TAAGAAAGGTCGATCGATGATCCTGACGACACACTTCATGGACGAAGCGGACATATTA

9A.13 GAGAAGAAAGGTCGATCGATGATCCTGACGACACACTTCATGGACGAAGCGGACATATTA

9B.12 GAGAAGAAAGGTCGATCGATGATCCTGACGACACACTTCATGGACGAAGCGGACATATTA

9A.9 GAGAAGAAAGGTCGATCGATGATCCTGACGACACACTTCATGGACGAAGCGGACATATTA

9B.16 GAGAAGAAAGGTCGATCGATGATCCTGACGACACACTTCATGGACGAAGCGGACATATTA

9B.14 GAGAAGAAAGGTCGATCGATGATCCTGACGACACACTTCATGGACGAAGCGGACATATTA

APHIS-S GAGAAGAAAGGTCGATCGATGATCCTGACGACACACTTCATGGACGAAGCGGACATATTA

* *********************************************************

9A.10 GGGGATAGAGTTGCCATTATGGCGGACGGTCGTCTCCAGTGCGTGGGCTCACCTTACTTC

9A.8 GGGGATAGAGTTGCCATTATGGCGGACGGTCGTCTCCAGTGCGTGGGCTCACCTTACTTC

9A.13 GGGGATAGAGTTGCCATTATGGCGGACGGTCGTCTCCAGTGCGTGGGCTCACCTTACTTC

9B.12 GGGGATAGAGTTGCCATTATGGCGGACGGTCGTCTCCAGTGCGTGGGCTCACCTTACTTC

9A.9 GGGGATAGAGTTGCCATTATGGCGGACGGTCGTCTCCAGTGCGTGGGCTCACCTTACTTC

9B.16 GGGGATAGAGTTGCCATTATGGCGGACGGTCGTCTCCAGTGCGTGGGCTCACCTTACTTC

9B.14 GGGGATAGAGTTGCCATTATGGCGGACGGTCGTCTCCAGTGCGTGGGCTCACCTTACTTC

APHIS-S GGGGATAGAGTTGCCATTATGGCGGACGGTCGTCTCCAGTGCGTGGGCTCACCTTACTTC

************************************************************

9A.10 CTCAAGAGACACTATGGAGTCGGCTACACGCTAGTTGTGGTCAAGAAGGAAGATTTCCGA

9A.8 CTCAAGAGACACTATGGAGTCGGCTACACGCTAGTTGTGGTCAAGAAGGAAGATTTCCGA

9A.13 CTCAAGAGACACTATGGAGTCGGCTACACGCTAGTTGTGGTCAAGAAGGAAGATTTCCGA

9B.12 CTCAAGAGACACTATGGAGTCGGCTACACGCTAGTTGTGGTCAAGAAGGAAGATTTCCGA

9A.9 CTCAAGAGACACTATGGAGTCGGCTACACGCTAGTTGTGGTCAAGAAGGAAGATTTCCGA

9B.16 CTCAAGAGACACTATGGAGTCGGCTACACGCTAGTTGTGGTCAAGAAGGAAGATTTCCGA

9B.14 CTCAAGAGACACTATGGAGTCGGCTACACGCTAGTTGTGGTCAAGAAGGAAGATTTCCGA

APHIS-S CTCAAGAGACACTATGGAGTCGGCTACACGCTAGTTGTGGTCAAGAAGGAAGATTTCCGA

************************************************************

9A.10 CTGGACACCTGCACAGAGCTGATCAATAGATACATCCCTGGAACTGTTGTGAAGGAAGAC

9A.8 CTGGACACCTGCACAGAGCTGATCAATAGATACATCCCTGGAACTGTTGTGAAGGAAGAC

9A.13 CTGGACACCTGCACAGAGCTGATCAATAGATACATCCCTGGAACTGTTGTGAAGGAAGAC

9B.12 CTGGACACCTGCACAGAGCTGATCAATAGATACATCCCTGGAACTGTTGTGAAGGAAGAC

9A.9 CTGGACACCTGCACAGAGCTGATCAATAGATACATCCCTGGAACTGTTGTGAAGGAAGAC

9B.16 CTGGACACCTGCACAGAGCTGATCAATAGATACATCCCTGGAACTGTTGTGAAGGAAGAC

9B.14 CTGGACACCTGCACAGAGCTGATCAATAGATACATCCCTGGAACTGTTGTGAAGGAAGAC

APHIS-S CTGGACACCTGCACAGAGCTGATCAATAGATACATCCCTGGAACTGTTGTGAAGGAAGAC

************************************************************

9A.10 CGAGGCACTGAAGTGACATATAGCATGACTAATGAGTATTCGCACGTGTTTGAATCTATG

9A.8 CGAGGCACTGAAGTGACATATAGCATGACTAATGAGTATTCGCACGTGTTTGAATCTATG

9A.13 CGAGGCACTGAAGTGACATATAGCATGACTAATGAGTATTCGCACGTGTTTGAATCTATG

9B.12 CGAGGCACTGAAGTGACATATAGCATGACTAATGAGTATTCGCACGTGTTTGAATCTATG

9A.9 CGAGGCACTGAAGTGACATATAGCATGACTAATGAGTATTCGCACGTGTTTGAATCTATG

9B.16 CGAGGCACTGAAGTGACATATAGCATGACTAATGAGTATTCGCACGTGTTTGAATCTATG

9B.14 CGAGGCACTGAAGTGACATATAGCATGACTAATGAGTATTCGCACGTGTTTGAATCTATG

APHIS-S CGAGGCACTGAAGTGACATATAGCATGACTAATGAGTATTCGCACGTGTTTGAATCTATG

************************************************************

9A.10 CTGCGCGATTTGGAGGCAAAAGCCGATGAGATAAACTTTAAAAACTACGGCCTACTGGCT

9A.8 CTGCGCGATTTGGAGGCAAAAGCCGATGAGATAAACTTTAAAAACTACGGCCTACTGGCT

9A.13 CTGCGCGATTTGGAGGCAAAAGCCGATGAGATAAACTTTAAAAACTACGGCCTACTGGCT

9B.12 CTGCGCGATTTGGAGGCAAAAGCCGATGAGATAAACTTTAAAAACTACGGCCTACTGGCT

9A.9 CTGCGCGATTTGGAGGCAAAAGCCGATGAGATAAACTTTAAAAACTACGGCCTACTGGCT

9B.16 CTGCGCGATTTGGAGGCAAAAGCCGATGAGATAAACTTTAAAAACTACGGCCTACTGGCT

9B.14 CTGCGCGATTTGGAGGCAAAGGCCGATGAGATAAACTTTAAAAACTACGGCCTACTGGCT

APHIS-S CTGCGCGATTTGGAGGCAAAGGCCGATGAGATAAACTTTAAAAACTACGGCCTACTGGCT

******************** ***************************************

9A.10 ACTACATTAGAAGATGTGTTCATGTCCGTGGGCACAGATGTGGTCGCAACTTCAGATGTG

9A.8 ACTACATTAGAAGATGTGTTCATGTCCGTGGGCACAGATGTGGTCGCAACTTCAGATGTG

9A.13 ACTACATTAGAAGATGTGTTCATGTCCGTGGGCACAGATGTGGTCGCAACTTCAGATGTG

9B.12 ACTACATTAGAAGATGTGTTCATGTCCGTGGGCACAGATGTGGTCGCAACTTCAGATGTG

9A.9 ACTACATTAGAAGATGTGTTCATGTCCGTGGGCACAGATGTGGTCGCAACTTCAGATGTG

9B.16 ACTACATTAGAAGATGTGTTCATGTCCGTGGGCACAGATGTGGTCGCAACTTCAGATGTG

9B.14 ACTACATTAGAAGATGTGTTCATGTCCGTGGGCACAGATGTGGTCGCAACTTCAGATGTG

APHIS-S ACTACATTAGAAGATGTGTTCATGTCCGTGGGCACAGATGTGGTCGCAACTTCAGATGTG

************************************************************

9A.10 GACGACAATACAACCGTTTCATCTAGTGCTGATACTCTAGCATTTGAATATGATTCTTTA

9A.8 GACGACAATACAACCGTTTCATCTAGTGCTGATACTCTAGCATTTGAATATGATTCTTTA

9A.13 GACGACAATACAACCGTTTCATCTAGTGCTGATACTCTAGCATTTGAATATGATTCTTTA

9B.12 GACGACAATACAACCGTTTCATCTAGTGCTGATACTCTAGCATTTGAATATGATTCTTTA

9A.9 GACGACAATACAACCGTTTCATCTAGTGCTGATACTCTAGCATTTGAATATGATTCTTTA

9B.16 GACGACAATACAACCGTTTCATCTAGTGCTGATACTCTAGCATTTGAATATGATTCTTTA

9B.14 GACGACAATACAACCGTTTCATCTAGTGCTGATACTCTAGCATTTGAATATGATTCTTTA

APHIS-S GACGACAATACAACCGTTTCATCTAGTGCTGATACTCTAGCATTTGAATATGATTCTTTA

************************************************************

9A.10 GAAAAATTGGACGGGACTGGCTATGGGGATGAAAAAGGGATCCGATTAATTTCCCAACAC

9A.8 GAAAAATTGGACGGGACTGGCTATGGGGATGAAAAAGGGATCCGATTAATTTCCCAACAC

9A.13 GAAAAATTGGACGGGACTGGCTATGGGGATGAAAAAGGGATCCGATTAATTTCCCAACAC

9B.12 GAAAAATTGGACGGGACTGGCTATGGGGATGAAAAAGGGATCCGATTAATTTCCCAACAC

9A.9 GAAAAATTGGACGGGACTGGCTATGGGGATGAAAAAGGGATCCGATTAATTTCCCAACAC

9B.16 GAAAAATTGGACGGGACTGGCTATGGGGATGAAAAAGGGATCCGATTAATTTCCCAACAC

9B.14 GAAAAATTGGACGGGACTGGCTATGGGGATGAAAAAGGGATCCGATTAATTTGCCAACAC

APHIS-S GAAAAATTGGACGGGACTGGCTATGGGGATGAAAAAGGGATCCGATTAATTTGCCAACAC

**************************************************** *******

9A.10 GTGGTAGCAATATGGATGAAACTGTTTCTGGTGCTGACAAGGTCTTGGCTTATCCTGTTG

9A.8 GTGGTAGCAATATGGATGAAACTGTTTCTGGTGCTGACAAGGTCTTGGCTTATCCTGTTG

9A.13 GTGGTAGCAATATGGATGAAACTGTTTCTGGTGCTGACAAGGTCTTGGCTTATCCTGTTG

9B.12 GTGGTAGCAATATGGATGAAACTGTTTCTGGTGCTGACAAGGTCTTGGCTTATCCTGTTG

9A.9 GTGGTAGCAATATGGATGAAACTGTTTCTGGTGCTGACAAGGTCTTGGCTTATCCTGTTG

9B.16 GTGGTAGCAATATGGATGAAACTGTTTCTGGTGCTGACAAGGTCTTGGCTTATCCTGTTG

9B.14 GTGGTAGCAATATGGATGAAACTGTTTCTGGTGCTGACAAGGTCTTGGCTTATCCTGTTG

APHIS-S GTGGTAGCAATATGGATGAAACTGTTTCTGGTGCTGACAAGGTCTTGGCTTATCCTGTTG

************************************************************

9A.10 CTCCAAGTATTGGTGTCCTTGGTACAAATCATTGCCACACTCGGAGTCATGCAGTATGTC

9A.8 CTCCAAGTATTGGTGTCCTTGGTACAAATCATTGCCACACTCGGAGTCATGCAGTATGTC

9A.13 CTCCAAGTATTGGTGTCCTTGGTACAAATCATTGCCACACTCGGAGTCATGCAGTATGTC

9B.12 CTCCAAGTATTGGTGTCCTTGGTACAAATCATTGCCACACTCGGAGTCATGCAGTATGTC

9A.9 CTCCAAGTATTGGTGTCCTTGGTACAAATCATTGCCACACTCGGAGTCATGCAGTATGTC

9B.16 CTCCAAGTATTGGTGTCCTTGGTACAAATCATTGCCACACTCGGAGTCATGCAGTATGTC

9B.14 CTCCAAGTATTGGTGTCCTTGGTACAAATCATTGCCACACTCGGAGTCATGCAGTATGTC

APHIS-S CTCCAAGTATTGGTGTCCTTGGTACAAATCATTGCCACACTCGGAGTCATGCAGTATGTC

************************************************************

9A.10 ATCTCTATGACCGAGCATATACAAAGAAGAGAACTTTCATTGGCTGAAGGTTTCGCAGGC

9A.8 ATCTCTATGACCGAGCATATACAAAGAAGAGAACTTTCATTGGCTGAAGGTTTCGCAGGC

9A.13 ATCTCTATGACCGAGCATATACAAAGAAGAGAACTTTCATTGGCTGAAGGTTTCGCAGGC

9B.12 ATCTCTATGACCGAGCATATACAAAGAAGAGAACTTTCATTGGCTGAAGGTTTCGCAGGC

9A.9 ATCTCTATGACCGAGCATATACAAAGAAGAGAACTTTCATTGGCTGAAGGTTTCGCAGGC

9B.16 ATCTCTATGACCGAGCATATACAAAGAAGAGAACTTTCATTGGCTGAAGGTTTCGCAGGC

9B.14 ATCTCTATGACCGAGCATATACAAAGAAGAGAACTTTCATTGGCTGAAGGTTTCGCAGGC

APHIS-S ATCTCTATGACCGAGCATATACAAAGAAGAGAACTTTCATTGGCTGAAGGTTTCGCAGGC

************************************************************

9A.10 ACAGAAACGCTAGTTAGTTTCAAAGGGTCGTCCCTTACATCGACAGGTTCGCTAGCGAAG

9A.8 ACAGAAACGCTAGTTAGTTTCAAAGGGTCGTCCCTTACATCGACAGGTTCGCTAGCGAAG

9A.13 ACAGAAACGCTAGTTAGTTTCAAAGGGTCGTCCCTTACATCGACAGGTTCGCTAGCGAAG

9B.12 ACAGAAACGCTAGTTAGTTTCAAAGGGTCGTCCCTTACATCGACAGGTTCGCTAGCGAAG

9A.9 ACAGAAACGCTAGTTAGTTTCAAAGGGTCGTCCCTTACATCGACAGGTTCGCTAGCGAAG

9B.16 ACAGAAACGCTAGTTAGTTTCAAAGGGTCGTCCCTTACATCGACAGGTTCGCTAGCGAAG

9B.14 ACAGAAACATTAGTTAGTTTCAAAGGGTTGTCCCCTACATCGACAGGTTCGCTAGCGAAG

APHIS-S ACAGAAACATTAGTTAGTTTCAAAGGGTTGTCCCCTACATCGACAGGTTCGCTAGCGAAG

******** ****************** ***** *************************

9A.10 GCTGCCTACGAGTCGATATTTGTAACCGCCAATAATCCCACAATGGAAATCACTGTTGTT

9A.8 GCTGCCTACGAGTCGATATTTGTAACCGCCAATAATCCCACAATGGAAATCACTGTTGTT

9A.13 GCTGCCTACGAGTCGATATTCGTAACCGCCAATAATCCCACAATGGAAATCACTGTTGTT

9B.12 GCTGCCTACGAGTCGATATTTGTAACCGCCAATAATCCCACAATGGAAATCACTGTTGTT

9A.9 GCTGCCTACGAGTCGATATTTGTAACCGCCAATAATCCCACAATGGAAATCACTGTTGTT

9B.16 GCTGCCTACGAGTCGATATTTGTAACCGCCAATAATCCCACAATGGAAATCACTGTTGTT

9B.14 GCTGCCTACGAGTCGATATTTGTAACCGCCAATAATCCCACAATGGAAATCACTGTTGTT

APHIS-S GCTGCCTACGAGTCGATATTTGTAACCGCCAATAATCCCACAATGGAAATCACTGTTGTT

******************** ***************************************

9A.10 GATAATACACCTATAGATGAATATTATTTGGAAAGAACAGATGACGTATCAGCGATGGCG

9A.8 GATAATACACCTATAGATGAATATTATTTGGAAAGAACAGATGACGTATCAGCGATGGCG

9A.13 GATAATACACCTATAGATGAATATTATTTGGAAAGAACAGATGACGTATCAGCGATGGCG

9B.12 GATAATACACCTATAGATGAATATTATTTGGAAAGAACAGATGACGTATCAGCGATGGCG

9A.9 GATAATACACCTATAGATGAATATTATTTGGAAAGAACAGATGACGTATCAGCGATGGCG

9B.16 GATAATACACCTATAGATGAATATTATTTGGAAAGAACAGATGACGTATCAGCGATGGCG

9B.14 GATAATACACCTATAGATGAATATTATTTGGAAAGAACAGATGACGTATCAGCGATGGCG

APHIS-S GATAATACACCTATAGATGAATATTATTTGGAAAGAACAGATGACGTATCAGCGATGGCG

************************************************************

9A.10 GTGCTCCGGCACAGTCTGTTGATCGGCGCGACGTTCGACGACAACTCCGCGACCGCGTGG

9A.8 GTGCTCCGGCACAGTCTGTTGATCGGCGCGACGTTCGACGACAACTCCGCGACCGCGTGG

9A.13 GTGCTCCGGCACAGTCTGTTGATCGGCGCGACGTTCGACGACAACTCCGCGACCGCGTGG

9B.12 GTGCTCCGGCACAGTCTGTTGATCGGCGCGACGTTCGACGACAACTCCGCGACCGCGTGG

9A.9 GTGCTCCGGCACAGTCTGTTGATCGGCGCGACGTTCGACGACAACTCCGCGACCGCGTGG

9B.16 GTGCTCCGGCACAGTCTGTTGATCGGCGCGACGTTCGACGACAACTCCGCGACCGCGTGG

9B.14 GTGCTCCGGCACAGTCTGTTGATCGGCGCGACGTTCGACGACCACTCCGCGACCGCGTGG

APHIS-S GTGCTCCGGCACAGTCTGTTGATCGGCGCGACGTTCGACGACCACTCCGCGACCGCGTGG

****************************************** *****************

9A.10 TTCAGCAACTTCGGTTACCACGACGTGGCCATGTCACTGGCGGCTGTGCACGCCGCCTTG

9A.8 TTCAGCAACTTCGGTTACCACGACGTGGCCATGTCACTGGCGGCTGTGCACGCCGCCTTG

9A.13 TTCAGCAACTTCGGTTACCACGACGTGGCCATGTCACTGGCGGCTGTGCACGCCGCCTTG

9B.12 TTCAGCAACTTCGGTTACCACGACGTGGCCATGTCACTGGCGGCTGTGCACGCCGCCTTG

9A.9 TTCAGCAACTTCGGTTACCACGACGTGGCCATGTCACTGGCGGCTGTGCACGCCGCCTTG

9B.16 TTCAGCAACTTCGGTTACCACGACGTGGCCATGTCACTGGCGGCTGTGCACGCCGCCTTG

9B.14 TTCAGCAACTTCGGTTACCACGACGTGGCCATGTCACTGGCTGCTGTGCACGCCGCCTTG

APHIS-S TTCAGCAACTTCGGTTACCACGACGTGGCCATGTCACTGGCTGCTGTGCACGCCGCCTTG

***************************************** ******************

9A.10 CTCAGAGCTGTCAATCCTGCAGCCAACTTGACTGTTTACAACCACCCACTTGAGGCCAAT

9A.8 CTCAGAGCTGTCAATCCTGCAGCCAACTTGACTGTTTACAACCACCCACTTGAGGCCAAT

9A.13 CTCAGAGCTGTCAATCCTGCAGCCAACTTGACTGTTTACAACCACCCACTTGAGGCCAAT

9B.12 CTCAGAGCTGTCAATCCTGCAGCCAACTTGACTGTTTACAACCACCCACTTGAGGCCAAT

9A.9 CTCAGAGCTGTCAATCCTGCAGCCAACTTGACTGTTTACAACCACCCACTTGAGGCCAAT

9B.16 CTCAGAGCTGTCAATCCTGCAGCCAACTTGACTGTTTACAACCACCCACTTGAGGCCAAT

9B.14 CTCAGAGCTGTCAATCCTGCAGCCAACTTGACTGTTTACAACCACCCACTTGAGGCCAAT

APHIS-S CTCAGAGCTGTCAATCCTGCAGCCAACTTGACTGTTTACAACCACCCACTTGAGGCCAAT

************************************************************

9A.10 TATGTCAACCAGAACGACATGCAAACGATGGTAGCGTTCCTCTCGATGCAACTTGCGTCG

9A.8 TATGTCAACCAGAACGACATGCAAACGATGGTAGCGTTCCTCTCGATGCAACTTGCGTCG

9A.13 TATGTCAACCAGAACGACATGCAAACGATGGTAGCGTTCCTCTCGATGCAACTTGCGTCG

9B.12 TATGTCAACCAGAACGACATGCAAACGATGGTAGCGTTCCTCTCGATGCAACTTGCGTCG

9A.9 TATGTCAACCAGAACGACATGCAAACGATGGTAGCGTTCCTCTCGATGCAACTTGCGTCG

9B.16 TATGTCAACCAGAACGACATGCAAACGATGGTAGCGTTCCTCTCGATGCAACTTGCGTCG

9B.14 TATGTCAACCAGAACGACATGCAAACAATGGTAGCGTTCCTCTCGATGCAACTTGCGTCG

APHIS-S TATGTCAACCAGAACGACATGCAAACAATGGTAGCGTTCCTCTCGATGCAACTTGCGTCG

************************** *********************************

9A.10 GGCATCGGCAGCAGTCTGTCAATTGTCAGTGCTGTGTTCATCATGTTCTATATCAAGGAG

9A.8 GGCATCGGCAGCAGTCTGTCAATTGTCAGTGCTGTGTTCATCATGTTCTATATCAAGGAG

9A.13 GGCATCGGCAGCAGTCTGTCAATTGTCAGTGCTGTGTTCATCATGTTCTATATCAAGGAG

9B.12 GGCATCGGCAGCAGTCTGTCAATTGTCAGTGCTGTGTTCATCATGTTCTATATCAAGGAG

9A.9 GGCATCGGCAGCAGTCTGTCAATTGTCAGTGCTGTGTTCATCATGTTCTATATCAAGGAG

9B.16 GGCATCGGCAGCAGTCTGTCAATTGTCAGTGCTGTGTTCATCATGTTCTATATCAAGGAG

9B.14 GGCATCGGCAGCAGTCTGTCAATTGTCAGTGCTGTGTTCATCATGTTCTATATCAAGGAG

APHIS-S GGCATCGGCAGCAGTCTGTCAATTGTCAGTGCTGTGTTCATCATGTTCTATATCAAGGAG

************************************************************

9A.10 CGAGTATCTCGCGCCAAGCTGCTGCAGAAGGCGGCAGGCATCCAGCCGTTAGTGATGTGG

9A.8 CGAGTATCTCGCGCCAAGCTGCTGCAGAAGGCGGCAGGCATCCAGCCGTTAGTGATGTGG

9A.13 CGAGTATCTCGCGCCAAGCTGCTGCAGAAGGCGGCAGGCATCCAGCCGTTAGTGATGTGG

9B.12 CGAGTATCTCGCGCCAAGCTGCTGCAGAAGGCGGCAGGCATCCAGCCGTTAGTGATGTGG

9A.9 CGAGTATCTCGCGCCAAGCTGCTGCAGAAGGCGGCAGGCATCCAGCCGTTAGTGATGTGG

9B.16 CGAGTATCTCGCGCCAAGCTGCTGCAGAAGGCGGCAGGCATCCAGCCGTTAGTGATGTGG

9B.14 CGAGTATCTCGCGCCAAGCTGCTGCAGAAGGCGGCAGGCATCCAGCCGTTAGTGATGTGG

APHIS-S CGAGTATCTCGCGCCAAGCTGCTGCAGAAGGCGGCAGGCATCCAGCCGTTAGTGATGTGG

************************************************************

9A.10 CTCAGCGCCGCCGTGTTCGACTGGATCTGGTTCTGCATCATCGCCGTCGGCATCGTTATC

9A.8 CTCAGCGCCGCCGTGTTCGACTGGATCTGGTTCTGCATCATCGCCGTCGGCATCGTTATC

9A.13 CTCAGCGCCGCCGTGTTCGACTGGATCTGGTTCTGCATCATCGCCGTCGGCATCGTTATC

9B.12 CTCAGCGCCGCCGTGTTCGACTGGATCTGGTTCTGCATCATCGCCGTCGGCATCGTTATC

9A.9 CTCAGCGCCGCCGTGTTCGACTGGATCTGGTTCTGCATCATCGCCGTCGGCATCGTTATC

9B.16 CTCAGCGCCGCCGTGTTCGACTGGATCTGGTTCTGCATCATCGCCGTCGGCATCGTTATC

9B.14 CTCAGCGCCGCCGTGTTCGACTGGATCTGGTTCTGCGTCATCGCCGTCGGCATCGTTATC

APHIS-S CTCAGCGCCGCCGTGTTCGACTGGATCTGGTTCTGCGTCATCGCCGTCGGCATCGTTATC

************************************ ***********************

9A.10 GCCTGCGCCGCTTT----------------------------------------------

9A.8 GCCTGCGCCGCTTT----------------------------------------------

9A.13 GCCTGCGCCGCTTT----------------------------------------------

9B.12 GCCTGCGCCGCTTT----------------------------------------------

9A.9 GCCTGCGCCGCTTT----------------------------------------------

9B.16 GCCTGCGCCGCTTT----------------------------------------------

9B.14 GCCTGCGCCGCTTTTGCGCGCCGTGTTTATTACGGTCATGGCGTCAAAAGATATAGAGCT

APHIS-S GCCTGCGCCGCTTT----------------------------------------------

**************

9A.10 ---TAACGTCATTGGGC----------------TCTCTTCTGTCGATGAACTGGGTCGGA

9A.8 ---TAACGTCATTGGGC----------------TCTCTTCTGTCGATGAACTGGGTCGGA

9A.13 ---TAACGTCATTGGGC----------------TCTCTTCTGTCGATGAACTGGGTCGGA

9B.12 ---TAACGTCATTGGGC----------------TCTCTTCTGTCGATGAACTG-------

9A.9 ---TAACGTCATTGGGC----------------TCTCTTCTGTCGATGAACTGGGTCGGA

9B.16 ---TAACGTCATTGGGC----------------TCTCTTCTGTCGATGAACTGGGTCGGA

9B.14 GAATACCTTTATTGTGTATTTCAGTGTTCCACATCGCTTTACTCGGTGAA----GTCGGA

APHIS-S ---TAACGTCATTGGGC----------------TCTCTTCTGTCGATGAACTGGGTCGGA

** * * **** * ** *** *** ****

9A.10 TGTACTTGTGCATCATAGTGTATGGCGCCGCCAGTCTGCCGATAGGCTACGTGTTCTCCT

9A.8 TGTACTTGTGCATCATAGTGTATGGCGCCGCCAGTCTGCCGATAGGCTACGTGTTCTCCT

9A.13 TGTACTTGTGCATCATAGTGTATGGCGCCGCCAGTCTGCCGATAGGCTACGTGTTCTCCT

9B.12 ------------------------------------------------------------

9A.9 TGTACTTGTGCATCATAGTGTATGGCGCCGCCAGTCTGCCGATAGGCTACGTGTTCTCCT

9B.16 TGTACTTGTGCATCATAGTGTATGGCGCCGCCAGTCTGCCGATAGGCTACGTGTTCTCCT

9B.14 TGTACTTGTGCATCATAGTGTATGGCGCCGCCAGTCTACCGATAGGCTACGTGTTCTCCT

APHIS-S TGTACTTGTGCATCATAGTGTATGGCGCCGCCAGTCTACCGATAGGCTACGTGTTCTCCT

9A.10 ATTTCTTCAAAGGCCCTGCCGTCGGTTTTGTCACCATGTTCTTTATCAACATTCTCTTTG

9A.8 ATTTCTTCAAAGGCCCTGCCGTCGGTTTTGTCACCATGTTCTTTATCAACATTCTCTTTG

9A.13 ATTTCTTCAAAGGCCCTGCCGTCGGTTTTGTCACCATGTTCTTTATCAACATTCTCTTTG

9B.12 -----------------------------------------------------------G

9A.9 ATTTCTTCAAAGGCCCTGCCGTCGGTTTTGTCACCATGTTCTTTATCAACATTCTCTTTG

9B.16 ATTTCTTCAAAGGCCCTGCCGTCGGTTTTGTCACCATGTTCTTTATCAACATTCTCTTTG

9B.14 ATTTCTTCAAAGGCCCTGCCGTCGGTTTTGTCACCATGTTCTTTATCAACATTCTCTTTG

APHIS-S ATTTCTTCAAAGGCCCTGCCGTCGGTTTTGTCACCATGTTCTTTATCAACATTCTCTTTG

*

9A.10 GTATGATGGGGGCGCAGATTGTGGAGGCCTTGTTGTCACCGCAGCTTGATACTGAAAATG

9A.8 GTATGATGGGGGCGCAGATTGTGGAGGCCTTGTTGTCACCGCAGCTTGATACTGAAAATG

9A.13 GTATGATGGGGGCGCAGATTGTGGAGGCCTTGTTGTCACCGCAGCTTGATACTGAAAATG

9B.12 GTATGATGGGGGCGCAGATTGTGGAGGCCTTGTTGTCACCGCAGCTTGATACTGAAAATG

9A.9 GTATGATGGGGGCGCAGATTGTGGAGGCCTTGTTGTCACCGCAGCTTGATACTGAAAATG

9B.16 GTATGATGGGGGCGCAGATTGTGGAGGCCTTGTTGTCACCGCAGCTTGATACTGAAAATG

9B.14 GTATGATGGGGGCGCAGATTGTGGAGGCCTTGTTGTCACCGCAGCTTGATACTGAAAATG

APHIS-S GTATGATGGGGGCGCAGATTGTGGAGGCCTTGTTGTCACCGCAGCTTGATACTGAAAATG

************************************************************

9A.10 TCGCTAATATACTTGACTCCATCTTGCAATTCTTCCCACTCTATGGTCTTGTCACATCTG

9A.8 TCGCTAATATACTTGACTCCATCTTGCAATTCTTCCCACTCTATGGTCTTGTCACATCTG

9A.13 TCGCTAATATACTTGACTCCATCTTGCAATTCTTCCCACTCTATGGTCTTGTCACATCTG

9B.12 TCGCTAATATACTTGACTCCATCTTGCAATTCTTCCCACTCTATGGTCTTGTCACATCTG

9A.9 TCGCTAATATACTTGACTCCATCTTGCAATTCTTCCCACTCTATGGTCTTGTCACATCTG

9B.16 TCGCTAATATACTTGACTCCATCTTGCAATTCTTCCCACTCTATGGTCTTGTCACATCTG

9B.14 TCGCTAATATACTTGACTCCATCTTGCAATTCTTCCCACTCTATAGTCTTGTCACATCTG

APHIS-S TCGCTAATATACTTGACTCCATCTTGCAATTCTTCCCACTCTATAGTCTTGTCACATCTG

******************************************** ***************

9A.10 CCAGACTGTTGAATCAGGTGGGACTGCTGGAGTGGTCATGCCTGCAGAACTGCGAGTACC

9A.8 CCAGACTGTTGAATCAGGTGGGACTGCTGGAGTGGTCATGCCTGCAGAACTGCGAGTACC

9A.13 CCAGACTGTTGAATCAGGTGGGACTGCTGGAGTGGTCATGCCTGCAGAACTGCGAGTACC

9B.12 CCAGACTGTTGAATCAGGTGGGACTGCTGGAGTGGTCATGCCTGCAGAACTGCGAGTACC

9A.9 CCAGACTGTTGAATCAGGTGGGACTGCTGGAGTGGTCATGCCTGCAGAACTGCGAGTACC

9B.16 CCAGACTGTTGAATCAGGTGGGACTGCTGGAGTGGTCATGCCTGCAGAACTGCGAGTACC

9B.14 CCAGACTGTTGAATCAGGTGGGACTGCTGGAGTGGTCGTGCCTGCAGAACTGCGAGTACC

APHIS-S CCAGACTGTTGAATCAGGTGGGACTGCTGGAGTGGTCGTGCCTGCAGAACTGCGAGTACC

************************************* **********************

9A.10 TGTCCGCAGTGATGCCCAACTTGACCGAATGCTCCATGGACGTAATGTGCCAGACGTTCT

9A.8 TGTCCGCAGTGATGCCCAACTTGACCGAATGCTCCATGGACGTAATGTGCCAGACGTTCT

9A.13 TGTCCGCAGTGATGCCCAACTTGACCGAATGCTCCATGGACGTAATGTGCCAGACGTTCT

9B.12 TGTCCGCAGTGATGCCCAACTTGACCGAATGCTCCATGGACGTAATGTGCCAGACGTTCT

9A.9 TGTCCGCAGTGATGCCCAACTTGACCGAATGCTCCATGGACGTAATGTGCCAGACGTTCT

9B.16 TGTCCGCAGTGATGCCCAACTTGACCGAATGCTCCATGGACGTAATGTGCCAGACGTTCT

9B.14 TGTCCGCAGTGATGCCCAACTTGACCGAATGCTCCATGGACGTTATGTGCCAGACGTTCT

APHIS-S TGTCCGCAGTGATGCCCAACTTGACCGAATGCTCCATGGACGTTATGTGCCAGACGTTCT

******************************************* ****************

9A.10 CACAATGTTGCATCCCTGACGATCCTTGGTTCATGTGGGATCACCCTGGAGTACTCCGCT

9A.8 CACAATGTTGCATCCCTGACGATCCTTGGTTCATGTGGGATCACCCTGGAGTACTCCGCT

9A.13 CACAATGTTGCATCCCTGACGATCCTTGGTTCATGTGGGATCACCCTGGAGTACTCCGCT

9B.12 CACAATGTTGCATCCCTGACGATCCTTGGTTCATGTGGGATCACCCTGGAGTACTCCGCT

9A.9 CACAATGTTGCATCCCTGACGATCCTTGGTTCATGTGGGATCACCCTGGAGTACTCCGCT

9B.16 CACAATGTTGCATCCCTGACGATCCTTGGTTCATGTGGGATCACCCTGGAGTACTCCGCT

9B.14 CACAATGTTGCATCCCAGACGATCCTTGGTTCATGTGGGATCACCCTGGAGTACTCCGCT

APHIS-S CACAATGTTGCATCCCAGACGATCCTTGGTTCATGTGGGATCACCCTGGAGTACTCCGCT

**************** *******************************************

9A.10 ACATAGTATGCATGATCGTCAGTGGAGTTGTCATGTGGTTCGTACTCTTGATCGCCGAGT

9A.8 ACATAGTATGCATGATCGTCAGTGGAGTTGTCATGTGGTTCGTACTCTTGATCGCCGAGT

9A.13 ACATAGTATGCATGATCGTCAGTGGAGTTGTCATGTGGTTCGTACTCTTGATCGCCGAGT

9B.12 ACATAGTATGCATGATCGTCAGTGGAGTTGTCATGTGGTTCGTACTCTTGATCGCCGAGT

9A.9 ACATAGTATGCATGATCGTCAGTGGAGTTGTCATGTGGTTCGTACTCTTGATCGCCGAGT

9B.16 ACATAGTATGCATGATCGTCAGTGGAGTTGTCATGTGGTTCGTACTCTTGATCGCCGAGT

9B.14 ACATAGTATGCATGATCGTCAGTGGAGTTGTCATGTGGTTCGTACTCTTGATCGCCGAGT

APHIS-S ACATAGTATGCATGATCGTCAGTGGAGTTGTCATGTGGTTCGTACTCTTGATCGCCGAGT

************************************************************

9A.10 ATCGATTGTTCCAGAAGGTGATCTACCGGGAAAAGAAAGCTCCTCCAGTTGATGAGAGCG

9A.8 ATCGATTGTTCCAGAAGGTGATCTACCGGGAAAAGAAAGCTCCTCCAGTTGATGAGAGCG

9A.13 ATCGATTGTTCCAGAAGGTGATCTACCGGGAAAAGAAAGCTCCTCCAGTTGATGAGAGCG

9B.12 ATCGATTGTTCCAGAAGGTGATCTACCGGGAAAAGAAAGCTCCTCCAGTTGATGAGAGCG

9A.9 ATCGATTGTTCCAGAAGGTGATCTACCGGGAAAAGAAAGCTCCTCCAGTTGATGAGAGCG

9B.16 ATCGATTGTTCCAGAAGGTGATCTACCGGG-AAAGAAAGCTCCTCCAGTTGATGAGAGCG

9B.14 ATCGATTGTTCCAGAAGGTGATCTACCGGGAAAAGAAAGCTCCTCCAGTTGATGAGAGCG

APHIS-S ATCGATTGTTCCAGAAGGTGATCTACCGGGAAAAGAAAGCTCCTCCAGTTGATGAGAGCG

****************************** *****************************

9A.10 CACTGGACAATGACGTGGCGGACGAGGCCAGACACGTGGCGCGAGTTGGAGCAGGAGCAA

9A.8 CACTGGACAATGACGTGGCGGACGAGGCCAGACACGTGGCGCGAGTTGGAGCAGGAGCAA

9A.13 CACTGGACAATGACGTGGCGGACGAGGCCAGACACGTGGCGCGAGTTGGAGCAGGAGCAA

9B.12 CACTGGACAATGACGTGGCGGACGAGGCCAGACACGTGGCGCGAGTTGGAGCAGGAGCAA

9A.9 CACTGGACAATGACGTGGCGGACGAGGCCAGACACGTGGCGCGAGTTGGAGCAGGAGCAA

9B.16 CACTGGACAATGACGTGGCGGACGAGGCCAGACACGTGGCGCGAGTTGGAGCAGGAGCAA

9B.14 CACTGGACAATGACGTGGCGGACGAGGCCAGACACGTGGCGCGAGTTGGAGCAGGAGCAA

APHIS-S CACTGGACAATGACGTGGCGGACGAGGCCAGACACGTGGCGCGAGTTGGAGCAGGAGCAA

************************************************************

9A.10 TCCTCGGGCAGCACAGCCTAGTAGCAAATGGCCTCACCAAGTATTATGGGAAACACCTTG

9A.8 TCCTCGGGCAGCACAGCCTAGTAGCAAATGGCCTCACCAAGTATTATGGGAAACACCTTG

9A.13 TCCTCGGGCAGCACAGCCTAGTAGCAAATGGCCTCACCAAGTATTATGGGAAACACCTTG

9B.12 TCCTCGGGCAGCACAGCCTAGTAGCAAATGGCCTCACCAAGTATTATGGGAAACACCTTG

9A.9 TCCTCGGGCAGCACAGCCTAGTAGCAAATGGCCTCACCAAGTATTATGGGAAACACCTTG

9B.16 TCCTCGGGCAGCACAGCCTAGTAGCAAATGGCCTCACCAAGTATTATGGGAAACACCTTG

9B.14 TCCTCGGGCAGCACAGCCTAGTAGCAAATGGCCTCACCAAGTATTATGGGAAACACCTTG

APHIS-S TCCTCGGGCAGCACAGCCTAGTAGCAAATGGCCTCACCAAGTATTATGGGAAACACCTTG

************************************************************

9A.10 CAGTCAATCAAGTGTCATTCACCGTGGGCGACACGGAATGCTTTGGTCTTCTGGGTGTGA

9A.8 CAGTCAATCAAGTGTCATTCACCGTGGGCGACACGGAATGCTTTGGTCTTCTGGGTGTGA

9A.13 CAGTCAATCAAGTGTCATTCACCGTGGGCGACACGGAATGCTTTGGTCTTCTGGGTGTGA

9B.12 CAGTCAATCAAGTGTCATTCACCGTGGGCGACACGGAATGCTTTGGTCTTCTGGGTGTGA

9A.9 CAGTCAATCAAGTGTCATTCACCGTGGGCGACACGGAATGCTTTGGTCTTCTGGGTGTGA

9B.16 CAGTCAATCAAGTGTCATTCACCGTGGGCGACACGGAATGCTTTGGTCTTCTGGGTGTGA

9B.14 CAGTCAATCAAGTGTCATTCACCGTGGGCGACACGGAATGCTTTGGTCTTCTGGGTGTGA

APHIS-S CAGTCAATCAAGTGTCATTCACCGTGGGCGACACGGAATGCTTTGGTCTTCTGGGTGTGA

************************************************************

9A.10 ACGGCGCCGGTAAGACGACCACCTTCAAGATGTTGATGGGAGATGAGACCGTCTCCAGCG

9A.8 ACGGCGCCGGTAAGACGACCACCTTCAAGATGTTGATGGGAGATGAGACCGTCTCCAGCG

9A.13 ACGGCGCCGGTAAGACGACCACCTTCAAGATGTTGATGGGAGATGAGACCGTCTCCAGCG

9B.12 ACGGCGCCGGTAAGACGACCACCTTCAAGATGTTGATGGGAGATGAGACCGTCTCCAGCG

9A.9 ACGGCGCCGGTAAGACGACCACCTTCAAGATGTTGATGGGAGATGAGACCGTCTCCAGCG

9B.16 ACGGCGCCGGTAAGACGACCACCTTCAAGATGTTGATGGGAGATGAGACCGTCTCCAGCG

9B.14 ACGGCGCCGGTAAGACGACCACCTTCAAGATGTTGATGGGAGATGAGACCGTCTCCAGCG

APHIS-S ACGGCGCCGGTAAGACGACCACCTTCAAGATGTTGATGGGAGATGAGACCGTCTCCAGCG

************************************************************

9A.10 GAGATGCCTTCGTGAGTGGCCATTCTGTCAAGACTAATATCACTCAAGTTTACAAAAATA

9A.8 GAGATGCCTTCGTGAGTGGCCATTCTGTCAAGACTAATATCACTCAAGTTTACAAAAATA

9A.13 GAGATGCCTTCGTGAGTGGCCATTCTGTCAAGACTAATATCACTCAAGTTTACAAAAATA

9B.12 GAGATGCCTTCGTGAGTGGCCATTCTGTCAAGACTAATATCACTCAAGTTTACAAAAATA

9A.9 GAGATGCCTTCGTGAGTGGCCATTCTGTCAAGACTAATATCACTCAAGTTTACAAAAATA

9B.16 GAGATGCCTTCGTGAGTGGCCATTCTGTCAAGACTAATATCACTCAAGTTTACAAAAATA

9B.14 GAGATGCCTTCGTGAGTGGCCATTCTGTCAAGACTAATATCACTCAAGTTTACAAAAATA

APHIS-S GAGATGCCTTCGTGAGTGGCCATTCTGTCAAGACTAATATCACTCAAGTTTACAAAAATA

************************************************************

9A.10 TTGGTGAGTGCAACTTGACTTTGAAGACTTTGTTCAGTTTTAAATGGGTTTATTTTAGAA

9A.8 TT----------------------------------------------------------

9A.13 TT----------------------------------------------------------

9B.12 TT----------------------------------------------------------

9A.9 TT----------------------------------------------------------

9B.16 TT----------------------------------------------------------

9B.14 TT----------------------------------------------------------

APHIS-S TT----------------------------------------------------------

**

9A.10 GTTAATGCCATCCCAGATTAATGGTAGAACTTTCAACTCTTAGTGTCATGCAGATGTTAT

9A.8 ------------------------------------------------------------

9A.13 ------------------------------------------------------------

9B.12 ------------------------------------------------------------

9A.9 ------------------------------------------------------------

9B.16 ------------------------------------------------------------

9B.14 ------------------------------------------------------------

APHIS-S ------------------------------------------------------------

9A.10 TATTGAAATATTTACTTTTTTCTGTTCTCTGCTCTTAACGCACTTCCATTTGTTTTATGT

9A.8 ------------------------------------------------------------

9A.13 ------------------------------------------------------------

9B.12 ------------------------------------------------------------

9A.9 ------------------------------------------------------------

9B.16 ------------------------------------------------------------

9B.14 ------------------------------------------------------------

APHIS-S ------------------------------------------------------------

9A.10 TGTGTTTGTTTCATTTGGATATTATTTAGTTTTTCGTAATGACAGTCCACCATAATCATC

9A.8 ------------------------------------------------------------

9A.13 ------------------------------------------------------------

9B.12 ------------------------------------------------------------

9A.9 ------------------------------------------------------------

9B.16 ------------------------------------------------------------

9B.14 ------------------------------------------------------------

APHIS-S ------------------------------------------------------------

9A.10 AACTTTTTTCGGGCAGTGTGATTACTCACTCGAGGAGAAATAAAACCGATATGATGCATT

9A.8 ------------------------------------------------------------

9A.13 ------------------------------------------------------------

9B.12 ------------------------------------------------------------

9A.9 ------------------------------------------------------------

9B.16 ------------------------------------------------------------

9B.14 ------------------------------------------------------------

APHIS-S ------------------------------------------------------------

9A.10 TTCATATTATTATGAATTCGATTGTGGTGGAGTATGCTCCATACCCCCTCCGGTTGATTG

9A.8 ------------------------------------------------------------

9A.13 ------------------------------------------------------------

9B.12 ------------------------------------------------------------

9A.9 ------------------------------------------------------------

9B.16 ------------------------------------------------------------

9B.14 ------------------------------------------------------------

APHIS-S ------------------------------------------------------------

9A.10 AGGGGAGGCCTGTGCCCAGCAGTGGGACGTATATAGGCAGTTTATGTTATGTTATGAATT

9A.8 ------------------------------------------------------------

9A.13 ------------------------------------------------------------

9B.12 ------------------------------------------------------------

9A.9 ------------------------------------------------------------

9B.16 ------------------------------------------------------------

9B.14 ------------------------------------------------------------

APHIS-S ------------------------------------------------------------

9A.10 CGATTATTTAAATTCGAGAACTGTTTACTGTTCGAGACAATTGCAGAGGTTAGGGAATAA

9A.8 ------------------------------------------------------------

9A.13 ------------------------------------------------------------

9B.12 ------------------------------------------------------------

9A.9 ------------------------------------------------------------

9B.16 ------------------------------------------------------------

9B.14 ------------------------------------------------------------

APHIS-S ------------------------------------------------------------

9A.10 CGGCTTTCGCGTGGATTATAAATACGGTTAGGTTACAACTATAAGTCCAAACGCCTCCAC

9A.8 ------------------------------------------------------------

9A.13 ------------------------------------------------------------

9B.12 ------------------------------------------------------------

9A.9 ------------------------------------------------------------

9B.16 ------------------------------------------------------------

9B.14 ------------------------------------------------------------

APHIS-S ------------------------------------------------------------

9A.10 CACCCCAAATTCAGTGGATTCAGTGGAGTAGGCTGCATGTCTACTACGGATGATTGAGGA

9A.8 ------------------------------------------------------------

9A.13 ------------------------------------------------------------

9B.12 ------------------------------------------------------------

9A.9 ------------------------------------------------------------

9B.16 ------------------------------------------------------------

9B.14 ------------------------------------------------------------

APHIS-S ------------------------------------------------------------

9A.10 GGAAGCCTGTGCCTATGTGGGGGGTGCCTAGCAGTGGGACGCCAACAAGTCTTCTTTTCC

9A.8 ------------------------------------------------------------

9A.13 ------------------------------------------------------------

9B.12 ------------------------------------------------------------

9A.9 ------------------------------------------------------------

9B.16 ------------------------------------------------------------

9B.14 ------------------------------------------------------------

APHIS-S ------------------------------------------------------------

9A.10 TTGATCTTTGCCTATAAGTATTAATGTGTGTTTTAGGTTCACTTCTACACTTCACTTTTT

9A.8 ------------------------------------------------------------

9A.13 ------------------------------------------------------------

9B.12 ------------------------------------------------------------

9A.9 ------------------------------------------------------------

9B.16 ------------------------------------------------------------

9B.14 ------------------------------------------------------------

APHIS-S ------------------------------------------------------------

9A.10 AAAAGTTTATTGCTGATGTTTTAGGTTACTGTCCGCAATTCGAAGCGACATTCGGCGAGC

9A.8 -----------------------GGTTACTGTCCGCAATTCGAAGCGACATTCGGCGAGC

9A.13 -----------------------GGTTACTGTCCGCAATTCGAAGCGACATTCGGCGAGC

9B.12 -----------------------GGTTACTGTCCGCAATTCGAAGCGACATTCGGCGAGC

9A.9 -----------------------GGTTACTGTCCGCAATTCGAAGCGACATTCGGCGAGC

9B.16 -----------------------GGTTACTGTCCGCAATTCGAAGCGACATTCGGCGAGC

9B.14 -----------------------GGTTACTGTCCGCAATTCGAAGCGACATTCGGCGAGC

APHIS-S -----------------------GGTTACTGTCCGCAATTCGAAGCGACATTCGGCGAGC

*************************************

9A.10 TGACGGGACGCGAGACACTACGGCTGTTCTCGGCGCTGCGAGGGTTGCCAGTGCGAGGCG

9A.8 TGACGGGACGCGAGACACTACGGCTGTTCTCGGCGCTGCGAGGGTTGCCAGTGCGAGGCG

9A.13 TGACGGGACGCGAGACACTACGGCTGTTCTCGGCGCTGCGAGGGTTGCCAGTGCGAGGCG

9B.12 TGACGGGACGCGAGACACTACGGCTGTTCTCGGCGCTGCGAGGGTTGCCAGTGCGAGGCG

9A.9 TGACGGGACGCGAGACACTACGGCTGTTCTCGGCGCTGCGAGGGTTGCCAGTGCGAGGCG

9B.16 TGACGGGACGCGAGACACTACGGCTGTTCTCGGCGCTGCGAGGGTTGCCAGTGCGAGGCG

9B.14 TGACGGGACGCGAGACACTACGGCTGTTCTCGGCGCTGCGAGGGTTGCCAGTGCGAGGCG

APHIS-S TGACGGGACGCGAGACACTACGGCTGTTCTCGGCGCTGCGAGGGTTGCCAGTGCGAGGCG

************************************************************

9A.10 CCACGCTCCACGCGGAGGCCTTAGCACATGCTCTTGGTTTCTATAAGCATCTTGATAAAA

9A.8 CCACGCTCCACGCGGAGGCCTTAGCACATGCTCTTGGTTTCTATAAGCATCTTGATAAAA

9A.13 CCACGCTCCACGCGGAGGCCTTAGCACATGCTCTTGGTTTCTATAAGCATCTTGATAAAA

9B.12 CCACGCTCCACGCGGAGGCCTTAGCACATGCTCTTGGTTTCTATAAGCATCTTGATAAAA

9A.9 CCACGCTCCACGCGGAGGCCTTAGCACATGCTCTTGGTTTCTATAAGCATCTTGATAAAA

9B.16 CCACGCTCCACGCGGAGGCCTTAGCACATGCTCTTGGTTTCTATAAGCATCTTGATAAAA

9B.14 CCACGCTCCACGCGGAGGCCTTAGCACATGCTCTTGGTTTCTATAAGCATCTTGATAAAA

APHIS-S CCACGCTCCACGCGGAGGCCTTAGCACATGCTCTTGGTTTCTATAAGCATCTTGATAAAA

************************************************************

9A.10 GGGTGGACCACTACTCTGGTGGCAACAAGCGCAAGTTGAGCACGGCTGTGGCGTTGCTTG

9A.8 GGGTGGACCACTACTCTGGTGGCAACAAGCGCAAGTTGAGCACGGCTGTGGCGTTGCTGG

9A.13 GGGTGGACCACTACTCTGGTGGCAACAAGCGCAAGTTGAGCACGGCTGTGGCGTTGCTTG

9B.12 GGGTGGACCACTACTCTGGTGGCAACAAGCGCAAGTTGAGCACGGCTGTGGCGTTGCTTG

9A.9 GGGTGGACCACTACTCTGGTGGCAACAAGCGCAAGTTGAGCACGGCTGTGGCGTTGCTTG

9B.16 GGGTGGACCACTACTCTGGTGGCAACAAGCGCAAGTTGAGCACGGCTGTGGCGTTGCTTG

9B.14 GGGTGGACCACTACTCTGGTGGCAACAAGCGCAAGTTGAGCACGGCTGTGGCGTTGCTGG

APHIS-S GGGTGGACCACTACTCTGGTGGCAACAAGCGCAAGTTGAGCACGGCTGTGGCGTTGCTGG

********************************************************** *

9A.10 GGCGCACGCGGCTTATATTCGTCGACGAACCCACTACTGGAGTCGATCCCGCTGCTAAGA

9A.8 GGCGCACGCGGCTTATATTCGTCGACGAACCCACTACTGGAGTCGATCCTGCTGCTAAGA

9A.13 GGCGCACGCGGCTTATATTCGTCGACGAACCCACTACTGGAGTCGATCCCGCTGCTAAGA

9B.12 GGCGCACGCGGCTTATATTCGTCGACGAACCCACTACTGGAGTCGATCCCGCTGCTAAGA

9A.9 GGCGCACGCGGCTTATATTCGTCGACGAACCCACTACTGGAGTCGATCCCGCTGCTAAGA

9B.16 GGCGCACGCGGCTTATATTCGTCGACGAACCCACTACTGGAGTCGATCCCGCTGCTAAGA

9B.14 GGCGCACGCGGCTTATATTCGTCGACGAACCCACTACTGGAGTCGATCCTGCTGCTAAGA

APHIS-S GGCGCACGCGGCTTATATTCGTCGACGAACCCACTACTGGAGTCGATCCTGCTGCTAAGA

************************************************* **********

9A.10 GACAGATGTGGAACGCGGTYSRRRAAGCTCGCCGGTCGGGTCGTGGTGTGGTGCTGACAT

9A.8 GACAGATGTGGAACGCGGTTCGAGAAGCTCGCCGGTCGGGTCGTGGTGTGGTGCTGACAT

9A.13 GACAGATGTGGAACGCGGTTCGAGAAGCTCGCCGGTCGGGTCGTGGTGTGGTGCTGACAT

9B.12 GACAGATGTGGAACGCGGTTCGAGAAGCTCGCCGGTCGGGTCGTGGTGTGGTGCTGACAT

9A.9 GACAGATGTGGAACGCGGTTCGAGAAGCTCGCCGGTCGGGTCGTGGTGTGGTGCTGACAT

9B.16 GACAGATGTGGAACGCGGTTCGAGAAGCTCGCCGGTCGGGTCGTGGTGTGGTGCTGACAT

9B.14 GACAGATGTGGAACGCGGTTCGAGAAGCTCGCCGGTCGGGTCGTGGTGTGGTGCTGACAT

APHIS-S GACAGATGTGGAACGCGGTTCGAGAAGCTCGCCGGTCGGGTCGTGGTGTGGTGCTGACAT

******************* ************************************

9A.10 CACACAGCATGGAGGAGTGTGAGGCTCTGTGCTCGCGGCTCACAATCATGGTCAACGGAC

9A.8 CACACAGCATGGAGGAGTGTGAGGCTCTGTGCTCGCGGCTCACAATCATGGTCAACGGAC

9A.13 CACACAGCATGGAGGAGTGTGAGGCTCTGTGCTCGCGGCTCACAATCATGGTCAACGGAC

9B.12 CACACAGCATGGAGGAGTGTGAGGCTCTGTGCTCGCGGCTCACAATCATGGTCAACGGAC

9A.9 CACACAGCATGGAGGAGTGTGAGGCTCTGTGCTCGCGGCTCACAATCATGGTCAACGGAC

9B.16 CACACAGCATGGAGGAGTGTGAGGCTCTGTGCTCGCGGCTCACAATCATGGTCAACGGAC

9B.14 CACACAGCATGGAGGAGTGTGAGGCTCTGTGCTCGCGGCTCACAATCATGGTCAACGGAC

APHIS-S CACACAGCATGGAGGAGTGTGAGGCTCTGTGCTCGCGGCTCACAATCATGGTCAACGGAC

************************************************************

9A.10 AGTTCCAGTGCCTCGGCACGCCGCAACATTTAAAGAATAAGTTCTCTGAAGGTTTCACAT

9A.8 AGTTCCAGTGCCTCGGCACGCCGCAACATTTAAAGAATAAGTTCTCTGAAGGTTTCACAT

9A.13 AGTTCCAGTGCCTCGGCACGCCGCAACATTTAAAGAATAAGTTCTCTGAAGGTTTCACAT

9B.12 AGTTCCAGTGCCTCGGCACGCCGCAACATTTAAAGAATAAGTTCTCTGAAGGTTTCACAT

9A.9 AGTTCCAGTGCCTCGGCACGCCGCAACATTTAAAGAATAAGTTCTCTGAAGGTTTCACAT

9B.16 AGTTCCAGTGCCTCGGCACGCCGCAACATTTAAAGAATAAGTTCTCTGAAGGTTTCACAT

9B.14 AGTTCCAGTGCCTCGGCACGCCGCAACATTTAAAGAATAAGTTCTCTGAAGGTTTCACAT

APHIS-S AGTTCCAGTGCCTCGGCACGCCGCAACATTTAAAGAATAAGTTCTCTGAAGGTTTCACAT

************************************************************

9A.10 TGACAATTAAAATTAAAGTGGACGACGAGACGAAGACTGTACGGCCTGAAGTCTGCGATG

9A.8 TGACAATTAAAATTAAAGTGGACGACGAGACGAAGACTGTACGGCCTGAAGTCTGTGATG

9A.13 TGACAATTAAAATTAAAGTGGACGACGAGACGAAGACTGTACGGCCTGAAGTCTGCGATG

9B.12 TGACAATTAAAATTAAAGTGGACGACGAGACGAAGACTGTACGGCCTGAAGTCTGCGATG

9A.9 TGACAATTAAAATTAAAGTGGACGACGAGACGAAGACTGTACGGCCTGAAGTCTGCGATG

9B.16 TGACAATTAAAATTAAAGTGGACGACGAGACGAAGACTGTACGGCCTGAAGTCTGCGATG

9B.14 TGACAATTAAAATTAAAGTGGACGACGAGACGAAGACTGTACGGCCTGAAGTCTGCGATG

APHIS-S TGACAATTAAAATTAAAGTGGACGACGAGACGAAGACTGTACGGCCTGAAGTCTGCGATG

******************************************************* ****

9A.10 CTGTGAAGCATTACGTCAGTACCAACTTCAGAGAGCCGAAGATTATGGAGGAGTACCAGG

9A.8 CTGTGAAGCATTACGTCAGTACCAACTTCAGAGAGCCGAAGATTATGGAGGAGTACCAGG

9A.13 CTGTGAAGCATTACGTCAGTACCAACTTCAGAGAGCCGAAGATTATGGAGGAGTACCAGG

9B.12 CTGTGAAGCATTACGTCAGTACCAACTTCAGAGAGCCGAAGATTATGGAGGAGTACCAGG

9A.9 CTGTGAAGCATTACGTCAGTACCAACTTCAGAGAGCCGAAGATTATGGAGGAGTACCAGG

9B.16 CTGTGAAGCATTACGTCAGTACCAACTTCAGAGAGCCGAAGATTATGGAGGAGTACCAGG

9B.14 CTGTGAAGCATTACGTCAGTACCAACTTCAGAGAGCCGAAGATTATGGAGGAGTACCAGG

APHIS-S CTGTGAAGCATTACGTCAGTACCAACTTCAGAGAGCCGAAGATTATGGAGGAGTACCAGG

************************************************************

9A.10 GTCTGTTAACATACTATTTGCCAGACAAGTCGGTGGCGTGGTCCAGAATGTTCGGCATAA

9A.8 GTCTGTTAACATACTATTTGCCAGACAAGTCGGTGGCGTGGTCCAGAATGTTCGGCATAA

9A.13 GTCTGTTAACATACTATTTGCCAGACAAGTCGGTGGCGTGGTCCAGAATGTTCGGCATAA

9B.12 GTCTGTTAACATACTATTTGCCAGACAAGTCGGTGGCGTGGTCCAGAATGTTCGGCATAA

9A.9 GTCTGTTAACATACTATTTGCCAGACAAGTCGGTGGCGTGGTCCAGAATGTTCGGCATAA

9B.16 GTCTGTTAACATACTATTTGCCAGACAAGTCGGTGGCGTGGTCCAGAATGTTCGGCATAA

9B.14 GTCTGTTAACATACTATTTGCCAGACAAGTCGGTGGCGTGGTCCAGAATGTTCGGCATAA

APHIS-S GTCTGTTAACATACTATTTGCCAGACAAGTCGGTGGCGTGGTCCAGAATGTTCGGCATAA

************************************************************

9A.10 TGGAGGCGGCCAAACGCGACCTCCCCGTCGAAGACTACAGCATATCACAAACTACCCTCG

9A.8 TGGAGGCGGCCAAACGCGACCTCCCCGTCGAAGACTACAGCATATCACAAACTACCCTCG

9A.13 TGGAGGCGGCCAAACGCGACCTCCCCGTCGAAGACTACAGCATATCACAAACTACCCTCG

9B.12 TGGAGGCGGCCAAACGCGACCTCCCCGTCGAAGACTACAGCATATCACAAACTACCCTCG

9A.9 TGGAGGCGGCCAAACGCGACCTCCCCGTCGAAGACTACAGCATATCACAAACTACCCTCG

9B.16 TGGAGGCGGCCAAACGCGACCTCCCCGTCGAAGACTACAGCATATCACAAACTACCCTCG

9B.14 TGGAGGCGGCCAAACGCGACCTCCCCGTCGAAGACTACAGCATATCACAAACTACCCTCG

APHIS-S TGGAGGCGGCCAAACGCGACCTCCCCGTCGAAGACTACAGCATATCACAAACTACCCTCG

************************************************************

9A.10 AG---------------------------------------------------

9A.8 AG---------------------------------------------------

9A.13 AG---------------------------------------------------

9B.12 AG---------------------------------------------------

9A.9 AG---------------------------------------------------

9B.16 AG---------------------------------------------------

9B.14 AG---------------------------------------------------

APHIS-S AGCAGATATTCCTACAGTTCACAAAGTATCAACATGAAGCACAACAGACATAA

**

**D**

10.22 ATGCGGGCGCGTGGAGAGCGGAAGGAGGCGGGCTCATGGGTGAAGTTTAGGCTGTTGATG

10.24 ATGCGGGCGCGTGGAGAGCGGAAGGAGGCGGGCTCATGGGTGAAGTTTAGGCTGTTGATG

10.29 ATGCGGGCGCGTGGAGAGCGGAAGGAGGCGGGCTCATGGGTGAAGTTTAGGCTGTTGATG

10.1 ATGCGGGCGCGTGGAGAGCGGAAGGAGGCGGGCTCATGGGTGAAGTTTAGGCTGTTGATG

10.21 ATGCGGGCGCGTGGAGAGCGGAAGGAGGCGGGCTCATGGGTGAAGTTTAGGCTGTTGATG

10.5 ATGCGGGCGCGTGGAGAGCGGAAGGAGGCGGGCTCATGGGTGAAGTTTAGGCTGTTGATG

10.11 ATGCGGGCGCGTGGAGAGCGGAAGGAGGCGGGCTCATGGGTGAAGTTTAGGCTGTTGATG

APHIS-S ATGCGGGCGCGTGGAGAGCGGAAGGAGGCGGGCTCATGGGTGAAGTTTAGGCTGTTGATG

10.19 ATGCGGGCGCGTGGAGAGCGGAAGGAGGCGGGCTCATGGGTGAAGTTTAGGCTGTTGATG

************************************************************

10.22 TGGAAGAACTTCGTGCAGCAGTTGAGGCACCCAGTGCAGACGGCGGCTGAGCTGCTGCTA

10.24 TGGAAGAACTTCGTGCAGCAGTTGAGGCACCCAGTGCAGACGGCGGCTGAGCTGCTGCTA

10.29 TGGAAGAACTTCGTGCAGCAGTTGAGGCACCCAGTGCAGACGGCGGCTGAGCTGCTGCTA

10.1 TGGAAGAACTTCGTGCAGCAGTTGAGGCACCCAGTGCAGACGGCGGCTGAGCTGCTGCTA

10.21 TGGAAGAACTTCGTGCAGCAGTTGAGGCACCCAGTGCAGACGGCGGCTGAGCTGCTGCTA

10.5 TGGAAGAACTTCGTGCAGCAGTTGAGGCACCCAGTGCAGACGGCGGCTGAGCTGCTGCTA

10.11 TGGAAGAACTTCGTGCAGCAGTTGAGGCACCCAGTGCAGACGGCGGCTGAGCTGCTGCTA

APHIS-S TGGAAGAACTTCGTGCAGCAGTTGAGGCACCCAGTGCAGACGGCGGCTGAGCTGCTGCTA

10.19 TGGAAGAACTTCGTGCAGCAGTTGAGGCACCCAGTGCAGACGGCGGCTGAGCTGCTGCTA

************************************************************

10.22 CCAGTCCTAACCATGAGCCTGGTCCTGGTGCTACGGTCACAGATCGACCCCGAAGTCTTG

10.24 CCAGTCCTAACCATGAGCCTGGTCCTGGTGCTACGGTCACAGATCGACCCCGAAGTCTTG

10.29 CCAGTCCTAACCATGAGCCTGGTCCTGGTGCTACGGTCACAGATCGACCCCGAAGTCTTG

10.1 CCAGTCCTAACCATGAGCCTGGTCCTGGTGCTACGGTCACAGATCGACCCCGAAGTCTTG

10.21 CCAGTCCTAACCATGAGCCTGGTCCTGGTGCTACGGTCACAGATCGACCCCGAAGTCTTG

10.5 CCAGTCCTAACCATGAGCCTGGTCCTGGTGCTACGGTCACAGATCGACCCCGAAGTCTTG

10.11 CCAGTCCTAACCATGAGCCTGGTCCTGGTGCTACGGTCACAGATCGACCCCGAAGTCTTG

APHIS-S CCAGTCCTAACCATGAGCCTGGTCCTGGTGCTACGGTCACAGATCGACCCCGAAGTCTTG

10.19 CCAGTCCTAACCATGAGCCTGGTCCTGGTGCTACGGTCACAGATCGACCCCGAAGTCTTG

************************************************************

10.22 GAAACCAGAACCTACCCGCCAATACCAGCCCACACTTTAAACTATTCCGTGACTGTTTTG

10.24 GAAACCAGAACCTACCCGCCAATACCAGCCCACACTTTAAACTATTCCGTGACTGTTTTG

10.29 GAAACCAGAACCTACCCGCCAATACCAGCCCACACTTTAAACTATTCCGTGACTGTTTTG

10.1 GAAACCAGAACCTACCCGCCAATACCAGCCCACACTTTAAACTATTCCGTGACTGTTTTG

10.21 GAAACCAGAACCTACCCGCCAATACCAGCCCACACTTTAAACTATTCCGTGACTGTTTTG

10.5 GAAACCAGAACCTACCCGCCAATACCAGCCCACACTTTAAACTATTCCGTGACTGTTTTG

10.11 GAAACCAGAACCTACCCGCCAATACCAGCCCACACTTTAAACTATTCCGTGACTGTTTTG

APHIS-S GAAACCAGAACCTACCCGCCAATACCAGCCCACACTTTAAACTATTCCGTGACTGTTTTG

10.19 GAAACCAGAACCTACCCGCCAATACCAGCCCACACTTTAAACTATTCCGTGACTGTTTTG

************************************************************

10.22 GGCGGAATGAATTTAACAAGAATGTCCATGGCATTCTCACCCGAGAATGCCGTATTGAGG

10.24 GGCGGAATGAATTTAACAAGAATGTCCATGGCATTCTCACCCGAGAATGCCGTATTGAGG

10.29 GGCGGAATGAATTTAACAAGAATGTCCATGGCATTCTCACCCGAGAATGCCGTATTGAGG

10.1 GGCGGAATGAATTTAACAAGAATGTCCATGGCATTCTCACCCGAGAATGCCGTATTGAGG

10.21 GGCGGAATGAATTTAACAAGAATGTCCATGGCATTCTCACCCGAGAATGCCGTATTGAGG

10.5 GGCGGAATGAATTTAACAAGAATGTCCATGGCATTCTCACCCGAGAATGCCGTATTGAGG

10.11 GGCGGAATGAATTTAACAAGAATGTCCATGGCATTCTCACCCGAGAATGCCGTATTGAGG

APHIS-S GGCGGAATGAATTTAACAAGAATGTCCATGGCATTCTCACCCGAGAATGCCGTATTGAGG

10.19 GGCGGAATGAATTTAACAAGAATGTCCATGGCATTCTCACCCGAGAATGCCGTATTGAGG

************************************************************

10.22 GACGTCGTATCCAGTGCTACAACAAAGTTACTGCTTAAAAACATGAGAGACCAAGTACTG

10.24 GACGTCGTATCCAGTGCTACAACAAAGTTACTGCTTAAAAACATGAGAGACCAAGTACTG

10.29 GACGTCGTATCCAGTGCTACAACAAAGTTACTGCTTAAAAACATGAGAGACCAAGTACTG

10.1 GACGTCGTATCCAGTGCTACAACAAAGTTACTGCTTAAAAACATGAGAGACCAAGTACTG

10.21 GACGTCGTATCCAGTGCTACAACAAAGTTACTGCTTAAAAACATGAGAGACCAAGTACTG

10.5 GACGTCGTATCCAGTGCTACAACAAAGTTACTGCTTAAAAACATGAGAGACCAAGTACTG

10.11 GACGTCGTATCCAGTGCTACAACAAAGTTACTGCTTAAAAACATGAGAGACCAAGTACTG

APHIS-S GACGTCGTATCCAGTGCTACAACAAAGTTACTGCTTAAAAACATGAGAGACCAAGTACTG

10.19 GACGTCGTATCCAGTGCTACAACAAAGTTACTGCTTAAAAACATGAGAGACCAAGTACTG

************************************************************

10.22 CCCATCATTGAGGCATTGCCAATAGAAATACCGCCGGGACTGGTAAACTCGTCACAGGTG

10.24 CCCATCATTGAGGCATTGCCAATAGAAATACCGCCGGGACTGGTAAACTCGTCACAGGTG

10.29 CCCATCATTGAGGCATTGCCAATAGAAATACCGCCGGGACTGGTAAACTCGTCACAGGTG

10.1 CCCATCATTGAGGCATTGCCAATAGAAATACCGCCGGGACTGGTAAACTCGTCACAGGTG

10.21 CCCATCATTGAGGCATTGCCAATAGAAATACCGCCGGGACTGGTAAACTCGTCACAGGTG

10.5 CCCATCATTGAGGCATTGCCAATAGAAATACCGCCGGGACTGGTAAACTCGTCACAGGTG

10.11 CCCATCATTGAGGCATTGCCAATAGAAATACCGCCGGGACTGGTAAACTCGTCACAGGTG

APHIS-S CCCATCATTGAGGCATTGCCAATAGAAATACCGCCGGGACTGGTAAACTCGTCACAGGTG

10.19 CCCATCATTGAGGCATTGCCAATAGAAATACCGCCGGGACTGGTAAACTCGTCACAGGTG

************************************************************

10.22 TACGAAATAGTTAAATTATTTGTCGACGAGAACGTTGTTACCGGATACAATAGCAGTGCG

10.24 TACGAAATAGTTAAATTATTTGTCGACGAGAACGTTGTTACCGGATACAATAGCAGTGCG

10.29 TACGAAATAGTTAAATTATTTGTCGACGAGAACGTTGTTACCGGATACAATAGCAGTGCG

10.1 TACGAAATAGTTAAATTATTTGTCGACGAGAACGTTGTTACCGGATACAATAGCAGTGCG

10.21 TACGAAATAGTTAAATTATTTGTCGACGAGAACGTTGTTACCGGATACAATAGCAGTGCG

10.5 TACGAAATAGTTAAATTATTTGTCGACGAGAACGTTGTTACCGGATACAATAGCAGTGCG

10.11 TACGAAATAGTTAAATTATTTGTCGACGAGAACGTTGTTACCGGATACAATAGCAGTGCG

APHIS-S TACGAAATAGTTAAATTATTTGTCGACGAGAACGTTGTTACCGGATACAATAGCAGTGCG

10.19 TACGAAATAGTTAAATTATTTGTCGACGAGAACGTTGTTACCGGATACAATAGCAGTGCG

************************************************************

10.22 GCAATGAGAGGAATATACGCAGAGGAAGAAGCCACGAGAAGGGTGATAGCTGGCATAGAA

10.24 GCAATGAGAGGAATATACGCAGAGGAAGAAGCCACGAGAAGGGTGATAGCTGGCATAGAA

10.29 GCAATGAGAGGAATATACGCAGAGGAAGAAGCCACGAGAAGGGTGATAGCTGGCATAGAA

10.1 GCAATGAGAGGAATATACGCAGAGGAAGAAGCCACGAGAAGGGTGATAGCTGGCATAGAA

10.21 GCAATGAGAGGAATATACGCAGAGGAAGAAGCCACGAGAAGGGTGATAGCTGGCATAGAA

10.5 GCAATGAGAGGAATATACGCAGAGGAAGAAGCCACGAGAAGGGTGATAGCTGGCATAGAA

10.11 GCAATGAGAGGAATATACGCAGAGGAAGAAGCCACGAGAAGGGTGATAGCTGGCATAGAA

APHIS-S GCAATGAGAGGAATATACGCAGAGGAAGAAGCCACGAGAAGGGTGATAGCTGGCATAGAA

10.19 GCAATGAGAGGAATATACGCAGAGGAAGAAGCCACGAGAAGGGTGATAGCTGGCATAGAA

************************************************************

10.22 TTCGATGACTCATTGCGTGAAATAACGGAGCTACCACTAGACTTGTCGTATGCGCTTCGT

10.24 TTCGATGACTCATTGCGT------------------------------------------

10.29 TTCGATGACTCATTGCGTGAAATAACGGAGCTACCACTAGACTTGTCGTATGCGCTTCGT

10.1 TTCGATGACTCATTGCGTGAAATAACGGAGCTACCACTAGACTTGTCGTATGCGCTTCGT

10.21 TTCGATGACTCATTGCGTGAAATAACGGAGCTACCACTAGACTTGTCGTATGCGCTTCGT

10.5 TTCGATGACTCATTGCGTGAAATAACGGAGCTACCACTAGACTTGTCGTATGCGCTTCGT

10.11 TTCGATGACTCATTGCGTGAAATAACGGAGCTACCACTAGACTTGTCGTATGCGCTTCGT

APHIS-S TTCGATGACTCATTGCGTGAAATAACGGAGCTACCACTAGACTTGTCGTATGCGCTTCGT

10.19 TTCGATGACTCATTGCGTGAAATAACGGAGCTACCACTAGACTTGTCGTATGCGCTTCGT

******************

10.22 TTTCCGGAGAGACCTCGCTTGAATTCCTTCTTCATGACAGGCGGTCGGACTTGGCGCACA

10.24 ------------------------------------------------------------

10.29 TTTCCGGAGAGACCTCGCTTGAATTCCTTCTTCATGACAGGCGGTCGGACTTGGCGCACA

10.1 TTTCCGGAGAGACCTCGCTTGAATTCCTTCTTCATGACAGGCGGTCGGACTTGGCGCACA

10.21 TTTCCGGAGAGACCTCGCTTGAATTCCTTCTTCATGACAGGCGGTCGGACTTGGCGCACA

10.5 TTTCCGGAGAGACCTCGCTTGAATTCCTTCTTCATGACAGGCGGTCGGACTTGGCGCACA

10.11 TTTCCGGAGAGACCTCGCTTGAATTCCTTCTTCATGACAGGCGGTCGGACTTGGCGCACA

APHIS-S TTTCCGGAGAGACCTCGCTTGAATTCCTTCTTCATGACAGGCGGTCGGACTTGGCGCACA

10.19 TTTCCGGAGAGACCTCGCTTGAATTCCTTCTTCATGACAGGCGGTCGGACTTGGCGCACA

10.22 GATAACGTGTTTCCTATGTTCGAAGTTCCCGGANCTCGCTTTCCGTATTCATGGGAAGGT

10.24 ------------------------------------------------------------

10.29 GATAACGTGTTTCCTATGTTCGAAGTTCCCGGACCTCGCTTTCCGTATTCATGGGAAGGT

10.1 GATAACGTGTTTCCTATGTTCGAAGTTCCCGGACCTCGCTTTCCGTATTCATGGGAAGGT

10.21 GATAACGTGTTTCCTATGTTCGAAGTTCCCGGACCTCGCTTTCCGTATTCATGGGAAGGT

10.5 GATAACGTGTTTCCTATGTTCGAAGTTCCCGGACCTCGCTTTCCGTATTCATGGGAAGGT

10.11 GATAACGTGTTTCCTATGTTCGAAGTTCCCGGACCTCGCTTTCCGTATTCATGGGAAGGT

APHIS-S GATAACGTGTTTCCTATGTTCGAAGTTCCCGGACCTCGCTTTCCGTATTCATGGGAAGGT

10.19 GATAACGTGTTTCCTATGTTCGAAGTTCCCGGACCTCGCTTTCCGTATTCATGGGAAGGT

10.22 GGAAATGATCCAGGATACGTAAACGAGATGTTCATAGCCTTGCAGCACATGATATCTTCA

10.24 ------------------------------------------------------------

10.29 GGAAATGATCCAGGATACGTAAACGAGATGTTCATAGCCTTGCAGCACATGATATCTTCA

10.1 GGAAATGATCCAGGATACGTAAACGAGATGTTCATAGCCTTGCAGCACATGATATCTTCA

10.21 GGAAATGATCCAGGATACGTAAACGAGATGTTCATAGCCTTGCAGCACATGATATCTTCA

10.5 GGAAATGATCCAGGATACGTAAACGAGATGTTCATAGCCTTGCAGCACATGATATCTTCA

10.11 GGAAATGATCCAGGATACGTAAACGAGATGTTCATAGCCTTGCAGCACATGATATCTTCA

APHIS-S GGAAATGATCCAGGATACGTAAACGAGATGTTCATAGCCTTGCAGCACATGATATCTTCA

10.19 GGAAATGATCCAGGATACGTAAACGAGATGTTCATAGCCTTGCAGCACATGATATCTTCA

10.22 GAACTGGTATCTAAAGTGGCGGGAGTGAACCTAGACTTCGATGTGCACATACAGAGGTAC

10.24 ------------------------------------------------------------

10.29 GAACTGGTATCTAAAGTGGCGGGAGTGAACCTAGACTTCGATGTGCACATACAGAGGTAC

10.1 GAACTGGTATCTAAAGTGGCGGGAGTGAACCTAGACTTCGATGTGCACATACAGAGGTAC

10.21 GAACTGGTATCTAAAGTGGCGGGAGTGAACCTAGACTTCGATGTGCACATACAGAGGTAC

10.5 GAACTGGTATCTAAAGTGGCGGGAGTGAACCTAGACTTCGATGTGCACATACAGAGGTAC

10.11 GAACTGGTATCTAAAGTGGCGGGAGTGAACCTAGACTTCGATGTGCACATACAGAGGTAC

APHIS-S GAACTGGTATCTAAAGTGGCGGGAGTGAACCTAGACTTCGATGTGCACATACAGAGGTAC

10.19 GAACTGGTATCTAAAGTGGCGGGAGTGAACCTAGACTTCGATGTGCACATACAGAGGTAC

10.22 CCACATCCAGCATACATCATGGACTTGGCGAAGGAAGCCCTGCAGTTCCTCTTCCCATCA

10.24 ------------------------------------------------------------

10.29 CCACATCCAGCATACATCATGGACTTGGCGAAGGAAGCCCTGCAGTTCCTCTTCCCATCA

10.1 CCACATCCAGCATACATCATGGACTTGGCGAAGGAAGCCCTGCAGTTCCTCTTCCCATCA

10.21 CCACATCCAGCATACATCATGGACTTGGCGAAGGAAGCCCTGCAGTTCCTCTTCCCATCA

10.5 CCACATCCAGCATACATCATGGACTTGGCGAAGGAAGCCCTGCAGTTCCTCTTCCCATCA

10.11 CCACATCCAGCATACATCATGGACTTGGCGAAGGAAGCCCTGCAGTTCCTCTTCCCATCA

APHIS-S CCACATCCAGCATACATCATGGACTTGGCGAAGGAAGCCCTGCAGTTCCTCTTCCCATCA

10.19 CCACATCCAGCATACATCATGGACTTGGCGAAGGAAGCCCTGCAGTTCCTCTTCCCATCA

10.22 TTCATCATGATCAGCTTCAGTTACACCGCTATCAATATTATACGATCCGTGACCGTGGAA

10.24 ------------------------------------------------------------

10.29 TTCATCATGATCAGCTTCAGTTACACCGCTATCAATATTATACGATCCGTGACCGTGGAA

10.1 TTCATCATGATCAGCTTCAGTTACACCGCTATCAATATTATACGATCCGTGACCGTGGAA

10.21 TTCATCATGATCAGCTTCAGTTACACCGCTATCAATATTATACGATCCGTGACCGTGGAA

10.5 TTCATCATGATCAGCTTCAGTTACACCGCTATCAATATTATACGATCCGTGACCGTGGAA

10.11 TTCATCATGATCAGCTTCAGTTACACCGCTATCAATATTATACGATCCGTGACCGTGGAA

APHIS-S TTCATCATGATCAGCTTCAGTTACACCGCTATCAATATTATACGATCCGTGACCGTGGAA

10.19 TTCATCATGATCAGCTTCAGTTACACCGCTATCAATATTATACGATCCGTGACCGTGGAA

10.22 AAAGAAATGCAATTGAAGGAAACGATGAAGATCATGGGACTCCCAACGTGGCTGCATTGG

10.24 ------------------------------------------------------------

10.29 AAAGAAATGCAATTGAAGGAAACGATGAAGATCATGGGACTCCCAACGTGGCTGCATTGG

10.1 AAAGAAATGCAATTGAAGGAAACGATGAAGATCATGGGACTCCCAACGTGGCTGCATTGG

10.21 AAAGAAATGCAATTGAAGGAAACGATGAAGATCATGGGACTCCCAACGTGGCTGCATTGG

10.5 AAAGAAATGCAATTGAAGGAAACGATGAAGATCATGGGACTCCCAACGTGGCTGCATTGG

10.11 AAAGAAATGCAATTGAAGGAAACGATGAAGATCATGGGACTCCCAACGTGGCTGCATTGG

APHIS-S AAAGAAATGCAATTGAAGGAAACGATGAAGATCATGGGACTCCCAACGTGGCTGCATTGG

10.19 AAAGAAATGCAATTGAAGGAAACGATGAAGATCATGGGACTCCCAACGTGGCTGCATTGG

10.22 ------------------------------------------------------------

10.24 ------------------------------------------------------------

10.29 ATGGCATGGTTTTTTAAACAATTTATTTATTTGCTGATTGCTTCGGTTTTGATACTTGTT

10.1 ------------------------------------------------------------

10.21 ------------------------------------------------------------

10.5 ATGGCATGGTTTTTTAAACAATTTATTTATTTGCTGATTGCTTCGGTTTTGATACTTGTT

10.11 ATGGCATGGTTTTTTAAACAATTTATTTATTTGCTGATTGCTTCGGTTTTGATACTTGTT

APHIS-S ATGGCATGGTTTTTTAAACAATTTATTTATTTGCTGATTGCTTCGGTTTTGATACTTGTT

10.19 ATGGCATGGTTTTTTAAACAATTTATTTATTTGCTGATTGCTTCGGTTTTGATACTTGTT

10.22 ------------------------------------------------------------

10.24 ------------------------------------------------------------

10.29 ATATTAAAGGTAAATTGGTTTACTACAGAAGAAGGCTTTAGCGACTATGCCGTATTCACT

10.1 ------------------------------------------------------------

10.21 ------------------------------------------------------------

10.5 ATATTAAAG---------------------------------------------------

10.11 ATATTAAAG---------------------------------------------------

APHIS-S ATATTAAAGGTAAATTGGTTTACTACAGAAGAAGGCTTTAGCGACTATGCCGTATTCACT

10.19 ATATTAAAGGTAAATTGGTTTACTACAGAAGAAGGCTTTAGCGACTATGCCGTATTCACT

10.22 ------------------------------------------------------------

10.24 ------------------------------------------------------------

10.29 AATACACCTTGGACCGTCCTCTTCTTCTTCCTAACACTGTATCTTACGTGTACCATATTT

10.1 ------------------------------------------------------------

10.21 ------------------------------------------------------------

10.5 ------------------------------------------------------------

10.11 ------------------------------------------------------------

APHIS-S AATACACCTTGGACCGTCCTCTTCTTCTTCCTAACACTGTATCTTACGTGTACCATATTT

10.19 AATACACCTTGGACCGTCCTCTTCTTCTTCCTAACACTGTATCTTACGTGTACCATATTT

10.22 ------------------------------------------------------------

10.24 ------------------------------------------------------------

10.29 TTCTGTTTCATGATAAGTGGTTTCTTTTCAAAAGCCAGTACAGCGGCGTTGTTTGGTGGG

10.1 ------------------------------------------------------------

10.21 ------------------------------------------------------------

10.5 ----------------------------------CCAGTACAGCGGCGTTGTTTGGTGGG

10.11 ----------------------------------CCAGTACAGCGGCGTTGTTTGGTGGG

APHIS-S TTCTGTTTCATGATAAGTGGTTTCTTTTCAAAAGCCAGTACAGCGGCGTTGTTTGGTGGG

10.19 TTCTGTTTCATGATAAGTGGTTTCTTTTCAAAAGCCAGTACAGCGGCGTTGTTTGGTGGG

10.22 ------------------------------------------------------------

10.24 ------------------------------------------------------------

10.29 GTGATCTGGTTTCTGACGTATATCCCCGCATTCCTCCTGGCTATGGACGTGAACATGTCT

10.1 ------------------------------------------------------------

10.21 ------------------------------------------------------------

10.5 GTGATCTGGTTTCTGACGTATATCCCCGCATTCCTCCTGGCTATGGACGTGAACATGTCT

10.11 GTGATCTGGTTTCTGACGTATATCCCCGCATTCCTCCTGGCTATGGACGTGAACATGTCT

APHIS-S GTGATCTGGTTTCTGACGTATATCCCCGCATTCCTCCTGGCTATGGACGTGAACATGTCT

10.19 GTGATCTGGTTTCTGACGTATATCCCCGCATTCCTCCTGGCTATGGACGTGAACATGTCT

10.22 ------------------------------------------------------------

10.24 ------------------------------------------------------------

10.29 ACCTCTCTACAAGCGGTCACCTGCCTAATGCTCAACTCCGCCATGTCTTACGGCTTCCAG

10.1 ------------------------------------------------------------

10.21 ------------------------------------------------------------

10.5 ACCTCTCTACAAGCGGTCACCTGCCTAATGCTCAACTCCGCCATGTCTTACGGCTTCCAG

10.11 ACCTCTCTACAAGCGGTCACCTGCCTAATGCTCAACTCCGCCATGTCTTACGGCTTCCAG

APHIS-S ACCTCTCTACAAGCGGTCACCTGCCTAATGCTCAACTCCGCCATGTCTTACGGCTTCCAG

10.19 ACCTCTCTACAAGCGGTCACCTGCCTAATGCTCAACTCCGCCATGTCTTACGGCTTCCAG

10.22 ------------------------------------------------------------

10.24 ------------------------------------------------------------

10.29 CTGTTACTGGCCCGGGAAAGTACCGGAGGAATGCAGTGGGGTGATTTTATGACGTCACCA

10.1 ------------------------------------------------------------

10.21 ------------------------------------------------------------

10.5 CTGTTACTGGCCCGGGAAAGTACCGGAGGAATGCAGTGGGGTGATTTTATGACGTCACCA

10.11 CTGTTACTGGCCCGGGAAAGTACCGGAGGAATGCAGTGGGGTGATTTTATGACGTCACCA

APHIS-S CTGTTACTGGCCCGGGAAAGTACCGGAGGAATGCAGTGGGGTGATTTTATGACGTCACCA

10.19 CTGTTACTGGCCCGGGAAAGTACCGGAGGAATGCAGTGGGGTGATTTTATGACGTCACCA

10.22 ------------------------------------------------------------

10.24 ------------------------------------------------------------

10.29 GCAACGGACTCGTCACGATTCGTATTCGGTCACGTCGTTATAATGATGGCTTTGAACT-G

10.1 ------------------------------------------------------------

10.21 ------------------------------------------------------------

10.5 GCAACGGACTCGTCACGATTCGTATTCGGTCACGTCGTTATAATGATGGCTTTGAACTGG

10.11 GCAACGGACTCGTCACGATTCGTATTCGGTCACGTCGTTATAATGATGGCTTTGAACTGG

APHIS-S GCAACGGACTCGTCACGATTCGTATTCGGTCACGTCGTTATAATGATGGCTTTGAACT-G

10.19 GCAACGGACTCGTCACGATTCGTATTCGGTCACGTCGTTATAATGATGGCTTTGAACT-G

10.22 ------------------------------------------------------------

10.24 ------------------------------------------------------------

10.29 TGTGCTCTACATGTTGATTGCCCTATATCTAGAGCAAGTACTACCCGGGCCGTATGGCAC

10.1 ------------------------------------------------------------

10.21 ------------------------------------------------------------

10.5 TGTGCTCTACATGTTGATTGCCCTATATCTAGAGCAAGTACTACCCGGGCCGTATGGCAC

10.11 TGTGCTCTACATGTTGATTGCCCTATATCTAGAGCAAGTACTACCCGGGCCGTATGGCAC

APHIS-S TGTGCTCTACATGTTGATTGCCCTATATCTAGAGCAAGTACTACCCGGGCCGTATGGCAC

10.19 TGTGCTCTACATGTTGATTGCCCTATATCTAGAGCAAGTACTACCCGGGCCGTATGGCAC

10.22 ------------------------------------------------------------

10.24 ------------------------------------------------------------

10.29 ACCGAAGCCCTGGTATTTCTTCGTCCAAAGACAGTTCTGGTGTAGCAGCAAAACTACTCA

10.1 ------------------------------------------------------------

10.21 ------------------------------------------------------------

10.5 ACCGAAGCCCTGGTATTTCTTCGTCCAAAGACAGTTCTGGTGTAGCAGCAAAACTACTCA

10.11 ACCGAAGCCCTGGTATTTCTTCGTCCAAAGACAGTTCTGGTGTAGCAGCAAAACTACTCA

APHIS-S ACCGAAGCCCTGGTATTTCTTCGTCCAAAGACAGTTCTGGTGTAGCAGCAAAACTACTCA

10.19 ACCGAAGCCCTGGTATTTCTTCGTCCAAAGACAGTTCTGGTGTAGCAGCAAAACTACTCA

10.22 ------------------------------------------------------------

10.24 ------------------------------------------------------------

10.29 TGATATCGGTACAGACAACAGCGACACATCAAGTTTAACAAAAGAAAGCGACCCTACAGA

10.1 ------------------------------------------------------------

10.21 ------------------------------------------------------------

10.5 TGATATCGGTACAGACAACAGCGACACATCAAGTTTAACAAAAGAAAGCGACCCTACAGA

10.11 TGATATCGGTACAGACAACAGCGACACATCAAGTTTAACAAAAGAAAGCGACCCTACAGA

APHIS-S TGATATCGGTACAGACAACAGCGACACATCAAGTTTAACAAAAGAAAGCGACCCTACAGA

10.19 TGATATCGGTACAGACAACAGCGACACATCAAGTTTAACAAAAGAAAGCGACCCTACAGA

10.22 ------------------------------------------------------------

10.24 ------------------------------------------------------------

10.29 CCTTCCGATTGGAGTTAAAATACAAAACCTTAAAAAGGTTTACGGGAGCAACGTTGCGGT

10.1 ------------------------------------------------------------

10.21 ------------------------------------------------------------

10.5 CCTTCCGATTGGAGTTAAAATACAAAACCTTAAAAAGGTTTACGGGAGCAACGTTGCGGT

10.11 CCTTCCGATTGGAGTTAAAATACAAAACCTTAAAAAGGTTTACGGGAGCAACGTTGCGGT

APHIS-S CCTTCCGATTGGAGTTAAAATACAAAACCTTAAAAAGGTTTACGGGAGCAACGTTGCGGT

10.19 CCTTCCGATTGGAGTTAAAATACAAAACCTTAAAAAGGTTTACGGGAGCAACGTTGCGGT

10.22 ------------------------------------------------------------

10.24 ------------------------------------------------------------

10.29 AAACAATTTATCCCTCAACATTTACGACGACCAAATCACGGCTCTACTTGGACACAACGG

10.1 ------------------------------------------------------------

10.21 ------------------------------------------------------------

10.5 AAACAATTTATCCCTCAACATTTACGACGACCAAATCACGGTTCTACTTGGACACAACGG

10.11 AAACAATTTATCCCTCAACATTTACGACGACCAAATCACGGTTCTACTTGGACACAACGG

APHIS-S AAACAATTTATCCCTCAACATTTACGACGACCAAATCACGGTTCTACTTGGACACAACGG

10.19 AAACAATTTATCCCT---------------------------------------------

10.22 ------------------------------------------------------------

10.24 ------------------------------------------------------------

10.29 AGCGGGAAAATCCACAACCATTTCAATGCTCACAGGTAACGTGGACATAACCAGCGGGTC

10.1 ------------------------------------------------------------

10.21 ------------------------------------------------------------

10.5 AGCGGGAAAATCCACAACCATTTCAATGCTCACAGGTAACGTGGACATAACCAGCGGGTC

10.11 AGCGGGAAAATCCACAACCATTTCAATGCTCACAGGTAACGTGGACATAACCAGCGGGTC

APHIS-S AGCGGGAAAATCCACAACCATTTCAATGCTCACAGGTAACGTGGACATAACCAGCGGGTC

10.19 ------------------------------------------------------------

10.22 ------------------------------------------------------------

10.24 ------------------------------------------------------------

10.29 GGTGACGGTGGCTGGCTACGACATAGAAAAACAAACAAGTTCAGCACGCTCACACATTGG

10.1 ------------------------------------------------------------

10.21 ------------------------------------------------------------

10.5 GGTGACGGTGGCTGGCTACGACATAGAAAAACAAACAAGTTCAGCACGCTCACACATTGG

10.11 GGTGACGGTGGCTGGCTACGACATAGAAAAACAAACAAGTTCAGCACGCTCACACATTGG

APHIS-S GGTGACGGTGGCTGGCTACGACATAGAAAAACAAACAAGTTCAGCACGCTCACACATTGG

10.19 ------------------------------------------------------------

10.22 ------------------------------------------------------------

10.24 ------------------------------------------------------------

10.29 ACTCTGCCCTCAACATAACGTACTCTTCAACGAACTCACAGTCAAAGAACATTTACAGTT

10.1 ------------------------------------------------------------

10.21 ------------------------------------------------------------

10.5 ACTCTGCCCTCAACATAACGTACTCTTCAACGAACTCACAGTCAAAGAACATTTACAGTT

10.11 ACTCTGCCCTCAACATAACGTACTCTTCAACGAACTCACAGTCAAAGAACATTTACAGTT

APHIS-S ACTCTGCCCTCAACATAACGTACTCTTCAACGAACTCACAGTCAAAGAACATTTACAGTT

10.19 ------------------------------------------------------------

10.22 ------------------------------------------------------------

10.24 ------------------------------------------------------------

10.29 CTTCTCTCGTCTGAAAGGCTTCAGCGGTAAAGAGTTGGATGAAGAAATTGAGACGCTTAT

10.1 ------------------------------------------------------------

10.21 ------------------------------------------------------------

10.5 CTTCTCTCGTCTGAAAGGCTTCAGCGGTAAAGAGTTGGATGAAGAAATTGAGACGCTTAT

10.11 CTTCTCTCGTCTGAAAGGCTTCAGCGGTAAAGAGTTGGATGAAGAAATTGAGACGCTTAT

APHIS-S CTTCTCTCGTCTGAAAGGCTTCAGCGGTAAAGAGTTGGATGAAGAAATTGAGACGCTTAT

10.19 ------------------------------------------------------------

10.22 ------------------------------------------------------------

10.24 ------------------------------------------------------------

10.29 TGAAAAATTGGAATTGCAAGAAAAGAGGGATTACCAATCAGCGGGGTTATCAGGGGGACA

10.1 ------------------------------------------------------------

10.21 ------------------------------------------------------------

10.5 TGAAAAATTGGAATTGCAAGAAAAGAGGGATTACCAATCAGCGGGGTTATCAGGGGGACA

10.11 TGAAAAATTGGAATTGCAAGAAAAGAGGGATTACCAATCAGCGGGGTTATCAGGGGGACA

APHIS-S TGAAAAATTGGAATTGCAAGAAAAGAGGGATTACCAATCAGCGGGGTTATCAGGGGGACA

10.19 ------------------------------------------------------------

10.22 ------------------------------------------------------------

10.24 ------------------------------------------------------------

10.29 GAAGCGACGATTAGGAGTGGGCGTCGCGCTATGCGGGGCGGCTAAAGTGGTTCTACTGGA

10.1 ------------------------------------------------------------

10.21 ------------------------------------------------------------

10.5 GAAGCGACGATTAGGAGTGGGCGTCGCGCTATGCGGGGCGGCTAAAGTGGTTCTACTGGA

10.11 GAAGCGACGATTAGGAGTGGGCGTCGCGCTATGCGGGGCGGCTAAAGTGGTTCTACTGGA

APHIS-S GAAGCGACGATTAGGAGTGGGCGTCGCGCTATGCGGGGCGGCTAAAGTGGTTCTACTGGA

10.19 ------------------------------------------------------------

10.22 ------------------------------------------------------------

10.24 ------------------------------------------------------------

10.29 CGAGCCCACTTCTGGCATGGACCCGGCCTCACGTCGTGCCCTATGGGACTTGTTGCAGAG

10.1 ------------------------------------------------------------

10.21 ------------------------------------------------------------

10.5 CGAGCCCACTTCTGGCATGGACCCGGCCTCACGTCGTGCCCTATGGGACTTGTTGCAGAG

10.11 CGAGCCCACTTCTGGCATGGACCCGGCCTCACGTCGTGCCCTATGGGACTTGTTGCAGAG

APHIS-S CGAGCCCACTTCTGGCATGGACCCGGCCTCACGTCGTGCCCTATGGGACTTGTTGCAGAG

10.19 ------------------------------------------------------------

10.22 ------------------------------------------------------------

10.24 ------------------------------------------------------------

10.29 AGAGAAGAAAGGTCGATCGATGATCCTGACGACACACTTCATGGACGAAGCGGACATATT

10.1 ------------------------------------------------------------

10.21 ------------------------------------------------------------

10.5 AGAGAAGAAAGGTCGATCGATGATCCTGACGACACACTTCATGGACGAAGCGGACATATT

10.11 AGAGAAGAAAGGTCGATCGATGATCCTGACGACACACTTCATGGACGAAGCGGACATATT

APHIS-S AGAGAAGAAAGGTCGATCGATGATCCTGACGACACACTTCATGGACGAAGCGGACATATT

10.19 ------------------------------------------------------------

10.22 ------------------------------------------------------------

10.24 ------------------------------------------------------------

10.29 AGGGGATAGAGTTGCCATTATGGCGGACGGTCGTCTCCAGTGCGTGGGCTCACCTTACTT

10.1 ------------------------------------------------------------

10.21 ------------------------------------------------------------

10.5 AGGGGATAGAGTTGCCATTATGGCGGACGGTCGTCTCCAGTGCGTGGGCTCACCTTACTT

10.11 AGGGGATAGAGTTGCCATTATGGCGGACGGTCGTCTCCAGTGCGTGGGCTCACCTTACTT

APHIS-S AGGGGATAGAGTTGCCATTATGGCGGACGGTCGTCTCCAGTGCGTGGGCTCACCTTACTT

10.19 ------------------------------------------------------------

10.22 ------------------------------------------------------------

10.24 ------------------------------------------------------------

10.29 CCTCAAGAGACACTATGGAGTCGGCTACACGCTAGTTGTGGTCAAGAAGGAAGATTTCCG

10.1 ------------------------------------------------------------

10.21 ------------------------------------------------------------

10.5 CCTCAAGAGACACTATGGAGTCGGCTACACGCTAGTTGTGGTCAAGAAGGAAGATTTCCG

10.11 CCTCAAGAGACACTATGGAGTCGGCTACACGCTAGTTGTGGTCAAGAAGGAAGATTTCCG

APHIS-S CCTCAAGAGACACTATGGAGTCGGCTACACGCTAGTTGTGGTCAAGAAGGAAGATTTCCG

10.19 ------------------------------------------------------------

10.22 ------------------------------------------------------------

10.24 ------------------------------------------------------------

10.29 ACTGGACACCTGCACAGAGCTGATCAATAGATACATCCCTGGAACTGTTGTGAAGGAAGA

10.1 ------------------------------------------------------------

10.21 ------------------------------------------------------------

10.5 ACTGGACACCTGCACAGAGCTGATCAATAGATACATCCCTGGAACTGTTGTGAAGGAAGA

10.11 ACTGGACACCTGCACAGAGCTGATCAATAGATACATCCCTGGAACTGTTGTGAAGGAAGA

APHIS-S ACTGGACACCTGCACAGAGCTGATCAATAGATACATCCCTGGAACTGTTGTGAAGGAAGA

10.19 ------------------------------------------------------------

10.22 ------------------------------------------------------------

10.24 ------------------------------------------------------------

10.29 CCGAGGCACTGAAGTGACATATAGCATGACTAATGAGTATTCGCACGTGTTTGAATCTAT

10.1 ------------------------------------------------------------

10.21 ------------------------------------------------------------

10.5 CCGAGGCACTGAAGTGACATATAGCATGACTAATGAGTATTCGCACGTGTTTGAATCTAT

10.11 CCGAGGCACTGAAGTGACATATAGCATGACTAATGAGTATTCGCACGTGTTTGAATCTAT

APHIS-S CCGAGGCACTGAAGTGACATATAGCATGACTAATGAGTATTCGCACGTGTTTGAATCTAT

10.19 ------------------------------------------------------------

10.22 ------------------------------------------------------------

10.24 ------------------------------------------------------------

10.29 GCTGCGCGATTTGGAGGCAAAGGCCGATGAGATAAACTTTAAAAACTACGGCCTACTGGC

10.1 ------------------------------------------------------------

10.21 ------------------------------------------------------------

10.5 GCTGCGCGATTTGGAGGCAAAGGCCGATGAGATAAACTTTAAAAACTACGGCCTACTGGC

10.11 GCTGCGCGATTTGGAGGCAAAGGCCGATGAGATAAACTTTAAAAACTACGGCCTACTGGC

APHIS-S GCTGCGCGATTTGGAGGCAAAGGCCGATGAGATAAACTTTAAAAACTACGGCCTACTGGC

10.19 ------------------------------------------------------------

10.22 ------------------------------------------------------------

10.24 ------------------------------------------------------------

10.29 TACTACATTAGAAGATGTGTTCATGTCCGTGGGCACAGATGTGGTCGCAACTTCAGATGT

10.1 ------------------------------------------------------------

10.21 ------------------------------------------------------------

10.5 TACTACATTAGAAGATGTGTTCATGTCCGTGGGCACAGATGTGGTCGCAACTTCAGATGT

10.11 TACTACATTAGAAGATGTGTTCATGTCCGTGGGCACAGATGTGGTCGCAACTTCAGATGT

APHIS-S TACTACATTAGAAGATGTGTTCATGTCCGTGGGCACAGATGTGGTCGCAACTTCAGATGT

10.19 ------------------------------------------------------------

10.22 ------------------------------------------------------------

10.24 ------------------------------------------------------------

10.29 GGACGACAATACAACCGTTTCATCTAGTGCTGATACTCTAGCATTTGAATATGATTCTTT

10.1 ------------------------------------------------------------

10.21 ------------------------------------------------------------

10.5 GGACGACAATACAACCGTTTCATCTAGTGCTGATACTCTAGCATTTGAATATGATTCTTT

10.11 GGACGACAATACAACCGTTTCATCTAGTGCTGATACTCTAGCATTTGAATATGATTCTTT

APHIS-S GGACGACAATACAACCGTTTCATCTAGTGCTGATACTCTAGCATTTGAATATGATTCTTT

10.19 ------------------------------------------------------------

10.22 ------------------------------------------------------------

10.24 ------------------------------------------------------------

10.29 AGAAAAATTGGACGGGACTGGCTATGGGGATGAAAAAGGGATCCGATTAATTTGCCAACA

10.1 ------------------------------------------------------------

10.21 ------------------------------------------------------------

10.5 AGAAAAATTGGACGGGACTGGCTATGGGGATGAAAAAGGGATCCGATTAATTTGCCAACA

10.11 AGAAAAATTGGACGGGACTGGCTATGGGGATGAAAAAGGGATCCGATTAATTTGCCAACA

APHIS-S AGAAAAATTGGACGGGACTGGCTATGGGGATGAAAAAGGGATCCGATTAATTTGCCAACA

10.19 ------------------------------------------------------------

10.22 ------------------------------------------------------------

10.24 ------------------------------------------------------------

10.29 CGTGGTAGCAATATGGATGAAACTGTTTCTGGTGCTGACAAGGTCTTGGCTTATCCTGTT

10.1 ------------------------------------------------------------

10.21 ------------------------------------------------------------

10.5 CGTGGTAGCAATATGGATGAAACTGTTTCTGGTGCTGACAAGGTCTTGGCTTATCCTGTT

10.11 CGTGGTAGCAATATGGATGAAACTGTTTCTGGTGCTGACAAGGTCTTGGCTTATCCTGTT

APHIS-S CGTGGTAGCAATATGGATGAAACTGTTTCTGGTGCTGACAAGGTCTTGGCTTATCCTGTT

10.19 ------------------------------------------------------------

10.22 ------------------------------------------------------------

10.24 ------------------------------------------------------------

10.29 GCTCCAAGTATTGGTGTCCTTGGTACAAATCATTGCCACACTCGGAGTCATGCAGTATGT

10.1 ------------------------------------------------------------

10.21 ------------------------------------------------------------

10.5 GCTCCAAGTATTGGTGTCCTTGGTACAAATCATTGCCACACTCGGAGTCATGCAGTATGT

10.11 GCTCCAAGTATTGGTGTCCTTGGTACAAATCATTGCCACACTCGGAGTCATGCAGTATGT

APHIS-S GCTCCAAGTATTGGTGTCCTTGGTACAAATCATTGCCACACTCGGAGTCATGCAGTATGT

10.19 ------------------------------------------------------------

10.22 ------------------------------------------------------------

10.24 ------------------------------------------------------------

10.29 CATCTCTATGACCGAGCATATACAAAGAAGAGAACTTTCATTGGCTGAAGGTTTCGCAGG

10.1 ------------------------------------------------------------

10.21 ------------------------------------------------------------

10.5 CATCTCTATGACCGAGCATATACAAAGAAGAGAACTTTCATTGGCTGAAGGTTTCGCAGG

10.11 CATCTCTATGACCGAGCATATACAAAGAAGAGAACTTTCATTGGCTGAAGGTTTCGCAGG

APHIS-S CATCTCTATGACCGAGCATATACAAAGAAGAGAACTTTCATTGGCTGAAGGTTTCGCAGG

10.19 ------------------------------------------------------------

10.22 ------------------------------------------------------------

10.24 ------------------------------------------------------------

10.29 CACAGAAACATTAGTTAGTTTCAAAGGGTTGTCCCCTACATCGACAGGTTCGCTAGCGAA

10.1 ------------------------------------------------------------

10.21 ------------------------------------------------------------

10.5 CACAGAAACATTAGTTAGTTTCAAAGGGTTGTCCCCTACATCGACAGGTTCGCTAGCGAA

10.11 CACAGAAACATTAGTTAGTTTCAAAGGGTTGTCCCCTACATCGACAGGTTCGCTAGCGAA

APHIS-S CACAGAAACATTAGTTAGTTTCAAAGGGTTGTCCCCTACATCGACAGGTTCGCTAGCGAA

10.19 ------------------------------------------------------------

10.22 ------------------------------------------------------------

10.24 ------------------------------------------------------------

10.29 GGCTGCCTACGAGTCGATATTTGTAACCGCCAATAATCCCACAATGGAAATCACTGTTGT

10.1 ------------------------------------------------------------

10.21 ------------------------------------------------------------

10.5 GGCTGCCTACGAGTCGATATTTGTAACCGCCAATAATCCCACAATGGAAATCACTGTTGT

10.11 GGCTGCCTACGAGTCGATATTTGTAACCGCCAATAATCCCACAATGGAAATCACTGTTGT

APHIS-S GGCTGCCTACGAGTCGATATTTGTAACCGCCAATAATCCCACAATGGAAATCACTGTTGT

10.19 ------------------------------------------------------------

10.22 ------------------------------------------------------------

10.24 ------------------------------------------------------------

10.29 TGATAATACACCTATAGATGAATATTATTTGGAAAGAACAGATGACGTATCAGCGATGGC

10.1 ------------------------------------------------------------

10.21 ------------------------------------------------------------

10.5 TGATAATACACCTATAGATGAATATTATTTGGAAAGAACAGATGACGTATCAGCGATGGC

10.11 TGATAATACACCTATAGATGAATATTATTTGGAAAGAACAGATGACGTATCAGCGATGGC

APHIS-S TGATAATACACCTATAGATGAATATTATTTGGAAAGAACAGATGACGTATCAGCGATGGC

10.19 ------------------------------------------------------------

10.22 ------------------------------------------------------------

10.24 ------------------------------------------------------------

10.29 GGTGCTCCGGCACAGTCTGTTGATCGGCGCGACGTTCGACGACCACTCCGCGACCGCGTG

10.1 ------------------------------------------------------------

10.21 ------------------------------------------------------------

10.5 GGTGCTCCGGCACAGTCTGTTGATCGGCGCGACGTTCGACGACCACTCCGCGACCGCGTG

10.11 GGTGCTCCGGCACAGTCTGTTGATCGGCGCGACGTTCGACGACCACTCCGCGACCGCGTG

APHIS-S GGTGCTCCGGCACAGTCTGTTGATCGGCGCGACGTTCGACGACCACTCCGCGACCGCGTG

10.19 ------------------------------------------------------------

10.22 ------------------------------------------------------------

10.24 ------------------------------------------------------------

10.29 GTTCAGCAACTTCGGTTACCACGACGTGGCCATGTCACTGGCTGCTGTGCACGCCGCCTT

10.1 ------------------------------------------------------------

10.21 ------------------------------------------------------------

10.5 GTTCAGCAACTTCGGTTACCACGACGTGGCCATGTCACTGGCTGCTGTGCACGCCGCCTT

10.11 GTTCAGCAACTTCGGTTACCACGACGTGGCCATGTCACTGGCTGCTGTGCACGCCGCCTT

APHIS-S GTTCAGCAACTTCGGTTACCACGACGTGGCCATGTCACTGGCTGCTGTGCACGCCGCCTT

10.19 ------------------------------------------------------------

10.22 ------------------------------------------------------------

10.24 ------------------------------------------------------------

10.29 GCTCAGAGCTGTCAATCCTGCAGCCAACTTGACTGTTTACAACCACCCACTTGAGGCCAA

10.1 ------------------------------------------------------------

10.21 ------------------------------------------------------------

10.5 GCTCAGAGCTGTCAATCCTGCAGCCAACTTGACTGTTTACAACCACCCACTTGAGGCCAA

10.11 GCTCAGAGCTGTCAATCCTGCAGCCAACTTGACTGTTTACAACCACCCACTTGAGGCCAA

APHIS-S GCTCAGAGCTGTCAATCCTGCAGCCAACTTGACTGTTTACAACCACCCACTTGAGGCCAA

10.19 ------------------------------------------------------------

10.22 ------------------------------------------------------------

10.24 ------------------------------------------------------------

10.29 TTATGTCAACCAG-----------------------------------------------

10.1 ------------------------------------------------------------

10.21 ------------------------------------------------------------

10.5 TTATGTCAACCAGAACGACATGCAAACAATGGTAGCGTTCCTCTCGATGCAACTTGCGTC

10.11 TTATGTCAACCAGAACGACATGCAAACAATGGTAGCGTTCCTCTCGATGCAACTTGCGTC

APHIS-S TTATGTCAACCAGAACGACATGCAAACAATGGTAGCGTTCCTCTCGATGCAACTTGCGTC

10.19 ------------------------------------------------------------

10.22 ------------------------------------------------------------

10.24 ------------------------------------------------------------

10.29 ------------------------------------------------------------

10.1 ------------------------------------------------------------

10.21 ------------------------------------------------------------

10.5 GGGCATCGGCAGCAGTCTGTCAATTGTCAGTGCTGTGTTCATCATGTTCTATATCAAGG-

10.11 GGGCATCGGCAGCAGTCTGTCAATTGTCAGTGCTGTGTTCATCATGTTCTATATCAAGG-

APHIS-S GGGCATCGGCAGCAGTCTGTCAATTGTCAGTGCTGTGTTCATCATGTTCTATATCAAGGA

10.19 ------------------------------------------------------------

10.22 ------------------------------------------------------------

10.24 ------------------------------------------------------------

10.29 ------------------------------------------------------------

10.1 ------------------------------------------------------------

10.21 ------------------------------------------------------------

10.5 ------------------------------------------------------------

10.11 ------------------------------------------------------------

APHIS-S GCGAGTATCTCGCGCCAAGCTGCTGCAGAAGGCGGCAGGCATCCAGCCGTTAGTGATGTG

10.19 ------------------------------------------------------------

10.22 ------------------------------------------------------------

10.24 ------------------------------------------------------------

10.29 ------------------------------------------------------------

10.1 ------------------------------------------------------------

10.21 ------------------------------------------------------------

10.5 ------------------------------------------------------------

10.11 ------------------------------------------------------------

APHIS-S GCTCAGCGCCGCCGTGTTCGACTGGATCTGGTTCTGCGTCATCGCCGTCGGCATCGTTAT

10.19 ------------------------------------------------------------

10.22 ------------------------------------------------------------

10.24 ------------------------------------------------------------

10.29 --------------------------------------------------GTCGGATGTA

10.1 ------------------------------------------------------------

10.21 ------------------------------------------------------------

10.5 ---------------------------------------------------TCGGATGTA

10.11 ---------------------------------------------------TCGGATGTA

APHIS-S CGCCTGCGCCGCTTTTAACGTCATTGGGCTCTCTTCTGTCGATGAACTGGGTCGGATGTA

10.19 ------------------------------------------------------------

10.22 ------------------------------------------------------------

10.24 ------------------------------------------------------------

10.29 CTTGTGCATCATAGTGTATGGCGCCGCCAGTCTACCGATAGGCTACGTGTTCTCCTATTT

10.1 ------------------------------------------------------------

10.21 ------------------------------------------------------------

10.5 CTTGTGCATCATAGTGTATGGCGCCGCCAGTCTACCGATAGGCTACGTGTTCTCCTATTT

10.11 CTTGTGCATCATAGTGTATGGCGCCGCCAGTCTACCGATAGGCTACGTGTTCTCCTATTT

APHIS-S CTTGTGCATCATAGTGTATGGCGCCGCCAGTCTACCGATAGGCTACGTGTTCTCCTATTT

10.19 ------------------------------------------------------------

10.22 ------------------------------------------------------------

10.24 ------------------------------------------------------------

10.29 CTTCAAAGGCCCTGCCGTCGGTTTTGTCACCATGTTCTTTATCAACATTCTCTTTGGTAT

10.1 ------------------------------------------------------------

10.21 ------------------------------------------------------------

10.5 CTTCAAAGGCCCTGCCGTCGGTTTTGTCACCATGTTCTTTATCAACATTCTCTTTGGTAT

10.11 CTTCAAAGGCCCTGCCGTCGGTTTTGTCACCATGTTCTTTATCAACATTCTCTTTGGTAT

APHIS-S CTTCAAAGGCCCTGCCGTCGGTTTTGTCACCATGTTCTTTATCAACATTCTCTTTGGTAT

10.19 ------------------------------------------------------------

10.22 ------------------------------------------------------------

10.24 ------------------------------------------------------------

10.29 GATGGGGGCGCAGATTGTGGAGGCCTTGTTGTCACCGCAGCTTGATACTGAAAATGTCGC

10.1 ------------------------------------------------------------

10.21 ------------------------------------------------------------

10.5 GATGGGGGCGCAGATTGTGGAGGCCTTGTTGTCACCGCAGCTTGATACTGAAAATGTCGC

10.11 GATGGGGGCGCAGATTGTGGAGGCCTTGTTGTCACCGCAGCTTGATACTGAAAATGTCGC

APHIS-S GATGGGGGCGCAGATTGTGGAGGCCTTGTTGTCACCGCAGCTTGATACTGAAAATGTCGC

10.19 ------------------------------------------------------------

10.22 ------------------------------------------------------------

10.24 ------------------------------------------------------------

10.29 TAATATACTTGACTCCATCTTGCAATTCTTCCCACTCTATAGTCTTGTCACATCTGCCAG

10.1 ------------------------------------------------------------

10.21 ------------------------------------------------------------

10.5 TAATATACTTGACTCCATCTTGCAATTCTTCCCACTCTATAGTCTTGTCACATCTGCCAG

10.11 TAATATACTTGACTCCATCTTGCAATTCTTCCCACTCTATAGTCTTGTCACATCTGCCAG

APHIS-S TAATATACTTGACTCCATCTTGCAATTCTTCCCACTCTATAGTCTTGTCACATCTGCCAG

10.19 ------------------------------------------------------------

10.22 ------------------------------------------------------------

10.24 ------------------------------------------------------------

10.29 ACTGTTGAATCAGGTGGGACTGCTGGAGTGGTCGTGCCTGCAGAACTGCGAGTACCTGTC

10.1 ------------------------------------------------------------

10.21 ------------------------------------------------------------

10.5 ACTGTTGAATCAGGTGGGACTGCTGGAGTGGTCGTGCCTGCAGAACTGCGAGTACCTGTC

10.11 ACTGTTGAATCAGGTGGGACTGCTGGAGTGGTCGTGCCTGCAGAACTGCGAGTACCTGTC

APHIS-S ACTGTTGAATCAGGTGGGACTGCTGGAGTGGTCGTGCCTGCAGAACTGCGAGTACCTGTC

10.19 ------------------------------------------------------------

10.22 ------------------------------------------------------------

10.24 ------------------------------------------------------------

10.29 CGCAGTGATGCCCAACTTGACCGAATGCTCCATGGACGTTATGTGCCAGACGTTCTCACA

10.1 ------------------------------------------------------------

10.21 ------------------------------------------------------------

10.5 CGCAGTGATGCCCAACTTGACCGAATGCTCCATGGACGTTATGTGCCAGACGTTCTCACA

10.11 CGCAGTGATGCCCAACTTGACCGAATGCTCCATGGACGTTATGTGCCAGACGTTCTCACA

APHIS-S CGCAGTGATGCCCAACTTGACCGAATGCTCCATGGACGTTATGTGCCAGACGTTCTCACA

10.19 ------------------------------------------------------------

10.22 ------------------------------------------------------------

10.24 ------------------------------------------------------------

10.29 ATGTTGCATCCCAGACGATCCTTGGTTCATGTGGGATCACCCTGGAGTACTCCGCTACAT

10.1 ------------------------------------------------------------

10.21 ------------------------------------------------------------

10.5 ATGTTGCATCCCAGACGATCCTTGGTTCATGTGGGATCACCCTGGAGTACTCCGCTACAT

10.11 ATGTTGCATCCCAGACGATCCTTGGTTCATGTGGGATCACCCTGGAGTACTCCGCTACAT

APHIS-S ATGTTGCATCCCAGACGATCCTTGGTTCATGTGGGATCACCCTGGAGTACTCCGCTACAT

10.19 ------------------------------------------------------------

10.22 ------------------------------------------------------------

10.24 ------------------------------------------------------------

10.29 AGTATGCATGATCGTCAGTGGAGTTGTCATGTGGTTCGTACTCTTGATCGCCGAGTATCG

10.1 ------------------------------------------------------------

10.21 ------------------------------------------------------------

10.5 AGTATGCATGATCGTCAGTGGAGTTGTCATGTGGTTCGTACTCTTGATCGCCGAGTATCG

10.11 AGTATGCATGATCGTCAGTGGAGTTGTCATGTGGTTCGTACTCTTGATCGCCGAGTATCG

APHIS-S AGTATGCATGATCGTCAGTGGAGTTGTCATGTGGTTCGTACTCTTGATCGCCGAGTATCG

10.19 ------------------------------------------------------------

10.22 ------------------------------------------------------------

10.24 ------------------------------------------------------------

10.29 ATTGTTCCAGAAGGTGATCTACCGGGAAAAGAAAGCTCCTCCAGTTGATGAGAGCGCACT

10.1 ------------------------------------------------------------

10.21 ------------------------------------------------------------

10.5 ATTGTTCCAGAAGGTGATCTACCGGGAAAAGAAAGCTCCTCCAGTTGATGAGAGCGCACT

10.11 ATTGTTCCAGAAGGTGATCTACCGGGAAAAGAAAGCTCCTCCAGTTGATGAGAGCGCACT

APHIS-S ATTGTTCCAGAAGGTGATCTACCGGGAAAAGAAAGCTCCTCCAGTTGATGAGAGCGCACT

10.19 ------------------------------------------------------------

10.22 ------------------------------------------------------------

10.24 ------------------------------------------------------------

10.29 GGACAATGACGTGGCGGACGAGGCCAGACACGTGGCGCGAGTTGGAGCAGGAGCAATCCT

10.1 ------------------------------------------------------------

10.21 ------------------------------------------------------------

10.5 GGACAATGACGTGGCGGACGAGGCCAGACACGTGGCGCGAGTTGGAGCAGGAGCAATCCT

10.11 GGACAATGACGTGGCGGACGAGGCCAGACACGTGGCGCGAGTTGGAGCAGGAGCAATCCT

APHIS-S GGACAATGACGTGGCGGACGAGGCCAGACACGTGGCGCGAGTTGGAGCAGGAGCAATCCT

10.19 ------------------------------------------------------------

10.22 ------------------------------------------------------------

10.24 ------------------------------------------------------------

10.29 CGGGCAGCACAGCCTAGTAGCAAATGGCCTCACCAAGTATTATGGGAAACACCTTGCAGT

10.1 ------------------------------------------------------------

10.21 ------------------------------------------------------------

10.5 CGGGCAGCACAGCCTAGTAGCAAATGGCCTCACCAAGTATTATGGGAAACACCTTGCAGT

10.11 CGGGCAGCACAGCCTAGTAGCAAATGGCCTCACCAAGTATTATGGGAAACACCTTGCAGT

APHIS-S CGGGCAGCACAGCCTAGTAGCAAATGGCCTCACCAAGTATTATGGGAAACACCTTGCAGT

10.19 ------------------------------------------------------------

10.22 ------------------------------------------------------------

10.24 ------------------------------------------------------------

10.29 CAATCAAGTGTCATTCACCGTGGGCGACACGGAATGCTTTGGTCTTCTGGGTGTGAACGG

10.1 ------------------------------------------------------------

10.21 ------------------------------------------------------------

10.5 CAATCAAGTGTCATTCACCGTGGGCGACACGGAATGCTTTGGTCTTCTGGGTGTGAACGG

10.11 CAATCAAGTGTCATTCACCGTGGGCGACACGGAATGCTTTGGTCTTCTGGGTGTGAACGG

APHIS-S CAATCAAGTGTCATTCACCGTGGGCGACACGGAATGCTTTGGTCTTCTGGGTGTGAACGG

10.19 ------------------------------------------------------------

10.22 ------------------------------------------------------------

10.24 ------------------------------------------------------------

10.29 CGCCGGTAAGACGACCACCTTCAAGATGTTGATGGGAGATGAGACCGTCTCCAGCGGAGA

10.1 ------------------------------------------------------------

10.21 ------------------------------------------------------------

10.5 CGCCGGTAAGACGACCACCTTCAAGATGTTGATGGGAGATGAGACCGTCTCCAGCGGAGA

10.11 CGCCGGTAAGACGACCACCTTCAAGATGTTGATGGGAGATGAGACCGTCTCCAGCGGAGA

APHIS-S CGCCGGTAAGACGACCACCTTCAAGATGTTGATGGGAGATGAGACCGTCTCCAGCGGAGA

10.19 ------------------------------------------------------------

10.22 ------------------------------------------------------------

10.24 ------------------------------------------------------------

10.29 TGCCTTCGTGAGTGGCCATTCTGTCAAGACTAATATCACTCAAGTTTACAAAAATATTGG

10.1 ------------------------------------------------------------

10.21 ------------------------------------------------------------

10.5 TGCCTTCGTGAGTGGCCATTCTGTCAAGACTAATATCACTCAAGTTTACAAAAATATTGG

10.11 TGCCTTCGTGAGTGGCCATTCTGTCAAGACTAATATCACTCAAGTTTACAAAAATATTGG

APHIS-S TGCCTTCGTGAGTGGCCATTCTGTCAAGACTAATATCACTCAAGTTTACAAAAATATTGG

10.19 ------------------------------------------------------------

10.22 ------------------------------------------------------------

10.24 ------------------------------------------------------------

10.29 TTACTGTCCGCAATTCGAAGCGACATTCGGCGAGCTGACGGGACGCGAGACACTACGGCT

10.1 ------------------------------------------------------------

10.21 ------------------------------------------------------------

10.5 TTACTGTCCGCAATTCGAAGCGACATTCGGCGAGCTGACGGGACGCGAGACACTACGGCT

10.11 TTACTGTCCGCAATTCGAAGCGACATTCGGCGAGCTGACGGGACGCGAGACACTACGGCT

APHIS-S TTACTGTCCGCAATTCGAAGCGACATTCGGCGAGCTGACGGGACGCGAGACACTACGGCT

10.19 ------------------------------------------------------------

10.22 ------------------------------------------------------------

10.24 ------------------------------------------------------------

10.29 GTTCTCGGCGCTGCGAGGGTTGCCAGTGCGAGGCGCCACGCTCCACGCGGAGGCCTTAGC

10.1 ------------------------------------------------------------

10.21 ------------------------------------------------------------

10.5 GTTCTCGGCGCTGCGAGGGTTGCCAGTGCGAGGCGCCACGCTCCACGCGGAGGCCTTAGC

10.11 GTTCTCGGCGCTGCGAGGGTTGCCAGTGCGAGGCGCCACGCTCCACGCGGAGGCCTTAGC

APHIS-S GTTCTCGGCGCTGCGAGGGTTGCCAGTGCGAGGCGCCACGCTCCACGCGGAGGCCTTAGC

10.19 ------------------------------------------------------------

10.22 ------------------------------------------------------------

10.24 ------------------------------------------------------------

10.29 ACATGCTCTTGGTTTCTATAAGCATCTTGATAAAAGGGTGGACCACTACTCTGGTGGCAA

10.1 ------------------------------------------------------------

10.21 ------------------------------------------------------------

10.5 ACATGCTCTTGGTTTCTATAAGCATCTTGATAAAAGGGTGGACCACTACTCTGGTGGCAA

10.11 ACATGCTCTTGGTTTCTATAAGCATCTTGATAAAAGGGTGGACCACTACTCTGGTGGCAA

APHIS-S ACATGCTCTTGGTTTCTATAAGCATCTTGATAAAAGGGTGGACCACTACTCTGGTGGCAA

10.19 ------------------------------------------------------------

10.22 -------------------------------------------------ATATTCGTCGA

10.24 ------------------------------------------------------------

10.29 CAAGCGCAAGTTGAGCACGGCTGTGGCGTTGCTGGGGCGCACGCGGCTTATATTCGTCGA

10.1 -------------------------------------------------ATATTCGTCGA

10.21 -------------------------------------------------ATATTCGTCGA

10.5 CAAGCGCAAGTTGAGCACGGCTGTGGCGTTGCTGGGGCGCACGCGGCTTATATTCGTCGA

10.11 CAAGCGCAAGTTGAGCACGGCTGTGGCGTTGCTGGGGCGCACGCGGCTTATATTCGTCGA

APHIS-S CAAGCGCAAGTTGAGCACGGCTGTGGCGTTGCTGGGGCGCACGCGGCTTATATTCGTCGA

10.19 ------------------------------------------------------------

10.22 CGAACCCACTACTGGAGTCGATCCTGCTGCTAAGAGACAGATGTGGAACGCGGTTCGAGA

10.24 ------------------------------------------------------------

10.29 CGAACCCACTACTGGAGTCGATCCTGCTGCTAAGAGACAGATGTGGAACGCGGTTCGAGA

10.1 CGAACCCACTACTGGAGTCGATCCTGCTGCTAAGAGACAGATGTGGAACGCGGTTCGAGA

10.21 CGAACCCACTACTGGAGTCGATCCTGCTGCTAAGAGACAGATGTGGAACGCGGTTCGAGA

10.5 CGAACCCACTACTGGAGTCGATCCTGCTGCTAAGAGACAGATGTGGAACGCGGTTCGAGA

10.11 CGAACCCACTACTGGAGTCGATCCTGCTGCTAAGAGACAGATGTGGAACGCGGTTCGAGA

APHIS-S CGAACCCACTACTGGAGTCGATCCTGCTGCTAAGAGACAGATGTGGAACGCGGTTCGAGA

10.19 ------------------------------------------------------------

10.22 AGCTCGCCGGTCGGGTCGTGGTGTGGTGCTGACATCACACAGCATGGAGGAGTGTGAGGC

10.24 ------------------------------------------------------------

10.29 AGCTCGCCGGTCGGGTCGTGGTGTGGTGCTGACATCACACAGCATGGAGGAGTGTGAGGC

10.1 AGCTCGCCGGTCGGGTCGTGGTGTGGTGCTGACATCACACAGCATGGAGGAGTGTGAGGC

10.21 AGCTCGCCGGTCGGGTCGTGGTGTGGTGCTGACATCACACAGCATGGAGGAGTGTGAGGC

10.5 AGCTCGCCGGTCGGGTCGTGGTGTGGTGCTGACATCACACAGCATGGAGGAGTGTGAGGC

10.11 AGCTCGCCGGTCGGGTCGTGGTGTGGTGCTGACATCACACAGCATGGAGGAGTGTGAGGC

APHIS-S AGCTCGCCGGTCGGGTCGTGGTGTGGTGCTGACATCACACAGCATGGAGGAGTGTGAGGC

10.19 ------------------------------------------------------------

10.22 TCTGTGCTCGCGGCTCACAATCATGGTCAACGGACAGTTCCAGTGCCTCGGCACGCCGCA

10.24 ------------------------------------------------------------

10.29 TCTGTGCTCGCGGCTCACAATCATGGTCAACGGACAGTTCCAGTGCCTCGGCACGCCGCA

10.1 TCTGTGCTCGCGGCTCACAATCATGGTCAACGGACAGTTCCAGTGCCTCGGCACGCCGCA

10.21 TCTGTGCTCGCGGCTCACAATCATGGTCAACGGACAGTTCCAGTGCCTCGGCACGCCGCA

10.5 TCTGTGCTCGCGGCTCACAATCATGGTCAACGGACAGTTCCAGTGCCTCGGCACGCCGCA

10.11 TCTGTGCTCGCGGCTCACAATCATGGTCAACGGACAGTTCCAGTGCCTCGGCACGCCGCA

APHIS-S TCTGTGCTCGCGGCTCACAATCATGGTCAACGGACAGTTCCAGTGCCTCGGCACGCCGCA

10.19 ----------------------------------------------------------CA

10.22 ACATTTAAAGAATAAGTTCTCTGAAGGTTTCACATTGACAATTAAAATTAAAGTGGACGA

10.24 -------------------------GGTTTCACATTGACAATTAAAATTAAAGTGGACGA

10.29 ACATTTAAAGAATAAGTTCTCTGAAGGTTTCACATTGACAATTAAAATTAAAGTGGACGA

10.1 ACATTTAAAGAATAAGTTCTCTGAAGGTTTCACATTGACAATTAAAATTAAAGTGGACGA

10.21 ACATTTAAAGAATAAGTTCTCTGAAGGTTTCACATTGACAATTAAAATTAAAGTGGACGA

10.5 ACATTTAAAGAATAAGTTCTCTGAAGGTTTCACATTGACAATTAAAATTAAAGTGGACGA

10.11 ACATTTAAAGAATAAGTTCTCTGAAGGTTTCACATTGACAATTAAAATTAAAGTGGACGA

APHIS-S ACATTTAAAGAATAAGTTCTCTGAAGGTTTCACATTGACAATTAAAATTAAAGTGGACGA

10.19 ACATTTAAAGAATAAGTTCTCTGAAGGTTTCACATTGACAATTAAAATTAAAGTGGACGA

***********************************

10.22 CGAGACGAAGACTGTACGGCCTGAAGTCTGCGATGCTGTGAAGCATTACGTCAGTACCAA

10.24 CGAGACGAAGACTGTACGGCCTGAAGTCTGCGATGCTGTGAAGCATTACGTCAGTACCAA

10.29 CGAGACGAAGACTGTACGGCCTGAAGTCTGCGATGCTGTGAAGCATTACGTCAGTACCAA

10.1 CGAGACGAAGACTGTACGGCCTGAAGTCTGCGATGCTGTGAAGCATTACGTCAGTACCAA

10.21 CGAGACGAAGACTGTACGGCCTGAAGTCTGCGATGCTGTGAAGCATTACGTCAGTACCAA

10.5 CGAGACGAAGACTGTACGGCCTGAAGTCTGCGATGCTGTGAAGCATTACGTCAGTACCAA

10.11 CGAGACGAAGACTGTACGGCCTGAAGTCTGCGATGCTGTGAAGCATTACGTCAGTACCAA

APHIS-S CGAGACGAAGACTGTACGGCCTGAAGTCTGCGATGCTGTGAAGCATTACGTCAGTACCAA

10.19 CGAGACGAAGACTGTACGGCCTGAAGTCTGCGATGCTGTGAAGCATTACGTCAGTACCAA

************************************************************

10.22 CTTCAGAGAGCCGAAGATTATGGAGGAGTACCAGGGTCTGTTAACATACTATTTGCCAGA

10.24 CTTCAGAGAGCCGAAGATTATGGAGGAGTACCAGGGTCTGTTAACATACTATTTGCCAGA

10.29 CTTCAGAGAGCCGAAGATTATGGAGGAGTACCAGGGTCTGTTAACATACTATTTGCCAGA

10.1 CTTCAGAGAGCCGAAGATTATGGAGGAGTACCAGGGTCTGTTAACATACTATTTGCCAGA

10.21 CTTCAGAGAGCCGAAGATTATGGAGGAGTACCAGGGTCTGTTAACATACTATTTGCCAGA

10.5 CTTCAGAGAGCCGAAGATTATGGAGGAGTACCAGGGTCTGTTAACATACTATTTGCCAGA

10.11 CTTCAGAGAGCCGAAGATTATGGAGGAGTACCAGGGTCTGTTAACATACTATTTGCCAGA

APHIS-S CTTCAGAGAGCCGAAGATTATGGAGGAGTACCAGGGTCTGTTAACATACTATTTGCCAGA

10.19 CTTCAGAGAGCCGAAGATTATGGAGGAGTACCAGGGTCTGTTAACATACTATTTGCCAGA

************************************************************

10.22 CAAGTCGGTGGCGTGGTCCAGAATGTTCGGCATAATGGAGGCGGCCAAACGCGACCTCCC

10.24 CAAGTCGGTGGCGTGGTCCAGAATGTTCGGCATAATGGAGGCGGCCAAACGCGACCTCCC

10.29 CAAGTCGGTGGCGTGGTCCAGAATGTTCGGCATAATGGAGGCGGCCAAACGCGACCTCCC

10.1 CAAGTCGGTGGCGTGGTCCAGAATGTTCGGCATAATGGAGGCGGCCAAACGCGACCTCCC

10.21 CAAGTCGGTGGCGTGGTCCAGAATGTTCGGCATAATGGAGGCGGCCAAACGCGACCTCCC

10.5 CAAGTCGGTGGCGTGGTCCAGAATGTTCGGCATAATGGAGGCGGCCAAACGCGACCTCCC

10.11 CAAGTCGGTGGCGTGGTCCAGAATGTTCGGCATAATGGAGGCGGCCAAACGCGACCTCCC

APHIS-S CAAGTCGGTGGCGTGGTCCAGAATGTTCGGCATAATGGAGGCGGCCAAACGCGACCTCCC

10.19 CAAGTCGGTGGCGTGGTCCAGAATGTTCGGCATAATGGAGGCGGCCAAACGCGACCTCCC

************************************************************

10.22 CGTCGAAGACTACAGCATATCACAAACTACCCTCGAG-----------------------

10.24 CGTCGAAGACTACAGCATATCACAAACTACCCTCGAG-----------------------

10.29 CGTCGAAGACTACAGCATATCACAAACTACCCTCGAG-----------------------

10.1 CGTCGAAGACTACAGCATATCACAAACTACCCTCGAG-----------------------

10.21 CGTCGAAGACTACAGCATATCACAAACTACCCTCGAG-----------------------

10.5 CGTCGAAGACTACAGCATATCACAAACTACCCTCGAG-----------------------

10.11 CGTCGAAGACTACAGCATATCACAAACTACCCTCGAG-----------------------

APHIS-S CGTCGAAGACTACAGCATATCACAAACTACCCTCGAGCAGATATTCCTACAGTTCACAAA

10.19 CGTCGAAGACTACAGCATATCACAAACTACCCTCGAG-----------------------

*************************************

10.22 ----------------------------

10.24 ----------------------------

10.29 ----------------------------

10.1 ----------------------------

10.21 ----------------------------

10.5 ----------------------------

10.11 ----------------------------

APHIS-S GTATCAACATGAAGCACAACAGACATAA

10.19 ----------------------------

**E**

APHIS-S ATGCGGGCGCGTGGAGAGCGGAAGGAGGCGGGCTCATGGGTGAAGTTTAGGCTGTTGATG

18.20 ATGCGGGCGCGTGGAGAGCGGAAGGAGGCGGGCTCATGGGTGAAGTTTAGGCTGTTGATG

18.6 ATGCGGGCGCGTGGAGAGCGGAAGGAGGCGGGCTCATGGGTGAAGTTTAGGCTGTTGATG

18.7 ATGCGGGCGCGTGGAGAGCGGAAGGAGGCGGGCTCATGGGTGAAGTTTAGGCTGTTGATG

************************************************************

APHIS-S TGGAAGAACTTCGTGCAGCAGTTGAGGCACCCAGTGCAGACGGCGGCTGAGCTGCTGCTA

18.20 TGGAAGAACTTCGTGCAGCAGTTGAGGCACCCAGTGCAGACGGCGGCTGAGCTGCTGCTA

18.6 TGGAAGAACTTCGTGCAGCAGTTGAGGCACCCAGTGCAGACGGCGGCTGAGCTGCTGCTA

18.7 TGGAAGAACTTCGTGCAGCAGTTGAGGCACCCAGTGCAGACGGCGGCTGAGCTGCTGCTA

************************************************************

APHIS-S CCAGTCCTAACCATGAGCCTGGTCCTGGTGCTACGGTCACAGATCGACCCCGAAGTCTTG

18.20 CCAGTCCTAACCATGAGCCTGGTCCTGGTGCTACGGTCACAGATCGACCCCGAAGTCTTG

18.6 CCAGTCCTAACCATGAGCCTGGTCCTGGTGCTACGGTCACAGATCGACCCCGAAGTCTTG

18.7 CCAGTCCTAACCATGAGCCTGGTCCTGGTGCTACGGTCACAGATCGACCCCGAAGTCTTG

************************************************************

APHIS-S GAAACCAGAACCTACCCGCCAATACCAGCCCACACTTTAAACTATTCCGTGACTGTTTTG

18.20 GAAACCAGAACCTACCCGCCAATACCAGCCCACACTTTAAACTATTCCGTGACTGTTTTG

18.6 GAAACCAGAACCTACCCGCCAATACCAGCCCACACTTTAAACTATTCCGTGACTGTTTTG

18.7 GAAACCAGAACCTACCCGCCAATACCAGCCCACACTTTAAACTATTCCGTGACTGTTTTG

************************************************************

APHIS-S GGCGGAATGAATTTAACAAGAATGTCCATGGCATTCTCACCCGAGAATGCCGTATTGAGG

18.20 GGCGGAATGAATTTAACAAGAATGTCCATGGCATTCTCACCCGAGAATGCCGTATTGAGG

18.6 GGCGGAATGAATTTAACAAGAATGTCCATGGCATTCTCACCCGAGAATGCCGTATTGAGG

18.7 GGCGGAATGAATTTAACAAGAATGTCCATGGCATTCTCACCCGAGAATGCCGTATTGAGG

************************************************************

APHIS-S GACGTCGTATCCAGTGCTACAACAAAGTTACTGCTTAAAAACATGAGAGACCAAGTACTG

18.20 GACGTCGTATCCAGTGCTACAACAAAGTTACTGCTTAAAAACATGAGAGACCAAGTACTG

18.6 GACGTCGTATCCAGTGCTACAACAAAGTTACTGCTTAAAAACATGAGAGACCAAGTACTG

18.7 GACGTCGTATCCAGTGCTACAACAAAGTTACTGCTTAAAAACATGAGAGACCAAGTACTG

************************************************************

APHIS-S CCCATCATTGAGGCATTGCCAATAGAAATACCGCCGGGACTGGTAAACTCGTCACAGGTG

18.20 CCCATCATTGAGGCATTGCCAATAGAAATACCGCCGGGACTGGTAAACTCGTCACAGGTG

18.6 CCCATCATTGAGGCATTGCCAATAGAAATACCGCCGGGACTGGTAAACTCGTCACAGGTG

18.7 CCCATCATTGAGGCATTGCCAATAGAAATACCGCCGGGACTGGTAAACTCGTCACAGGTG

************************************************************

APHIS-S TACGAAATAGTTAAATTATTTGTCGACGAGAACGTTGTTACCGGATACAATAGCAGTGCG

18.20 TACGAAATAGTTAAATTATTTGTCGACGAGAACGTTGTTACCGGATACAATAGCAGTGCG

18.6 TACGAAATAGTTAAATTATTTGTCGACGAGAACGTTGTTACCGGATACAATAGCAGTGCG

18.7 TACGAAATAGTTAAATTATTTGTCGACGAGAACGTTGTTACCGGATACAATAGCAGTGCG

************************************************************

APHIS-S GCAATGAGAGGAATATACGCAGAGGAAGAAGCCACGAGAAGGGTGATAGCTGGCATAGAA

18.20 GCAATGAGAGGAATATACGCAGAGGAAGAAGCCACGAGAAGGGTGATAGCTGGCATAGAA

18.6 GCAATGAGAGGAATATACGCAGAGGAAGAAGCCACGAGAAGGGTGATAGCTGGCATAGAA

18.7 GCAATGAGAGGAATATACGCAGAGGAAGAAGCCACGAGAAGGGTGATAGCTGGCATAGAA

************************************************************

APHIS-S TTCGATGACTCATTGCGTGAAATAACGGAGCTACCACTAGACTTGTCGTATGCGCTTCGT

18.20 TTCGATGACTCATTGCGTGAAATAACGGAGCTACCACTAGACTTGTCGTATGCGCTTCGT

18.6 TTCGATGACTCATTGCGTGAAATAACGGAGCTACCACTAGACTTGTCGTATGCGCTTCGT

18.7 TTCGATGACTCATTGCGTGAAATAACGGAGCTACCACTAGACTTGTCGTATGCGCTTCGT

************************************************************

APHIS-S TTTCCGGAGAGACCTCGCTTGAATTCCTTCTTCATGACAGGCGGTCGGACTTGGCGCACA

18.20 TTTCCGGAGAGACCTCGCTTGAATTCCTTCTTCATGACAGGCGGTCGGACTTGGCGCACA

18.6 TTTCCGGAGAGACCTCGCTTGAATTCCTTCTTCATGACAGGCGGTCGGACTTGGCGCACA

18.7 TTTCCGGAGAGACCTCGCTTGAATTCCTTCTTCATGACAGGCGGTCGGACTTGGCGCACA

************************************************************

APHIS-S GATAACGTGTTTCCTATGTTCGAAGTTCCCGGACCTCGCTTTCCGTATTCATGGGAAGGT

18.20 GATAACGTGTTTCCTATGTTCGAAGTTCCCGGACCTCGCTTTCCGTATTCATGGGAAGGT

18.6 GATAACGTGTTTCCTATGTTCGAAGTTCCCGGACCTCGCTTTCCGTATTCATGGGAAGGT

18.7 GATAACGTGTTTCCTATGTTCGAAGTTCCCGGACCTCGCTTTCCGTATTCATGGGAAGGT

************************************************************

APHIS-S GGAAATGATCCAGGATACGTAAACGAGATGTTCATAGCCTTGCAGCACATGATATCTTCA

18.20 GGAAATGATCCAGGATACGTAAACGAGATGTTCATAGCCTTGCAGCACATGATATCTTCA

18.6 GGAAATGATCCAGGATACGTAAACGAGATGTTCATAGCCTTGCAGCACATGATATCTTCA

18.7 GGAAATGATCCAGGATACGTAAACGAGATGTTCATAGCCTTGCAGCACATGATATCTTCA

************************************************************

APHIS-S GAACTGGTATCTAAAGTGGCGGGAGTGAACCTAGACTTCGATGTGCACATACAGAGGTAC

18.20 GAACTGGTATCTAAAGTGGCGGGAGTGAACCTAGACTTCGATGTGCACATACAGAGGTAC

18.6 GAACTGGTATCTAAAGTGGCGGGAGTGAACCTAGACTTCGATGTGCACATACAGAGGTAC

18.7 GAACTGGTATCTAAAGTGGCGGGAGTGAACCTAGACTTCGATGTGCACATACAGAGGTAC

************************************************************

APHIS-S CCACATCCAGCATACATCATGGACTTGGCGAAGGAAGCCCTGCAGTTCCTCTTCCCATCA

18.20 CCACATCCAGCATACATCATGGACTTGGCGAAGGAAGCCCTGCAGTTCCTCTTCCCATCA

18.6 CCACATCCAGCATACATCATGGACTTGGCGAAGGAAGCCCTGCAGTTCCTCTTCCCATCA

18.7 CCACATCCAGCATACATCATGGACTTGGCGAAGGAAGCCCTGCAGTTCCTCTTCCCATCA

************************************************************

APHIS-S TTCATCATGATCAGCTTCAGTTACACCGCTATCAATATTATACGATCCGTGACCGTGGAA

18.20 TTCATCATGATCAGCTTCAGTTACACCGCTATCAATATTATACGATCCGTGACCGTGGAA

18.6 TTCATCATGATCAGCTTCAGTTACACCGCTATCAATATTATACGATCCGTGACCGTGGAA

18.7 TTCATCATGATCAGCTTCAGTTACACCGCTATCAATATTATACGATCCGTGACCGTGGAA

************************************************************

APHIS-S AAAGAAATGCAATTGAAGGAAACGATGAAGATCATGGGACTCCCAACGTGGCTGCATTGG

18.20 AAAGAAATGCAATTGAAGGAAACGATGAAGATCATGGGACTCCCAACGTGGCTGCATTGG

18.6 AAAGAAATGCAATTGAAGGAAACGATGAAGATCATGGGACTCCCAACGTGGCTGCATTGG

18.7 AAAGAAATGCAATTGAAGGAAACGATGAAGATCATGGGACTCCCAACGTGGCTGCATTGG

************************************************************

APHIS-S ATGGCATGGTTTTTTAAACAATTTATTTATTTGCTGATTGCTTCGGTTTTGATACTTGTT

18.20 ATGGCATGGTTTTTTAAACAATTTATTTATTTGCTGATTGCTTCGGTTTTGATACTTGTT

18.6 ATGGCATGGTTTTTTAAACAATTTATTTATTTGCTGATTGCTTCGGTTTTGATACTTGTT

18.7 ATGGCATGGTTTTTTAAACAATTTATTTATTTGCTGATTGCTTCGGTTTTGATACTTGTT

************************************************************

APHIS-S ATATTAAAGGTAAATTGGTTTACTACAGAAGAAGGCTTTAGCGACTATGCCGTATTCACT

18.20 ATATTAAAGGTAAATTGGTTTACTACAGAAGAAGGCTTTAGCGACTATGCCGTATTCACT

18.6 ATATTAAAGGTAAATTGGTTTACTACAGAAGAAGGCTTTAGCGACTATGCCGTATTCACT

18.7 ATATTAAAGGTAAATTGGTTTACTACAGAAGAAGGCTTTAGCGACTATGCCGTATTCACT

************************************************************

APHIS-S AATACACCTTGGACCGTCCTCTTCTTCTTCCTAACACTGTATCTTACGTGTACCATATTT

18.20 AATACACCTTGGACCGTCCTCTTCTTCTTCCTAACACTGTATCTTACGTGTACCATATTT

18.6 AATACACCTTGGACCGTCCTCTTCTTCTTCCTAACACTGTATCTTACGTGTACCATATTT

18.7 AATACACCTTGGACCGTCCTCTTCTTCTTCCTAACACTGTATCTTACGTGTACCATATTT

************************************************************

APHIS-S TTCTGTTTCATGATAAGTGGTTTCTTTTCAAAAGCCAGTACAGCGGCGTTGTTTGGTGGG

18.20 TTCTGTTTCATGATAAGTGGTTTCTTTTCAAAAGCCAGTACAGCGGCGTTGTTTGGTGGG

18.6 TTCTGTTTCATGATAAGTGGTTTCTTTTCAAAAGCCAGTACAGCGGCGTTGTTTGGTGGG

18.7 TTCTGTTTCATGATAAGTGGTTTCTTTTCAAAAGCCAGTACAGCGGCGTTGTTTGGTGGG

************************************************************

APHIS-S GTGATCTGGTTTCTGACGTATATCCCCGCATTCCTCCTGGCTATGGACGTGAACATGTCT

18.20 GTGATCTGGTTTCTGACGTATATCCCCGCATTCCTCCTGGCTATGGACGTGAACATGTCT

18.6 GTGATCTGGTTTCTGACGTATATCCCCGCATTCCTCCTGGCTATGGACGTGAACATGTCT

18.7 GTGATCTGGTTTCTGACGTATATCCCCGCATTCCTCCTGGCTATGGACGTGAACATGTCT

************************************************************

APHIS-S ACCTCTCTACAAGCGGTCACCTGCCTAATGCTCAACTCCGCCATGTCTTACGGCTTCCAG

18.20 ACCTCTCTACAAGCGGTCACCTGCCTAATGCTCAACTCCGCCATGTCTTACGGCTTCCAG

18.6 ACCTCTCTACAAGCGGTCACCTGCCTAATGCTCAACTCCGCCATGTCTTACGGCTTCCAG

18.7 ACCTCTCTACAAGCGGTCACCTGCCTAATGCTCAACTCCGCCATGTCTTACGGCTTCCAG

************************************************************

APHIS-S CTGTTACTGGCCCGGGAAAGTACCGGAGGAATGCAGTGGGGTGATTTTATGACGTCACCA

18.20 CTGTTACTGGCCCGGGAAAGTACCGGAGGAATGCAGTGGGGTGATTTTATGACGTCACCA

18.6 CTGTTACTGGCCCGGGAAAGTACCGGAGGAATGCAGTGGGGTGATTTTATGACGTCACCA

18.7 CTGTTACTGGCCCGGGAAAGTACCGGAGGAATGCAGTGGGGTGATTTTATGACGTCACCA

************************************************************

APHIS-S GCAACGGACTCGTCACGATTCGTATTCGGTCACGTCGTTATAATGATGGCTTTGAACTGT

18.20 GCAACGGACTCGTCACGATTCGTATTCGGTCACGTCGTTATAATGATGGCTTTGAACTGT

18.6 GCAACGGACTCGTCACGATTCGTATTCGGTCACGTCGTTATAATGATGGCTTTGAACTGT

18.7 GCAACGGACTCGTCACGATTCGTATTCGGTCACGTCGTTATAATGATGGCTTTGAACTGT

************************************************************

APHIS-S GTGCTCTACATGTTGATTGCCCTATATCTAGAGCAAGTACTACCCGGGCCGTATGGCACA

18.20 GTGCTCTACATGTTGATTGCCCTATATCTAGAGCAAGTACTACCCGGGCCGTATGGCACA

18.6 GTGCTCTACATGTTGATTGCCCTATATCTAGAGCAAGTACTACCCGGGCCGTATGGCACA

18.7 GTGCTCTACATGTTGATTGCCCTATATCTAGAGCAAGTACTACCCGGGCCGTATGGCACA

************************************************************

APHIS-S CCGAAGCCCTGGTATTTCTTCGTCCAAAGACAGTTCTGGTGTAGCAGCAAAACTACTCAT

18.20 CCGAAGCTCTGGTATTTCTTCGTCCAAAGACAGTTCTGGTGTAGCAGCAAAACTACTCAT

18.6 CCGAAGCCCTGGTATTTCTTCGTCCAAAGACAGTTCTGGTGTAGCAGCAAAACTACTCAT

18.7 CCGAAGCCCTGGTATTTCTTCGTCCAAAGACAGTTCTGGTGTAGCAGCAAAACTACTCAT

******* ****************************************************

APHIS-S GATATCGGTACAGACAACAGCGACACATCAAGTTTAACAAAAGAAAGCGACCCTACAGAC

18.20 GATATCGGTACAGACAACAGCGACACATCAAGTTTAACAAAAGAAAGCGACCCTACAGAC

18.6 GATATCGGTACAGACAACAGCGACACATCAAGTTTAACAAAAGAAAGCGACCCTACAGAC

18.7 GATATCGGTACAGACAACAGCGACACATCAAGTTTAACAAAAGAAAGCGACCCTACAGAC

************************************************************

APHIS-S CTTCCGATTGGAGTTAAAATACAAAACCTTAAAAAGGTTTACGGGAGCAACGTTGCGGTA

18.20 CTTCCGATTGGAGTTAAAATACAAAACCTTAAAAAGGTTTACGGGAGCAACGTTGCGGTA

18.6 CTTCCGATTGGAGTTAAAATACAAAACCTTAAAAAGGTTTACGGGAGCAACGTTGCGGTA

18.7 CTTCCGATTGGAGTTAAAATACAAAACCTTAAAAAGGTTTACGGGAGCAACGTTGCGGTA

************************************************************

APHIS-S AACAATTTATCCCTCAACATTTACGACGACCAAATCACGGTTCTACTTGGACACAACGGA

18.20 AACAATTTATCCCTCAACATTTACGACGACCAAATCACGGTTCTACTTGGACACAACGGA

18.6 AACAATTTATCCCTCAACATTTACGACGACCAAATCACGGTTCTACTTGGACACAACGGA

18.7 AACAATTTATCCCTCAACATTTACGACGACCAAATCACGGTTCTACTTGGACACAACGGA

************************************************************

APHIS-S GCGGGAAAATCCACAACCATTTCAATGCTCACAGGTAACGTGGACATAACCAGCGGGTCG

18.20 GCGGGAAAATCCACAACCATTTCAATGCTCACAGGTAACGTGGACATAACCAGCGGGTCG

18.6 GCGGGAAAATCCACAACCATTTCAATGCTCACAGGTAACGTGGACATAACCAGCGGGTCG

18.7 GCGGGAAAATCCACAACCATTTCAATGCTCACAGGTAACGTGGACATAACCAGCGGGTCG

************************************************************

APHIS-S GTGACGGTGGCTGGCTACGACATAGAAAAACAAACAAGTTCAGCACGCTCACACATTGGA

18.20 GTGACGGTGGCTGGCTACGACATAGAAAAACAAACAAGTTCAGCACGCTCACACATTGGA

18.6 GTGACGGTGGCTGGCTACGACATAGAAAAACAAACAAGTTCAGCACGCTCACACATTGGA

18.7 GTGACGGTGGCTGGCTACGACATAGAAAAACAAACAAGTTCAGCACGCTCACACATTGGA

************************************************************

APHIS-S CTCTGCCCTCAACATAACGTACTCTTCAACGAACTCACAGTCAAAGAACATTTACAGTTC

18.20 CTCTGCCCTCAACATAACGTACTCTTCAACGAACTCACAGTCAAAGAACATTTACAGTTC

18.6 CTCTGCCCTCAACATAACGTACTCTTCAACGAACTCACAGTCAAAGAACATTTACAGTTC

18.7 CTCTGCCCTCAACATAACGTACTCTTCAACGAACTCACAGTCAAAGAACATTTACAGTTC

************************************************************

APHIS-S TTCTCTCGTCTGAAAGGCTTCAGCGGTAAAGAGTTGGATGAAGAAATTGAGACGCTTATT

18.20 TTCTCTCGTCTGAAAGGCTTCAGCGGTAAAGAGTTGGATGAAGAAATTGAGACGCTTATT

18.6 TTCTCTCGTCTGAAAGGCTTCAGCGGTAAAGAGTTGGATGAAGAAATTGAGACGCTTATT

18.7 TTCTCTCGTCTGAAAGGCTTCAGCGGTAAAGAGTTGGATGAAGAAATTGAGACGCTTATT

************************************************************

APHIS-S GAAAAATTGGAATTGCAAGAAAAGAGGGATTACCAATCAGCGGGGTTATCAGGGGGACAG

18.20 GAAAAATTGGAATTGCAAGAAAAGAGGGATTACCAATCAGCGGGGTTATCAGGGGGACAG

18.6 GAAAAATTGGAATTGCAAGAAAAGAGGGATTACCAATCAGCGGGGTTATCAGGGGGACAG

18.7 GAAAAATTGGAATTGCAAGAAAAGAGGGATTACCAATCAGCGGGGTTATCAGGGGGACAG

************************************************************

APHIS-S AAGCGACGATTAGGAGTGGGCGTCGCGCTATGCGGGGCGGCTAAAGTGGTTCTACTGGAC

18.20 AAGCGACGATTAGGAGTGGGCGTCGCGCTATGCGGGGCGGCTAAAGTGGTTCTACTGGAC

18.6 AAGCGACGATTAGGAGTGGGCGTCGCGCTATGCGGGGCGGCTAAAGTGGTTCTACTGGAC

18.7 AAGCGACGATTAGGAGTGGGCGTCGCGCTATGCGGGGCGGCTAAAGTGGTTCTACTGGAC

************************************************************

APHIS-S GAGCCCACTTCTGGCATGGACCCGGCCTCACGTCGTGCCCTATGGGACTTGTTGCAGAGA

18.20 GAGCCCACTTCTGGCATGGACCCGGCCTCACGTCGTGCCCTATGGGACTTGTTGCAGAGA

18.6 GAGCCCACTTCTGGCATGGACCCGGCCTCACGTCGTGCCCTATGGGACTTGTTGCAGAGA

18.7 GAGCCCACTTCTGGCATGGACCCGGCCTCACGTCGTGCCCTATGGGACTTGTTGCAGAGA

************************************************************

APHIS-S GAGAAGAAAGGTCGATCGATGATCCTGACGACACACTTCATGGACGAAGCGGACATATTA

18.20 GAGAAGAAAGGTCGATCGATGATCCTGACGACACACTTCATGGACGAAGCGGACATATTA

18.6 GAGAAGAAAGGTCGATCGATGATCCTGACGACACACTTCATGGACGAAGCGGACATATTA

18.7 GAGAAGAAAGGTCGATCGATGATCCTGACGACACACTTCATGGACGAAGCGGACATATTA

************************************************************

APHIS-S GGGGATAGAGTTGCCATTATGGCGGACGGTCGTCTCCAGTGCGTGGGCTCACCTTACTTC

18.20 GGGGATAGAGTTGCCATTATGGCGGACGGTCGTCTCCAGTGCGTGGGCTCACCTTACTTC

18.6 GGGGATAGAGTTGCCATTATGGCGGACGGTCGTCTCCAGTGCGTGGGCTCACCTTACTTC

18.7 GGGGATAGAGTTGCCATTATGGCGGACGGTCGTCTCCAGTGCGTGGGCTCACCTTACTTC

************************************************************

APHIS-S CTCAAGAGACACTATGGAGTCGGCTACACGCTAGTTGTGGTCAAGAAGGAAGATTTCCGA

18.20 CTCAAGAGACACTATGGAGTCGGCTACACGCTAGTTGTGGTCAAGAAGGAAGATTTCCGA

18.6 CTCAAGAGACACTATGGAGTCGGCTACACGCTAGTTGTGGTCAAGAAGGAAGATTTCCGA

18.7 CTCAAGAGACACTATGGAGTCGGCTACACGCTAGTTGTGGTCAAGAAGGAAGATTTCCGA

************************************************************

APHIS-S CTGGACACCTGCACAGAGCTGATCAATAGATACATCCCTGGAACTGTTGTGAAGGAAGAC

18.20 CTGGACACCTGCACAGAGCTGATCAATAGATACATCCCTGGAACTGTTGTGAAGGAAGAC

18.6 CTGGACACCTGCACAGAGCTGATCAATAGATACATCCCTGGAACTGTTGTGAAGGAAGAC

18.7 CTGGACACCTGCACAGAGCTGATCAATAGATACATCCCTGGAACTGTTGTGAAGGAAGAC

************************************************************

APHIS-S CGAGGCACTGAAGTGACATATAGCATGACTAATGAGTATTCGCACGTGTTTGAATCTATG

18.20 CGAGGCACTGAAGTGACATATAGCATGACTAATGAGTATTCGCACGTGTTTGAATCTATG

18.6 CGAGGCACTGAAGTGACATATAGCATGACTAATGAGTATTCGCACGTGTTTGAATCTATG

18.7 CGAGGCACTGAAGTGACATATAGCATGACTAATGAGTATTCGCACGTGTTTGAATCTATG

************************************************************

APHIS-S CTGCGCGATTTGGAGGCAAAGGCCGATGAGATAAACTTTAAAAACTACGGCCTACTGGCT

18.20 CTGCGCGATTTGGAGGCAAAGGCCGATGAGATAAACTTTAAAAACTACGGCCTACTGGCT

18.6 CTGCGCGATTTGGAGGCAAAGGCCGATGAGATAAACTTTAAAAACTACGGCCTACTGGCT

18.7 CTGCGCGATTTGGAGGCAAAGGCCGATGAGATAAACTTTAAAAACTACGGCCTACTGGCT

************************************************************

APHIS-S ACTACATTAGAAGATGTGTTCATGTCCGTGGGCACAGATGTGGTCGCAACTTCAGATGTG

18.20 ACTACATTAGAAGATGTGTTCATGTCCGTGGGCACAGATGTGGTCGCAACTTCAGATGTG

18.6 ACTACATTAGAAGATGTGTTCATGTCCGTGGGCACAGATGTGGTCGCAACTTCAGATGTG

18.7 ACTACATTAGAAGATGTGTTCATGTCCGTGGGCACAGATGTGGTCGCAACTTCAGATGTG

************************************************************

APHIS-S GACGACAATACAACCGTTTCATCTAGTGCTGATACTCTAGCATTTGAATATGATTCTTTA

18.20 GACGACAATACAACCGTTTCATCTAGTGCTGATACTCTAGCATTTGAATATGATTCTTTA

18.6 GACGACAATACAACCGTTTCATCTAGTGCTGATACTCTAGCATTTGAATATGATTCTTTA

18.7 GACGACAATACAACCGTTTCATCTAGTGCTGATACTCTAGCATTTGAATATGATTCTTTA

************************************************************

APHIS-S GAAAAATTGGACGGGACTGGCTATGGGGATGAAAAAGGGATCCGATTAATTTGCCAACAC

18.20 GAAAAATTGGACGGGACTGGCTATGGGGATGAAAAAGGGATCCGATTAATTTGCCAACAC

18.6 GAAAAATTGGACGGGACTGGCTATGGGGATGAAAAAGGGATCCGATTAATTTGCCAACAC

18.7 GAAAAATTGGACGGGACTGGCTATGGGGATGAAAAAGGGATCCGATTAATTTGCCAACAC

************************************************************

APHIS-S GTGGTAGCAATATGGATGAAACTGTTTCTGGTGCTGACAAGGTCTTGGCTTATCCTGTTG

18.20 GTGGTAGCAATATGGATGAAGCTGTTTCTGGTGCTGACAAGGTCTTGGCTTATCCTGTTG

18.6 GTGGTAGCAATATGGATGAAACTGTTTCTGGTGCTGACAAGGTCTTGGCTTATCCTGTTG

18.7 GTGGTAGCAATATGGATGAAACTGTTTCTGGTGCTGACAAGGTCTTGGCTTATCCTGTTG

******************** ***************************************

APHIS-S CTCCAAGTATTGGTGTCCTTGGTACAAATCATTGCCACACTCGGAGTCATGCAGTATGTC

18.20 CTCCAAGTATTGGTGTCCTTGGTACAAATCATTGCCACACTCGGAGTCATGCAGTATGTC

18.6 CTCCAAGTATTGGTGTCCTTGGTACAAATCATTGCCACACTCGGAGTCATGCAGTATGTC

18.7 CTCCAAGTATTGGTGTCCTTGGTACAAATCATTGCCACACTCGGAGTCATGCAGTATGTC

************************************************************

APHIS-S ATCTCTATGACCGAGCATATACAAAGAAGAGAACTTTCATTGGCTGAAGGTTTCGCAGGC

18.20 ATCTCTATGACCGAGCATATACAAAGAAGAGAACTTTCATTGGCTGAAGGTTTCGCAGGC

18.6 ATCTCTATGACCGAGCATATACAAAGAAGAGAACTTTCATTGGCTGAAGGTTTCGCAGGC

18.7 ATCTCTATGACCGAGCATATACAAAGAAGAGAACTTTCATTGGCTGAAGGTTTCGCAGGC

************************************************************

APHIS-S ACAGAAACATTAGTTAGTTTCAAAGGGTTGTCCCCTACATCGACAGGTTCGCTAGCGAAG

18.20 ACAGAAACATTAGTTAGTTTCAAAGGGTTGTCCCCTACATCGACAGGTTCGCTAGCGAAG

18.6 ACAGAAACATTAGTTAGTTTCAAAGGGTTGTCCCCTACATCGACAGGTTCGCTAGCGAAG

18.7 ACAGAAACATTAGTTAGTTTCAAAGGGTTGTCCCCTACATCGACAGGTTCGCTAGCGAAG

************************************************************

APHIS-S GCTGCCTACGAGTCGATATTTGTAACCGCCAATAATCCCACAATGGAAATCACTGTTGTT

18.20 GCTGCCTACGAGTCGATATTTGTAACCGCCAATAATCCCACAATGGAAATCACTGTTGTT

18.6 GCTGCCTACGAGTCGATATTTGTAACCGCCAATAATCCCACAATGGAAATCACTGTTGTT

18.7 GCTGCCTACGAGTCGATATTTGTAACCGCCAATAATCCCACAATGGAAATCACTGTTGTT

************************************************************

APHIS-S GATAATACACCTATAGATGAATATTATTTGGAAAGAACAGATGACGTATCAGCGATGGCG

18.20 GATAATACACCTATAGATGAATATTATTTGGAAAGAACAGATGACGTATCAGCGATGGCG

18.6 GATAATACACCTATAGATGAATATTATTTGGAAAGAACAGATGACGTATCAGCGATGGCG

18.7 GATAATACACCTATAGATGAATATTATTTGGAAAGAACAGATGACGTATCAGCGATGGCG

************************************************************

APHIS-S GTGCTCCGGCACAGTCTGTTGATCGGCGCGACGTTCGACGACCACTCCGCGACCGCGTGG

18.20 GTGCTCCGGCACAGTCTGTTGATCGGCGCGACGTTCGACGACCACTCCGCGACCGCGTGG

18.6 GTGCTCCGGCACAGTCTGTTGATCGGCGCGACGTTCGACGACCACTCCGCGACCGCGTGG

18.7 GTGCTCCGGCACAGTCTGTTGATCGGCGCGACGTTCGACGACCACTCCGCGACCGCGTGG

************************************************************

APHIS-S TTCAGCAACTTCGGTTACCACGACGTGGCCATGTCACTGGCTGCTGTGCACGCCGCCTTG

18.20 TTCAGCAACTTCGGTTACCACGACGTGGCCATGTCACTGGCTGCTGTGCACGCCGCCTTG

18.6 TTCAGCAACTTCGGTTACCACGACGTGGCCATGTCACTGGCTGCTGTGCACGCCGCCTTG

18.7 TTCAGCAACTTCGGTTACCACGACGTGGCCATGTCACTGGCTGCTGTGCACGCCGCCTTG

************************************************************

APHIS-S CTCAGAGCTGTCAATCCTGCAGCCAACTTGACTGTTTACAACCACCCACTTGAGGCCAAT

18.20 CTCAGAGCTGTCAATCCTGCAGCCAACTTGACTGTTTACAACCACCCACTTGAGGCCAAT

18.6 CTCAGAGCTGTCAATCCTGCAGCCAACTTGACTGTTTACAACCACCCACTTGAGGCCAAT

18.7 CTCAGAGCTGTCAATCCTGCAGCCAACTTGACTGTTTACAACCACCCACTTGAGGCCAAT

************************************************************

APHIS-S TATGTCAACCAGAACGACATGCAAACAATGGTAGCGTTCCTCTCGATGCAACTTGCGTCG

18.20 TATGTCAACCAGAACGACATGCAAACAATGGTAGCGTTCCTCTCGATGCAACTTGCGTCG

18.6 TATGTCAACCAGAACGACATGCAAACAATGGTAGCGTTCCTCTCGATGCAACTTGCGTCG

18.7 TATGTCAACCAGAACGACATGCAAACAATGGTAGCGTTCCTCTCGATGCAACTTGCGTCG

************************************************************

APHIS-S GGCATCGGCAGCAGTCTGTCAATTGTCAGTGCTGTGTTCATCATGTTCTATATCAAGGAG

18.20 GGCATCGGCAGCAGTCTGTCAATTGTCAGTGCTGTGTTCATCATGTTCTATATCAAGGAG

18.6 GGCATCGGCAGCAGTCTGTCAATTGTCAGTGCTGTGTTCATCATGTTCTATATCAAGGAG

18.7 GGCATCGGCAGCAGTCTGTCAATTGTCAGTGCTGTGTTCATCATGTTCTATATCAAGGAG

************************************************************

APHIS-S CGAGTATCTCGCGCCAAGCTGCTGCAGAAGGCGGCAGGCATCCAGCCGTTAGTGATGTGG

18.20 CGAGTATCTCGCGCCAAGCTGCTGCAGAAGGCGGCAGGCATCCAGCCGTTAGTGATGTGG

18.6 CGAGTATCTCGCGCCAAGCTGCTGCAGAAGGCGGCAGGCATCCAGCCGTTAGTGATGTGG

18.7 CGAGTATCTCGCGCCAAGCTGCTGCAGAAGGCGGCAGGCATCCAGCCGTTAGTGATGTGG

************************************************************

APHIS-S CTCAGCGCCGCCGTGTTCGACTGGATCTGGTTCTGCGTCATCGCCGTCGGCATCGTTATC

18.20 CTCAGCGCCGCCGTGTTCGACTGGATCTGGTTCTGCGTCATCGCCGTCGGCATCGTTATC

18.6 CTCAGCGCCGCCGTGTTCGACTGGATCTGGTTCTGCGTCATCGCCGTCGGCATCGTTATC

18.7 CTCAGCGCCGCCGTGTTCGACTGGATCTGGTTCTGCGTCATCGCCGTCGGCATCGTTATC

************************************************************

APHIS-S GCCTGCGCCGCTTTTA--------------------------------------------

18.20 GCCTGCGCCGCTTTTGCGCGCCGTGTTTATTACGGTCATGGCGTCAAAAGATATAGAGCT

18.6 GCCTGCGCCGCTTTTGCGCGCCGTGTTTATTACGGTCATGGCGTCAAAAGATATAGAGCT

18.7 GCCTGCGCCGCTTTTGCGCGCCGTGTTTATTACGGTCATGGCGTCAAAAGATATAGAGCT

***************

APHIS-S ------------------------------ACGTCATTGGGCTC----------------

18.20 GAATACCTTTATTGTGTATTTCAGTGTTCCACATCGCTGTACTCGGTGAAGTATGCATAA

18.6 GAATACCTTTATTGTGTATTTCAGTGTTCCACATCGCTGTACTCGGTGAAGTATGCATAA

18.7 GAATACCTTTATTGTGTATTTCAGTGTTCCACATCGCTGTACTCGGTGAAGTATGCATAA

** ** ** ***

APHIS-S ------------------------------------------------------------

18.20 AATATGTCGCCTTTTCGGAACTGGCTTGATGGAAATCAATATTTATGTTATCAGTCAGGA

18.6 AATATGTCGCCTTTTCGGAACTGGCTTGATGGAAATCAATATTTATGTTATCAGTCAGGA

18.7 AATATGTCGCCTTTTCGGAACTGGCTTGATGGAAATCAATATTTATGTTATCAGTCAGGA

APHIS-S ----------------------------------TCTTCTG-------------------

18.20 TTAGTTGGACGACTCATAAAATATCAATCAATATTCTCTTGCAGCCTCATGAGATCGATA

18.6 TTAGTTGGACGACTCATAAAATATCAATCAATATTCTCTTGCAGCCTCATGAGATCGATA

18.7 TTAGTTGGACGACTCATAAAATATCAATCAATATTCTCTTGCAGCCTCATGAGATCGATA

*** **

APHIS-S ----TCGATGAACT----------------------------------------------

18.20 TGACTCAATGTACTTTACTGAAGTGCTTGCACCTACTGGGTAATAGATAAAACCAATTGT

18.6 TGACTCAATGTACTTTACTGAAGTGCTTGCACCTACTGGGTAATAGATAAAACCAATTGT

18.7 TGACTCAATGTACTTTACTGAAGTGCTTGCACCTACTGGGTAATAGATAAAACCAATTGT

** *** ***

APHIS-S --------------------------------------GGGTCGGATGTACTTGTGCATC

18.20 TTTGTTCATATGTTAGAACCATGTTTTTTTTTTCCTTCAGGTCGGATGTACTTGTGCATC

18.6 TTTGTTCATATGTTAGAACCATG-TTTTTTTTTCCTTCAGGTCGGATGTACTTGTGCATC

18.7 TTTGTTCATATGTTAGAACCATGTTTTTTTTTTCCTTCAGGTCGGATGTACTTGTGCATC

*********************

APHIS-S ATAGTGTATGGCGCCGCCAGTCTACCGATAGGCTACGTGTTCTCCTATTTCTTCAAAGGC

18.20 ATAGTGTATGGCGCCGCCAGTCTACCGATAGGCTACGTGTTCTCCTATTTCTTCAAAGGC

18.6 ATAGTGTATGGCGCCGCCAGTCTACCGATAGGCTACGTGTTCTCCTATTTCTTCAAAGGC

18.7 ATAGTGTATGGCGCCGCCAGTCTACCGATAGGCTACGTGTTCTCCTATTTCTTCAAAGGC

************************************************************

APHIS-S CCTGCCGTCGGTTTTGTCACCATGTTCTTTATCAACATTCTCTTTGGTATGATGGGGGCG

18.20 CCTGCCGTCGGTTTTGTCACCATGTTCTTTATCAACATTCTCTTTGGTATGATGGGGGCG

18.6 CCTGCCGTCGGTTTTGTCACCATGTTCTTTATCAACATTCTCTTTGGTATGATGGGGGCG

18.7 CCTGCCGTCGGTTTTGTCACCATGTTCTTTATCAACATTCTCTTTGGTATGATGGGGGCG

************************************************************

APHIS-S CAGATTGTGGAGGCCTTGTTGTCACCGCAGCTTGATACTGAAAATGTCGCTAATATACTT

18.20 CAGATTGTGGAGGCCTTGTTGTCACCGCAGCTTGATACTGAAAATGTCGCTAATATACTT

18.6 CAGATTGTGGAGGCCTTGTTGTCACCGCAGCTTGATACTGAAAATGTCGCTAATATACTT

18.7 CAGATTGTGGAGGCCTTGTTGTCACCGCAGCTTGATACTGAAAATGTCGCTAATATACTT

************************************************************

APHIS-S GACTCCATCTTGCAATTCTTCCCACTCTATAGTCTTGTCACATCTGCCAGACTGTTGAAT

18.20 GACTCCATCTTGCAATTCTTCCCACTCTATAGTCTTGTCACATCTGCCAGACTGTTGAAT

18.6 GACTCCATCTTGCAATTCTTCCCACTCTATAGTCTTGTCACATCTGCCAGACTGTTGAAT

18.7 GACTCCATCTTGCAATTCTTCCCACTCTATAGTCTTGTCACATCTGCCAGACTGTTGAAT

************************************************************

APHIS-S CAGGTGGGACTGCTGGAGTGGTCGTGCCTGCAGAACTGCGAGTACCTGTCCGCAGTGATG

18.20 CAGGTGGGACTGCTGGAGTGGTCGTGCCTGCAGAACTGCGAGTACCTGTCCGCAGTGATG

18.6 CAGGTGGGACTGCTGGAGTGGTCGTGCCTGCAGAACTGCGAGTACCTGTCCGCAGTGATG

18.7 CAGGTGGGACTGCTGGAGTGGTCGTGCCTGCAGAACTGCGAGTACCTGTCCGCAGTGATG

************************************************************

APHIS-S CCCAACTTGACCGAATGCTCCATGGACGTTATGTGCCAGACGTTCTCACAATGTTGCATC

18.20 CCCAACTTGACCGAATGCTCCATGGACGTTATGTGCCAGACGTTCTCACAATGTTGCATC

18.6 CCCAACTTGACCGAATGCTCCATGGACGTTATGTGCCAGACGTTCTCACAATGTTGCATC

18.7 CCCAACTTGACCGAATGCTCCATGGACGTTATGTGCCAGACGTTCTCACAATGTTGCATC

************************************************************

APHIS-S CCAGACGATCCTTGGTTCATGTGGGATCACCCTGGAGTACTCCGCTACATAGTATGCATG

18.20 CCAGACGATCCTTGGTTCATGTGGGATCACCCTGGAGTACTCCGCTACATAGTATGCATG

18.6 CCAGACGATCCTTGGTTCATGTGGGATCACCCTGGAGTACTCCGCTACATAGTATGCATG

18.7 CCAGACGATCCTTGGTTCATGTGGGATCACCCTGGAGTACTCCGCTACATAGTATGCATG

************************************************************

APHIS-S ATCGTCAGTGGAGTTGTCATGTGGTTCGTACTCTTGATCGCCGAGTATCGATTGTTCCAG

18.20 ATCGTCAGTGGAGTTGTCATGTGGTTCGTACTCTTGATCGCCGAGTATCGATTGTTCCAG

18.6 ATCGTCAGTGGAGTTGTCATGTGGTTCGTACTCTTGATCGCCGAGTATCGATTGTTCCAG

18.7 ATCGTCAGTGGAGTTGTCATGTGGTTCGTACTCTTGATCGCCGAGTATCGATTGTTCCAG

************************************************************

APHIS-S AAGGTGATCTACCGGGAAAAGAAAGCTCCTCCAGTTGATGAGAGCGCACTGGACAATGAC

18.20 AAGGTGATCTACCGGGAAAAGAAAGCTCCTCCAGTTGATGAGAGCGCACTGGACAATGAC

18.6 AAGGTGATCTACCGGGAAAAGAAAGCTCCTCCAGTTGATGAGAGCGCACTGGACAATGAC

18.7 AAGGTGATCTACCGGGAAAAGAAAGCTCCTCCAGTTGATGAGAGCGCACTGGACAATGAC

************************************************************

APHIS-S GTGGCGGACGAGGCCAGACACGTGGCGCGAGTTGGAGCAGGAGCAATCCTCGGGCAGCAC

18.20 GTGGCGGACGAGGCCAGACACGTGGCGCGAGTTGGAGCAGGAGCAATCCTCGGGCAGCAC

18.6 GTGGCGGACGAGGCCAGACACGTGGCGCGAGTTGGAGCAGGAGCAATCCTCGGGCAGCAC

18.7 GTGGCGGACGAGGCCAGACACGTGGCGCGAGTTGGAGCAGGAGCAATCCTCGGGCAGCAC

************************************************************

APHIS-S AGCCTAGTAGCAAATGGCCTCACCAAGTATTATGGGAAACACCTTGCAGTCAATCAAGTG

18.20 AGCCTAGTAGCAAATGGCCTCACCAAGTATTATGGGAAACACCTTGCAGTCAATCAAGTG

18.6 AGCCTAGTAGCAAATGGCCTCACCAAGTATTATGGGAAACACCTTGCAGTCAATCAAGTG

18.7 AGCCTAGTAGCAAATGGCCTCACCAAGTATTATGGGAAACACCTTGCAGTCAATCAAGTG

************************************************************

APHIS-S TCATTCACCGTGGGCGACACGGAATGCTTTGGTCTTCTGGGTGTGAACGGCGCCGGTAAG

18.20 TCATTCACCGTGGGCGACACGGAATGCTTTGGTCTTCTGGGTGTGAACGGCGCCGGTAAG

18.6 TCATTCACCGTGGGCGACACGGAATGCTTTGGTCTTCTGGGTGTGAACGGCGCCGGTAAG

18.7 TCATTCACCGTGGGCGACACGGAATGCTTTGGTCTTCTGGGTGTGAACGGCGCCGGTAAG

************************************************************

APHIS-S ACGACCACCTTCAAGATGTTGATGGGAGATGAGACCGTCTCCAGCGGAGATGCCTTCGTG

18.20 ACGACCACCTTCAAGATGTTGATGGGAGATGAGACCGTCTCCAGCGGAGATGCCTTCGTG

18.6 ACGACCACCTTCAAGATGTTGATGGGAGATGAGACCGTCTCCAGCGGAGATGCCTTCGTG

18.7 ACGACCACCTTCAAGATGTTGATGGGAGATGAGACCGTCTCCAGCGGAGATGCCTTCGTG

************************************************************

APHIS-S AGTGGCCATTCTGTCAAGACTAATATCACTCAAGTTTACAAAAATATTGGTTACTGTCCG

18.20 AGTGGCCATTCTGTCAAGACTAATATCACTCAAGTTTACAAAAATATTGGTTACTGTCCG

18.6 AGTGGCCATTCTGTCAAGACTAATATCACTCAAGTTTACAAAAATATTGGTTACTGTCCG

18.7 AGTGGCCATTCTGTCAAGACTAATATCACTCAAGTTTACAAAAATATTGGTTACTGTCCG

************************************************************

APHIS-S CAATTCGAAGCGACATTCGGCGAGCTGACGGGACGCGAGACACTACGGCTGTTCTCGGCG

18.20 CAATTCGAAGCGACATTCGGCGAGCTGACGGGACGCGAGACACTACGGCTGTTCTCGGCG

18.6 CAATTCGAAGCGACATTCGGCGAGCTGACGGGACGCGAGACACTACGGCTGTTCTCGGCG

18.7 CAATTCGAAGCGACATTCGGCGAGCTGACGGGACGCGAGACACTACGGCTGTTCTCGGCG

************************************************************

APHIS-S CTGCGAGGGTTGCCAGTGCGAGGCGCCACGCTCCACGCGGAGGCCTTAGCACATGCTCTT

18.20 CTGCGAGGGTTGCCAGTGCGAGGCGCCACGCTCCACGCGGAGGCCTTAGCACATGCTCTT

18.6 CTGCGAGGGTTGCCAGTGCGAGGCGCCACGCTCCACGCGGAGGCCTTAGCACATGCTCTT

18.7 CTGCGAGGGTTGCCAGTGCGAGGCGCCACGCTCCACGCGGAGGCCTTAGCACATGCTCTT

************************************************************

APHIS-S GGTTTCTATAAGCATCTTGATAAAAGGGTGGACCACTACTCTGGTGGCAACAAGCGCAAG

18.20 GGTTTCTATAAGCATCTTGATAAAAGGGTGGACCACTACTCTGGTGGCAACAAGCGCAAG

18.6 GGTTTCTATAAGCATCTTGATAAAAGGGTGGACCACTACTCTGGTGGCAACAAGCGCAAG

18.7 GGTTTCTATAAGCATCTTGATAAAAGGGTGGACCACTACTCTGGTGGCAACAAGCGCAAG

************************************************************

APHIS-S TTGAGCACGGCTGTGGCGTTGCTGGGGCGCACGCGGCTTATATTCGTCGACGAACCCACT

18.20 TTGAGCACGGCTGTGGCGTTGCTGGGGCGCACGCGGCTTATATTCGTCGACGAACCCACT

18.6 TTGAGCACGGCTGTGGCGTTGCTGGGGCGCACGCGGCTTATATTCGTCGACGAACCCACT

18.7 TTGAGCACGGCTGTGGCGTTGCTGGGGCGCACGCGGCTTATATTCGTCGACGAACCCACT

************************************************************

APHIS-S ACTGGAGTCGATCCTGCTGCTAAGAGACAGATGTGGAACGCGGTTCGAGAAGCTCGCCGG

18.20 ACTGGAGTCGATCCTGCTGCTAAGAGACAGATGTGGAACGCGGTTCGAGAAGCTCGCCGG

18.6 ACTGGAGTCGATCCTGCTGCTAAGAGACAGATGTGGAACGCGGTTCGAGAAGCTCGCCGG

18.7 ACTGGAGTCGATCCTGCTGCTAAGAGACAGATGTGGAACGCGGTTCGAGAAGCTCGCCGG

************************************************************

APHIS-S TCGGGTCGTGGTGTGGTGCTGACATCACACAGCATGGAGGAGTGTGAGGCTCTGTGCTCG

18.20 TCGGGTCGTGGTGTGGTGCTGACATCACACAGCATGGAGGAGTGTGAGGCTCTGTGCTCG

18.6 TCGGGTCGTGGTGTGGTGCTGACATCACACAGCATGGAGGAGTGTGAGGCTCTGTGCTCG

18.7 TCGGGTCGTGGTGTGGTGCTGACATCACACAGCATGGAGGAGTGTGAGGCTCTGTGCTCG

************************************************************

APHIS-S CGGCTCACAATCATGGTCAACGGACAGTTCCAGTGCCTCGGCACGCCGCAACATTTAAAG

18.20 CGGCTCACAATCATGGTCAACGGACAGTTCCAGTGCCTCGGCACGCCGCAACATTTAAAG

18.6 CGGCTCACAATCATGGTCAACGGACAGTTCCAGTGCCTCGGCACGCCGCAACATTTAAAG

18.7 CGGCTCACAATCATGGTCAACGGACAGTTCCAGTGCCTCGGCACGCCGCAACATTTAAAG

************************************************************

APHIS-S AATAAGTTCTCTGAAGGTTTCACATTGACAATTAAAATTAAAGTGGACGACGAGACGAAG

18.20 AATAAGTTCTCTGAAGGTTTCACATTGACAATTAAAATTAAAGTGGACGACGAGACGAAG

18.6 AATAAGTTCTCTGAAGGTTTCACATTGACAATTAAAATTAAAGTGGACGACGAGACGAAG

18.7 AATAAGTTCTCTGAAGGTTTCACATTGACAATTAAAATTAAAGTGGACGACGAGACGAAG

************************************************************

APHIS-S ACTGTACGGCCTGAAGTCTGCGATGCTGTGAAGCATTACGTCAGTACCAACTTCAGAGAG

18.20 ACTGTACGGCCTGAAGTCTGCGATGCTGTGAAGCATTACGTCAGTACCAACTTCAGAGAG

18.6 ACTGTACGGCCTGAAGTCTGCGATGCTGTGAAGCATTACGTCAGTACCAACTTCAGAGAG

18.7 ACTGTACGGCCTGAAGTCTGCGATGCTGTGAAGCATTACGTCAGTACCAACTTCAGAGAG

************************************************************

APHIS-S CCGAAGATTATGGAGGAGTACCAGGGTCTGTTAACATACTATTTGCCAGACAAGTCGGTG

18.20 CCGAAGATTATGGAGGAGTACCAGGGTCTGTTAACATACTATTTGCCAGACAAGTCGGTG

18.6 CCGAAGATTATGGAGGAGTACCAGGGTCTGTTAACATACTATTTGCCAGACAAGTCGGTG

18.7 CCGAAGATTATGGAGGAGTACCAGGGTCTGTTAACATACTATTTGCCAGACAAGTCGGTG

************************************************************

APHIS-S GCGTGGTCCAGAATGTTCGGCATAATGGAGGCGGCCAAACGCGACCTCCCCGTCGAAGAC

18.20 GCGTGGTCCAGAATGTTCGGCATAATGGAGGCGGCCAAACGCGACCTCCCCGTCGAAGAC

18.6 GCGTGGTCCAGAATGTTCGGCATAATGGAGGCGGCCAAACGCGACCTCCCCGTCGAAGAC

18.7 GCGTGGTCCAGAATGTTCGGCATAATGGAGGCGGCCAAACGCGACCTCCCCGTCGAAGAC

************************************************************

APHIS-S TACAGCATATCACAAACTACCCTCGAGCAGATATTCCTACAGTTCACAAAGTATCAACAT

18.20 TACAGCATATCACAAACTACCCTCGAG---------------------------------

18.6 TACAGCATATCACAAACTACCCTCGAG---------------------------------

18.7 TACAGCATATCACAAACTACCCTCGAG---------------------------------

***************************

APHIS-S GAAGCACAACAGACATAA

18.20 ------------------

18.6 ------------------

18.7 ------------------

**Supplementary Figure S5. Alignment of translated *PgABCA2* amino acid sequences from CRISPR-R2 G_4_ survivors on 3 μg Cry2Ab per mL lacking gDNA mutations corresponding to sgRNA target sites.** Translated *PgABCA2* cDNA sequences from five CRISPR-R2 G_4_ survivors (without gDNA mutations in sgRNA target regions) were aligned with *PgABCA2* (AXZ96960.1) from APHIS-S using Clustal MUSCLE (<https://www.ebi.ac.uk/Tools/msa/muscle/>). Stars show amino acids conserved in all of the sequences.

5B.8 MRARGERXEAGSWVKFRLLMWKNFVQQLRHPVQTAAELLLPVLTMSLVLVLRSQIDPEVL

3.1 MRARGERKEAGSWVKFRLLMWKNFVQQLRHPVQTAAELLLPVLTMSLVLVLRSQIDPEVL

9B.16 MRARGERKEAGSWVKFRLLMWKNFVQQLRHPVQTAAELLLPVLTMSLVLVLRSQIDPEVL

9B.14 MRARGERKEAGSWVKFRLLMWKNFVQQLRHPVQTAAELLLPVLTMSLVLVLRSQIDPEVL

9B.12 MRARGERKEAGSWVKFRLLMWKNFVQQLRHPVQTAAELLLPVLTMSLVLVLRSQIDPEVL

9A.13 MRARGERKEAGSWVKFRLLMWKNFVQQLRHPVQTAAELLLPVLTMSLVLVLRSQIDPEVL

9A.10 MRARGERKEAGSWVKFRLLMWKNFVQQLRHPVQTAAELLLPVLTMSLVLVLRSQIDPEVL

9A.9 MRARGERKEAGSWVKFRLLMWKNFVQQLRHPVQTAAELLLPVLTMSLVLVLRSQIDPEVL

9A.8 MRARGERKEAGSWVKFRLLMWKNFVQQLRHPVQTAAELLLPVLTMSLVLVLRSQIDPEVL

APHIS-S MRARGERKEAGSWVKFRLLMWKNFVQQLRHPVQTAAELLLPVLTMSLVLVLRSQIDPEVL

5A.7 MRARGERKEAGSWVKFRLLMWKNFVQQLRHPVQTAAELLLPVLTMSLVLVLRSQIDPEVL

5A.11 MRARGERKEAGSWVKFRLLMWKNFVQQLRHPVQTAAELLLPVLTMSLVLVLRSQIDPEVL

10.29 MRARGERKEAGSWVKFRLLMWKNFVQQLRHPVQTAAELLLPVLTMSLVLVLRSQIDPEVL

18.7 MRARGERKEAGSWVKFRLLMWKNFVQQLRHPVQTAAELLLPVLTMSLVLVLRSQIDPEVL

18.6 MRARGERKEAGSWVKFRLLMWKNFVQQLRHPVQTAAELLLPVLTMSLVLVLRSQIDPEVL

10.19 MRARGERKEAGSWVKFRLLMWKNFVQQLRHPVQTAAELLLPVLTMSLVLVLRSQIDPEVL

10.1 MRARGERKEAGSWVKFRLLMWKNFVQQLRHPVQTAAELLLPVLTMSLVLVLRSQIDPEVL

10.5 MRARGERKEAGSWVKFRLLMWKNFVQQLRHPVQTAAELLLPVLTMSLVLVLRSQIDPEVL

10.11 MRARGERKEAGSWVKFRLLMWKNFVQQLRHPVQTAAELLLPVLTMSLVLVLRSQIDPEVL

10.21 MRARGERKEAGSWVKFRLLMWKNFVQQLRHPVQTAAELLLPVLTMSLVLVLRSQIDPEVL

10.24 MRARGERKEAGSWVKFRLLMWKNFVQQLRHPVQTAAELLLPVLTMSLVLVLRSQIDPEVL

10.22 MRARGERKEAGSWVKFRLLMWKNFVQQLRHPVQTAAELLLPVLTMSLVLVLRSQIDPEVL

5B.6 MRARGERKEAGSWVKFRLLMWKNFVQQLRHPVQTAAELLLPVLTMSLVLVLRSQIDPEVL

5B.4 MRARGERKEAGSWVKFRLLMWKNFVQQLRHPVQTAAELLLPVLTMSLVLVLRSQIDPEVL

3.3 MRARGERKEAGSWVKFRLLMWKNFVQQLRHPVQTAAELLLPVLTMSLVLVLRSQIDPEVL

3.15 MRARGERKEAGSWVKFRLLMWKNFVQQLRHPVQTAAELLLPVLTMSLVLVLRSQIDPEVL

5A.12 MRARGERKEAGSWVKFRLLMWKNFVQQLRHPVQTAAELLLPVLTMSLVLVLRSQIDPEVL

18.20 MRARGERKEAGSWVKFRLLMWKNFVQQLRHPVQTAAELLLPVLTMSLVLVLRSQIDPEVL

3.2 MRARGERKEAGSWVKFRLLMWKNFVQQLRHPVQTAAELLLPVLTMSLVLVLRSQIDPEVL

******* ****************************************************

5B.8 ETRTYPPIPAHTLNYSVTVLNAVG------------------------------------

3.1 ETRTYLPIPAHTLNYSVTVLGGMNLTRMSMAFSLENAVAV--------------------

9B.16 ETRTYPPIPAHTLNYSVTVLGGMNLTRMSMAFSPENAVYA--------------------

9B.14 ETRTYPPIPAHTLNYSVTVLGGMNLTRMSMAFSPENAVYA--------------------

9B.12 ETRTYPPIPAHTLNYSVTVLGGMNLTRMSMAFSPENAVYA--------------------

9A.13 ETRTYPPIPAHTLNYSVTVLGGMNLTRMSMAFSPENAVYA--------------------

9A.10 ETRTYPPIPAHTLNYSVTVLGGMNLTRMSMAFSPENAVYA--------------------

9A.9 ETRTYPPIPAHTLNYSVTVLGGMNLTRMSMAFSPENAVYA--------------------

9A.8 ETRTYPPIPAHTLNYSVTVLGGMNLTRMSMAFSPENAVYA--------------------

APHIS-S ETRTYPPIPAHTLNYSVTVLGGMNLTRMSMAFSPENAVLRDVVSSATTKLLLKNMRDQVL

5A.7 ETRTYPPIPAHTLNYSVTVLGGMNLTRMSMAFSPENAVLRDVVSSATTKLLLKNMRDQVL

5A.11 ETRTYPPIPAHTLNYSVTVLGGMNLTRMSMAFSPENAVLRDVVSSATTKLLLKNMRDQVL

10.29 ETRTYPPIPAHTLNYSVTVLGGMNLTRMSMAFSPENAVLRDVVSSATTKLLLKNMRDQVL

18.7 ETRTYPPIPAHTLNYSVTVLGGMNLTRMSMAFSPENAVLRDVVSSATTKLLLKNMRDQVL

18.6 ETRTYPPIPAHTLNYSVTVLGGMNLTRMSMAFSPENAVLRDVVSSATTKLLLKNMRDQVL

10.19 ETRTYPPIPAHTLNYSVTVLGGMNLTRMSMAFSPENAVLRDVVSSATTKLLLKNMRDQVL

10.1 ETRTYPPIPAHTLNYSVTVLGGMNLTRMSMAFSPENAVLRDVVSSATTKLLLKNMRDQVL

10.5 ETRTYPPIPAHTLNYSVTVLGGMNLTRMSMAFSPENAVLRDVVSSATTKLLLKNMRDQVL

10.11 ETRTYPPIPAHTLNYSVTVLGGMNLTRMSMAFSPENAVLRDVVSSATTKLLLKNMRDQVL

10.21 ETRTYPPIPAHTLNYSVTVLGGMNLTRMSMAFSPENAVLRDVVSSATTKLLLKNMRDQVL

10.24 ETRTYPPIPAHTLNYSVTVLGGMNLTRMSMAFSPENAVLRDVVSSATTKLLLKNMRDQVL

10.22 ETRTYPPIPAHTLNYSVTVLGGMNLTRMSMAFSPENAVLRDVVSSATTKLLLKNMRDQVL

5B.6 ETRTYPPIPAHTLNYSVTVLNAVG------------------------------------

5B.4 ETRTYPPIPAHTLNYSVTVLNAVG------------------------------------

3.3 ETRTYPPIPAHTLNYSVTVLGGMNLTRMSMAFSLENAVAV--------------------

3.15 ETRTYPPIPAHTLNYSVTVL----------------------------------------

5A.12 ETRTYPPIPAHTLNYSVTVLGGMNLTRMSMAFSPENAVLRDVVSSATTKLLLKNMRDQVL

18.20 ETRTYPPIPAHTLNYSVTVLGGMNLTRMSMAFSPENAVLRDVVSSATTKLLLKNMRDQVL

3.2 ETRTYPPIPAHTLNYSVTVL----------------------------------------

***** **************

5B.8 ------------------------------------------------------------

3.1 ------------------------------------------------------------

9B.16 ------------------------------------------------------------

9B.14 ------------------------------------------------------------

9B.12 ------------------------------------------------------------

9A.13 ------------------------------------------------------------

9A.10 ------------------------------------------------------------

9A.9 ------------------------------------------------------------

9A.8 ------------------------------------------------------------

APHIS-S PIIEALPIEIPPGLVNSSQVYEIVKLFVDENVVTGYNSSAAMRGIYAEEEATRRVIAGIE

5A.7 PIIEALPIEIPPGLVNSSQVYEIVKLFVDENVVTGYNSSAAMRGIYAEEEATRRVIAGIE

5A.11 PIIEALPIEIPPGLVNSSQVYEIVKLFVDENVVTGYNSSAAMRGIYAEEEATRRVIAGIE

10.29 PIIEALPIEIPPGLVNSSQVYEIVKLFVDENVVTGYNSSAAMRGIYAEEEATRRVIAGIE

18.7 PIIEALPIEIPPGLVNSSQVYEIVKLFVDENVVTGYNSSAAMRGIYAEEEATRRVIAGIE

18.6 PIIEALPIEIPPGLVNSSQVYEIVKLFVDENVVTGYNSSAAMRGIYAEEEATRRVIAGIE

10.19 PIIEALPIEIPPGLVNSSQVYEIVKLFVDENVVTGYNSSAAMRGIYAEEEATRRVIAGIE

10.1 PIIEALPIEIPPGLVNSSQVYEIVKLFVDENVVTGYNSSAAMRGIYAEEEATRRVIAGIE

10.5 PIIEALPIEIPPGLVNSSQVYEIVKLFVDENVVTGYNSSAAMRGIYAEEEATRRVIAGIE

10.11 PIIEALPIEIPPGLVNSSQVYEIVKLFVDENVVTGYNSSAAMRGIYAEEEATRRVIAGIE

10.21 PIIEALPIEIPPGLVNSSQVYEIVKLFVDENVVTGYNSSAAMRGIYAEEEATRRVIAGIE

10.24 PIIEALPIEIPPGLVNSSQVYEIVKLFVDENVVTGYNSSAAMRGIYAEEEATRRVIAGIE

10.22 PIIEALPIEIPPGLVNSSQVYEIVKLFVDENVVTGYNSSAAMRGIYAEEEATRRVIAGIE

5B.6 ------------------------------------------------------------

5B.4 ------------------------------------------------------------

3.3 ------------------------------------------------------------

3.15 ------------------------------------------------------------

5A.12 PIIEALPIEIPPGLVNSSQVYEIVKLFVDENVVTGYNSSAAMRGIYAEEEATRRVIAGIE

18.20 PIIEALPIEIPPGLVNSSQVYEIVKLFVDENVVTGYNSSAAMRGIYAEEEATRRVIAGIE

3.2 ------------------------------------------------------------

5B.8 ------------------------------------------------------------

3.1 ------------------------------------------------------------

9B.16 ------------------------------------------------------------

9B.14 ------------------------------------------------------------

9B.12 ------------------------------------------------------------

9A.13 ------------------------------------------------------------

9A.10 ------------------------------------------------------------

9A.9 ------------------------------------------------------------

9A.8 ------------------------------------------------------------

APHIS-S FDDSLREITELPLDLSYALRFPERPRLNSFFMTGGRTWRTDNVFPMFEVPGPRFPYSWEG

5A.7 FDDSLREITELPLDLSYALRFPERPRLNSFFMTGGRTWRTDNVFPMFEVPGPRFPYSWEG

5A.11 FDDSLREITELPLDLSYALRFPERPRLNSFFMTGGRTWRTDNVFPMFEVPGPRFPYSWEG

10.29 FDDSLREITELPLDLSYALRFPERPRLNSFFMTGGRTWRTDNVFPMFEVPGPRFPYSWEG

18.7 FDDSLREITELPLDLSYALRFPERPRLNSFFMTGGRTWRTDNVFPMFEVPGPRFPYSWEG

18.6 FDDSLREITELPLDLSYALRFPERPRLNSFFMTGGRTWRTDNVFPMFEVPGPRFPYSWEG

10.19 FDDSLREITELPLDLSYALRFPERPRLNSFFMTGGRTWRTDNVFPMFEVPGPRFPYSWEG

10.1 FDDSLREITELPLDLSYALRFPERPRLNSFFMTGGRTWRTDNVFPMFEVPGPRFPYSWEG

10.5 FDDSLREITELPLDLSYALRFPERPRLNSFFMTGGRTWRTDNVFPMFEVPGPRFPYSWEG

10.11 FDDSLREITELPLDLSYALRFPERPRLNSFFMTGGRTWRTDNVFPMFEVPGPRFPYSWEG

10.21 FDDSLREITELPLDLSYALRFPERPRLNSFFMTGGRTWRTDNVFPMFEVPGPRFPYSWEG

10.24 FDDSLR------------------------------------------------------

10.22 FDDSLREITELPLDLSYALRFPERPRLNSFFMTGGRTWRTDNVFPMFEVPGXRFPYSWEG

5B.6 ------------------------------------------------------------

5B.4 ------------------------------------------------------------

3.3 ------------------------------------------------------------

3.15 ------------------------------------------------------------

5A.12 FDDSLREITELPLDLSYALRFPERPRLNSFFMTGGRTWRTDNVFPMFEVPGPRFPYSWEG

18.20 FDDSLREITELPLDLSYALRFPERPRLNSFFMTGGRTWRTDNVFPMFEVPGPRFPYSWEG

3.2 ------------------------------------------------------------

5B.8 ------------------------------------------------------------

3.1 ------------------------------------------------------------

9B.16 ------------------------------------------------------------

9B.14 ------------------------------------------------------------

9B.12 ------------------------------------------------------------

9A.13 ------------------------------------------------------------

9A.10 ------------------------------------------------------------

9A.9 ------------------------------------------------------------

9A.8 ------------------------------------------------------------

APHIS-S GNDPGYVNEMFIALQHMISSELVSKVAGVNLDFDVHIQRYPHPAYIMDLAKEALQFLFPS

5A.7 GNDPGYVNEMFIALQHMISSELVSKVAGVNLDFDVHIQRYPHPAYIMDLAKEALQFLFPS

5A.11 GNDPGYVNEMFIALQHMISSELVSKVAGVNLDFDVHIQRYPHPAYIMDLAKEALQFLFPS

10.29 GNDPGYVNEMFIALQHMISSELVSKVAGVNLDFDVHIQRYPHPAYIMDLAKEALQFLFPS

18.7 GNDPGYVNEMFIALQHMISSELVSKVAGVNLDFDVHIQRYPHPAYIMDLAKEALQFLFPS

18.6 GNDPGYVNEMFIALQHMISSELVSKVAGVNLDFDVHIQRYPHPAYIMDLAKEALQFLFPS

10.19 GNDPGYVNEMFIALQHMISSELVSKVAGVNLDFDVHIQRYPHPAYIMDLAKEALQFLFPS

10.1 GNDPGYVNEMFIALQHMISSELVSKVAGVNLDFDVHIQRYPHPAYIMDLAKEALQFLFPS

10.5 GNDPGYVNEMFIALQHMISSELVSKVAGVNLDFDVHIQRYPHPAYIMDLAKEALQFLFPS

10.11 GNDPGYVNEMFIALQHMISSELVSKVAGVNLDFDVHIQRYPHPAYIMDLAKEALQFLFPS

10.21 GNDPGYVNEMFIALQHMISSELVSKVAGVNLDFDVHIQRYPHPAYIMDLAKEALQFLFPS

10.24 ------------------------------------------------------------

10.22 GNDPGYVNEMFIALQHMISSELVSKVAGVNLDFDVHIQRYPHPAYIMDLAKEALQFLFPS

5B.6 ------------------------------------------------------------

5B.4 ------------------------------------------------------------

3.3 ------------------------------------------------------------

3.15 ------------------------------------------------------------

5A.12 GNDPGYVNEMFIALQHMISSELVSEVAGVNLDFDVHIQRYPHPAYIMDLAKEALQFLFPS

18.20 GNDPGYVNEMFIALQHMISSELVSKVAGVNLDFDVHIQRYPHPAYIMDLAKEALQFLFPS

3.2 ------------------------------------------------------------

5B.8 ------------------------------------------------------------

3.1 ------------------------------------------------------------

9B.16 --------------------------------GRLKWFYW--------------------

9B.14 --------------------------------GRLKWFYW--------------------

9B.12 --------------------------------GRLKWFYW--------------------

9A.13 --------------------------------GRLKWFYW--------------------

9A.10 --------------------------------GRLKWFYW--------------------

9A.9 --------------------------------GRLKWFYW--------------------

9A.8 --------------------------------GRLKWFYW--------------------

APHIS-S FIMISFSYTAINIIRSVTVEKEMQLKETMKIMGLPTWLHWMAWFFKQFIYLLIASVLILV

5A.7 FIMISFSYTAINIIRSVTVEKEMQLKETMKIMGLPTWLHWMAWFFKQFIYLLIASVLILV

5A.11 FIMISFSYTAINIIRSVTVEKEMQLKETMKIMGLPTWLHWMAWFFKQFIYLLIASVLILV

10.29 FIMISFSYTAINIIRSVTVEKEMQLKETMKIMGLPTWLHWMAWFFKQFIYLLIASVLILV

18.7 FIMISFSYTAINIIRSVTVEKEMQLKETMKIMGLPTWLHWMAWFFKQFIYLLIASVLILV

18.6 FIMISFSYTAINIIRSVTVEKEMQLKETMKIMGLPTWLHWMAWFFKQFIYLLIASVLILV

10.19 FIMISFSYTAINIIRSVTVEKEMQLKETMKIMGLPTWLHWMAWFFKQFIYLLIASVLILV

10.1 FIMISFSYTAINIIRSVTVEKEMQLKETMKIMGLPTWLHW--------------------

10.5 FIMISFSYTAINIIRSVTVEKEMQLKETMKIMGLPTWLHWMAWFFKQFIYLLIASVLILV

10.11 FIMISFSYTAINIIRSVTVEKEMQLKETMKIMGLPTWLHWMAWFFKQFIYLLIASVLILV

10.21 FIMISFSYTAINIIRSVTVEKEMQLKETMKIMGLPTWLHW--------------------

10.24 ------------------------------------------------------------

10.22 FIMISFSYTAINIIRSVTVEKEMQLKETMKIMGLPTWLHW--------------------

5B.6 ------------------------------------------------------------

5B.4 ------------------------------------------------------------

3.3 ------------------------------------------------------------

3.15 ---------------------------------QMTYQRW--------------------

5A.12 FIMISFSYTAINIIRSVTVEKEMQLKETMKIMGLPTWLHWMAWFFKQFIYLLIASVLILV

18.20 FIMISFSYTAINIIRSVTVEKEMQLKETMKIMGLPTWLHWMAWFFKQFIYLLIASVLILV

3.2 ------------------------------------------------------------

5B.8 ------------------------------------------------------------

3.1 ------------------------------------------------------------

9B.16 ------------------------------------------------------------

9B.14 ------------------------------------------------------------

9B.12 ------------------------------------------------------------

9A.13 ------------------------------------------------------------

9A.10 ------------------------------------------------------------

9A.9 ------------------------------------------------------------

9A.8 ------------------------------------------------------------

APHIS-S ILKVNWFTTEEGFSDYAVFTNTPWTVLFFFLTLYLTCTIFFCFMISGFFSKASTAALFGG

5A.7 ILKVNWFTTEEGFSDYAVFTNTPWTVLFFFLTLYLTCTIFFCFMISGFFSKASTAALFGG

5A.11 ILKVNWFTTEEGFSDYAVFTNTPWTVLFFFLTLYLTCTIFFCFMISGFFSKASTAALFGG

10.29 ILKVNWFTTEEGFSDYAVFTNTPWTVLFFFLTLYLTCTIFFCFMISGFFSKASTAALFGG

18.7 ILKVNWFTTEEGFSDYAVFTNTPWTVLFFFLTLYLTCTIFFCFMISGFFSKASTAALFGG

18.6 ILKVNWFTTEEGFSDYAVFTNTPWTVLFFFLTLYLTCTIFFCFMISGFFSKASTAALFGG

10.19 ILKVNWFTTEEGFSDYAVFTNTPWTVLFFFLTLYLTCTIFFCFMISGFFSKASTAALFGG

10.1 ------------------------------------------------------------

10.5 ILK---------------------------------------------------------

10.11 ILK---------------------------------------------------------

10.21 ------------------------------------------------------------

10.24 ------------------------------------------------------------

10.22 ------------------------------------------------------------

5B.6 ------------------------------------------------------------

5B.4 ------------------------------------------------------------

3.3 ------------------------------------------------------------

3.15 ------------------------------------------------------------

5A.12 ILKVNWFTTEEGFSDYAVFTNTPWTVLFFFLTLYLTCTIFFCFMISGFFSKASTAALFGG

18.20 ILKVNWFTTEEGFSDYAVFTNTPWTVLFFFLTLYLTCTIFFCFMISGFFSKASTAALFGG

3.2 ------------------------------------------------------------

5B.8 ------------------------------------------------------------

3.1 ------------------------------------------------------------

9B.16 ------------------------------------------------------------

9B.14 ------------------------------------------------------------

9B.12 ------------------------------------------------------------

9A.13 ------------------------------------------------------------

9A.10 ------------------------------------------------------------

9A.9 ------------------------------------------------------------

9A.8 ------------------------------------------------------------

APHIS-S VIWFLTYIPAFLLAMDVNMSTSLQAVTCLMLNSAMSYGFQLLLARESTGGMQWGDFMTSP

5A.7 VIWFLTYIPAFLLAMDVNMSTSLQAVTCLMLNSAMSYGFQLLLARESTGGMQWGDFMTSP

5A.11 VIWFLTYIPAFLLAMDVNMSTSLQAVTCLMLNSAMSYGFQLLLARESTGGMQWGDFMTSP

10.29 VIWFLTYIPAFLLAMDVNMSTSLQAVTCLMLNSAMSYGFQLLLARESTGGMQWGDFMTSP

18.7 VIWFLTYIPAFLLAMDVNMSTSLQAVTCLMLNSAMSYGFQLLLARESTGGMQWGDFMTSP

18.6 VIWFLTYIPAFLLAMDVNMSTSLQAVTCLMLNSAMSYGFQLLLARESTGGMQWGDFMTSP

10.19 VIWFLTYIPAFLLAMDVNMSTSLQAVTCLMLNSAMSYGFQLLLARESTGGMQWGDFMTSP

10.1 ------------------------------------------------------------

10.5 ------------------------------------------------------------

10.11 ------------------------------------------------------------

10.21 ------------------------------------------------------------

10.24 ------------------------------------------------------------

10.22 ------------------------------------------------------------

5B.6 ------------------------------------------------------------

5B.4 ------------------------------------------------------------

3.3 ------------------------------------------------------------

3.15 ------------------------------------------------------------

5A.12 VIWFLTYIPAFLLAMDVNMSTSLQAVTCLMLNSAMSYGFQLLLARESTGGMQWGDFMTSP

18.20 VIWFLTYIPAFLLAMDVNMSTSLQAVTCLMLNSAMSYGFQLLLARESTGGMQWGDFMTSP

3.2 ------------------------------------------------------------

5B.8 ------------------------------------------------------------

3.1 ------------------------------------------------------------

9B.16 ------------------------------------------------------------

9B.14 ------------------------------------------------------------

9B.12 ------------------------------------------------------------

9A.13 ------------------------------------------------------------

9A.10 ------------------------------------------------------------

9A.9 ------------------------------------------------------------

9A.8 ------------------------------------------------------------

APHIS-S ATDSSRFVFGHVVIMMALNCVLYMLIALYLEQVLPGPYGTPKPWYFFVQRQFWCSSKTTH

5A.7 ATDSSRFVFGHVVIMMALNCVLYMLIALYLEQVLPGPYGTPKPWYFFVQRQFWCSSKTTH

5A.11 ATDSSRFVFGHVVIMMALNCVLYMLIALYLEQVLPGPYGTPKPWYFFVQRQFWCSSKTTH

10.29 ATDSSRFVFGHVVIMMALNCVLYMLIALYLEQVLPGPYGTPKPWYFFVQRQFWCSSKTTH

18.7 ATDSSRFVFGHVVIMMALNCVLYMLIALYLEQVLPGPYGTPKPWYFFVQRQFWCSSKTTH

18.6 ATDSSRFVFGHVVIMMALNCVLYMLIALYLEQVLPGPYGTPKPWYFFVQRQFWCSSKTTH

10.19 ATDSSRFVFGHVVIMMALNCVLYMLIALYLEQVLPGPYGTPKPWYFFVQRQFWCSSKTTH

10.1 ------------------------------------------------------------

10.5 ------------------------------------------------------------

10.11 ------------------------------------------------------------

10.21 ------------------------------------------------------------

10.24 ------------------------------------------------------------

10.22 ------------------------------------------------------------

5B.6 ------------------------------------------------------------

5B.4 ------------------------------------------------------------

3.3 ------------------------------------------------------------

3.15 ------------------------------------------------------------

5A.12 ATDSSRFVFGHVVIMMALNCVLYMLIALYLEQVLPGPYGTPKPWYFFVQRQFWCSSKTTH

18.20 ATDSSRFVFGHVVIMMALNCVLYMLIALYLEQVLPGPYGTPKLWYFFVQRQFWCSSKTTH

3.2 ------------------------------------------------------------

5B.8 ------------------------------------------------------------

3.1 ------------------------------------------------------------

9B.16 --------TSPLLAWTRPHVVPCGTCCRERRKVDR-------------------------

9B.14 --------TSPLLAWTRPHVVPCGTCCRERRKVDR-------------------------

9B.12 --------TSPLLAWTRPHVVPCGTCCRERRKVDR-------------------------

9A.13 --------TSPLLAWTRPHVVPCGTCCRERRKVDR-------------------------

9A.10 --------TSPLLAWTRPHVVPCGTCCRERRKVDR-------------------------

9A.9 --------TSPLLAWTRPHVVPCGTCCRERRKVDR-------------------------

9A.8 --------TSPLLAWTRPHVVPCGTCCRE-------------------------------

APHIS-S DIGTDNSDTSSLTKESDPTDLPIGVKIQNLKKVYGSNVAVNNLSLNIYDDQITVLLGHNG

5A.7 DIGTDNSDTSSLTKESDPTDLPIGVKIQNLKKVYGSNVAVNNLSLNIYDDQITVLLGHNG

5A.11 DIGTDNSDTSSLTKESDPTDLPIGVKIQNLKKVYGSNVAVNNLSLNIYDDQITVLLGHNG

10.29 DIGTDNSDTSSLTKESDPTDLPIGVKIQNLKKVYGSNVAVNNLSLNIYDDQITALLGHNG

18.7 DIGTDNSDTSSLTKESDPTDLPIGVKIQNLKKVYGSNVAVNNLSLNIYDDQITVLLGHNG

18.6 DIGTDNSDTSSLTKESDPTDLPIGVKIQNLKKVYGSNVAVNNLSLNIYDDQITVLLGHNG

10.19 DIGTDNSDTSSLTKESDPTDLPIGVKIQNLKKVYGSNVAVNNLSLNI-------------

10.1 ------------------------------------------------------------

10.5 ------------------------------------------------------------

10.11 ------------------------------------------------------------

10.21 ------------------------------------------------------------

10.24 ------------------------------------------------------------

10.22 ------------------------------------------------------------

5B.6 ------------------------------------------------------------

5B.4 ------------------------------------------------------------

3.3 ------------------------------------------------------------

3.15 ------------------------------------------------------------

5A.12 DIGTDNSDTSSLTKESDPTDLPIGVKIQNLKKVYGSNVAVNNLSLNIYDDQITVLLGHNG

18.20 DIGTDNSDTSSLTKESDPTDLPIGVKIQNLKKVYGSNVAVNNLSLNIYDDQITVLLGHNG

3.2 ------------------------------------------------------------

5B.8 ------------------------------------------------------------

3.1 ------------------------------------------------------------

9B.16 ------------------------------------------------------------

9B.14 ------------------------------------------------------------

9B.12 ------------------------------------------------------------

9A.13 ------------------------------------------------------------

9A.10 ------------------------------------------------------------

9A.9 ------------------------------------------------------------

9A.8 ------------------------------------------------------------

APHIS-S AGKSTTISMLTGNVDITSGSVTVAGYDIEKQTSSARSHIGLCPQHNVLFNELTVKEHLQF

5A.7 AGKSTTISMLTGNVDITSGSVTVAGYDIEKQTSSARSHIGLCPQHNVLFNELTVKEHLQF

5A.11 AGKSTTISMLTGNVDITSGSVTVAGYDIEKQTSSARSHIGLCPQHNVLFNELTVKEHLQF

10.29 AGKSTTISMLTGNVDITSGSVTVAGYDIEKQTSSARSHIGLCPQHNVLFNELTVKEHLQF

18.7 AGKSTTISMLTGNVDITSGSVTVAGYDIEKQTSSARSHIGLCPQHNVLFNELTVKEHLQF

18.6 AGKSTTISMLTGNVDITSGSVTVAGYDIEKQTSSARSHIGLCPQHNVLFNELTVKEHLQF

10.19 ------------------------------------------------------------

10.1 ------------------------------------------------------------

10.5 ------------------------------------------------------------

10.11 ------------------------------------------------------------

10.21 ------------------------------------------------------------

10.24 ------------------------------------------------------------

10.22 ------------------------------------------------------------

5B.6 ------------------------------------------------------------

5B.4 ------------------------------------------------------------

3.3 ------------------------------------------------------------

3.15 ------------------------------------------------------------

5A.12 AGKSTTISMLTGNVDITSGSVTVAGYDIEKQTSSARSHIGLCPQHNVLFNELTVKEHLQF

18.20 AGKSTTISMLTGNVDITSGSVTVAGYDIEKQTSSARSHIGLCPQHNVLFNELTVKEHLQF

3.2 ------------------------------------------------------------

5B.8 ------------------------------------------------------------

3.1 ------------------------------------------------------------

9B.16 ------------------------------------------------------------

9B.14 ------------------------------------------------------------

9B.12 ------------------------------------------------------------

9A.13 ------------------------------------------------------------

9A.10 ------------------------------------------------------------

9A.9 ------------------------------------------------------------

9A.8 ------------------------------------------------------------

APHIS-S FSRLKGFSGKELDEEIETLIEKLELQEKRDYQSAGLSGGQKRRLGVGVALCGAAKVVLLD

5A.7 FSRLKGFSGKELDEEIETLIEKLELQEKRDYQSAGLSGGQKRRLGVGVALCGAAKVVLLD

5A.11 FSRLKGFSGKELDEEIETLIEKLELQEKRDYQSAGLSGGQKRRLGVGVALCGAAKVVLLD

10.29 FSRLKGFSGKELDEEIETLIEKLELQEKRDYQSAGLSGGQKRRLGVGVALCGAAKVVLLD

18.7 FSRLKGFSGKELDEEIETLIEKLELQEKRDYQSAGLSGGQKRRLGVGVALCGAAKVVLLD

18.6 FSRLKGFSGKELDEEIETLIEKLELQEKRDYQSAGLSGGQKRRLGVGVALCGAAKVVLLD

10.19 ------------------------------------------------------------

10.1 --------------------------------------------------------IFVD

10.5 ------------------------------------------------------------

10.11 ------------------------------------------------------------

10.21 --------------------------------------------------------IFVD

10.24 ------------------------------------------------------------

10.22 --------------------------------------------------------IFVD

5B.6 ------------------------------------------------------------

5B.4 ------------------------------------------------------------

3.3 ------------------------------------------------------------

3.15 ------------------------------------------------------------

5A.12 FSRLKGFSGKELDEEIETLIEKLELQEKRDYQSAGLSGGQKRRLGVGVALCGAAKVVLLD

18.20 FSRLKGFSGKELDEEIETLIEKLELQEKRDYQSAGLSGGQKRRLGVGVALCGAAKVVLLD

3.2 ------------------------------------------------------------

5B.8 ------------------------------------------------------------

3.1 ------------------------------------------------------------

9B.16 ------------------------------------------------------------

9B.14 ------------------------------------------------------------

9B.12 ------------------------------------------------------------

9A.13 ------------------------------------------------------------

9A.10 ------------------------------------------------------------

9A.9 ------------------------------------------------------------

9A.8 ------------------------------------------------------------

APHIS-S EPTSGMDPASRRALWD-LLQREKKGRSMILTTHFMDEADILGDRVAIMADGRLQCVGSPY

5A.7 EPTSGMDPASRRALWD-LLQREKKGRSMILTTHFMDEADILGDRVAIMADGRLQCVGSPY

5A.11 EPTSGMDPASRRALWD-LLQREKKGRSMILTTHFMDEADILGDRVAIMADGRLQCVGSPY

10.29 EPTSGMDPASRRALWD-LLQREKKGRSMILTTHFMDEADILGDRVAIMADGRLQCVGSPY

18.7 EPTSGMDPASRRALWD-LLQREKKGRSMILTTHFMDEADILGDRVAIMADGRLQCVGSPY

18.6 EPTSGMDPASRRALWD-LLQREKKGRSMILTTHFMDEADILGDRVAIMADGRLQCVGSPY

10.19 ------------------------------------------------------------

10.1 EPTTGVDPAAKRQMWNAVREARRSGRGVVLTSHSMEECEALCSRLTIMVNGQFQCLGTPQ

10.5 -------PVQRRCLVG--------------------------------------------

10.11 -------PVQRRCLVG--------------------------------------------

10.21 EPTTGVDPAAKRQMWNAVREARRSGRGVVLTSHSMEECEALCSRLTIMVNGQFQCLGTPQ

10.24 ------------------------------------------------------------

10.22 EPTTGVDPAAKRQMWNAVREARRSGRGVVLTSHSMEECEALCSRLTIMVNGQFQCLGTPQ

5B.6 ------------------------------------------------------------

5B.4 ------------------------------------------------------------

3.3 ------------------------------------------------------------

3.15 -----------------------------------------------------RCSGTVC

5A.12 EPTSGMDPASRRALWD-LLQREKKGRSMILTTHFMDEADILGDRVAIMADGRLQCVGSPY

18.20 EPTSGMDPASRRALWD-LLQREKKGRSMILTTHFMDEADILGDRVAIMADGRLQCVGSPY

3.2 ------------------------------------------------------------

5B.8 ------------------------------------------------------------

3.1 ------------------------------------------------------------

9B.16 ------------------------------------------------------------

9B.14 ------------------------------------------------------------

9B.12 ------------------------------------------------------------

9A.13 ------------------------------------------------------------

9A.10 ------------------------------------------------------------

9A.9 ------------------------------------------------------------

9A.8 ------------------------------------------------------------

APHIS-S FLKRHYGVGYTLVVVKKEDFRLDTCTELINRYIPGTVVKEDRGTEVTYSMTNEYSHVFES

5A.7 FLKRHYGVGYTLVVVKKEDFRLDTCTELINRYIPGTVVKEDRGTEVTYSMTNEYSHVFES

5A.11 FLKRHYGVGYTLVVVKKEDFRLDTCTELINRYIPGTVVKEDRGTEVTYSMTNEYSHVFES

10.29 FLKRHYGVGYTLVVVKKEDFRLDTCTELINRYIPGTVVKEDRGTEVTYSMTNEYSHVFES

18.7 FLKRHYGVGYTLVVVKKEDFRLDTCTELINRYIPGTVVKEDRGTEVTYSMTNEYSHVFES

18.6 FLKRHYGVGYTLVVVKKEDFRLDTCTELINRYIPGTVVKEDRGTEVTYSMTNEYSHVFES

10.19 ------------------------------------------------------------

10.1 HLKNKFSEGFTLTI----------------------------------------------

10.5 ------------------------------------------------------------

10.11 ------------------------------------------------------------

10.21 HLKNKFSEGFTLTI----------------------------------------------

10.24 --------GFTLTI----------------------------------------------

10.22 HLKNKFSEGFTLTI----------------------------------------------

5B.6 ------------------------------------------------------------

5B.4 ------------------------------------------------------------

3.3 ------------------------------------------------------------

3.15 ------------------------------------------------------------

5A.12 FLKRHYGVGYTLVVVKKEDFRLDTCTELINRYIPGTVVKEDRGTEVTYSMTNEYSHVFES

18.20 FLKRHYGVGYTLVVVKKEDFRLDTCTELINRYIPGTVVKEDRGTEVTYSMTNEYSHVFES

3.2 ------------------------------------------------------------

5B.8 ------------------------------------------------------------

3.1 ------------------------------------------------------------

9B.16 ------------------------------------------------------------

9B.14 ------------------------------------------------------------

9B.12 ------------------------------------------------------------

9A.13 ------------------------------------------------------------

9A.10 ------------------------------------------------------------

9A.9 ------------------------------------------------------------

9A.8 ------------------------------------------------------------

APHIS-S MLRDLEAKADEINFKNYGLLATTLEDVFMSVGTDVVATSDVDDNTTVSSSADTLAFEYDS

5A.7 MLRDLEAKADEINFKNYGLLATTLEDVFMSVGTDVVATSDVDDNTTVSSSADTLAFEYDS

5A.11 MLRDLEAKADEINFKNYGLLATTLEDVFMSVGTDVVATSDVDDNTTVSSSADTLAFEYDS

10.29 MLRDLEAKADEINFKNYGLLATTLEDVFMSVGTDVVATSDVDDNTTVSSSADTLAFEYDS

18.7 MLRDLEAKADEINFKNYGLLATTLEDVFMSVGTDVVATSDVDDNTTVSSSADTLAFEYDS

18.6 MLRDLEAKADEINFKNYGLLATTLEDVFMSVGTDVVATSDVDDNTTVSSSADTLAFEYDS

10.19 ------------------------------------------------------------

10.1 ------------------------------------------------------------

10.5 ------------------------------------------------------------

10.11 ------------------------------------------------------------

10.21 ------------------------------------------------------------

10.24 ------------------------------------------------------------

10.22 ------------------------------------------------------------

5B.6 ------------------------------------------------------------

5B.4 ------------------------------------------------------------

3.3 ------------------------------------------------------------

3.15 ------------------------------------------------------------

5A.12 MLRDLEAKADEINFKNYGLLATTLEDVFMSVGTDVVATSDVDDNTTVSSSADTLAFEYDS

18.20 MLRDLEAKADEINFKNYGLLATTLEDVFMSVGTDVVATSDVDDNTTVSSSADTLAFEYDS

3.2 ------------------------------------------------------------

5B.8 ------------------------------------------------------------

3.1 ------------------------------------------------------------

9B.16 ------------------------------------------------------------

9B.14 ------------------------------------------------------------

9B.12 ------------------------------------------------------------

9A.13 ------------------------------------------------------------

9A.10 ------------------------------------------------------------

9A.9 ------------------------------------------------------------

9A.8 ------------------------------------------------------------

APHIS-S LEKLDGTGYGDEKGIRLICQHVVAIWMKLFLVLTRSWLILLLQVLVSLVQIIATLGVMQY

5A.7 LEKLDGTGYGDEKGIRLICQHVVAIWMKLFLVLTRSWLILLLQVLVSLVQIIATLGVMQY

5A.11 LEKLDGTGYGDEKGIRLICQHVVAIWMKLFLVLTRSWLILLLQVLVSLVQIIATLGVMQY

10.29 LEKLDGTGYGDEKGIRLICQHVVAIWMKLFLVLTRSWLILLLQVLVSLVQIIATLGVMQY

18.7 LEKLDGTGYGDEKGIRLICQHVVAIWMKLFLVLTRSWLILLLQVLVSLVQIIATLGVMQY

18.6 LEKLDGTGYGDEKGIRLICQHVVAIWMKLFLVLTRSWLILLLQVLVSLVQIIATLGVMQY

10.19 ------------------------------------------------------------

10.1 ------------------------------------------------------------

10.5 ------------------------------------------------------------

10.11 ------------------------------------------------------------

10.21 ------------------------------------------------------------

10.24 ------------------------------------------------------------

10.22 ------------------------------------------------------------

5B.6 ------------------------------------------------------------

5B.4 ------------------------------------------------------------

3.3 ------------------------------------------------------------

3.15 ------------------------------------------------------------

5A.12 LEKLDGTGYGDEKGIRLICQHVVAIWMKLFLVLTRSWLILLLQVLVSLVQIIATLGVMQY

18.20 LEKLDGTGYGDEKGIRLICQHVVAIWMKLFLVLTRSWLILLLQVLVSLVQIIATLGVMQY

3.2 ------------------------------------------------------------

5B.8 ------------------------------------------------------------

3.1 ------------------------------------------------------------

9B.16 ------------------------------------------------------------

9B.14 ------------------------------------------------------------

9B.12 ------------------------------------------------------------

9A.13 ------------------------------------------------------------

9A.10 ------------------------------------------------------------

9A.9 ------------------------------------------------------------

9A.8 ------------------------------------------------------------

APHIS-S VISMTEHIQRRELSLAEGFAGTETLVSFKGLSPTSTGSLAKAAYESIFVTANNPTMEITV

5A.7 VISMTEHIQRRELSLAEGFAGTETLVSFKGLSPTSTGSLAKAAYESIFVTANNPTMEITV

5A.11 VISMTEHIQRRELSLAEGFAGTETLVSFKGLSPTSTGSLAKAAYESIFVTANNPTMEITV

10.29 VISMTEHIQRRELSLAEGFAGTETLVSFKGLSPTSTGSLAKAAYESIFVTANNPTMEITV

18.7 VISMTEHIQRRELSLAEGFAGTETLVSFKGLSPTSTGSLAKAAYESIFVTANNPTMEITV

18.6 VISMTEHIQRRELSLAEGFAGTETLVSFKGLSPTSTGSLAKAAYESIFVTANNPTMEITV

10.19 ------------------------------------------------------------

10.1 ------------------------------------------------------------

10.5 ------------------------------------------------------------

10.11 ------------------------------------------------------------

10.21 ------------------------------------------------------------

10.24 ------------------------------------------------------------

10.22 ------------------------------------------------------------

5B.6 ------------------------------------------------------------

5B.4 ------------------------------------------------------------

3.3 ------------------------------------------------------------

3.15 ------------------------------------------------------------

5A.12 VISMTEHIQRRELSLAEGFAGTETLVSFKGLSPTSTGSLAKAAYESIFVTANNPTMEITV

18.20 VISMTEHIQRRELSLAEGFAGTETLVSFKGLSPTSTGSLAKAAYESIFVTANNPTMEITV

3.2 ------------------------------------------------------------

5B.8 ------------------------------------------------------------

3.1 ------------------------------------------------------------

9B.16 ------------------------------------------------------------

9B.14 ------------------------------------------------------------

9B.12 ------------------------------------------------------------

9A.13 ------------------------------------------------------------

9A.10 ------------------------------------------------------------

9A.9 ------------------------------------------------------------

9A.8 ------------------------------------------------------------

APHIS-S VDNTPIDEYYLERTDDVSAMAVLRHSLLIGATFDDHSATAWFSNFGYHDVAMSLAAVHAA

5A.7 VDNTPIDEYYLERTDDVSAMAVLRHSLLIGATFDDHSATAWFSNFGYHDVAMSLAAVHAA

5A.11 VDNTPIDEYYLERM----------------------TYQRWRCS----------------

10.29 VDNTPIDEYYLERTDDVSAMAVLRHSLLIGATFDDHSATAWFSNFGYHDVAMSLAAVHAA

18.7 VDNTPIDEYYLERTDDVSAMAVLRHSLLIGATFDDHSATAWFSNFGYHDVAMSLAAVHAA

18.6 VDNTPIDEYYLERTDDVSAMAVLRHSLLIGATFDDHSATAWFSNFGYHDVAMSLAAVHAA

10.19 ------------------------------------------------------------

10.1 ------------------------------------------------------------

10.5 ------------------------------------------------------------

10.11 ------------------------------------------------------------

10.21 ------------------------------------------------------------

10.24 ------------------------------------------------------------

10.22 ------------------------------------------------------------

5B.6 ------------------------------------------------------------

5B.4 ------------------------------------------------------------

3.3 ------------------------------------------------------------

3.15 ------------------------------------------------------------

5A.12 VDNTPIDEYYLERTDDVSAMAVLRHSLLIGATFDDHSATAWFSNFGYHDVAMSLAAVHAA

18.20 VDNTPIDEYYLERTDDVSAMAVLRHSLLIGATFDDHSATAWFSNFGYHDVAMSLAAVHAA

3.2 ------------------------------------------------------------

5B.8 ------------------------------------------------------------

3.1 ------------------------------------------------------------

9B.16 ------------------------------------------------------------

9B.14 ------------------------------------------------------------

9B.12 ------------------------------------------------------------

9A.13 ------------------------------------------------------------

9A.10 ------------------------------------------------------------

9A.9 ------------------------------------------------------------

9A.8 ------------------------------------------------------------

APHIS-S LLRAVNPAANLTVYNHPLEANYVNQNDMQTMVAFLSMQLASGIGSSLSIVSAVFIMFYIK

5A.7 LLRAVNPAANLTVYNHPLEANYVNQVGCTCAS----------------------------

5A.11 --------------------------GTVC------------------------------

10.29 LLRAVNPAANLTVYNHPLEANYVNQVGCTCAS----------------------------

18.7 LLRAVNPAANLTVYNHPLEANYVNQNDMQTMVAFLSMQLASGIGSSLSIVSAVFIMFYIK

18.6 LLRAVNPAANLTVYNHPLEANYVNQNDMQTMVAFLSMQLASGIGSSLSIVSAVFIMFYIK

10.19 ------------------------------------------------------------

10.1 ------------------------------------------------------------

10.5 ------------------------------------------------------------

10.11 ------------------------------------------------------------

10.21 ------------------------------------------------------------

10.24 ------------------------------------------------------------

10.22 ------------------------------------------------------------

5B.6 ------------------------------------------------------------

5B.4 ------------------------------------------------------------

3.3 ------------------------------------------------------------

3.15 ------------------------------------------------------------

5A.12 LLRAVNPAANLTVYNHPLEANYVNQNDMQTMVAFLSMQLASGIGSSLSIVSAVFIMFYIK

18.20 LLRAVNPAANLTVYNHPLEANYVNQNDMQTMVAFLSMQLASGIGSSLSIVSAVFIMFYIK

3.2 ------------------------------------------------------------

5B.8 ------------------------------------------------------------

3.1 ------------------------------------------------------------

9B.16 ------------------------------------------------------------

9B.14 ------------------------------------------------------------

9B.12 ------------------------------------------------------------

9A.13 ------------------------------------------------------------

9A.10 ------------------------------------------------------------

9A.9 ------------------------------------------------------------

9A.8 ------------------------------------------------------------

APHIS-S ERVSRAKLLQKAAGIQPLVMWLSAAVFDWIWFCVIAVGIVIACAAFNVIGLSSVDELGRM

5A.7 ------------------------------------------------------------

5A.11 ------------------------------------------------------------

10.29 ------------------------------------------------------------

18.7 ERVSRAKLLQKAAGIQPLVMWLSAAVFDWIWFCVIAVGIVIACAAFARRVYYGHGVKRYR

18.6 ERVSRAKLLQKAAGIQPLVMWLSAAVFDWIWFCVIAVGIVIACAAFARRVYYGHGVKRYR

10.19 ------------------------------------------------------------

10.1 ------------------------------------------------------------

10.5 ------------------------------------------------------------

10.11 ------------------------------------------------------------

10.21 ------------------------------------------------------------

10.24 ------------------------------------------------------------

10.22 ------------------------------------------------------------

5B.6 ------------------------------------------------------------

5B.4 ------------------------------------------------------------

3.3 ------------------------------------------------------------

3.15 ------------------------------------------------------------

5A.12 VGCTCAS-----------------------------------------------------

18.20 ERVSRAKLLQKAAGIQPLVMWLSAAVFDWIWFCVIAVGIVIACAAFARRVYYGHGVKRYR

3.2 ------------------------------------------------------------

5B.8 ------------------------------------------------------------

3.1 ------------------------------------------------------------

9B.16 ------------------------------------------------------------

9B.14 ------------------------------------------------------------

9B.12 ------------------------------------------------------------

9A.13 ------------------------------------------------------------

9A.10 ------------------------------------------------------------

9A.9 ------------------------------------------------------------

9A.8 ------------------------------------------------------------

APHIS-S YLCIIVYGAASLPIGYVFSYFFKGPAVGFVTMFFINILFGMMGAQIVEALLSPQLDTENV

5A.7 ------------------------------------------------------------

5A.11 ------------------------------------------------------------

10.29 ------------------------------------------------------------

18.7 AEYLYCVFQCSTSLYSVKYA----------------------------------------

18.6 AEYLYCVFQCSTSLYSVKYA----------------------------------------

10.19 ------------------------------------------------------------

10.1 ------------------------------------------------------------

10.5 ------------------------------------------------------------

10.11 ------------------------------------------------------------

10.21 ------------------------------------------------------------

10.24 ------------------------------------------------------------

10.22 ------------------------------------------------------------

5B.6 ------------------------------------------------------------

5B.4 ------------------------------------------------------------

3.3 ------------------------------------------------------------

3.15 ------------------------------------------------------------

5A.12 ------------------------------------------------------------

18.20 AEYLYCVFQCSTSLYSVKYA----------------------------------------

3.2 ------------------------------------------------------------

5B.8 ------------------------------------------------------------

3.1 ------------------------------------------------------------

9B.16 ------------------------------------------------------------

9B.14 ------------------------------------------------------------

9B.12 ------------------------------------------------------------

9A.13 ------------------------------------------------------------

9A.10 ------------------------------------------------------------

9A.9 ------------------------------------------------------------

9A.8 ------------------------------------------------------------

APHIS-S ANILDSILQFFPLYSLVTSARLLNQVGLLEWSCLQNCEYLSAVMPNLTECSMDVMCQTFS

5A.7 ------------------------------------------------------------

5A.11 ------------------------------------------------------------

10.29 ------------------------------------------------------------

18.7 ------------------------------------------------------------

18.6 ------------------------------------------------------------

10.19 ------------------------------------------------------------

10.1 ------------------------------------------------------------

10.5 ------------------------------------------------------------

10.11 ------------------------------------------------------------

10.21 ------------------------------------------------------------

10.24 ------------------------------------------------------------

10.22 ------------------------------------------------------------

5B.6 ------------------------------------------------------------

5B.4 ------------------------------------------------------------

3.3 ------------------------------------------------------------

3.15 ------------------------------------------------------------

5A.12 ------------------------------------------------------------

18.20 ------------------------------------------------------------

3.2 ------------------------------------------------------------

5B.8 ------------------------------------------------------------

3.1 ------------------------------------------------------------

9B.16 ------------------------------------------------------------

9B.14 ------------------------------------------------------------

9B.12 ------------------------------------------------------------

9A.13 ------------------------------------------------------------

9A.10 ------------------------------------------------------------

9A.9 ------------------------------------------------------------

9A.8 ------------------------------------------------------------

APHIS-S QCCIPDDPWFMWDHPGVLRYIVCMIVSGVVMWFVLLIAEYRLFQKVIYREKKAPPVDESA

5A.7 ------------------------------------------------------------

5A.11 ------------------------------------------------------------

10.29 ------------------------------------------------------------

18.7 ------------------------------------------------------------

18.6 ------------------------------------------------------------

10.19 ------------------------------------------------------------

10.1 ------------------------------------------------------------

10.5 ------------------------------------------------------------

10.11 ------------------------------------------------------------

10.21 ------------------------------------------------------------

10.24 ------------------------------------------------------------

10.22 ------------------------------------------------------------

5B.6 ------------------------------------------------------------

5B.4 ------------------------------------------------------------

3.3 ------------------------------------------------------------

3.15 ------------------------------------------------------------

5A.12 ------------------------------------------------------------

18.20 ------------------------------------------------------------

3.2 ------------------------------------------------------------

5B.8 ------------------------------------------------------------

3.1 ------------------------------------------------------------

9B.16 ------------------------------------------------------------

9B.14 ------------------------------------------------------------

9B.12 ------------------------------------------------------------

9A.13 ------------------------------------------------------------

9A.10 ------------------------------------------------------------

9A.9 ------------------------------------------------------------

9A.8 ------------------------------------------------------------

APHIS-S LDNDVADEARHVARVGAGAILGQHSLVANGLTKYYGKHLAVNQVSFTVGDTECFGLLGVN

5A.7 ------------------------------------------------------------

5A.11 ------------------------------------------------------------

10.29 ------------------------------------------------------------

18.7 ------------------------------------------------------------

18.6 ------------------------------------------------------------

10.19 ------------------------------------------------------------

10.1 ------------------------------------------------------------

10.5 ------------------------------------------------------------

10.11 ------------------------------------------------------------

10.21 ------------------------------------------------------------

10.24 ------------------------------------------------------------

10.22 ------------------------------------------------------------

5B.6 ------------------------------------------------------------

5B.4 ------------------------------------------------------------

3.3 ------------------------------------------------------------

3.15 ------------------------------------------------------------

5A.12 ------------------------------------------------------------

18.20 ------------------------------------------------------------

3.2 ------------------------------------------------------------

5B.8 ------------------------------------------------------------

3.1 ------------------------------------------------------------

9B.16 ------------------------------------------------------------

9B.14 ------------------------------------------------------------

9B.12 ------------------------------------------------------------

9A.13 ------------------------------------------------------------

9A.10 ------------------------------------------------------------

9A.9 ------------------------------------------------------------

9A.8 ------------------------------------------------------------

APHIS-S GAGKTTTFKMLMGDETVSSGDAFVSGHSVKTNITQVYKNIGYCPQFEATFGELTGRETLR

5A.7 ------------------------------------------------------------

5A.11 ------------------------------------------------------------

10.29 ------------------------------------------------------------

18.7 ------------------------------------------------------------

18.6 ------------------------------------------------------------

10.19 ------------------------------------------------------------

10.1 ------------------------------------------------------------

10.5 ------------------------------------------------------------

10.11 ------------------------------------------------------------

10.21 ------------------------------------------------------------

10.24 ------------------------------------------------------------

10.22 ------------------------------------------------------------

5B.6 ------------------------------------------------------------

5B.4 ------------------------------------------------------------

3.3 ------------------------------------------------------------

3.15 ------------------------------------------------------------

5A.12 ------------------------------------------------------------

18.20 ------------------------------------------------------------

3.2 ------------------------------------------------------------

5B.8 ------------------------------------------------------------

3.1 ------------------------------------------------------------

9B.16 ------------------------------------------------------------

9B.14 ------------------------------------------------------------

9B.12 ------------------------------------------------------------

9A.13 ------------------------------------------------------------

9A.10 ------------------------------------------------------------

9A.9 ------------------------------------------------------------

9A.8 ------------------------------------------------------------

APHIS-S LFSALRGLPVRGATLHAEALAHALGFYKHLDKRVDHYSGGNKRKLSTAVALLGRTRLIFV

5A.7 ------------------------------------------------------------

5A.11 ------------------------------------------------------------

10.29 ------------------------------------------------------------

18.7 ------------------------------------------------------------

18.6 ------------------------------------------------------------

10.19 ------------------------------------------------------------

10.1 ------------------------------------------------------------

10.5 ------------------------------------------------------------

10.11 ------------------------------------------------------------

10.21 ------------------------------------------------------------

10.24 ------------------------------------------------------------

10.22 ------------------------------------------------------------

5B.6 ------------------------------------------------------------

5B.4 ------------------------------------------------------------

3.3 ------------------------------------------------------------

3.15 ------------------------------------------------------------

5A.12 ------------------------------------------------------------

18.20 ------------------------------------------------------------

3.2 ------------------------------------------------------------

5B.8 ------------------------------------------------------------

3.1 ------------------------------------------------------------

9B.16 ------------------------------------------------------------

9B.14 ------------------------------------------------------------

9B.12 ------------------------------------------------------------

9A.13 ------------------------------------------------------------

9A.10 ------------------------------------------------------------

9A.9 ------------------------------------------------------------

9A.8 ------------------------------------------------------------

APHIS-S DEPTTGVDPAAKRQMWNAVREARRSGRGVVLTSHSMEECEALCSRLTIMVNGQFQCLGTP

5A.7 ------------------------------------------------------------

5A.11 ------------------------------------------------------------

10.29 ------------------------------------------------------------

18.7 ------------------------------------------------------------

18.6 ------------------------------------------------------------

10.19 ------------------------------------------------------------

10.1 ------------------------------------------------------------

10.5 ------------------------------------------------------------

10.11 ------------------------------------------------------------

10.21 ------------------------------------------------------------

10.24 ------------------------------------------------------------

10.22 ------------------------------------------------------------

5B.6 ------------------------------------------------------------

5B.4 ------------------------------------------------------------

3.3 ------------------------------------------------------------

3.15 ------------------------------------------------------------

5A.12 ------------------------------------------------------------

18.20 ------------------------------------------------------------

3.2 ------------------------------------------------------------

5B.8 ------------------------------------------------------------

3.1 ------------------------------------------------------------

9B.16 ------------------------------------------------------------

9B.14 ------------------------------------------------------------

9B.12 ------------------------------------------------------------

9A.13 ------------------------------------------------------------

9A.10 ------------------------------------------------------------

9A.9 ------------------------------------------------------------

9A.8 ------------------------------------------------------------

APHIS-S QHLKNKFSEGFTLTIKIKVDDETKTVRPEVCDAVKHYVSTNFREPKIMEEYQGLLTYYLP

5A.7 ------------------------------------------------------------

5A.11 ------------------------------------------------------------

10.29 ------------------------------------------------------------

18.7 ------------------------------------------------------------

18.6 ------------------------------------------------------------

10.19 ------------------------------------------------------------

10.1 ---------------KIKVDDETKTVRPEVCDAVKHYVSTNFREPKIMEEYQGLLTYYLP

10.5 ------------------------------------------------------------

10.11 ------------------------------------------------------------

10.21 ---------------KIKVDDETKTVRPEVCDAVKHYVSTNFREPKIMEEYQGLLTYYLP

10.24 ---------------KIKVDDETKTVRPEVCDAVKHYVSTNFREPKIMEEYQGLLTYYLP

10.22 ---------------KIKVDDETKTVRPEVCDAVKHYVSTNFREPKIMEEYQGLLTYYLP

5B.6 ------------------------------------------------------------

5B.4 ------------------------------------------------------------

3.3 ------------------------------------------------------------

3.15 ------------------------------------------------------------

5A.12 ------------------------------------------------------------

18.20 ------------------------------------------------------------

3.2 ------------------------------------------------------------

5B.8 -------------------------------------------------

3.1 -------------------------------------------------

9B.16 -------------------------------------------------

9B.14 -------------------------------------------------

9B.12 -------------------------------------------------

9A.13 -------------------------------------------------

9A.10 -------------------------------------------------

9A.9 -------------------------------------------------

9A.8 -------------------------------------------------

APHIS-S DKSVAWSRMFGIMEAAKRDLPVEDYSISQTTLEQIFLQFTKYQHEAQQT

5A.7 -------------------------------------------------

5A.11 -------------------------------------------------

10.29 -------------------------------------------------

18.7 -------------------------------------------------

18.6 -------------------------------------------------

10.19 -------------------------------------------------

10.1 DKSVAWSRMFGIMEAAKRDLPVEDYSISQTTLE----------------

10.5 -------------------------------------------------

10.11 -------------------------------------------------

10.21 DKSVAWSRMFGIMEAAKRDLPVEDYSISQTTLE----------------

10.24 DKSVAWSRMFGIMEAAKRDLPVEDYSISQTTLE----------------

10.22 DKSVAWSRMFGIMEAAKRDLPVEDYSISQTTLE----------------

5B.6 -------------------------------------------------

5B.4 -------------------------------------------------

3.3 -------------------------------------------------

3.15 -------------------------------------------------

5A.12 -------------------------------------------------

18.20 -------------------------------------------------

3.2 -------------------------------------------------

**Supplementary Figure S6. Alignment of *PgABCA2* cDNA sequences from F_2_ survivors on 3 μg Cry2Ab per mL obtained from CRISPR-R2 X APHIS-S single-pair families.** Twelve *PgABCA2* cDNA clones from five individuals from three CRISPR-R2 X APHIS-S single-pair cross families are aligned with the full-length *PgABCA2* (MG637361.1) from susceptible APHIS-S strain (the first letter represents the family A, C, or J, number to the right of the first decimal point refers to the individual and the number to the right of the second decimal point refers to clone). sgRNA target sites 1-5 are highlighted in magenta, cyan, teal, yellow, and red, respectively. Predicted exon/intron splice sites are shown in gray. The order of sequences is based on their similarity determined by MUSCLE in Clustal. Premature stop codons are highlighted in black with white text. Stars show nucleotide bases conserved in all of the sequences.

J.5.2 ATGCGGGCGCGTGGAGAGCGGAAGGAGGCGGGCTCATGGGTGAAGTTTAGGCTGTTGATG

J.5.6 ATGCGGGCGCGTGGAGAGCGGAAGGAGGCGGGCTCATGGGTGAAGTTTAGGCTGTTGATG

J.5.3 ATGCGGGCGCGTGGAGAGCGGAAGGAGGCGGGCTCATGGGTGAAGTTTAGGCTGTTGATG

J.6.4 ATGCGGGCGCGTGGAGAGCGGAAGGAGGCGGGCTCATGGGTGAAGTTTAGGCTGTTGATG

C.3.6 ATGCGGGCGCGTGGAGAGCGGAAGGAGGCGGGCTCATGGGTGAAGTTTAGGCTGTTGATG

C.4.6 ATGCGGGCGCGTGGAGAGCGGAAGGAGGCGGGCTCATGGGTGAAGTTTAGGCTGTTGATG

C.4.8 ATGCGGGCGCGTGGAGAGCGGAAGGAGGCGGGCTCATGGGTGAAGTTTAGGCTGTTGATG

C.4.10 ATGCGGGCGCGTGGAGAGCGGAAGGAGGCGGGCTCATGGGTGAAGTTTAGGCTGTTGATG

A.2.9 ATGCGGGCGCGTGGAGAGCGGAAGGAGGCGGGCTCATGGGTGAAGTTTAGGCTGTTGATG

A.1.1 ATGCGGGCGCGTGGAGAGCGGAAGGAGGCGGGCTCATGGGTGAAGTTTAGGCTGTTGATG

A.2.2 ATGCGGGCGCGTGGAGAGCGGAAGGAGGCGGGCTCATGGGTGAAGTTTAGGCTGTTGATG

APHIS-S ATGCGGGCGCGTGGAGAGCGGAAGGAGGCGGGCTCATGGGTGAAGTTTAGGCTGTTGATG

C.3.4 ATGCGGGCGCGTGGAGAGCGGAAGGAGGCGGGCTCATGGGTGAAGTTTAGGCTGTTGATG

************************************************************

J.5.2 TGGAAGAACTTCGTGCAGCAGTTGAGGCACCCAGTGCAGACGGCGGCTGAGCTGCTGCTA

J.5.6 TGGAAGAACTTCGTGCAGCAGTTGAGGCACCCAGTGCAGACGGCGGCTGAGCTGCTGCTA

J.5.3 TGGAAGAACTTCGTGCAGCAGTTGAGGCACCCAGTGCAGACGGCGGCTGAGCTGCTGCTA

J.6.4 TGGAAGAACTTCGTGCAGCAGTTGAGGCACCCAGTGCAGACGGCGGCTGAGCTGCTGCTA

C.3.6 TGGAAGAACTTCGTGCAGCAGTTGAGGCACCCAGTGCAGACGGCGGCTGAGCTGCTGCTA

C.4.6 TGGAAGAACTTCGTGCAGCAGTTGAGGCACCCAGTGCAGACGGCGGCTGAGCTGCTGCTA

C.4.8 TGGAAGAACTTCGTGCAGCAGTTGAGGCACCCAGTGCAGACGGCGGCTGAGCTGCTGCTA

C.4.10 TGGAAGAACTTCGTGCAGCAGTTGAGGCACCCAGTGCAGACGGCGGCTGAGCTGCTGCTA

A.2.9 TGGAAGAACTTCGTGCAGCAGTTGAGGCACCCAGTGCAGACGGCGGCTGAGCTGCTGCTA

A.1.1 TGGAAGAACTTCGTGCAGCAGTTGAGGCACCCAGTGCAGACGGCGGCTGAGCTGCTGCTA

A.2.2 TGGAAGAACTTCGTGCAGCAGTTGAGGCACCCAGTGCAGACGGCGGCTGAGCTGCTGCTA

APHIS-S TGGAAGAACTTCGTGCAGCAGTTGAGGCACCCAGTGCAGACGGCGGCTGAGCTGCTGCTA

C.3.4 TGGAAGAACTTCGTGCAGCAGTTGAGGCACCCAGTGCAGACGGCGGCTGAGCTGCTGCTA

************************************************************

J.5.2 CCAGTCCTAACCATGAGCCTGGTCCTGGTGCTACGGTCACAGATCGACCCCGAAGTCTTG

J.5.6 CCAGTCCTAACCATGAGCCTGGTCCTGGTGCTACGGTCACAGATCGACCCCGAAGTCTTG

J.5.3 CCAGTCCTAACCATGAGCCTGGTCCTGGTGCTACGGTCACAGATCGACCCCGAAGTCTTG

J.6.4 CCAGTCCTAACCATGAGCCTGGTCCTGGTGCTACGGTCACAGATCGACCCCGAAGTCTTG

C.3.6 CCAGTCCTAACCATGAGCCTGGTCCTGGTGCTACGGTCACAGATCGACCCCGAAGTCTTG

C.4.6 CCAGTCCTAACCATGAGCCTGGTCCTGGTGCTACGGTCACAGATCGACCCCGAAGTCTTG

C.4.8 CCAGTCCTAACCATGAGCCTGGTCCTGGTGCTACGGTCACAGATCGACCCCGAAGTCTTG

C.4.10 CCAGTCCTAACCATGAGCCTGGTCCTGGTGCTACGGTCACAGATCGACCCCGAAGTCTTG

A.2.9 CCAGTCCTAACCATGAGCCTGGTCCTGGTGCTACGGTCACAGATCGACCCCGAAGTCTTG

A.1.1 CCAGTCCTAACCATGAGCCTGGTCCTGGTGCTACGGTCACAGATCGACCCCGAAGTCTTG

A.2.2 CCAGTCCTAACCATGAGCCTGGTCCTGGTGCTACGGTCACAGATCGACCCCGAAGTCTTG

APHIS-S CCAGTCCTAACCATGAGCCTGGTCCTGGTGCTACGGTCACAGATCGACCCCGAAGTCTTG

C.3.4 CCAGTCCTAACCATGAGCCTGGTCCTGGTGCTACGGTCACAGATCGACCCCGAAGTCTTG

************************************************************

J.5.2 GAAACCAGAACCTACCCGCCAATACCAGCCCACACTTTAAACTATTCCGTGACTGTTTTG

J.5.6 GAAACCAGAACCTACCCGCCAATACCAGCCCACACTTTAAACTATTCCGTGACTGTTTTG

J.5.3 GAAACCAGAACCTACCCGCCAATACCAGCCCACACTTTAAACTATTCCGTGACTGTTTTG

J.6.4 GAAACCAGAACCTACCCGCCAATACCAGCCCACACTTTAAACTATTCCGTGACTGTTTTG

C.3.6 GAAACCAGAACCTACCCGCCAATACCAGCCCACACTTTAAACTATTCCGTGACTGTTTTG

C.4.6 GAAACCAGAACCTACCCGCCAATACCAGCCCACACTTTAAACTATTCCGTGACTGTTTTG

C.4.8 GAAACCAGAACCTACCCGCCAATACCAGCCCACACTTTAAACTATTCCGTGACTGTTTTG

C.4.10 GAAACCAGAACCTACCCGCCAATACCAGCCCACACTTTAAACTATTCCGTGACTGTTTTG

A.2.9 GAAACCAGAACCTACCCGCCAATACCAGCCCACACTTTAAACTATTCCGTGACTGTTTTG

A.1.1 GAAACCAGAACCTACCCGCCAATACCAGCCCACACTTTAAACTATTCCGTGACTGTTTTG

A.2.2 GAAACCAGAACCTACCCGCCAATACCAGCCCACACTTTAAACTATTCCGTGACTGTTTTG

APHIS-S GAAACCAGAACCTACCCGCCAATACCAGCCCACACTTTAAACTATTCCGTGACTGTTTTG

C.3.4 GAAACCAGAACCTACCCGCCAATACCAGCCCACACTTTAAACTATTCCGTGACTGTTTTG

************************************************************

J.5.2 GGCGGAATGAATTTAACAAGAATGTCCATGGCATTCTCACCC----------------GA

J.5.6 GGCGGAATGAATTTAACAAGAATGTCCATGGCATTCTCACCC----------------GA

J.5.3 GGCGGAATGAATTTAACAAGAATGTCCATGGCATTCTCACCC----------------GA

J.6.4 GGCGGAATGAATTTAACAAGAATGTCCATGGCATTCTCACCC----------------GA

C.3.6 GGCGGAATGAATTTAACAAGAATGTCCATGGCATTCTCACCCGAGAATGCCGT--ATTGA

C.4.6 GGCGGAATGAATTTAACAAGAATGTCCATGGCATTCTCACCCGAGAATGCCATGAGATGA

C.4.8 GGCGGAATGAATTTAACAAGAATGTCCATGGCATTCTCACCCGAGAATGCCATGAGATGA

C.4.10 GGCGGAATGAATTTAACAAGAATGTCCATGGCATTCTCACCCGAGAATGCCATGAGATGA

A.2.9 GGCGGAATGAATTTAACAAGAATGTCCATGGCATTCTCACCCGAGAATGCCGT--ATTGA

A.1.1 GGCGGAATGAATTTAACAAGAATGTCCATGGCATTCTCACCCGAGAATGCCGT--ATTGA

A.2.2 GGCGGAATGAATTTAACAAGAATGTCCATGGCATTCTCACCCGAGAATGCCGT--ATTGA

APHIS-S GGCGGAATGAATTTAACAAGAATGTCCATGGCATTCTCACCCGAGAATGCCGT--ATTGA

C.3.4 GGCGGAATGAATTTAACAAGAATGTCCATGGCATTCTCACCCGAGAATGCCGT--ATTGA

****************************************** **

J.5.2 GGGACGTCGTATCCAGTGCTACAACAAAGTTACTGCTTAAAAACATGAGAGACCAAGTAC

J.5.6 GGGACGTCGTATCCAGTGCTACAACAAAGTTACTGCTTAAAAACATGAGAGACCAAGTAC

J.5.3 GGGACGTCGTATCCAGTGCTACAACAAAGTTACTGCTTAAAAACATGAGAGACCAAGTAC

J.6.4 GGGACGTCGTATCCAGTGCTACAACAAAGTTACTGCTTAAAAACATGAGAGACCAAGTAC

C.3.6 GGGACGTCGTATCCAGTGCTACAACAAAGTTACTGCTTAAAAACATGAGAGACCAAGTAC

C.4.6 GGGACGTCGTATCCAGTGCTACAACAAAGTTACTGCTTAAAAACATGAGAGACCAAGTAC

C.4.8 GGGACGTCGTATCCAGTGCTACAACAAAGTTACTGCTTAAAAACATGAGAGACCAAGTAC

C.4.10 GGGACGTCGTATCCAGTGCTACAACAAAGTTACTGCTTAAAAACATGAGAGACCAAGTAC

A.2.9 GGGACGTCGTATCCAGTGCTACAACAAAGTTACTGCTTAAAAACATGAGAGACCAAGTAC

A.1.1 GGGACGTCGTATCCAGTGCTACAACAAAGTTACTGCTTAAAAACATGAGAGACCAAGTAC

A.2.2 GGGACGTCGTATCCAGTGCTACAACAAAGTTACTGCTTAAAAACATGAGAGACCAAGTAC

APHIS-S GGGACGTCGTATCCAGTGCTACAACAAAGTTACTGCTTAAAAACATGAGAGACCAAGTAC

C.3.4 GGGACGTCGTATCCAGTGCTACAACAAAGTTACTGCTTAAAAACATGAGAGACCAAGTAC

************************************************************

J.5.2 TGCCCATCATTGAGGCATTGCCAATAGAAATACCGCCGGGACTGGTAAACTCGTCACAGG

J.5.6 TGCCCATCATTGAGGCATTGCCAATAGAAATACCGCCGGGACTGGTAAACTCGTCACAGG

J.5.3 TGCCCATCATTGAGGCATTGCCAATAGAAATACCGCCGGGACTGGTAAACTCGTCACAGG

J.6.4 TGCCCATCATTGAGGCATTGCCAATAGAAATACCGCCGGGACTGGTAAACTCGTCACAGG

C.3.6 TGCCCATCATTGAGGCATTGCCAATAGAAATACCGCCGGGACTGGTAAACTCGTCACAGG

C.4.6 TGCCCATCATTGAGGCATTGCCAATAGAAATACCGCCGGGACTGGTAAACTCGTCACAGG

C.4.8 TGCCCATCATTGAGGCATTGCCAATAGAAATACCGCCGGGACTGGTAAACTCGTCACAGG

C.4.10 TGCCCATCATTGAGGCATTGCCAATAGAAATACCGCCGGGACTGGTAAACTCGTCACAGG

A.2.9 TGCCCATCATTGAGGCATTGCCAATAGAAATACCGCCGGGACTGGTAAACTCGTCACAGG

A.1.1 TGCCCATCATTGAGGCATTGCCAATAGAAATACCGCCGGGACTGGTAAACTCGTCACAGG

A.2.2 TGCCCATCATTGAGGCATTGCCAATAGAAATACCGCCGGGACTGGTAAACTCGTCACAGG

APHIS-S TGCCCATCATTGAGGCATTGCCAATAGAAATACCGCCGGGACTGGTAAACTCGTCACAGG

C.3.4 TGCCCATCATTGAGGCATTGCCAATAGAAATACCGCCGGGACTGGTAAACTCGTCACAGG

************************************************************

J.5.2 TGTACGAAATAGTTAAATTATTTGTCGACGAGAACGTTGTTACCGGATACAATAGCAGTG

J.5.6 TGTACGAAATAGTTAAATTATTTGTCGACGAGAACGTTGTTACCGGATACAATAGCAGTG

J.5.3 TGTACGAAATAGTTAAATTATTTGTCGACGAGAACGTTGTTACCGGATACAATAGCAGTG

J.6.4 TGTACGAAATAGTTAAATTATTTGTCGACGAGAACGTTGTTACCGGATACAATAGCAGTG

C.3.6 TGTACGAAATAGTTAAATTATTTGTCGACGAGAACGTTGTTACCGGATACAATAGCAGTG

C.4.6 TGTACGAAATAGTTAAATTATTTGTCGACGAGAACGTTGTTACCGGATACAATAGCAGTG

C.4.8 TGTACGAAATAGTTAAATTATTTGTCGACGAGAACGTTGTTACCGGATACAATAGCAGTG

C.4.10 TGTACGAAATAGTTAAATTATTTGTCGACGAGAACGTTGTTACCGGATACAATAGCAGTG

A.2.9 TGTACGAAATAGTTAAATTATTTGTCGACGAGAACGTTGTTACCGGATACAATAGCAGTG

A.1.1 TGTACGAAATAGTTAAATTATTTGTCGACGAGAACGTTGTTACCGGATACAATAGCAGTG

A.2.2 TGTACGAAATAGTTAAATTATTTGTCGACGAGAACGTTGTTACCGGATACAATAGCAGTG

APHIS-S TGTACGAAATAGTTAAATTATTTGTCGACGAGAACGTTGTTACCGGATACAATAGCAGTG

C.3.4 TGTACGAAATAGTTAAATTATTTGTCGACGAGAACGTTGTTACCGGATACAATAGCAGTG

************************************************************

J.5.2 CGGCAATGAGAGGAATATACGCAGAGGAAGAAGCCACGAGAAGGGTGATAGCTGGCATAG

J.5.6 CGGCAATGAGAGGAATATACGCAGAGGAAGAAGCCACGAGAAGGGTGATAGCTGGCATAG

J.5.3 CGGCAATGAGAGGAATATACGCAGAGGAAGAAGCCACGAGAAGGGTGATAGCTGGCATAG

J.6.4 CGGCAATGAGAGGAATATACGCAGAGGAAGAAGCCACGAGAAGGGTGATAGCTGGCATAG

C.3.6 CGGCAATGAGAGGAATATACGCAGAGGAAGAAGCCACGAGAAGGGTGATAGCTGGCATAG

C.4.6 CGGCAATGAGAGGAATATACGCAGAGGAAGAAGCCACGAGAAGGGTGATAGCTGGCATAG

C.4.8 CGGCAATGAGAGGAATATACGCAGAGGAAGAAGCCACGAGAAGGGTGATAGCTGGCATAG

C.4.10 CGGCAATGAGAGGAATATACGCAGAGGAAGAAGCCACGAGAAGGGTGATAGCTGGCATAG

A.2.9 CGGCAATGAGAGGAATATACGCAGAGGAAGAAGCCACGAGAAGGGTGATAGCTGGCATAG

A.1.1 CGGCAATGAGAGGAATATACGCAGAGGAAGAAGCCACGAGAAGGGTGATAGCTGGCATAG

A.2.2 CGGCAATGAGAGGAATATACGCAGAGGAAGAAGCCACGAGAAGGGTGATAGCTGGCATAG

APHIS-S CGGCAATGAGAGGAATATACGCAGAGGAAGAAGCCACGAGAAGGGTGATAGCTGGCATAG

C.3.4 CGGCAATGAGAGGAATATACGCAGAGGAAGAAGCCACGAGAAGGGTGATAGCTGGCATAG

************************************************************

J.5.2 AATTCGATGACTCATTGCGTGAAATAACGGAGCTACCACTAGACTTGTCGTATGCGCTTC

J.5.6 AATTCGATGACTCATTGCGTGAAATAACGGAGCTACCACTAGACTTGTCGTATGCGCTTC

J.5.3 AATTCGATGACTCATTGCGTGAAATAACGGAGCTACCACTAGACTTGTCGTATGCGCTTC

J.6.4 AATTCGATGACTCATTGCGTGAAATAACGGAGCTACCACTAGACTTGTCGTATGCGCTTC

C.3.6 AATTCGATGACTCATTGCGTGAAATAACGGAGCTACCACTAGACTTGTCGTATGCGCTTC

C.4.6 AATTCGATGACTCATTGCGTGAAATAACGGAGCTACCACTAGACTTGTCGTATGCGCTTC

C.4.8 AATTCGATGACTCATTGCGTGAAATAACGGAGCTACCACTAGACTTGTCGTATGCGCTTC

C.4.10 AATTCGATGACTCATTGCGTGAAATAACGGAGCTACCACTAGACTTGTCGTATGCGCTTC

A.2.9 AATTCGATGACTCATTGCGTGAAATAACGGAGCTACCACTAGACTTGTCGTATGCGCTTC

A.1.1 AATTCGATGACTCATTGCGTGAAATAACGGAGCTACCACTAGACTTGTCGTATGCGCTTC

A.2.2 AATTCGATGACTCATTGCGTGAAATAACGGAGCTACCACTAGACTTGTCGTATGCGCTTC

APHIS-S AATTCGATGACTCATTGCGTGAAATAACGGAGCTACCACTAGACTTGTCGTATGCGCTTC

C.3.4 AATTCGATGACTCATTGCGTGAAATAACGGAGCTACCACTAGACTTGTCGTATGCGCTTC

************************************************************

J.5.2 GTTTTCCGGAGAGACCTCGCTTGAATTCCTTCTTCATGACAGGCGGTCGGACTTGGCGCA

J.5.6 GTTTTCCGGAGAGACCTCGCTTGAATTCCTTCTTCATGACAGGCGGTCGGACTTGGCGCA

J.5.3 GTTTTCCGGAGAGACCTCGCTTGAATTCCTTCTTCATGACAGGCGGTCGGACTTGGCGCA

J.6.4 GTTTTCCGGAGAGACCTCGCTTGAATTCCTTCTTCATGACAGGCGGTCGGACTTGGCGCA

C.3.6 GTTTTCCGGAGAGACCTCGCTTGAATTCCTTCTTCATGACAGGCGGTCGGACTTGGCGCA

C.4.6 GTTTTCCGGAGAGACCTCGCTTGAATTCCTTCTTCATGACAGGCGGTCGGACTTGGCGCA

C.4.8 GTTTTCCGGAGAGACCTCGCTTGAATTCCTTCTTCATGACAGGCGGTCGGACTTGGCGCA

C.4.10 GTTTTCCGGAGAGACCTCGCTTGAATTCCTTCTTCATGACAGGCGGTCGGACTTGGCGCA

A.2.9 GTTTTCCGGAGAGACCTCGCTTGAATTCCTTCTTCATGACAGGCGGTCGGACTTGGCGCA

A.1.1 GTTTTCCGGAGAGACCTCGCTTGAATTCCTTCTTCATGACAGGCGGTCGGACTTGGCGCA

A.2.2 GTTTTCCGGAGAGACCTCGCTTGAATTCCTTCTTCATGACAGGCGGTCGGACTTGGCGCA

APHIS-S GTTTTCCGGAGAGACCTCGCTTGAATTCCTTCTTCATGACAGGCGGTCGGACTTGGCGCA

C.3.4 GTTTTCCGGAGAGACCTCGCTTGAATTCCTTCTTCATGACAGGCGGTCGGACTTGGCGCA

************************************************************

J.5.2 CAGATAACGTGTTTCCTATG-----------------------------------GGAA-

J.5.6 CAGATAACGTGTTTCCTATG-----------------------------------GGAA-

J.5.3 CAGATAACGTGTTTCCTATG-----------------------------------GGAA-

J.6.4 CAGATAACGTGTTTCCTATG-----------------------------------GGAA-

C.3.6 CAGATAACGTGTTTCCTATGTTCGAAGTTCCCGGACCTCGCTTTCCGTATTCATGGGAAG

C.4.6 CAGATAACGTGTTTC--------GAAGTTCCCGGACCTCGCTTTCCGTATTCATGGGAAG

C.4.8 CAGATAACGTGTTTC--------GAAGTTCCCGGACCTCGCTTTCCGTATTCATGGGAAG

C.4.10 CAGATAACGTGTTTC--------GAAGTTCCCGGACCTCGCTTTCCGTATTCATGGGAAG

A.2.9 CAGATAACGTGTTTCCTATGTTCGAAGTTCCCGGACCTCGCTTTCCGTATTCATGGGAAG

A.1.1 CAGATAACGTGTTTCCTATGTTCGAAGTTCCCGGACCTCGCTTTCCGTATTCATGGGAAG

A.2.2 CAGATAACGTGTTTCCTATGTTCGAAGTTCCCGGACCTCGCTTTCCGTATTCATGGGAAG

APHIS-S CAGATAACGTGTTTCCTATGTTCGAAGTTCCCGGACCTCGCTTTCCGTATTCATGGGAAG

C.3.4 CAGATAACGTGTTTCCTATGTTCGAAGTTCCCGGACCTCGCTTTCCGTATTCATGGGAAG

*************** ****

J.5.2 ------------------------------------------------------------

J.5.6 ------------------------------------------------------------

J.5.3 ------------------------------------------------------------

J.6.4 ------------------------------------------------------------

C.3.6 GTGGAAATGATCCAGGATACGTAAACGAGATGTTCATAGCCTTGCAGCACATGATATCTT

C.4.6 GTGGAAATGATCCAGGATACGTAAACGAGATGTTCATAGCCTTGCAGCACATGATATCTT

C.4.8 GTGGAAATGATCCAGGATACGTAAACGAGATGTTCATAGCCTTGCAGCACATGATATCTT

C.4.10 GTGGAAATGATCCAGGATACGTAAACGAGATGTTCATAGCCTTGCAGCACATGATATCTT

A.2.9 GTGGAAATGATCCAGGATACGTAAACGAGATGTTCATAGCCTTGCAGCACATGATATCTT

A.1.1 GTGGAAATGATCCAGGATACGTAAACGAGATGTTCATAGCCTTGCAGCACATGATATCTT

A.2.2 GTGGAAATGATCCAGGATACGTAAACGAGATGTTCATAGCCTTGCAGCACATGATATCTT

APHIS-S GTGGAAATGATCCAGGATACGTAAACGAGATGTTCATAGCCTTGCAGCACATGATATCTT

C.3.4 GTGGAAATGATCCAGGATACGTAAACGAGATGTTCATAGCCTTGCAGCACATGATATCTT

J.5.2 ------------------------------------------------ACACACA----T

J.5.6 ------------------------------------------------ACACACA----T

J.5.3 ------------------------------------------------ACACACA----T

J.6.4 ------------------------------------------------ACACACA----T

C.3.6 CAGAACTGGTATCTAAAGTGGCGGGAGTGAACCTAGACTTCGATGTGCACATACAGAGGT

C.4.6 CAGAACTGGTATCTAAAGTGGCGGGAGTGAACCTAGACTTCGATGTGCACATACAGAGGT

C.4.8 CAGAACTGGTATCTAAAGTGGCGGGAGTGAACCTAGACTTCGATGTGCACATACAGAGGT

C.4.10 CAGAACTGGTATCTAAAGTGGCGGGAGTGAACCTAGACTTCGATGTGCACATACAGAGGT

A.2.9 CAGAACTGGTATCTAAAGTGGCGGGAGTGAACCTAGACTTCGATGTGCACATACAGAGGT

A.1.1 CAGAACTGGTATCTAAAGTGGCGGGAGTGAACCTAGACTTCGATGTGCACATACAGAGGT

A.2.2 CAGAACTGGTATCTAAAGTGGCGGGAGTGAACCTAGACTTCGATGTGCACATACAGAGGT

APHIS-S CAGAACTGGTATCTAAAGTGGCGGGAGTGAACCTAGACTTCGATGTGCACATACAGAGGT

C.3.4 CAGAACTGGTATCTAAAGTGGCGGGAGTGAACCTAGACTTCGATGTGCACATACAGAGGT

*** *** *

J.5.2 ACACATATCTAATGCGTATC----------------------------------------

J.5.6 ACACATATCTAATGCGTATC----------------------------------------

J.5.3 ACACATATCTAATGCGTATC----------------------------------------

J.6.4 ACACATATCTAATGCGTATC----------------------------------------

C.3.6 ACCCACATCCAGCATACATCATGGACTTGGCGAAGGAAGCCCTGCAGTTCCTCTTCCCAT

C.4.6 ACCCACATCCAGCATACATCATGGACTTGGCGAAGGAAGCCCTGCAGTTCCTCTTCCCAT

C.4.8 ACCCACATCCAGCATACATCATGGACTTGGCGAAGGAAGCCCTGCAGTTCCTCTTCCCAT

C.4.10 ACCCACATCCAGCATACATCATGGACTTGGCGAAGGAAGCCCTGCAGTTCCTCTTCCCAT

A.2.9 ACCCACATCCAGCATACATCATGGACTTGGCGAAGGAAGCCCTGCAGTTCCTCTTCCCAT

A.1.1 ACCCACATCCAGCATACATCATGGACTTGGCGAAGGAAGCCCTGCAGTTCCTCTTCCCAT

A.2.2 ACCCACATCCAGCATACATCATGGACTTGGCGAAGGAAGCCCTGCAGTTCCTCTTCCCAT

APHIS-S ACCCACATCCAGCATACATCATGGACTTGGCGAAGGAAGCCCTGCAGTTCCTCTTCCCAT

C.3.4 ACCCACATCCAGCATACATCATGGACTTGGCGAAGGAAGCCCTGCAGTTCCTCTTCCCAT

** ** *** * ***

J.5.2 ------------------------------------------------------------

J.5.6 ------------------------------------------------------------

J.5.3 ------------------------------------------------------------

J.6.4 ------------------------------------------------------------

C.3.6 CATTCATCATGATCAGCTTCAGTTACACCGCTATCAATATTATACGATCCGTGACCGTGG

C.4.6 CATTCATCATGATCAGCTTCAGTTACACCGCTATCAATATTATACGATCCGTGACCGTGG

C.4.8 CATTCATCATGATCAGCTTCAGTTACACCGCTATCAATATTATACGATCCGTGACCGTGG

C.4.10 CATTCATCATGATCAGCTTCAGTTACACCGCTATCAATATTATACGATCCGTGACCGTGG

A.2.9 CATTCATCATGATCAGCTTCAGTTACACCGCTATCAATATTATACGATCCGTGACCGTGG

A.1.1 CATTCATCATGATCAGCTTCAGTTACACCGCTATCAATATTATACGATCCGTGACCGTGG

A.2.2 CATTCATCATGATCAGCTTCAGTTACACCGCTATCAATATTATACGATCCGTGACCGTGG

APHIS-S CATTCATCATGATCAGCTTCAGTTACACCGCTATCAATATTATACGATCCGTGACCGTGG

C.3.4 CATTCATCATGATCAGCTTCAGTTACACCGCTATCAATATTATACGATCCGTGACCGTGG

J.5.2 ------------------------------------------------------------

J.5.6 ------------------------------------------------------------

J.5.3 ------------------------------------------------------------

J.6.4 ------------------------------------------------------------

C.3.6 AAAAAGAAATGCAATTGAAGGAAACGATGAAGATCATGGGACTCCCAACGTGGCTGCATT

C.4.6 AAAAAGAAATGCAATTGAAGGAAACGATGAAGATCATGGGACTCCCAACGTGGCTGCATT

C.4.8 AAAAAGAAATGCAATTGAAGGAAACGATGAAGATCATGGGACTCCCAACGTGGCTGCATT

C.4.10 AAAAAGAAATGCAATTGAAGGAAACGATGAAGATCATGGGACTCCCAACGTGGCTGCATT

A.2.9 AAAAAGAAATGCAATTGAAGGAAACGATGAAGATCATGGGACTCCCAACGTGGCTGCATT

A.1.1 AAAAAGAAATGCAATTGAAGGAAACGATGAAGATCATGGGACTCCCAACGTGGCTGCATT

A.2.2 AAAAAGAAATGCAATTGAAGGAAACGATGAAGATCATGGGACTCCCAACGTGGCTGCATT

APHIS-S AAAAAGAAATGCAATTGAAGGAAACGATGAAGATCATGGGACTCCCAACGTGGCTGCATT

C.3.4 AAAAAGAAATGCAATTGAAGGAAACGATGAAGATCATGGGACTCCCAACGTGGCTGCATT

J.5.2 -----------------------TCTATTGAT----------------------------

J.5.6 -----------------------TCTATTGAT----------------------------

J.5.3 -----------------------TCTATTGAT----------------------------

J.6.4 -----------------------TCTATTGAT----------------------------

C.3.6 GGATGGCATGGTTTTTTAAACAATTTATTTATTTGCTGATTGCTTCGGTTTTGATACTTG

C.4.6 GGATGGCATGGTTTTTTAAACAATTTATTTATTTGCTGATTGCTTCGGTTTTGATACTTG

C.4.8 GGATGGCATGGTTTTTTAAACAATTTATTTATTTGCTGATTGCTTCGGTTTTGATACTTG

C.4.10 GGATGGCATGGTTTTTTAAACAATTTATTTATTTGCTGATTGCTTCGGTTTTGATACTTG

A.2.9 GGATGGCATGGTTTTTTAAACAATTTATTTATTTGCTGATTGCTTCGGTTTTGATACTTG

A.1.1 GGATGGCATGGTTTTTTAAACAATTTATTTATTTGCTGATTGCTTCGGTTTTGATACTTG

A.2.2 GGATGGCATGGTTTTTTAAACAATTTATTTATTTGCTGATTGCTTCGGTTTTGATACTTG

APHIS-S GGATGGCATGGTTTTTTAAACAATTTATTTATTTGCTGATTGCTTCGGTTTTGATACTTG

C.3.4 GGATGGCATGGTTTTTTAAACAATTTATTTATTTGCTGATTGCTTCGGTTTTGATACTTG

* **** **

J.5.2 ------------------------------------------------------------

J.5.6 ------------------------------------------------------------

J.5.3 ------------------------------------------------------------

J.6.4 ------------------------------------------------------------

C.3.6 TTATATTAAAGGTAAATTGGTTTACTACAGAAGAAGGCTTTAGCGACTATGCCGTATTCA

C.4.6 TTATATTAAAG-------------------------------------------------

C.4.8 TTATATTAAAGGTAAATTGGTTTACTACAGAAGAAGGCTTTAGCGACTATGCCGTATTCA

C.4.10 TTATATTAAAGGTAAATTGGTTTACTACAGAAGAAGGCTTTAGCGACTATGCCGTATTCA

A.2.9 TTATATTAAAG-------------------------------------------------

A.1.1 TTATATTAAAGGTAAATTGGTTTACTACAGAAGAAGGCTTTAGCGACTATGCCGTATTCA

A.2.2 TTATATTAAAGGTAAATTGGTTTACTACAGAAGAAGGCTTTAGCGACTATGCCGTATTCA

APHIS-S TTATATTAAAGGTAAATTGGTTTACTACAGAAGAAGGCTTTAGCGACTATGCCGTATTCA

C.3.4 TTATATTAAAG-------------------------------------------------

J.5.2 ------------------------------------------------------------

J.5.6 ------------------------------------------------------------

J.5.3 ------------------------------------------------------------

J.6.4 ------------------------------------------------------------

C.3.6 CTAATACACCTTGGACCGTCCTCTTCTTCTTCCTAACACTGTATCTTACGTGTACCATAT

C.4.6 ------------------------------------------------------------

C.4.8 CTAATACACCTTGGACCGTCCTCTTCTTCTTCCTAACACTGTATCTTACGTGTACCATAT

C.4.10 CTAATACACCTTGGACCGTCCTCTTCTTCTTCCTAACACTGTATCTTACGTGTACCATAT

A.2.9 ------------------------------------------------------------

A.1.1 CTAATACACCTTGGACCGTCCTCTTCTTCTTCCTAACACTGTATCTTACGTGTACCATAT

A.2.2 CTAATACACCTTGGACCGTCCTCTTCTTCTTCCTAACACTGTATCTTACGTGTACCATAT

APHIS-S CTAATACACCTTGGACCGTCCTCTTCTTCTTCCTAACACTGTATCTTACGTGTACCATAT

C.3.4 ------------------------------------------------------------

J.5.2 --------------------------------AAAG----------------GTTTAGTG

J.5.6 --------------------------------AAAG----------------GTTTAGTG

J.5.3 --------------------------------AAAG----------------GTTTAGTG

J.6.4 --------------------------------AAAG----------------GTTTAGTG

C.3.6 TTTTCTGTTTCATGATAAGTGGTTTCTTTTCAAAAGCCAGTACAGCGGCGTTGTTTGGTG

C.4.6 ------------------------------------CCAGTACAGCGGCGTTGTTTGGTG

C.4.8 TTTTCTGTTTCATGATAAGTGGTTTCTTTTCAAAAGCCAGTACAGCGGCGTTGTTTGGTG

C.4.10 TTTTCTGTTTCATGATAAGTGGTTTCTTTTCAAAAGCCAGTACAGCGGCGTTGTTTGGTG

A.2.9 ------------------------------------GCAGTACAGCGGCGTTGTTTGGTG

A.1.1 TTTTCTGTTTCATGATAAGTGGTTTCTTTTCAAAAGCCAGTACAGCGGCGTTGTTTGGTG

A.2.2 TTTTCTGTTTCATGATAAGTGGTTTCTTTTCAAAAGCCAGTACAGCGGCGTTGTTTGGTG

APHIS-S TTTTCTGTTTCATGATAAGTGGTTTCTTTTCAAAAGCCAGTACAGCGGCGTTGTTTGGTG

C.3.4 ------------------------------------CCAGTACAGCGGCGTTGTTTGGTG

**** ***

J.5.2 ---------TATGTTTGACG----------------------------------------

J.5.6 ---------TATGTTTGACG----------------------------------------

J.5.3 ---------TATGTTTGACG----------------------------------------

J.6.4 ---------TATGTTTGACG----------------------------------------

C.3.6 GGGTGATCTGGTTTCTGACGTATATCCCCGCATTCCTCCTGGCTATGGACGTGAACATGT

C.4.6 GGGTGATCTGGTTTCTGACGTATATCCCCGCATTCCTCCTGGCTATGGACGTGAACATGT

C.4.8 GGGTGATCTGGTTTCTGACGTATATCCCCGCATTCCTCCTGGCTATGGACGTGAACATGT

C.4.10 GGGTGATCTGGTTTCTGACGTATATCCCCGCATTCCTCCTGGCTATGGACGTGAACATGT

A.2.9 GGGTGATCTGGTTTCTGACGTATATCCCCGCATTCCTCCTGGCTATGGACGTGAACATGT

A.1.1 GGGTGATCTGGTTTCTGACGTATATCCCCGCATTCCTCCTGGCTATGGACGTGAACATGT

A.2.2 GGGTGATCTGGTTTCTGACGTATATCCCCGCATTCCTCCTGGCTATGGACGTGAACATGT

APHIS-S GGGTGATCTGGTTTCTGACGTATATCCCCGCATTCCTCCTGGCTATGGACGTGAACATGT

C.3.4 GGGTGATCTGGTTTCTGACGTATATCCCCGCATTCCTCCTGGCTATGGACGTGAACATGT

* * *****

J.5.2 ------------------------------TGCTTGACTCC-------------------

J.5.6 ------------------------------TGCTTGACTCC-------------------

J.5.3 ------------------------------TGCTTGACTCC-------------------

J.6.4 ------------------------------TGCTTGACTCC-------------------

C.3.6 CTACCTCTCTACAAGCGGTCACCTGCCTAATGCTCAACTCCGCCATGTCTTACGGCTTCC

C.4.6 CTACCTCTCTACAAGCGGTCACCTGCCTAATGCTCAACTCCGCCATGTCTTACGGCTTCC

C.4.8 CTACCTCTCTACAAGCGGTCACCTGCCTAATGCTCAACTCCGCCATGTCTTACGGCTTCC

C.4.10 CTACCTCTCTACAAGCGGTCACCTGCCTAATGCTCAACTCCGCCATGTCTTACGGCTTCC

A.2.9 CTACCTCTCTACAAGCGGTCACCTGCCTAATGCTCAACTCCGCCATGTCTTACGGCTTCC

A.1.1 CTACCTCTCTACAAGCGGTCACCTGCCTAATGCTCAACTCCGCCATGTCTTACGGCTTCC

A.2.2 CTACCTCTCTACAAGCGGTCACCTGCCTAATGCTCAACTCCGCCATGTCTTACGGCTTCC

APHIS-S CTACCTCTCTACAAGCGGTCACCTGCCTAATGCTCAACTCCGCCATGTCTTACGGCTTCC

C.3.4 CTACCTCTCTACAAGCGGTCACCTGCCTAATGCTCAACTCCGCCATGTCTTACGGCTTCC

**** *****

J.5.2 ------------------------------------------------------------

J.5.6 ------------------------------------------------------------

J.5.3 ------------------------------------------------------------

J.6.4 ------------------------------------------------------------

C.3.6 AGCTGTTACTGGCCCGGGAAAGTACCGGAGGAATGCAGTGGGGTGATTTTATGACGTCAC

C.4.6 AGCTGTTACTGGCCCGGGAAAGTACCGGAGGAATGCAGTGGGGTGATTTTATGACGTCAC

C.4.8 AGCTGTTACTGGCCCGGGAAAGTACCGGAGGAATGCAGTGGGGTGATTTTATGACGTCAC

C.4.10 AGCTGTTACTGGCCCGGGAAAGTACCGGAGGAATGCAGTGGGGTGATTTTATGACGTCAC

A.2.9 AGCTGTTACTGGCCCGGGAAAGTACCGGAGGAATGCAGTGGGGTGATTTTATGACGTCAC

A.1.1 AGCTGTTACTGGCCCGGGAAAGTACCGGAGGAATGCAGTGGGGTGATTTTATGACGTCAC

A.2.2 AGCTGTTACTGGCCCGGGAAAGTACCGGAGGAATGCAGTGGGGTGATTTTATGACGTCAC

APHIS-S AGCTGTTACTGGCCCGGGAAAGTACCGGAGGAATGCAGTGGGGTGATTTTATGACGTCAC

C.3.4 AGCTGTTACTGGCCCGGGAAAGTACCGGAGGAATGCAGTGGGGTGATTTTATGACGTCAC

J.5.2 ------------------------------------------------------------

J.5.6 ------------------------------------------------------------

J.5.3 ------------------------------------------------------------

J.6.4 ------------------------------------------------------------

C.3.6 CAGCAACGGACTCGTCACGATTCGTATTCGGTCACGTCGTTATAATGATGGCTTTGAACT

C.4.6 CAGCAACGGACTCGTCACGATTCGTATTCGGTCACGTCGTTATAATGATGGCTTTGAACT

C.4.8 CAGCAACGGACTCGTCACGATTCGTATTCGGTCACGTCGTTATAATGATGGCTTTGAACT

C.4.10 CAGCAACGGACTCGTCACGATTCGTATTCGGTCACGTCGTTATAATGATGGCTTTGAACT

A.2.9 CAGCAACGGACTCGTCACGATTCGTATTCGGTCACGTCGTTATAATGATGGCTTTGAACT

A.1.1 CAGCAACGGACTCGTCACGATTCGTATTCGGTCACGTCGTTATAATGATGGCTTTGAACT

A.2.2 CAGCAACGGACTCGTCACGATTCGTATTCGGTCACGTCGTTATAATGATGGCTTTGAACT

APHIS-S CAGCAACGGACTCGTCACGATTCGTATTCGGTCACGTCGTTATAATGATGGCTTTGAACT

C.3.4 CAGCAACGGACTCGTCACGATTCGTATTCGGTCACGTCGTTATAATGATGGCTTTGAACT

J.5.2 ------------------------------------------------------------

J.5.6 ------------------------------------------------------------

J.5.3 ------------------------------------------------------------

J.6.4 ------------------------------------------------------------

C.3.6 GTGTGCTCTACATGTTGATTGCCCTATATCTAGAGCAAGTACTACCCGGGCCGTATGGCA

C.4.6 GTGTGCTCTACATGTTGATTGCCCTATATCTAGAGCAAGTACTACCCGGGCCGTATGGCA

C.4.8 GTGTGCTCTACATGTTGATTGCCCTATATCTAGAGCAAGTACTACCCGGGCCGTATGGCA

C.4.10 GTGTGCTCTACATGTTGATTGCCCTATATCTAGAGCAAGTACTACCCGGGCCGTATGGCA

A.2.9 GTGTGCTCTACATGTTGATTGCCCTATATCTAGAGCAAGTACTACCCGGGCCGTATGGCA

A.1.1 GTGTGCTCTACATGTTGATTGCCCTATATCTAGAGCAAGTACTACCCGGGCCGTATGGCA

A.2.2 GTGTGCTCTACATGTTGATTGCCCTATATCTAGAGCAAGTACTACCCGGGCCGTATGGCA

APHIS-S GTGTGCTCTACATGTTGATTGCCCTATATCTAGAGCAAGTACTACCCGGGCCGTATGGCA

C.3.4 GTGTGCTCTACATGTTGATTGCCCTATATCTAGAGCAAGTACTACCCGGGCCGTATGGCA

J.5.2 ------------AGTATTGTTTAGT-----------------------------------

J.5.6 ------------AGTATTGTTTAGT-----------------------------------

J.5.3 ------------AGTATTGTTTAGT-----------------------------------

J.6.4 ------------AGTATTGTTTAGT-----------------------------------

C.3.6 CACCGAAGCCCTGGTATTTCTTCGTCCAAAGACAGTTCTGGTGTAGCAGCAAAACTACTC

C.4.6 CACCGAAGCCCTGGTATTTCTTCGTCCAAAGACAGTTCTGGTGTAGCAGCAAAACTACTC

C.4.8 CACCGAAGCCCTGGTATTTCTTCGTCCAAAGACAGTTCTGGTGTAGCAGCAAAACTACTC

C.4.10 CACCGAAGCCCTGGTATTTCTTCGTCCAAAGACAGTTCTGGTGTAGCAGCAAAACTACTC

A.2.9 CACCGAAGCCCTGGTATTTCTTCGTCCAAAGACAGTTCTGGTGTAGCAGCAAAACTACTC

A.1.1 CACCGAAGCCCTGGTATTTCTTCGTCCAAAGACAGTTCTGGTGTAGCAGCAAAACTACTC

A.2.2 CACCGAAGCCCTGGTATTTCTTCGTCCAAAGACAGTTCTGGTGTAGCAGCAAAACTACTC

APHIS-S CACCGAAGCCCTGGTATTTCTTCGTCCAAAGACAGTTCTGGTGTAGCAGCAAAACTACTC

C.3.4 CACCGAAGCCCTGGTATTTCTTCGTCCAAAGACAGTTCTGGTGTAGCAGCAAAACTACTC

***** ** **

J.5.2 ------------------------------------------------------------

J.5.6 ------------------------------------------------------------

J.5.3 ------------------------------------------------------------

J.6.4 ------------------------------------------------------------

C.3.6 ATGATATCGGTACAGACAACAGCGACACATCAAGTTTAACAAAAGAAAGCGACCCTACAG

C.4.6 ATGATATCGGTACAGACAACAGCGACACATCAAGTTTAACAAAAGAAAGCGACCCTACAG

C.4.8 ATGATATCGGTACAGACAACAGCGACACATCAAGTTTAACAAAAGAAAGCGACCCTACAG

C.4.10 ATGATATCGGTACAGACAACAGCGACACATCAAGTTTAACAAAAGAAAGCGACCCTACAG

A.2.9 ATGATATCGGTACAGACAACAGCGACACATCAAGTTTAACAAAAGAAAGCGACCCTACAG

A.1.1 ATGATATCGGTACAGACAACAGCGACACATCAAGTTTAACAAAAGAAAGCGACCCTACAG

A.2.2 ATGATATCGGTACAGACAACAGCGACACATCAAGTTTAACAAAAGAAAGCGACCCTACAG

APHIS-S ATGATATCGGTACAGACAACAGCGACACATCAAGTTTAACAAAAGAAAGCGACCCTACAG

C.3.4 ATGATATCGGTACAGACAACAGCGACACATCAAGTTTAACAAAAGAAAGCGACCCTACAG

J.5.2 ------------------------------------------------------------

J.5.6 ------------------------------------------------------------

J.5.3 ------------------------------------------------------------

J.6.4 ------------------------------------------------------------

C.3.6 ACCTTCCGATTGGAGTTAAAATACAAAACCTTAAAAAGGTTTACGGGAGCAACGTTGCGG

C.4.6 ACCTTCCGATTGGAGTTAAAATACAAAACCTTAAAAAGGTTTACGGGAGCAACGTTGCGG

C.4.8 ACCTTCCGATTGGAGTTAAAATACAAAACCTTAAAAAGGTTTACGGGAGCAACGTTGCGG

C.4.10 ACCTTCCGATTGGAGTTAAAATACAAAACCTTAAAAAGGTTTACGGGAGCAACGTTGCGG

A.2.9 ACCTTCCGATTGGAGTTAAAATACAAAACCTTAAAAAGGTTTACGGGAGCAACGTTGCGG

A.1.1 ACCTTCCGATTGGAGTTAAAATACAAAACCTTAAAAAGGTTTACGGGAGCAACGTTGCGG

A.2.2 ACCTTCCGATTGGAGTTAAAATACAAAACCTTAAAAAGGTTTACGGGAGCAACGTTGCGG

APHIS-S ACCTTCCGATTGGAGTTAAAATACAAAACCTTAAAAAGGTTTACGGGAGCAACGTTGCGG

C.3.4 ACCTTCCGATTGGAGTTAAAATACAAAACCTTAAAAAGGTTTACGGGAGCAACGTTGCGG

J.5.2 ------------------------------------------------------------

J.5.6 ------------------------------------------------------------

J.5.3 ------------------------------------------------------------

J.6.4 ------------------------------------------------------------

C.3.6 TAAACAATTTATCCCTCAACATTTACGACGACCAAATCACGGTTCTACTTGGACACAACG

C.4.6 TAAACAATTTATCCCTCAACATTTACGACGACCAAATCACGGTTCTACTTGGACACAACG

C.4.8 TAAACAATTTATCCCTCAACATTTACGACGACCAAATCACGGTTCTACTTGGACACAACG

C.4.10 TAAACAATTTATCCCTCAACATTTACGACGACCAAATCACGGTTCTACTTGGACACAACG

A.2.9 TAAACAATTTATCCCTCAACATTTACGACGACCAAATCACGGTTCTACTTGGACACAACG

A.1.1 TAAACAATTTATCCCTCAACATTTACGACGACCAAATCACGGTTCTACTTGGACACAACG

A.2.2 TAAACAATTTATCCCTCAACATTTACGACGACCAAATCACGGTTCTACTTGGACACAACG

APHIS-S TAAACAATTTATCCCTCAACATTTACGACGACCAAATCACGGTTCTACTTGGACACAACG

C.3.4 TAAACAATTTATCCCTCAACATTTACGACGACCAAATCACGGTTCTACTTGGACACAACG

J.5.2 ------------------------------------GTAAAGTGTGCATAGTTCCCGAGT

J.5.6 ------------------------------------GTAAAGTGTGCATAGTTCCCGAGT

J.5.3 ------------------------------------GTAAAGTGTGCATAGTTCCCGAGT

J.6.4 ------------------------------------GTAAAGTGTGCATAGTTCCCGAGT

C.3.6 GAGCGGGAAAATCCACAACCATTTCAATGCTCACAGGTAACGTGGACATAACCAGCGGGT

C.4.6 GAGCGGGAAAATCCACAACCATTTCAATGCTCACAGGTAACGTGGACATAACCAGCGGGT

C.4.8 GAGCGGGAAAATCCACAACCATTTCAATGCTCACAGGTAACGTGGACATAACCAGCGGGT

C.4.10 GAGCGGGAAAATCCACAACCATTTCAATGCTCACAGGTAACGTGGACATAACCAGCGGGT

A.2.9 GAGCGGGAAAATCCACAACCATTTCAATGCTCACAGGTAACGTGGACATAACCAGCGGGT

A.1.1 GAGCGGGAAAATCCACAACCATTTCAATGCTCACAGGTAACGTGGACATAACCAGCGGGT

A.2.2 GAGCGGGAAAATCCACAACCATTTCAATGCTCACAGGTAACGTGGACATAACCAGCGGGT

APHIS-S GAGCGGGAAAATCCACAACCATTTCAATGCTCACAGGTAACGTGGACATAACCAGCGGGT

C.3.4 GAGCGGGAAAATCCACAACCATTTCAATGCTCACAGGTAACGTGGACATAACCAGCGGGT

**** *** **** ** **

J.5.2 ------------------------------------------------------------

J.5.6 ------------------------------------------------------------

J.5.3 ------------------------------------------------------------

J.6.4 ------------------------------------------------------------

C.3.6 CGGTGACGGTGGCTGGCTACGACATAGAAAAACAAACAAGTTCAGCACGCTCACACATTG

C.4.6 CGGTGACGGTGGCTGGCTACGACATAGAAAAACAAACAAGTTCAGCACGCTCACACATTG

C.4.8 CGGTGACGGTGGCTGGCTACGACATAGAAAAACAAACAAGTTCAGCACGCTCACACATTG

C.4.10 CGGTGACGGTGGCTGGCTACGACATAGAAAAACAAACAAGTTCAGCACGCTCACACATTG

A.2.9 CGGTGACGGTGGCTGGCTACGACATAGAAAAACAAACAAGTTCAGCACGCTCACACATTG

A.1.1 CGGTGACGGTGGCTGGCTACGACATAGAAAAACAAACAAGTTCAGCACGCTCACACATTG

A.2.2 CGGTGACGGTGGCTGGCTACGACATAGAAAAACAAACAAGTTCAGCACGCTCACACATTG

APHIS-S CGGTGACGGTGGCTGGCTACGACATAGAAAAACAAACAAGTTCAGCACGCTCACACATTG

C.3.4 CGGTGACGGTGGCTGGCTACGACATAGAAAAACAAACAAGTTCAGCACGCTCACACATTG

J.5.2 --------------TATAGTGTGTACCT--------------------------------

J.5.6 --------------TATAGTGTGTACCT--------------------------------

J.5.3 --------------TATAGTGTGTACCT--------------------------------

J.6.4 --------------TATAGTGTGTACCT--------------------------------

C.3.6 GACTCTGCCCTCAACATAACGTACTCTTCAACGAACTCACAGTCAAAGAACATTTACAGT

C.4.6 GACTCTGCCCTCAACATAACGTACTCTTCAACGAACTCACAGTCAAAGAACATTTACAGT

C.4.8 GACTCTGCCCTCAACATAACGTACTCTTCAACGAACTCACAGTCAAAGAACATTTACAGT

C.4.10 GACTCTGCCCTCAACATAACGTACTCTTCAACGAACTCACAGTCAAAGAACATTTACAGT

A.2.9 GACTCTGCCCTCAACATAACGTACTCTTCAACGAACTCACAGTCAAAGAACATTTACAGT

A.1.1 GACTCTGCCCTCAACATAACGTACTCTTCAACGAACTCACAGTCAAAGAACATTTACAGT

A.2.2 GACTCTGCCCTCAACATAACGTACTCTTCAACGAACTCACAGTCAAAGAACATTTACAGT

APHIS-S GACTCTGCCCTCAACATAACGTACTCTTCAACGAACTCACAGTCAAAGAACATTTACAGT

C.3.4 GACTCTGCCCTCAACATAACGTACTCTTCAACGAACTCACAGTCAAAGAACATTTACAGT

*** ** * *

J.5.2 ----------TCTAACAGG-----------------------------------------

J.5.6 ----------TCTAACAGG-----------------------------------------

J.5.3 ----------TCTAACAGG-----------------------------------------

J.6.4 ----------TCTAACAGG-----------------------------------------

C.3.6 TCTTCTCTCGTCTGAAAGGCTTCAGCGGTAAAGAGTTGGATGAAGAAATTGAGACGCTTA

C.4.6 TCTTCTCTCGTCTGAAAGGCTTCAGCGGTAAAGAGTTGGATGAAGAAATTGAGACGCTTA

C.4.8 TCTTCTCTCGTCTGAAAGGCTTCAGCGGTAAAGAGTTGGATGAAGAAATTGAGACGCTTA

C.4.10 TCTTCTCTCGTCTGAAAGGCTTCAGCGGTAAAGAGTTGGATGAAGAAATTGAGACGCTTA

A.2.9 TCTTCTCTCGTCTGAAAGGCTTCAGCGGTAAAGAGTTGGATGAAGAAATTGAGACGCTTA

A.1.1 TCTTCTCTCGTCTGAAAGGCTTCAGCGGTAAAGAGTTGGATGAAGAAATTGAGACGCTTA

A.2.2 TCTTCTCTCGTCTGAAAGGCTTCAGCGGTAAAGAGTTGGATGAAGAAATTGAGACGCTTA

APHIS-S TCTTCTCTCGTCTGAAAGGCTTCAGCGGTAAAGAGTTGGATGAAGAAATTGAGACGCTTA

C.3.4 TCTTCTCTCGTCTGAAAGGCTTCAGCGGTAAAGAGTTGGATGAAGAAATTGAGACGCTTA

*** * ***

J.5.2 -----AATTTGGATTTG-------------------------------------------

J.5.6 -----AATTTGGATTTG-------------------------------------------

J.5.3 -----AATTTGGATTTG-------------------------------------------

J.6.4 -----AATTTGGATTTG-------------------------------------------

C.3.6 TTGAAAAATTGGAATTGCAAGAAAAGAGGGATTACCAATCAGCGGGGTTATCAGGGGGAC

C.4.6 TTGAAAAATTGGAATTGCAAGAAAAGAGGGATTACCAATCAGCGGGGTTATCAGGGGGAC

C.4.8 TTGAAAAATTGGAATTGCAAGAAAAGAGGGATTACCAATCAGCGGGGTTATCAGGGGGAC

C.4.10 TTGAAAAATTGGAATTGCAAGAAAAGAGGGATTACCAATCAGCGGGGTTATCAGGGGGAC

A.2.9 TTGAAAAATTGGAATTGCAAGAAAAGAGGGATTACCAATCAGCGGGGTTATCAGGGGGAC

A.1.1 TTGAAAAATTGGAATTGCAAGAAAAGAGGGATTACCAATCAGCGGGGTTATCAGGGGGAC

A.2.2 TTGAAAAATTGGAATTGCAAGAAAAGAGGGATTACCAATCAGCGGGGTTATCAGGGGGAC

APHIS-S TTGAAAAATTGGAATTGCAAGAAAAGAGGGATTACCAATCAGCGGGGTTATCAGGGGGAC

C.3.4 TTGAAAAATTGGAATTGCAAGAAAAGAGGGATTACCAATCAGCGGGGTTATCAGGGGGAC

** ***** ***

J.5.2 ------------------------------------------------------------

J.5.6 ------------------------------------------------------------

J.5.3 ------------------------------------------------------------

J.6.4 ------------------------------------------------------------

C.3.6 AGAAGCGACGATTAGGAGTGGGCGTCGCGCTATGCGGGGCGGCTAAAGTGGTTCTACTGG

C.4.6 AGAAGCGACGATTAGGAGTGGGCGTC-------GCGGAGCGGCTAAAGTGGTTCTACTGG

C.4.8 AGAAGCGACGATTAGGAGTGGGCGTC-------GCGGGGCGGCTAAAGTGGTTCTACTGG

C.4.10 AGAAGCGACGATTAGGAGTGGGCGTC-------GCGGGGCGGCTAAAGTGGTTCTACTGG

A.2.9 AGAAGCGACGATTAGGAGTGGGCGTCGCGCTATGCGGGGCGGCTAAAGTGGTTCTACTGG

A.1.1 AGAAGCGACGATTAGGAGTGGGCGTCGCGCTATGCGGGGCGGCTAAAGTGGTTCTACTGG

A.2.2 AGAAGCGACGATTAGGAGTGGGCGTCGCGCTATGCGGGGCGGCTAAAGTGGTTCTACTGG

APHIS-S AGAAGCGACGATTAGGAGTGGGCGTCGCGCTATGCGGGGCGGCTAAAGTGGTTCTACTGG

C.3.4 AGAAGCGACGATTAGGAGTGGGCGTCGCGCTATGCGGGGCGGCTAAAGTGGTTCTACTGG

J.5.2 ------------------------------------------------------------

J.5.6 ------------------------------------------------------------

J.5.3 ------------------------------------------------------------

J.6.4 ------------------------------------------------------------

C.3.6 ACGAGCCCACTTCTGGCATGGACCCGGCCTCACGTCGTGCCCTATGGGACTTGTTGCAGA

C.4.6 ACGAGCCCACTTCTGGCATGGACCCGGCCTCACGTCGTGCCCTATGGGACTTGTTGCAGA

C.4.8 ACGAGCCCACTTCTGGCATGGACCCGGCCTCACGTCGTGCCCTATGGGACTTGTTGCAGA

C.4.10 ACGAGCCCACTTCTGGCATGGACCCGGCCTCACGTCGTGCCCTATGGGACTTGTTGCAGA

A.2.9 ACGAGCCCACTTCTGGCATGGACCCGGCCTCACGTCGTGCCCTATGGGACTTGTTGCAGA

A.1.1 ACGAGCCCACTTCTGGCATGGACCCGGCCTCACGTCGTGCCCTATGGGACTTGTTGCAGA

A.2.2 ACGAGCCCACTTCTGGCATGGACCCGGCCTCACGTCGTGCCCTATGGGACTTGTTGCAGA

APHIS-S ACGAGCCCACTTCTGGCATGGACCCGGCCTCACGTCGTGCCCTATGGGACTTGTTGCAGA

C.3.4 ACGAGCCCACTTCTGGCATGGACCCGGCCTCACGTCGTGCCCTATGGGACTTGTTGCAGA

J.5.2 ---------AGGGTCGATCGATGATCCTGACGACACACTTCATGGACGAAGCGGACATAT

J.5.6 ---------AGGGTCGATCGATGATCCTGACGACACACTTCATGGACGAAGCGGACATAT

J.5.3 ---------AGGGTCGATCGATGATCCTGACGACACACTTCATGGACGAAGCGGACATAT

J.6.4 ---------AGGGTCGATCGATGATCCTGACGACACACTTCATGGACGAAGCGGACATAT

C.3.6 GAGAGAAGAAAGGTCGATCGATGATCCTGACGACACACTTCATGGACGAAGCGGACATAT

C.4.6 GAGAGAAGAAAGGTCGATCGATGATCCTGACGACACACTTCATGGACGAAGCGGACATAT

C.4.8 GAGAGAAGAAAGGTCGATCGATGATCCTGACGACACACTTCATGGACGAAGCGGACATAT

C.4.10 GAGAGAAGAAAGGTCGATCGATGATCCTGACGACACACTTCATGGAC-AAGCGGACATAT

A.2.9 GAGAGAAGAAAGGTCGATCGATGATCCTGACGACACACTTCATGGACGAAGCGGACATAT

A.1.1 GAGAGAAGAAAGGTCGATCGATGATCCTGACGACACACTTCATGGACGAAGCGGACATAT

A.2.2 GAGAGAAGAAAGGTCGATCGATGATCCTGACGACACACTTCATGGACGAAGCGGACATAT

APHIS-S GAGAGAAGAAAGGTCGATCGATGATCCTGACGACACACTTCATGGACGAAGCGGACATAT

C.3.4 GAGAGAAGAAAGGTCGATCGATGATCCTGACGACACACTTCATGGACGAAGCGGACATAT

* ************************************ ************

J.5.2 TAGGGGATAGAGTTGCCATTATGGCGGACGGTCGTCTCCAGTGCGTGGGCTCACCTTACT

J.5.6 TAGGGGATAGAGTTGCCATTATGGCGGACGGTCGTCTCCAGTGCGTGGGCTCACCTTACT

J.5.3 TAGGGGATAGAGTTGCCATTATGGCGGACGGTCGTCTCCAGTGCGTGGGCTCACCTTACT

J.6.4 TAGGGGATAGAGTTGCCATTATGGCGGACGGTCGTCTCCAGTGCGTGGGCTCACCTTACT

C.3.6 TAGGGGATAGAGTTGCCATTATGGCGGACGGTCGTCTCCAGTGCGTGGGCTCACCTTACT

C.4.6 TAGGGGATAGAGTTGCCATTATGGCGGACGGTCGTCTCCAGTGCGTGGGCTCACCTTACT

C.4.8 TAGGGGATAGAGTTGCCATTATGGCGGACGGTCGTCTCCAGTGCGTGGGCTCACCTTACT

C.4.10 TAGGGGATAGAGTTGCCATTATGGCGGACGGTCGTCTCCAGTGCGTGGGCTCACCTTACT

A.2.9 TAGGGGATAGAGTTGCCATTATGGCGGACGGTCGTCTCCAGTGCGTGGGCTCACCTTACT

A.1.1 TAGGGGATAGAGTTGCCATTATGGCGGACGGTCGTCTCCAGTGCGTGGGCTCACCTTACT

A.2.2 TAGGGGATAGAGTTGCCATTATGGCGGACGGTCGTCTCCAGTGCGTGGGCTCACCTTACT

APHIS-S TAGGGGATAGAGTTGCCATTATGGCGGACGGTCGTCTCCAGTGCGTGGGCTCACCTTACT

C.3.4 TAGGGGATAGAGTTGCCATTATGGCGGACGGTCGTCTCCAGTGCGTGGGCTCACCTTACT

************************************************************

J.5.2 TCCTCAAGAGACACTATGGAGTCGGCTACACGCTAGTTGTGGTCAAGAAGGAAGATTTCC

J.5.6 TCCTCAAGAGACACTATGGAGTCGGCTACACGCTAGTTGTGGTCAAGAAGGAAGATTTCC

J.5.3 TCCTCAAGAGACACTATGGAGTCGGCTACACGCTAGTTGTGGTCAAGAAGGAAGATTTCC

J.6.4 TCCTCAAGAGACACTATGGAGTCGGCTACACGCTAGTTGTGGTCAAGAAGGAAGATTTCC

C.3.6 TCCTCAAGAGACACTATGGAGTCGGCTACACGCTAGTTGTGGTCAAGAAGGAAGATTTCC

C.4.6 TCCTCAAGAGACACTATGGAGTCGGCTACACGCTAGTTGTGGTCAAGAAGGAAGATTTCC

C.4.8 TCCTCAAGAGACACTATGGAGTCGGCTACACGCTAGTTGTGGTCAAGAAGGAAGATTTCC

C.4.10 TCCTCAAGAGACACTATGGAGTCGGCTACACGCTAGTTGTGGTCAAGAAGGAAGATTTCC

A.2.9 TCCTCAAGAGACACTATGGAGTCGGCTACACGCTAGTTGTGGTCAAGAAGGAAGATTTCC

A.1.1 TCCTCAAGAGACACTATGGAGTCGGCTACACGCTAGTTGTGGTCAAGAAGGAAGATTTCC

A.2.2 TCCTCAAGAGACACTATGGAGTCGGCTACACGCTAGTTGTGGTCAAGAAGGAAGATTTCC

APHIS-S TCCTCAAGAGACACTATGGAGTCGGCTACACGCTAGTTGTGGTCAAGAAGGAAGATTTCC

C.3.4 TCCTCAAGAGACACTATGGAGTCGGCTACACGCTAGTTGTGGTCAAGAAGGAAGATTTCC

************************************************************

J.5.2 GACTGGACACCTGCACAGAGCTGATCAATAGATACATCCCTGGAACTGTTGTGAAGGAAG

J.5.6 RACTGGACACCTGCACAGAGCTGATCAATAGATACATCCCTGGAACTGTTGTGAAGGAAG

J.5.3 GACTGGACACCTGCACAGAGCTGATCAATAGATACATCCCTGGAACTGTTGTGAAGGAAG

J.6.4 GACTGGACACCTGCACAGAGCTGATCAATAGATACATCCCTGGAACTGTTGTGAAGGAAG

C.3.6 GACTGGACACCTGCACAGAGCTGATCAATAGATACATCCCTGGAACTGTTGTGAAGGAAG

C.4.6 GACTGGACACCTGCACAGAGCTGATCAATAGATACATCCCTGGAACTGTTGTGAAGGAAG

C.4.8 GACTGGACACCTGCACAGAGCTGATCAATAGATACATCCCTGGAACTGTTGTGAAGGAAG

C.4.10 GACTGGACACCTGCACAGAGCTGATCAATAGATACATCCCTGGAACTGTTGTGAAGGAAG

A.2.9 GACTGGACACCTGCACAGAGCTGATCAATAGATACATCCCTGGAACTGTTGTGAAGGAAG

A.1.1 GACTGGACACCTGCACAGAGCTGATCAATAGATACATCCCTGGAACTGTTGTGAAGGAAG

A.2.2 GACTGGACACCTGCACAGAGCTGATCAATAGATACATCCCTGGAACTGTTGTGAAGGAAG

APHIS-S GACTGGACACCTGCACAGAGCTGATCAATAGATACATCCCTGGAACTGTTGTGAAGGAAG

C.3.4 GACTGGACACCTGCACAGAGCTGATCAATAGATACATCCCTGGAACTGTTGTGAAGGAAG

***********************************************************

J.5.2 ACCGAGGCACTGAAGTGACATATAGCATGACTAATGAGTATTCGCACGTGTTTGAATCTA

J.5.6 ACCGAGGCACTGAAGTGACATATAGCATGACTAATGAGTATTCGCACGTGTTTGAATCTA

J.5.3 ACCGAGGCACTGAAGTGACATATAGCATGACTAATGAGTATTCGCACGTGTTTGAATCTA

J.6.4 ACCGAGGCACTGAAGTGACATATAGCATGACTAATGAGTATTCGCACGTGTTTGAATCTA

C.3.6 ACCGAGGCACTGAAGTGACATATAGCATGACTAATGAGTATTCGCACGTGTTTGAATCTA

C.4.6 ACCGAGGCACTGAAGTGACATATAGCATGACTAATGAGTATTCGCACGTGTTTGAATCTA

C.4.8 ACCGAGGCACTGAAGTGACATATAGCATGACTAATGAGTATTCGCACGTGTTTGAATCTA

C.4.10 ACCGAGGCACTGAAGTGACATATAGCATGACTAATGAGTATTCGCACGTGTTTGAATCTA

A.2.9 ACCGAGGCACTGAAGTGACATATAGCATGACTAATGAGTATTCGCACGTGTTTGAATCTA

A.1.1 ACCGAGGCACTGAAGTGACATATAGCATGACTAATGAGTATTCGCACGTGTTTGAATCTA

A.2.2 ACCGAGGCACTGAAGTGACATATAGCATGACTAATGAGTATTCGCACGTGTTTGAATCTA

APHIS-S ACCGAGGCACTGAAGTGACATATAGCATGACTAATGAGTATTCGCACGTGTTTGAATCTA

C.3.4 ACCGAGGCACTGAAGTGACATATAGCATGACTAATGAGTATTCGCACGTGTTTGAATCTA

************************************************************

J.5.2 TGCTGCGCGATTTGGAGGCAAAGGCCGATGAGATAAACTTTAAAAACTACGGCCTACTGG

J.5.6 TGCTGCGCGATTTGGAGGCAAAGGCCGATGAGATAAACTTTAAAAACTACGGCCTACTGG

J.5.3 TGCTGCGCGATTTGGAGGCAAAGGCCGATGAGATAAACTTTAAAAACTACGGCCTACTGG

J.6.4 TGCTGCGCGATTTGGAGGCAAAGGCCGATGAGATAAACTTTAAAAACTACGGCCTACTGG

C.3.6 TGCTGCGCGATTTGGAGGCAAAGGCCGATGAGATAAACTTTAAAAACTACGGCCTACTGG

C.4.6 TGCTGCGCGATTTGGAGGCAAAGGCCGATGAGATAAACTTTAAAAACTACGGCCTACTGG

C.4.8 TGCTGCGCGATTTGGAGGCAAAGGCCGATGAGATAAACTTTAAAAACTACGGCCTACTGG

C.4.10 TGCTGCGCGATTTGGAGGCAAAGGCCGATGAGATAAACTTTAAAAACTACGGCCTACTGG

A.2.9 TGCTGCGCGATTTGGAGGCAAAGGCCGATGAGATAAACTTTAAAAACTACGGCCTACTGG

A.1.1 TGCTGCGCGATTTGGAGGCAAAGGCCGATGAGATAAACTTTAAAAACTACGGCCTACTGG

A.2.2 TGCTGCGCGATTTGGAGGCAAAGGCCGATGAGATAAACTTTAAAAACTACGGCCTACTGG

APHIS-S TGCTGCGCGATTTGGAGGCAAAGGCCGATGAGATAAACTTTAAAAACTACGGCCTACTGG

C.3.4 TGCTGCGCGATTTGGAGGCAAAGGCCGATGAGATAAACTTTAAAAACTACGGCCTACTGG

************************************************************

J.5.2 CTACTACATTAGAAGATGTGTTCATGTCCGTGGGCACAGATGTGGTCGCAACTTCAGATG

J.5.6 CTACTACATTAGAAGATGTGTTCATGTCCGTGGGCACAGATGTGGTCGCAACTTCAGATG

J.5.3 CTACTACATTAGAAGATGTGTTCATGTCCGTGGGCACAGATGTGGTCGCAACTTCAGATG

J.6.4 CTACTACATTAGAAGATGTGTTCATGTCCGTGGGCACAGATGTGGTCGCAACTTCAGATG

C.3.6 CTACTACATTAGAAGATGTGTTCATGTCCGTGGGCACAGATGTGGTCGCAACTTCAGATG

C.4.6 CTACTACATTAGAAGATGTGTTCATGTCCGTGGGCACAGATGTGGTCGCAACTTCAGATG

C.4.8 CTACTACATTAGAAGATGTGTTCATGTCCGTGGGCACAGATGTGGTCGCAACTTCAGATG

C.4.10 CTACTACATTAGAAGATGTGTTCATGTCCGTGGGCACAGATGTGGTCGCAACTTCAGATG

A.2.9 CTACTACATTAGAAGATGTGTTCATGTCCGTGGGCACAGATGTGGTCGCAACTTCAGATG

A.1.1 CTACTACATTAGAAGATGTGTTCATGTCCGTGGGCACAGATGTGGTCGCAACTTCAGATG

A.2.2 CTACTACATTAGAAGATGTGTTCATGTCCGTGGGCACAGATGTGGTCGCAACTTCAGATG

APHIS-S CTACTACATTAGAAGATGTGTTCATGTCCGTGGGCACAGATGTGGTCGCAACTTCAGATG

C.3.4 CTACTACATTAGAAGATGTGTTCATGTCCGTGGGCACAGATGTGGTCGCAACTTCAGATG

************************************************************

J.5.2 TGGACGACAATACAACCGTTTCATCTAGTGCTGATACTCTAGCATTTGAATATGATTCTT

J.5.6 TGGACGACAATACAACCGTTTCATCTAGTGCTGATACTCTAGCATTTGAATATGATTCTT

J.5.3 TGGACGACAATACAACCGTTTCATCTAGTGCTGATACTCTAGCATTTGAATATGATTCTT

J.6.4 TGGACGACAATACAACCGTTTCATCTAGTGCTGATACTCTAGCATTTGAATATGATTCTT

C.3.6 TGGACGACAATACAACCGTTTCATCTAGTGCTGATACTCTAGCATTTGAATATGATTCTT

C.4.6 TGGACGACAATACAACCGTTTCATCTAGTGCTGATACTCTAGCATTTGAATATGATTCTT

C.4.8 TGGACGACAATACAACCGTTTCATCTAGTGCTGATACTCTAGCATTTGAATATGATTCTT

C.4.10 TGGACGACAATACAACCGTTTCATCTAGTGCTGATACTCTAGCATTTGAATATGATTCTT

A.2.9 TGGACGACAATACAACCGTTTCATCTAGTGCTGATACTCTAGCATTTGAATATGATTCTT

A.1.1 TGGACGACAATACAACCGTTTCATCTAGTGCTGATACTCTAGCATTTGAATATGATTCTT

A.2.2 TGGACGACAATACAACCGTTTCATCTAGTGCTGATACTCTAGCATTTGAATATGATTCTT

APHIS-S TGGACGACAATACAACCGTTTCATCTAGTGCTGATACTCTAGCATTTGAATATGATTCTT

C.3.4 TGGACGACAATACAACCGTTTCATCTAGTGCTGATACTCTAGCATTTGAATATGATTCTT

************************************************************

J.5.2 TAGAAAAATTGGACGGGACTGGCTATGGGGATGAAAAAGGGATCCGATTAATTTGCCAAC

J.5.6 TAGAAAAATTGGACGGGACTGGCTATGGGGATGAAAAAGGGATCCGATTAATTTGCCAAC

J.5.3 TAGAAAAATTGGACGGGACTGGCTATGGGGATGAAAAAGGGATCCGATTAATTTGCCAAC

J.6.4 TAGAAAAATTGGACGGGACTGGCTATGGGGATGAAAAAGGGATCCGATTAATTTGCCAAC

C.3.6 TAGAAAAATTGGACGGGACTGGCTATGGGGATGAAAAAGGGATCCGATTAATTTGCCAAC

C.4.6 TAGAAAAATTGGACGGGACTGGCTATGGGGATGAAAAAGGGATCCGATTAATTTGCCAAC

C.4.8 TAGAAAAATTGGACGGGACTGGCTATGGGGATGAAAAAGGGATCCGATTAATTTGCCAAC

C.4.10 TAGAAAAATTGGACGGGACTGGCTATGGGGATGAAAAAGGGATCCGATTAATTTGCCAAC

A.2.9 TAGAAAAATTGGACGGGACTGGCTATGGGGATGAAAAAGGGATCCGATTAATTTGCCAAC

A.1.1 TAGAAAAATTGGACGGGACTGGCTATGGGGATGAAAAAGGGATCCGATTAATTTGCCAAC

A.2.2 TAGAAAAATTGGACGGGACTGGCTATGGGGATGAAAAAGGGATCCGATTAATTTGCCAAC

APHIS-S TAGAAAAATTGGACGGGACTGGCTATGGGGATGAAAAAGGGATCCGATTAATTTGCCAAC

C.3.4 TAGAAAAATTGGACGGGACTGGCTATGGGGATGAAAAAGGGATCCGATTAATTTGCCAAC

************************************************************

J.5.2 ACGTGGTAGCAATATGGATGAAACTGTTTCTGGTGCTGACAAGGTCTTGGCTTATCCTGT

J.5.6 ACGTGGTAGCAATATGGATGAAACTGTTTCTGGTGCTGACAAGGTCTTGGCTTATCCTGT

J.5.3 ACGTGGTAGCAATATGGATGAAACTGTTTCTGGTGCTGACAAGGTCTTGGCTTATCCTGT

J.6.4 ACGTGGTAGCAATATGGATGAAACTGTTTCTGGTGCTGACAAGGTCTTGGCTTATCCTGT

C.3.6 ACGTGGTAGCAATATGGATGAAACTGTTTCTGGTGCTGACAAGGTCTTGGCTTATCCTGT

C.4.6 ACGTGGTAGCAATATGGATGAAACTGTTTCTGGTGCTGACAAGGTCTTGGCTTATCCTGT

C.4.8 ACGTGGTAGCAATATGGATGAAACTGTTTCTGGTGCTGACAAGGTCTTGGCTTATCCTGT

C.4.10 ACGTGGTAGCAATATGGATGAAACTGTTTCTGGTGCTGACAAGGTCTTGGCTTATCCTGT

A.2.9 ACGTGGTAGCAATATGGATGAAACTGTTTCTGGTGCTGACAAGGTCTTGGCTTATCCTGT

A.1.1 ACGTGGTAGCAATATGGATGAAACTGTTTCTGGTGCTGACAAGGTCTTGGCTTATCCTGT

A.2.2 ACGTGGTAGCAATATGGATGAAACTGTTTCTGGTGCTGACAAGGTCTTGGCTTATCCTGT

APHIS-S ACGTGGTAGCAATATGGATGAAACTGTTTCTGGTGCTGACAAGGTCTTGGCTTATCCTGT

C.3.4 ACGTGGTAGCAATATGGATGAAACTGTTTCTGGTGCTGACAAGGTCTTGGCTTATCCTGT

************************************************************

J.5.2 TGCTCCAAGTATTGGTGTCCTTGGTACAAATCATTGCCACACTCGGAGTCATGCAGTATG

J.5.6 TGCTCCAAGTATTGGTGTCCTTGGTACAAATCATTGCCACACTCGGAGTCATGCAGTATG

J.5.3 TGCTCCAAGTATTGGTGTCCTTGGTACAAATCATTGCCACACTCGGAGTCATGCAGTATG

J.6.4 TGCTCCAAGTATTGGTGTCCTTGGTACAAATCATTGCCACACTCGGAGTCATGCAGTATG

C.3.6 TGCTCCAAGTATTGGTGTCCTTGGTACAAATCATTGCCACACTCGGAGTCATGCAGTATG

C.4.6 TGCTCCAAGTATTGGTGTCCTTGGTACAAATCATTGCCACACTCGGAGTCATGCAGTATG

C.4.8 TGCTCCAAGTATTGGTGTCCTTGGTACAAATCATTGCCACACTCGGAGTCATGCAGTATG

C.4.10 TGCTCCAAGTATTGGTGTCCTTGGTACAAATCATTGCCACACTCGGAGTCATGCAGTATG

A.2.9 TGCTCCAAGTATTGGTGTCCTTGGTACAAATCATTGCCACACTCGGAGTCATGCAGTATG

A.1.1 TGCTCCAAGTATTGGTGTCCTTGGTACAAATCATTGCCACACTCGGAGTCATGCAGTATG

A.2.2 TGCTCCAAGTATTGGTGTCCTTGGTACAAATCATTGCCACACTCGGAGTCATGCAGTATG

APHIS-S TGCTCCAAGTATTGGTGTCCTTGGTACAAATCATTGCCACACTCGGAGTCATGCAGTATG

C.3.4 TGCTCCAAGTATTGGTGTCCTTGGTACAAATCATTGCCACACTCGGAGTCATGCAGTATG

************************************************************

J.5.2 TCATCTCTATGACCGAGCATATACAAAGAAGAGAACTTTCATTGGCTGAAGGTTTCGCAG

J.5.6 TCATCTCTATGACCGAGCATATACAAAGAAGAGAACTTTCATTGGCTGAAGGTTTCGCAG

J.5.3 TCATCTCTATGACCGAGCATATACAAAGAAGAGAACTTTCATTGGCTGAAGGTTTCGCAG

J.6.4 TCATCTCTATGACCGAGCATATACAAAGAAGAGAACTTTCATTGGCTGAAGGTTTCGCAG

C.3.6 TCATCTCTATGACCGAGCATATACAAAGAAGAGAACTTTCATTGGCTGAAGGTTTCGCAG

C.4.6 TCATCTCTATGACCGAGCATATACAAAGAAGAGAACTTTCATTGGCTGAAGGTTTCGCAG

C.4.8 TCATCTCTATGACCGAGCATATACAAAGAAGAGAACTTTCATTGGCTGAAGGTTTCGCAG

C.4.10 TCATCTCTATGACCGAGCATATACAAAGAAGAGAACTTTCATTGGCTGAAGGTTTCGCAG

A.2.9 TCATCTCTATGACCGAGCATATACAAAGAAGAGAACTTTCATTGGCTGAAGGTTTCGCAG

A.1.1 TCATCTCTATGACCGAGCATATACAAAGAAGAGAACTTTCATTGGCTGAAGGTTTCGCAG

A.2.2 TCATCTCTATGACCGAGCATATACAAAGAAGAGAACTTTCATTGGCTGAAGGTTTCGCAG

APHIS-S TCATCTCTATGACCGAGCATATACAAAGAAGAGAACTTTCATTGGCTGAAGGTTTCGCAG

C.3.4 TCATCTCTATGACCGAGCATATACAAAGAAGAGAACTTTCATTGGCTGAAGGTTTCGCAG

************************************************************

J.5.2 GCACAGAAACATTAGTTAGTTTCAAAGGGTTGTCCCCTACATCGACAGGTTCGCTAGCGA

J.5.6 GCACAGAAACATTAGTTAGTTTCAAAGGGTTGTCCCCTACATCGACAGGTTCGCTAGCGA

J.5.3 GCACAGAAACATTAGTTAGTTTCAAAGGGTTGTCCCCTACATCGACAGGTTCGCTAGCGA

J.6.4 GCACAGAAACATTAGTTAGTTTCAAAGGGTTGTCCCCTACATCGACAGGTTCGCTAGCGA

C.3.6 GCACAGAAACATTAGTTAGTTTCAAAGGGTTGTCCCCTACATCGACAGGTTCGCTAGCGA

C.4.6 GCACAGAAACATTAGTTAGTTTCAAAGGGTTGTCCCCTACATCGACAGGTTCGCTAGCGA

C.4.8 GCACAGAAACATTAGTTAGTTTCAAAGGGTTGTCCCCTACATCGACAGGTTCGCTAGCGA

C.4.10 GCACAGAAACATTAGTTAGTTTCAAAGGGTTGTCCCCTACATCGACAGGTTCGCTAGCGA

A.2.9 GCATAGAAACATTAGTTAGTTTCAAAGGGTTGTCCCCTACATCGACAGGTTCGCTAGCGA

A.1.1 GCACAGAAACATTAGTTAGTTTCAAAGGGTTGTCCCCTACATCGACAGGTTCGCTAGCGA

A.2.2 GCACAGAAACATTAGTTAGTTTCAAAGGGTTGTCCCCTACATCGACAGGTTCGCTAGCGA

APHIS-S GCACAGAAACATTAGTTAGTTTCAAAGGGTTGTCCCCTACATCGACAGGTTCGCTAGCGA

C.3.4 GCACAGAAACATTAGTTAGTTTCAAAGGGTTGTCCCCTACATCGACAGGTTCGCTAGCGA

*** ********************************************************

J.5.2 AGGCTGCCTACGAGTCGATATTTGTAACCGCCAATAATCCCACAATGGAAATCACTGTTG

J.5.6 AGGCTGCCTACGAGTCGATATTTGTAACCGCCAATAATCCCACAATGGAAATCACTGTTG

J.5.3 AGGCTGCCTACGAGTCGATATTTGTAACCGCCAATAATCCCACAATGGAAATCACTGTTG

J.6.4 AGGCTGCCTACGAGTCGATATTTGTAACCGCCAATAATCCCACAATGGAAATCACTGTTG

C.3.6 AGGCTGCCTACGAGTCGATATTTGTAACCGCCAATAATCCCACAATGGAAATCACTGTTG

C.4.6 AGGCTGCCTACGAGTCGATATTTGTAACCGCCAATAATCCCACAATGGAAATCACTGTTG

C.4.8 AGGCTGCCTACGAGTCGATATTTGTAACCGCCAATAATCCCACAATGGAAATCACTGTTG

C.4.10 AGGCTGCCTACGAGTCGATATTTGTAACCGCCAATAATCCCACAATGGAAATCACTGTTG

A.2.9 AGGCTGCCTACGAGTCGATATTTGTAACCGCCAATAATCCCACAATGGAAATCACTGTTG

A.1.1 AGGCTGCCTACGAGTCGATATTTGTAACCGCCAATAATCCCACAATGGAAATCACTGTTG

A.2.2 AGGCTGCCTACGAGTCGATATTTGTAACCGCCAATAATCCCACAATGGAAATCACTGTTG

APHIS-S AGGCTGCCTACGAGTCGATATTTGTAACCGCCAATAATCCCACAATGGAAATCACTGTTG

C.3.4 AGGCTGCCTACGAGTCGATATTTGTAACCGCCAATAATCCCACAATGGAAATCACTGTTG

************************************************************

J.5.2 TTGATAATACACCTATAGATGAATATTATTTGGAAAGAACAGATGACGTATCAGCGATGG

J.5.6 TTGATAATACACCTATAGATGAATATTATTTGGAAAGAACAGATGACGTATCAGCGATGG

J.5.3 TTGATAATACACCTATAGATGAATATTATTTGGAAAGAACAGATGACGTATCAGCGATGG

J.6.4 TTGATAATACACCTATAGATGAATATTATTTGGAAAGAACAGATGACGTATCAGCGATGG

C.3.6 TTGATAATACACCTATAGATGAATATTATTTGGAAAGAACAGATGACGTATCAGCGATGG

C.4.6 TTGATAATACACCTATAGATGAATATTATTTGGAAAGA----ATGACGTATCAGCGATGG

C.4.8 TTGATAATACACCTATAGATGAATATTATTTGGAAAGAACAGATGACGTATCAGCGATGG

C.4.10 TTGATAATACACCTATAGATGAATATTATTTGGAAAGAACAGATGACGTATCAGCGATGG

A.2.9 TTGATAATACACCTATAGATGAATATTATTTGGAAAGAACAGATGACGTATCAGCGATGG

A.1.1 TTGATAATACACCTATAGATGAATATTATTTGGAAAGAACAGATGACGTATCAGCGATGG

A.2.2 TTGATAATACACCTATAGATGAATATTATTTGGAAAGAACAGATGACGTATCAGCGATGG

APHIS-S TTGATAATACACCTATAGATGAATATTATTTGGAAAGAACAGATGACGTATCAGCGATGG

C.3.4 TTGATAATACACCTATAGATGAATATTATTTGGAAAGAACAGATGACGTATCAGCGATGG

************************************** ******************

J.5.2 CGGTGCTCCGGCACAGTCTGTTGATCGGCGCGACGTTCGACGACCACTCCGCGACCGCGT

J.5.6 CGGTGCTCCGGCACAGTCTGTTGATCGGCGCGACGTTCGACGACCACTCCGCGACCGCGT

J.5.3 CGGTGCTCCGGCACAGTCTGTTGATCGGCGCGACGTTCGACGACCACTCCGCGACCGCGT

J.6.4 CGGTGCTCCGGCACAGTCTGTTGATCGGCGCGACGTTCGACGACCACTCCGCGACCGCGT

C.3.6 CGGTGCTCCGGCACAGTCTGTTGATCGGCGCGACGTTCGACGACCACTCCGCGACCGCGT

C.4.6 CGGTGCTCCGGCACAGTCTGTTGATCGGCGCGACGTTCGACGACCACTCCGCGACCGCGT

C.4.8 CGGTGCTCCGGCACAGTCTGTTGATCGGCGCGACGTTCGACGACCACTCCGCGACCGCGT

C.4.10 CGGTGCTCCGGCACAGTCTGTTGATCGGCGCGACGTTCGACGACCACTCCGCGACCGCGT

A.2.9 CGGTGCTCCGGCACAGTCTGTTGATCGGCGCGACGTTCGACGACCACTCCGCGACCGCGT

A.1.1 CGGTGCTCCGGCACAGTCTGTTGATCGGCGCGACGTTCGACGACCACTCCGCGACCGCGT

A.2.2 CGGTGCTCCGGCACAGTCTGTTGATCGGCGCGACGTTCGACGACCACTCCGCGACCGCGT

APHIS-S CGGTGCTCCGGCACAGTCTGTTGATCGGCGCGACGTTCGACGACCACTCCGCGACCGCGT

C.3.4 CGGTGCTCCGGCACAGTCTGTTGATCGGCGCGACGTTCGACGACCACTCCGCGACCGCGT

************************************************************

J.5.2 GGTTCAGCAACTTCGGTTACCACGACGTGGCCATGTCACTGGCTGCTGTGCACGCCGCCT

J.5.6 GGTTCAGCAACTTCGGTTACCACGACGTGGCCATGTCACTGGCTGCTGTGCACGCCGCCT

J.5.3 GGTTCAGCAACTTCGGTTACCACGACGTGGCCATGTCACTGGCTGCTGTGCACGCCGCCT

J.6.4 GGTTCAGCAACTTCGGTTACCACGACGTGGCCATGTCACTGGCTGCTGTGCACGCCGCCT

C.3.6 GGTTCAGCAACTTCGGTTACCACGACGTGGCCATGTCACTGGCTGCTGTGCACGCCGCCT

C.4.6 GGTTCAGCAACTTCGGTTACCACGACGTGGCCATGTCACTGGCTGCTGTGCACGCCGCCT

C.4.8 GGTTCAGCAACTTCGGTTACCACGACGTGGCCATGTCACTGGCTGCTGTGCACGCCGCCT

C.4.10 GGTTCAGCAACTTCGGTTACCACGACGTGGCCATGTCACTGGCTGCTGTGCACGCCGCCT

A.2.9 GGTTCAGCAACTTCGGTTACCACGACGTGGCCATGTCACTGGCTGCTGTGCACGCCGCCT

A.1.1 GGTTCAGCAACTTCGGTTACCACGACGTGGCCATGTCACTGGCTGCTGTGCACGCCGCCT

A.2.2 GGTTCAGCAACTTCGGTTACCACGACGTGGCCATGTCACTGGCTGCTGTGCACGCCGCCT

APHIS-S GGTTCAGCAACTTCGGTTACCACGACGTGGCCATGTCACTGGCTGCTGTGCACGCCGCCT

C.3.4 GGTTCAGCAACTTCGGTTACCACGACGTGGCCATGTCACTGGCTGCTGTGCACGCCGCCT

************************************************************

J.5.2 TGCTCAGAGCTGTCAATCCTGCAGCCAACTTGACTGTTTACAACCACCCACTTGAGGCCA

J.5.6 TGCTCAGAGCTGTCAATCCTGCAGCCAACTTGACTGTTTACAACCACCCACTTGAGGCCA

J.5.3 TGCTCAGAGCTGTCAATCCTGCAGCCAACTTGACTGTTTACAACCACCCACTTGAGGCCA

J.6.4 TGCTCAGAGCTGTCAATCCTGCAGCCAACTTGACTGTTTACAACCACCCACTTGAGGCCA

C.3.6 TGCTCAGAGCTGTCAATCCTGCAGCCAACTTGACTGTTTACAACCACCCACTTGAGGCCA

C.4.6 TGCTCAGAGCTGTCAATCCTGCAGCCAACTTGACTGTTTACAACCACCCACTTGAGGCCA

C.4.8 TGCTCAGAGCTGTCAATCCTGCAGCCAACTTGACTGTTTACAACCACCCACTTGAGGCCA

C.4.10 TGCTCAGAGCTGTCAATCCTGCAGCCAACTTGACTGTTTACAACCACCCACTTGAGGCCA

A.2.9 TGCTCAGAGCTGTCAATCCTGCAGCCAACTTGACTGTTTACAACCACCCACTTGAGGCCA

A.1.1 TGCTCAGAGCTGTCAATCCTGCAGCCAACTTGACTGTTTACAACCACCCACTTGAGGCCA

A.2.2 TGCTCAGAGCTGTCAATCCTGCAGCCAACTTGACTGTTTACAACCACCCACTTGAGGCCA

APHIS-S TGCTCAGAGCTGTCAATCCTGCAGCCAACTTGACTGTTTACAACCACCCACTTGAGGCCA

C.3.4 TGCTCAGAGCTGTCAATCCTGCAGCCAACTTGACTGTTTACAACCACCCACTTGAGGCCA

************************************************************

J.5.2 ATTATGTCAACCAGAACGACATGCAAACAATGGTAGCGTTCCTCTCGATGCAACTTGCGT

J.5.6 ATTATGTCAACCAGAACGACATGCAAACAATGGTAGCGTTCCTCTCGATGCAACTTGCGT

J.5.3 ATTATGTCAACCAGAACGACATGCAAACAATGGTAGCGTTCCTCTCGATGCAACTTGCGT

J.6.4 ATTATGTCAACCAGAACGACATGCAAACAATGGTAGCGTTCCTCTCGATGCAACTTGCGT

C.3.6 ATTATGTCAACCAGAACGACATGCAAACAATGGTAGCGTTCCTCTCGATGCAACTTGCGT

C.4.6 ATTATGTCAACCAGAACGACATGCAAACAATGGTAGCGTTCCTCTCGATGCAACTTGCGT

C.4.8 ATTATGTCAACCAGAACGACATGCAAACAATGGTAGCGTTCCTCTCGATGCAACTTGCGT

C.4.10 ATTATGTCAACCAGAACGACATGCAAACAATGGTAGCGTTCCTCTCGATGCAACTTGCGT

A.2.9 ATTATGTCAACCAG----------------------------------------------

A.1.1 ATTATGTCAACCAG----------------------------------------------

A.2.2 ATTATGTCAACCAG----------------------------------------------

APHIS-S ATTATGTCAACCAGAACGACATGCAAACAATGGTAGCGTTCCTCTCGATGCAACTTGCGT

C.3.4 ATTATGTCAACCAGAACGACATGCAAACAATGGTAGCGTTCCTCTCGATGCAACTTGCGT

**************

J.5.2 CGGGCATCGGCAGCAGTCTGTCAATTGTCAGTGCTGTGTTCATCATGTTCTATATCAAGG

J.5.6 CGGGCATCGGCAGCAGTCTGTCAATTGTCAGTGCTGTGTTCATCATGTTCTATATCAAGG

J.5.3 CGGGCATCGGCAGCAGTCTGTCAATTGTCAGTGCTGTGTTCATCATGTTCTATATCAAGG

J.6.4 CGGGCATCGGCAGCAGTCTGTCAATTGTCAGTGCTGTGTTCATCATGTTCTATATCAAGG

C.3.6 CGGGCATCGGCAGCAGTCTGTCAATTGTCAGTGCTGTGTTCATCATGTTCTATATCAAGG

C.4.6 CGGGCATCGGCAGCAGTCTGTCAATTGTCAGTGCTGTGTTCATCATGTTCTATATCAAGG

C.4.8 CGGGCATCGGCAGCAGTCTGTCAATTGTCAGTGCTGTGTTCATCATGTTCTATATCAAGG

C.4.10 CGGGCATCGGCAGCAGTCTGTCAATTGTCAGTGCTGTGTTCATCATGTTCTATATCAAGG

A.2.9 ------------------------------------------------------------

A.1.1 ------------------------------------------------------------

A.2.2 ------------------------------------------------------------

APHIS-S CGGGCATCGGCAGCAGTCTGTCAATTGTCAGTGCTGTGTTCATCATGTTCTATATCAAGG

C.3.4 CGGGCATCGGCAGCAGTCTGTCAATTGTCAGTGCTGTGTTCATCATGTTCTATATCAAG-

J.5.2 AGCGAGTATCTCGCGCCAAGCTGCTGCAGAAGGCGGCAGGCATCCAGCCGTTAGTGATGT

J.5.6 AGCGAGTATCTCGCGCCAAGCTGCTGCAGAAGGCGGCAGGCATCCAGCCGTTAGTGATGT

J.5.3 AGCGAGTATCTCGCGCCAAGCTGCTGCAGAAGGCGGCAGGCATCCAGCCGTTAGTGATGT

J.6.4 AGCGAGTATCTCGCGCCAAGCTGCTGCAGAAGGCGGCAGGCATCCAGCCGTTAGTGATGT

C.3.6 AGCGAGTATCTCGCGCCAAGCTGCTGCAGAAGGCGGCAGGCATCCAGCCGTTAGTGATGT

C.4.6 AGCGAGTATCTCGCGCCAAGCTGCTGCAGAAGGCGGCAGGCATCCAGCCGTTAGTGATGT

C.4.8 AGCGAGTATCTCGCGCCAAGCTGCTGCAGAAGGCGGCAGGCATCCAGCCGTTAGTGATGT

C.4.10 AGCGAGTATCTCGCGCCAAGCTGCTGCAGAAGGCGGCAGGCATCCAGCCGTTAGTGATGT

A.2.9 ------------------------------------------------------------

A.1.1 ------------------------------------------------------------

A.2.2 ------------------------------------------------------------

APHIS-S AGCGAGTATCTCGCGCCAAGCTGCTGCAGAAGGCGGCAGGCATCCAGCCGTTAGTGATGT

C.3.4 ------------------------------------------------------------

J.5.2 GGCTCAGCGCCGCCGTGTTCGACTGGATCTGGTTCTGCGTCATCGCCGTCGGCATCGTTA

J.5.6 GGCTCAGCGCCGCCGTGTTCGACTGGATCTGGTTCTGCGTCATCGCCGTCGGCATCGTTA

J.5.3 GGCTCAGCGCCGCCGTGTTCGACTGGATCTGGTTCTGCGTCATCGCCGTCGGCATCGTTA

J.6.4 GGCTCAGCGCCGCCGTGTTCGACTGGATCTGGTTCTGCGTCATCGCCGTCGGCATCGTTA

C.3.6 GGCTCAGCGCCGCCGTGTTCGACTGGATCTGGTTCTGCGTCATCGCCGTCGGCATCGTTA

C.4.6 GGCTCAGCGCCGCCGTGTTCGACTGGATCTGGTTCTGCGTCATCGCCGTCGGCATCGTTA

C.4.8 GGCTCAGCGCCGCCGTGTTCGACTGGATCTGGTTCTGCGTCATCGCCGTCGGCATCGTTA

C.4.10 GGCTCAGCGCCGCCGTGTTCGACTGGATCTGGTTCTGCGTCATCGCCGTCGGCATCGTTA

A.2.9 ------------------------------------------------------------

A.1.1 ------------------------------------------------------------

A.2.2 ------------------------------------------------------------

APHIS-S GGCTCAGCGCCGCCGTGTTCGACTGGATCTGGTTCTGCGTCATCGCCGTCGGCATCGTTA

C.3.4 ------------------------------------------------------------

J.5.2 TCGCCTGCGCCGCTTT--------------------------------------------

J.5.6 TCGCCTGCGCCGCTTT--------------------------------------------

J.5.3 TCGCCTGCGCCGCTTT--------------------------------------------

J.6.4 TCGCCTGCGCCGCTTT--------------------------------------------

C.3.6 TCGCCTGCGCCGCTTTTGCGCGCCGTGTTTATTACGGTCATGGCGTCAAAAGATATAGAG

C.4.6 TCGCCTGCGCCGCTTT--------------------------------------------

C.4.8 TCGCCTGCGCCGCTTT--------------------------------------------

C.4.10 TCGCCTGCGCCGCTTT--------------------------------------------

A.2.9 ------------------------------------------------------------

A.1.1 ------------------------------------------------------------

A.2.2 ------------------------------------------------------------

APHIS-S TCGCCTGCGCCGCTTT--------------------------------------------

C.3.4 ------------------------------------------------------------

J.5.2 -----TAACGTCATTGGGC----------------TCTCTTCTGTCGATGAACT------

J.5.6 -----TAACGTCATTGGGC----------------TCTCTTCTGTCGATGAACT------

J.5.3 -----TAACGTCATTGGGC----------------TCTCTTCTGTCGATGAACT------

J.6.4 -----TAACGTCATTGGGC----------------TCTCTTCTGTCGATGAACT------

C.3.6 CTGAATACCTTTATTGTGTATTTCAGTGTTCCACATCGCTTTACTCGGTGAAGTATGCAT

C.4.6 -----TAACGTCATTGGGC----------------TCTCTTCTGTCGATGAACT------

C.4.8 -----TAACGTCATTGGGC----------------TCTCTTCTGTCGATGAACT------

C.4.10 -----TAACGTCATTGGGC----------------TCTCTTCTGTCGATGAACT------

A.2.9 ------------------------------------------------------------

A.1.1 ------------------------------------------------------------

A.2.2 ------------------------------------------------------------

APHIS-S -----TAACGTCATTGGGC----------------TCTCTTCTGTCGATGAACT------

C.3.4 ------------------------------------------------------------

J.5.2 ------------------------------------------------------------

J.5.6 ------------------------------------------------------------

J.5.3 ------------------------------------------------------------

J.6.4 ------------------------------------------------------------

C.3.6 AAAATATGTCGCCTTTTCGGAACTGGCTTGATGGAAATCAATATTTATGTTATCAGTCAG

C.4.6 ------------------------------------------------------------

C.4.8 ------------------------------------------------------------

C.4.10 ------------------------------------------------------------

A.2.9 ------------------------------------------------------------

A.1.1 ------------------------------------------------------------

A.2.2 ------------------------------------------------------------

APHIS-S ------------------------------------------------------------

C.3.4 ------------------------------------------------------------

J.5.2 ------------------------------------------------------------

J.5.6 ------------------------------------------------------------

J.5.3 ------------------------------------------------------------

J.6.4 ------------------------------------------------------------

C.3.6 GATTAGTTGGACGACTCATAAAATATCAATCAATATTCTCTTGCAGCCTCATGAGATCGA

C.4.6 ------------------------------------------------------------

C.4.8 ------------------------------------------------------------

C.4.10 ------------------------------------------------------------

A.2.9 ------------------------------------------------------------

A.1.1 ------------------------------------------------------------

A.2.2 ------------------------------------------------------------

APHIS-S ------------------------------------------------------------

C.3.4 ------------------------------------------------------------

J.5.2 ------------------------------------------------------------

J.5.6 ------------------------------------------------------------

J.5.3 ------------------------------------------------------------

J.6.4 ------------------------------------------------------------

C.3.6 TATGACTCAATGTACTTTACTGAAGTGCTTGCACCTACTGGGTAATAGATAAAACCAATT

C.4.6 ------------------------------------------------------------

C.4.8 ------------------------------------------------------------

C.4.10 ------------------------------------------------------------

A.2.9 ------------------------------------------------------------

A.1.1 ------------------------------------------------------------

A.2.2 ------------------------------------------------------------

APHIS-S ------------------------------------------------------------

C.3.4 ------------------------------------------------------------

J.5.2 ----------------------------------------GGGTCGGATGTACTTGTGCA

J.5.6 ----------------------------------------GGGTCGGATGTACTTGTGCA

J.5.3 ----------------------------------------GGGTCGGATGTACTTGTGCA

J.6.4 ----------------------------------------GGGTCGGATGTACTTGTGCA

C.3.6 GTTTTGTTCATATGTTAGAACCATGTTTTTTTTTTCCTTCAGGTCGGATGTACTTGTGCA

C.4.6 ----------------------------------------GGGTCGGATGTACTTGTGCA

C.4.8 ----------------------------------------GGGTCGGATGTACTTGTGCA

C.4.10 ----------------------------------------GGGTCGGATGTACTTGTGCA

A.2.9 ------------------------------------------GTCGGATGTACTTGTGCA

A.1.1 ------------------------------------------GTCGGATGTACTTGTGCA

A.2.2 ------------------------------------------GTCGGATGTACTTGTGCA

APHIS-S ----------------------------------------GGGTCGGATGTACTTGTGCA

C.3.4 ------------------------------------------GTCGGATGTACTTGTGCA

******************

J.5.2 TCATAGTGTATGGCGCCGCCAGTCTACCGATAGGCTACGTGTTCTCCTATTTCTTCAAAG

J.5.6 TCATAGTGTATGGCGCCGCCAGTCTACCGATAGGCTACGTGTTCTCCTATTTCTTCAAAG

J.5.3 TCATAGTGTATGGCGCCGCCAGTCTACCGATAGGCTACGTGTTCTCCTATTTCTTCAAAG

J.6.4 TCATAGTGTATGGCGCCGCCAGTCTACCGATAGGCTACGTGTTCTCCTATTTCTTCAAAG

C.3.6 TCATAGTGTATGGCGCCGCCAGTCTACCGATAGGCTACGTGTTCTCCTATTTCTTCAAAG

C.4.6 TCATAGTGTATGGCGCCGCCAGTCTACCGATAGGCTACGTGTTCTCCTATTTCTTCAAAG

C.4.8 TCATAGTGTATGGCGCCGCCAGTCTACCGATAGGCTACGTGTTCTCCTATTTCTTCAAAG

C.4.10 TCATAGTGTATGGCGCCGCCAGTCTACCGATAGGCTACGTGTTCTCCTATTTCTTCAAAG

A.2.9 TCATAGTGTATGGCGCCGCCAGTCTACCGATAGGCTACGTGTTCTCCTATTTCTTCAAAG

A.1.1 TCATAGTGTATGGCGCCGCCAGTCTACCGATAGGCTACGTGTTCTCCTATTTCTTCAAAG

A.2.2 TCATAGTGTATGGCGCCGCCAGTCTACCGATAGGCTACGTGTTCTCCTATTTCTTCAAAG

APHIS-S TCATAGTGTATGGCGCCGCCAGTCTACCGATAGGCTACGTGTTCTCCTATTTCTTCAAAG

C.3.4 TCATAGTGTATGGCGCCGCCAGTCTACCGATAGGCTACGTGTTCTCCTATTTCTTCAAAG

************************************************************

J.5.2 GCCCTGCCGTCGGTTTTGTCACCATGTTCTTTATCAACATTCTCTTTGGTATGATGGGGG

J.5.6 GCCCTGCCGTCGGTTTTGTCACCATGTTCTTTATCAACATTCTCTTTGGTATGATGGGGG

J.5.3 GCCCTGCCGTCGGTTTTGTCACCATGTTCTTTATCAACATTCTCTTTGGTATGATGGGGG

J.6.4 GCCCTGCCGTCGGTTTTGTCACCATGTTCTTTATCAACATTCTCTTTGGTATGATGGGGG

C.3.6 GCCCTGCCGTCGGTTTTGTCACCATGTTCTTTATCAACATTCTCTTTGGTATGATGGGGG

C.4.6 GCCCTGCCGTCGGTTTTGTCACCATGTTCTTTATCAACATTCTCTTTGGTATGATGGGGG

C.4.8 GCCCTGCCGTCGGTTTTGTCACCATGTTCTTTATCAACATTCTCTTTGGTATGATGGGGG

C.4.10 GCCCTGCCGTCGGTTTTGTCACCATGTTCTTTATCAACATTCTCTTTGGTATGATGGGGG

A.2.9 GCCCTGCCGTCGGTTTTGTCACCATGTTCTTTATCAACATTCTCTTTGGTATGATGGGGG

A.1.1 GCCCTGCCGTCGGTTTTGTCACCATGTTCTTTATCAACATTCTCTTTGGTATGATGGGGG

A.2.2 GCCCTGCCGTCGGTTTTGTCACCATGTTCTTTATCAACATTCTCTTTGGTATGATGGGGG

APHIS-S GCCCTGCCGTCGGTTTTGTCACCATGTTCTTTATCAACATTCTCTTTGGTATGATGGGGG

C.3.4 GCCCTGCCGTCGGTTTTGTCACCATGTTCTTTATCAACATTCTCTTTGGTATGATGGGGG

************************************************************

J.5.2 CGCAGATTGTGGAGGCCTTGTTGTCACCGCAGCTTGATACTGAAAATGTCGCTAATATAC

J.5.6 CGCAGATTGTGGAGGCCTTGTTGTCACCGCAGCTTGATACTGAAAATGTCGCTAATATAC

J.5.3 CGCAGATTGTGGAGGCCTTGTTGTCACCGCAGCTTGATACTGAAAATGTCGCTAATATAC

J.6.4 CGCAGATTGTGGAGGCCTTGTTGTCACCGCAGCTTGATACTGAAAATGTCGCTAATATAC

C.3.6 CGCAGATTGTGGAGGCCTTGTTGTCACCGCAGCTTGATACTGAAAATGTCGCTAATATAC

C.4.6 CGCAGATTGTGGAGGCCTTGTTGTCACCGCAGCTTGATACTGAAAATGTCGCTAATATAC

C.4.8 CGCAGATTGTGGAGGCCTTGTTGTCACCGCAGCTTGATACTGAAAATGTCGCTAATATAC

C.4.10 CGCAGATTGTGGAGGCCTTGTTGTCACCGCAGCTTGATACTGAAAATGTCGCTAATATAC

A.2.9 CGCAGATTGTGGAGGCCTTGTTGTCACCGCAGCTTGATACTGAAAATGTCGCTAATATAC

A.1.1 CGCAGATTGTGGAGGCCTTGTTGTCACCGCAGCTTGATACTGAAAATGTCGCTAATATAC

A.2.2 CGCAGATTGTGGAGGCCTTGTTGTCACCGCAGCTTGATACTGAAAATGTCGCTAATATAC

APHIS-S CGCAGATTGTGGAGGCCTTGTTGTCACCGCAGCTTGATACTGAAAATGTCGCTAATATAC

C.3.4 CGCAGATTGTGGAGGCCTTGTTGTCACCGCAGCTTGATACTGAAAATGTCGCTAATATAC

************************************************************

J.5.2 TTGACTCCATCTTGCAATTCTTCCCACTCTATAGTCTTGTCACATCTGCCAGACTGTTGA

J.5.6 TTGACTCCATCTTGCAATTCTTCCCACTCTATAGTCTTGTCACATCTGCCAGACTGTTGA

J.5.3 TTGACTCCATCTTGCAATTCTTCCCACTCTATAGTCTTGTCACATCTGCCAGACTGTTGA

J.6.4 TTGACTCCATCTTGCAATTCTTCCCACTCTATAGTCTTGTCACATCTGCCAGACTGTTGA

C.3.6 TTGACTCCATCTTGCAATTCTTCCCACTCTATAGTCTTGTCACATCTGCCAGACTGTTGA

C.4.6 TTGACTCCATCTTGCAATTCTTCCCACTCTATAGTCTTGTCACATCTGCCAGACTGTTGA

C.4.8 TTGACTCCATCTTGCAATTCTTCCCACTCTATAGTCTTGTCACATCTGCCAGACTGTTGA

C.4.10 TTGACTCCATCTTGCAATTCTTCCCACTCTATAGTCTTGTCACATCTGCCAGACTGTTGA

A.2.9 TTGACTCCATCTTGCAATTCTTCCCACTCTATAGTCTTGTCACATCTGCCAGACTGTTGA

A.1.1 TTGACTCCATCTTGCAATTCTTCCCACTCTATAGTCTTGTCACATCTGCCAGACTGTTGA

A.2.2 TTGACTCCATCTTGCAATTCTTCCCACTCTATAGTCTTGTCACATCTGCCAGACTGTTGA

APHIS-S TTGACTCCATCTTGCAATTCTTCCCACTCTATAGTCTTGTCACATCTGCCAGACTGTTGA

C.3.4 TTGACTCCATCTTGCAATTCTTCCCACTCTATAGTCTTGTCACATCTGCCAGACTGTTGA

************************************************************

J.5.2 ATCAGGTGGGACTGCTGGAGTGGTCGTGCCTGCAGAACTGCGAGTACCTGTCCGCAGTGA

J.5.6 ATCAGGTGGGACTGCTGGAGTGGTCGTGCCTGCAGAACTGCGAGTACCTGTCCGCAGTGA

J.5.3 ATCAGGTGGGACTGCTGGAGTGGTCGTGCCTGCAGAACTGCGAGTACCTGTCCGCAGTGA

J.6.4 ATCAGGTGGGACTGCTGGAGTGGTCGTGCCTGCAGAACTGCGAGTACCTGTCCGCAGTGA

C.3.6 ATCAGGTGGGACTGCTGGAGTGGTCGTGCCTGCAGAACTGCGAGTACCTGTCCGCAGTGA

C.4.6 ATCAGGTGGGACTGCTGGAGTGGTCGTGCCTGCAGAACTGCGAGTACCTGTCCGCAGTGA

C.4.8 ATCAGGTGGGACTGCTGGAGTGGTCGTGCCTGCAGAACTGCGAGTACCTGTCCGCAGTGA

C.4.10 ATCAGGTGGGACTGCTGGAGTGGTCGTGCCTGCAGAACTGCGAGTACCTGTCCGCAGTGA

A.2.9 ATCAGGTGGGACTGCTGGAGTGGTCGTGCCTGCAGAACTGCGAGTACCTGTCCGCAGTGA

A.1.1 ATCAGGTGGGACTGCTGGAGTGGTCGTGCCTGCAGAACTGCGAGTACCTGTCCGCAGTGA

A.2.2 ATCAGGTGGGACTGCTGGAGTGGTCGTGCCTGCAGAACTGCGAGTACCTGTCCGCAGTGA

APHIS-S ATCAGGTGGGACTGCTGGAGTGGTCGTGCCTGCAGAACTGCGAGTACCTGTCCGCAGTGA

C.3.4 ATCAGGTGGGACTGCTGGAGTGGTCGTGCCTGCAGAACTGCGAGTACCTGTCCGCAGTGA

************************************************************

J.5.2 TGCCCAACTTGACCGAATGCTCCATGGACGTTATGTGCCAGACGTTCTCACAATGTTGCA

J.5.6 TGCCCAACTTGACCGAATGCTCCATGGACGTTATGTGCCAGACGTTCTCACAATGTTGCA

J.5.3 TGCCCAACTTGACCGAATGCTCCATGGACGTTATGTGCCAGACGTTCTCACAATGTTGCA

J.6.4 TGCCCAACTTGACCGAATGCTCCATGGACGTTATGTGCCAGACGTTCTCACAATGTTGCA

C.3.6 TGCCCAACTTGACCGAATGCTCCATGGACGTTATGTGCCAGACGTTCTCACAATGTTGCA

C.4.6 TGCCCAACTTGACCGAATGCTCCATGGACGTTATGTGCCAGACGTTCTCACAATGTTGCA

C.4.8 TGCCCAACTTGACCGAATGCTCCATGGACGTTATGTGCCAGACGTTCTCACAATGTTGCA

C.4.10 TGCCCAACTTGACCGAATGCTCCATGGACGTTATGTGCCAGACGTTCTCACAATGTTGCA

A.2.9 TGCCCAACTTGACCGAATGCTCCATGGACGTTATGTGCCAGACGTTCTCACAATGTTGCA

A.1.1 TGCCCAACTTGACCGAATGCTCCATGGACGTTATGTGCCAGACGTTCTCACAATGTTGCA

A.2.2 TGCCCAACTTGACCGAATGCTCCATGGACGTTATGTGCCAGACGTTCTCACAATGTTGCA

APHIS-S TGCCCAACTTGACCGAATGCTCCATGGACGTTATGTGCCAGACGTTCTCACAATGTTGCA

C.3.4 TGCCCAACTTGACCGAATGCTCCATGGACGTTATGTGCCAGACGTTCTCACAATGTTGCA

************************************************************

J.5.2 TCCCAGACGATCCTTGGTTCATGTGGGATCACCCTGGAGTACTCCGCTACATAGTATGCA

J.5.6 TCCCAGACGATCCTTGGTTCATGTGGGATCACCCTGGAGTACTCCGCTACATAGTATGCA

J.5.3 TCCCAGACGATCCTTGGTTCATGTGGGATCACCCTGGAGTACTCCGCTACATAGTATGCA

J.6.4 TCCCAGACGATCCTTGGTTCATGTGGGATCACCCTGGAGTACTCCGCTACATAGTATGCA

C.3.6 TCCCAGACGATCCTTGGTTCATGTGGGATCACCCTGGAGTACTCCGCTACATAGTATGTA

C.4.6 TCCCAGACGATCCTTGGTTCATGTGGGATCACCCTGGAGTACTCCGCTACATAGTATGCA

C.4.8 TCCCAGACGATCCTTGGTTCATGTGGGATCACCCTGGAGTACTCCGCTACATAGTATGCA

C.4.10 TCCCAGACGATCCTTGGTTCATGTGGGATCACCCTGGAGTACTCCGCTACATAGTATGCA

A.2.9 TCCCAGACGATCCTTGGTTCATGTGGGATCACCCTGGAGTACTCCGCTACATAGTATGCA

A.1.1 TCCCAGACGATCCTTGGTTCATGTGGGATCACCCTGGAGTACTCCGCTACATAGTATGCA

A.2.2 TCCCAGACGATCCTTGGTTCATGTGGGATCACCCTGGAGTACTCCGCTACATAGTATGCA

APHIS-S TCCCAGACGATCCTTGGTTCATGTGGGATCACCCTGGAGTACTCCGCTACATAGTATGCA

C.3.4 TCCCAGACGATCCTTGGTTCATGTGGGATCACCCTGGAGTACTCCGCTACATAGTATGCA

********************************************************** *

J.5.2 TGATCGTCAGTGGAGTTGTCATGTGGTTCGTACTCTTGATCGCCGAGTATCGATTGTTCC

J.5.6 TGATCGTCAGTGGAGTTGTCATGTGGTTCGTACTCTTGATCGCCGAGTATCGATTGTTCC

J.5.3 TGATCGTCAGTGGAGTTGTCATGTGGTTCGTACTCTTGATCGCCGAGTATCGATTGTTCC

J.6.4 TGATCGTCAGTGGAGTTGTCATGTGGTTCGTACTCTTGATCGCCGAGTATCGATTGTTCC

C.3.6 TGATCGTCAGTGGAGTTGTCATGTGGTTCGTACTCTTGATCGCCGAGTATCGATTGTTCC

C.4.6 TGATCGTCAGTGGAGTTGTCATGTGGTTCGTACTCTTGATCGCCGAGTATCGATTGTTCC

C.4.8 TGATCGTCAGTGGAGTTGTCATGTGGTTCGTACTCTTGATCGCCGAGTATCGATTGTTCC

C.4.10 TGATCGTCAGTGGAGTTGTCATGTGGTTCGTACTCTTGATCGCCGAGTATCGATTGTTCC

A.2.9 TGATCGTCAGTGGAGTTGTCATGTGGTTCGTACTCTTGATCGCCGAGTATCGATTGTTCC

A.1.1 TGATCGTCAGTGGAGTTGTCATGTGGTTCGTACTCTTGATCGCCGAGTATCGATTGTTCC

A.2.2 TGATCGTCAGTGGAGTTGTCATGTGGTTCGTACTCTTGATCGCCGAGTATCGATTGTTCC

APHIS-S TGATCGTCAGTGGAGTTGTCATGTGGTTCGTACTCTTGATCGCCGAGTATCGATTGTTCC

C.3.4 TGATCGTCAGTGGAGTTGTCATGTGGTTCGTACTCTTGATCGCCGAGTATCGATTGTTCC

************************************************************

J.5.2 AGAAGGTGATCTACCGGGAAAAGAAAGCTCCTCCAGTTGATGAGAGCGCACTGGACAATG

J.5.6 AGAAGGTGATCTACCGGGAAAAGAAAGCTCCTCCAGTTGATGAGAGCGCACTGGACAATG

J.5.3 AGAAGGTGATCTACCGGGAAAAGAAAGCTCCTCCAGTTGATGAGAGCGCACTGGACAATG

J.6.4 AGAAGGTGATCTACCGGGAAAAGAAAGCTCCTCCAGTTGATGAGAGCGCACTGGACAATG

C.3.6 AGAAGGTGATCTACCGGGAAAAGAAAGCTCCTCCAGTTGATGAGAGCGCACTGGACAATG

C.4.6 AGAAGGTGATCTACCGGGAAAAGAAAGCTCCTCCAGTTGATGAGAGCGCACTGGACAATG

C.4.8 AGAAGGTGATCTACCGGGAAAAGAAAGCTCCTCCAGTTGATGAGAGCGCACTGGACAATG

C.4.10 AGAAGGTGATCTACCGGGAAAAGAAAGCTCCTCCAGTTGATGAGAGCGCACTGGACAATG

A.2.9 AGAAGGTGATCTACCGGGAAAAGAAAGCTCCTCCAGTTGATGAGAGCGCACTGGACAATG

A.1.1 AGAAGGTGATCTACCGGGAAAAGAAAGCTCCTCCAGTTGATGAGAGCGCACTGGACAATG

A.2.2 AGAAGGTGATCTACCGGGAAAAGAAAGCTCCTCCAGTTGATGAGAGCGCACTGGACAATG

APHIS-S AGAAGGTGATCTACCGGGAAAAGAAAGCTCCTCCAGTTGATGAGAGCGCACTGGACAATG

C.3.4 AGAAGGTGATCTACCGGGAAAAGAAAGCTCCTCCAGTTGATGAGAGCGCACTGGACAATG

************************************************************

J.5.2 ACGTGGCGGACGAGGCCAGACACGTGGCGCGAGTTGGAGCAGGAGCAATCCTCGGGCAGC

J.5.6 ACGTGGCGGACGAGGCCAGACACGTGGCGCGAGTTGGAGCAGGAGCAATCCTCGGGCAGC

J.5.3 ACGTGGCGGACGAGGCCAGACACGTGGCGCGAGTTGGAGCAGGAGCAATCCTCGGGCAGC

J.6.4 ACGTGGCGGACGAGGCCAGACACGTGGCGCGAGTTGGAGCAGGAGCAATCCTCGGGCAGC

C.3.6 ACGTGGCGGACGAGGCCAGACACGTGGCGCGAGTTGGRSMAGRAGCAATCCTCGGGCAGC

C.4.6 ACGTGGCGGACGAGGCCAGACACGTGGCGCGAGTTGGAGCAGGAGCAATCCTCGGGCAGC

C.4.8 ACGTGGCGGACGAGGCCAGACACGTGGCGCGAGTTGGAGCAGGAGCAATCCTCGGGCAGC

C.4.10 ACGTGGCGGACGAGGCCAGACACGTGGCGCGAGTTGGAGCAGGAGCAATCCTCGGGCAGC

A.2.9 ACGTGGCGGACGAGGCCAGACACGTGGCGCGAGTTGGAGCAGGAGCAATCCTCGGGCAGC

A.1.1 ACGTGGCGGACGAGGCCAGACACGTGGCGCGAGTTGGAGCAGGAGCAATCCTCGGGCAGC

A.2.2 ACGTGGCGGACGAGGCCAGACACGTGGCGCGAGTTGGAGCAGGAGCAATCCTCGGGCAGC

APHIS-S ACGTGGCGGACGAGGCCAGACACGTGGCGCGAGTTGGAGCAGGAGCAATCCTCGGGCAGC

C.3.4 ACGTGGCGGACGAGGCCAGACACGTGGCGCGAGTTGGAGCAGGAGCAATCCTCGGGCAGC

************************************* ** *****************

J.5.2 ACAGCCTAGTAGCAAATGGCCTCACCAAGTATTATGGGAAACACCTTGCAGTCAATCAAG

J.5.6 ACAGCCTAGTAGCAAATGGCCTCACCAAGTATTATGGGAAACACCTTGCAGTCAATCAAG

J.5.3 ACAGCCTAGTAGCAAATGGCCTCACCAAGTATTATGGGAAACACCTTGCAGTCAATCAAG

J.6.4 ACAGCCTAGTAGCAAATGGCCTCACCAAGTATTATGGGAAACACCTTGCAGTCAATCAAG

C.3.6 ACAGCCTAGTAGCAAATGGCCTCACCAAGTATTATGGGAAACACCTTGCAGTCAATCAAG

C.4.6 ACAGCCTAGTAGCAAATGGCCTCACCAAGTATTATGGGAAACACCTTGCAGTCAATCAAG

C.4.8 ACAGCCTAGTAGCAAATGGCCTCACCAAGTATTATGGGAAACACCTTGCAGTCAATCAAG

C.4.10 ACAGCCTAGTAGCAAATGGCCTCACCAAGTATTATGGGAAACACCTTGCAGTCAATCAAG

A.2.9 ACAGCCTAGTAGCAAATGGCCTCACCAAGTATTATGGGAAACACATTGCAGTCAATCAAG

A.1.1 ACAGCCTAGTAGCAAATGGCCTCACCAAGTATTATGGGAAACACCTTGCAGTCAATCAAG

A.2.2 ACAGCCTAGTAGCAAATGGCCTCACCAAGTATTATGGGAAACACCTTGCAGTCAATCAAG

APHIS-S ACAGCCTAGTAGCAAATGGCCTCACCAAGTATTATGGGAAACACCTTGCAGTCAATCAAG

C.3.4 ACAGCCTAGTAGCAAATGGCCTCACCAAGTATTATGGGAAACACCTTGCAGTCAATCAAG

******************************************** ***************

J.5.2 TGTCATTCACCGTGGGCGACACGGAATGCTTTGGTCTTCTGGGTGTGAACGGCGCCGGTA

J.5.6 TGTCATTCACCGTGGGCGACACGGAATGCTTTGGTCTTCTGGGTGTGAACGGCGCCGGTA

J.5.3 TGTCATTCACCGTGGGCGACACGGAATGCTTTGGTCTTCTGGGTGTGAACGGCGCCGGTA

J.6.4 TGTCATTCACCGTGGGCGACACGGAATGCTTTGGTCTTCTGGGTGTGAACGGCGCCGGTA

C.3.6 TGTCATTCACCGTGGGCGACACGGAATGCTTTGGTCTTCTGGGTGTGAACGGCGCCGGTA

C.4.6 TGTCATTCACCGTGGGCGACACGGAATGCTTTGGTCTTCTGGGTGTGAACGGCGCCGGTA

C.4.8 TGTCATTCACCGTGGGCGACACGGAATGCTTTGGTCTTCTGGGTGTGAACGGCGCCGGTA

C.4.10 TGTCATTCACCGTGGGCGACACGGAATGCTTTGGTCTTCTGGGTGTGAACGGCGCCGGTA

A.2.9 TGTCATTCACCGTGGGCGACACGGAATGCTTTGGTCTTCTGGGTGTGAACGGCGCCGGTA

A.1.1 TGTCATTCACCGTGGGCGACACGGAATGCTTTGGTCTTCTGGGTGTGAACGGCGCCGGTA

A.2.2 TGTCATTCACCGTGGGCGACACGGAATGCTTTGGTCTTCTGGGTGTGAACGGCGCCGGTA

APHIS-S TGTCATTCACCGTGGGCGACACGGAATGCTTTGGTCTTCTGGGTGTGAACGGCGCCGGTA

C.3.4 TGTCATTCACCGTGGGCGACACGGAATGCTTTGGTCTTCTGGGTGTGAACGGCGCCGGTA

************************************************************

J.5.2 AGACGACCACCTTCAAGATGTTGATGGGATATGAGACCGTCTCCAGCGGAGATGCCTTCG

J.5.6 AGACGACCACCTTCAAGATGTTGATGGGAGATGAGACCGTCTCCAGCGGAGATGCCTTCG

J.5.3 AGACGACCACCTTCAAGATGTTGATGGGAGATGAGACCGTCTCCAGCGGAGATGCCTTCG

J.6.4 AGACGACCACCTTCAAGATGTTGATGGGAGATGAGACCGTCTCCAGCGGAGATGCCTTCG

C.3.6 AGACGACCACCTTCAAGATGTTGATGGGAGATGAGACCGTCTCCAGCGGAGATGCCTTCG

C.4.6 AGACGACCACCTTCAAGATGTTGATGGGAGATGAGACCGTCTCCAGCGGAGATGCCTTCG

C.4.8 AGACGACCACCTTCAAGATGTTGATGGGAGATGAGACCGTCTCCAGCGGAGATGCCTTCG

C.4.10 AGACGACCACCTTCAAGATGTTGATGGGAGATGAGACCGTCTCCAGCGGAGATGCCTTCG

A.2.9 AGACGACCACCTTCAAGATGTTGATGGGAGATGAGACCGTCTCCAGCGGAGATGCCTTCG

A.1.1 AGACGACCACCTTCAAGATGTTGATGGGAGATGAGACCGTCTCCAGCGGAGATGCCTTCG

A.2.2 AGACGACCACCTTCAAGATGTTGATGGGAGATGAGACCGTCTCCAGCGGAGATGCCTTCG

APHIS-S AGACGACCACCTTCAAGATGTTGATGGGAGATGAGACCGTCTCCAGCGGAGATGCCTTCG

C.3.4 AGACGACCACCTTCAAGATGTTGATGGGAGATGAGACCGTCTCCAGCGGAGATGCCTTCG

***************************** ******************************

J.5.2 TGAGTGGCCATTCTGTCAAGACTAATATCACTCAAGTTTACAAAAATATTGGTTACTGTC

J.5.6 TGAGTGGCCATTCTGTCAAGACTAATATCACTCAAGTTTACAAAAATATTGGTTACTGTC

J.5.3 TGAGTGGCCATTCTGTCAAGACTAATATCACTCAAGTTTACAAAAATATTGGTTACTGTC

J.6.4 TGAGTGGCCATTCTGTCAAGACTAATATCACTCAAGTTTACAAAAATATTGGTTACTGTC

C.3.6 TGAGTGGCCATTCTGTCAAGACTAATATCACTCAAGTTTACAAAAATATTGGTTACTGTC

C.4.6 TGAGTGGCCATTCTGTCAAGACTAATATCACTCAAGTTTACAAAAATATTGGTTACTGTC

C.4.8 TGAGTGGCCATTCTGTCAAGACTAATATCACTCAAGTTTACAAAAATATTGGTTACTGTC

C.4.10 TGAGTGGCCATTCTGTCAAGACTAATATCACTCAAGTTTACAAAAATATTGGTTACTGTC

A.2.9 TGAGTGGCCATTCTGTCAAGACTAATATCACTCAAGTTTACAAAAATATTGGTTACTGTC

A.1.1 TGAGTGGCCATTCTGTCAAGACTAATATCACTCAAGTTTACAAAAATATTGGTTACTGTC

A.2.2 TGAGTGGCCATTCTGTCAAGACTAATATCACTCAAGTTTACAAAAATATTGGTTACTGTC

APHIS-S TGAGTGGCCATTCTGTCAAGACTAATATCACTCAAGTTTACAAAAATATTGGTTACTGTC

C.3.4 TGAGTGGCCATTCTGTCAAGACTAATATCACTCAAGTTTACAAAAATATTGGTTACTGTC

************************************************************

J.5.2 CGCAATTCGAAGCGACATTCGGCGAGCTGACGGGACGCGAGACACTACGGCTGTTCTCGG

J.5.6 CGCAATTCGAAGCGACATTCGGCGAGCTGACGGGACGCGAGACACTACGGCTGTTCTCGG

J.5.3 CGCAATTCGAAGCGACATTCGGCGAGCTGACGGGACGCGAGACACTACGGCTGTTCTCGG

J.6.4 CGCAATTCGAAGCGACATTCGGCGAGCTGACGGGACGCGAGACACTACGGCTGTTCTCGG

C.3.6 CGCAATTCGAAGCGACATTCGGCGAGCTGACGGGACGCGAGACACTACGGCTGTTCTCGG

C.4.6 CGCAATTCGAAGCGACATTCGGCGAGCTGACGGGACGCGAGACACTACGGCTGTTCTCGG

C.4.8 CGCAATTCGAAGCGACATTCGGCGAGCTGACGGGACGCGAGACACTACGGCTGTTCTCGG

C.4.10 CGCAATTCGAAGCGACATTCGGCGAGCTGACGGGACGCGAGACACTACGGCTGTTCTCGG

A.2.9 CGCAATTCGAAGCGACATTCGGCGAGCTGACGGGACGCGAGACACTACGGCTGTTCTCGG

A.1.1 CGCAATTCGAAGCGACATTCGGCGAGCTGACGGGACGCGAGACACTACGGCTGTTCTCGG

A.2.2 CGCAATTCGAAGCGACATTCGGCGAGCTGACGGGACGCGAGACACTACGGCTGTTCTCGG

APHIS-S CGCAATTCGAAGCGACATTCGGCGAGCTGACGGGACGCGAGACACTACGGCTGTTCTCGG

C.3.4 CGCAATTCGAAGCGACATTCGGCGAGCTGACGGGACGCGAGACACTACGGCTGTTCTCGG

************************************************************

J.5.2 CGCTGCGAGGGTTGCCAGTGCGAGGCGCCACGCTCCACGCGGAGGCCTTAGCACATGCTC

J.5.6 CGCTGCGAGGGTTGCCAGTGCGAGGCGCCACGCTCCACGCGGAGGCCTTAGCACATGCTC

J.5.3 CGCTGCGAGGGTTGCCAGTGCGAGGCGCCACGCTCCACGCGGAGGCCTTAGCACATGCTC

J.6.4 CGCTGCGAGGGTTGCCAGTGCGAGGCGCCACGCTCCACGCGGAGGCCTTAGCACATGCTC

C.3.6 CGCTGCGAGGGTTGCCAGTGCGAGGCGCCACGCTCCACGCGGAGGCCTTAGCACATGCTC

C.4.6 CGCTGCGAGGGTTGCCAGTGCGAGGCGCCACGCTCCACGCGGAGGCCTTAGCACATGCTC

C.4.8 CGCTGCGAGGGTTGCCAGTGCGAGGCGCCACGCTCCACGCGGAGGCCTTAGCACATGCTC

C.4.10 CGCTGCGAGGGTTGCCAGTGCGAGGCGCCACGCTCCACGCGGAGGCCTTAGCACATGCTC

A.2.9 CGCTGCGAGGGTTGCCAGTGCGAGGCGCCACGCTCCACGCGGAGGCCTTAGCACATGCTC

A.1.1 CGCTGCGAGGGTTGCCAGTGCGAGGCGCCACGCTCCACGCGGAGGCCTTAGCACATGCTC

A.2.2 CGCTGCGAGGGTTGCCAGTGCGAGGCGCCACGCTCCACGCGGAGGCCTTAGCACATGCTC

APHIS-S CGCTGCGAGGGTTGCCAGTGCGAGGCGCCACGCTCCACGCGGAGGCCTTAGCACATGCTC

C.3.4 CGCTGCGAGGGTTGCCAGTGCGAGGCGCCACGCTCCACGCGGAGGCCTTAGCACATGCTC

************************************************************

J.5.2 TTGGTTTCTATAAGCATCTTGATAAAAG--------------------------------

J.5.6 TTGGTTTCTATAAGCATCTTGATAAAAG--------------------------------

J.5.3 TTGGTTTCTATAAGCATCTTGATAAAAG--------------------------------

J.6.4 TTGGTTTCTATAAGCATCTTGATAAAAG--------------------------------

C.3.6 TTGGTTTCTATAAGCATCTTGATAAAAGGGTAAGAATGGAACTTGACTCTCAATAAGCGT

C.4.6 TTGGTTTCTATAAGCATCTTGATAAAAG--------------------------------

C.4.8 TTGGTTTCTATAAGCATCTTGATAAAAG--------------------------------

C.4.10 TTGGTTTCTATAAGCATCTTGATAAAAG--------------------------------

A.2.9 TTGGTTTCTATAAGCATCTTGATAAAAG--------------------------------

A.1.1 TTGGTTTCTATAAGCATCTTGATAAAAG--------------------------------

A.2.2 TTGGTTTCTATAAGCATCTTGATAAAAG--------------------------------

APHIS-S TTGGTTTCTATAAGCATCTTGATAAAAG--------------------------------

C.3.4 TTGGTTTCTATAAGCATCTTGATAAAAG--------------------------------

****************************

J.5.2 ------------------------------------------------------------

J.5.6 ------------------------------------------------------------

J.5.3 ------------------------------------------------------------

J.6.4 ------------------------------------------------------------

C.3.6 TTTTAAAATAAACGATTTTGAATAAAAATGCGACTAATGCGATATCCTAAATGCGGAGTT

C.4.6 ------------------------------------------------------------

C.4.8 ------------------------------------------------------------

C.4.10 ------------------------------------------------------------

A.2.9 ------------------------------------------------------------

A.1.1 ------------------------------------------------------------

A.2.2 ------------------------------------------------------------

APHIS-S ------------------------------------------------------------

C.3.4 ------------------------------------------------------------

J.5.2 ------------------------------------------------------------

J.5.6 ------------------------------------------------------------

J.5.3 ------------------------------------------------------------

J.6.4 ------------------------------------------------------------

C.3.6 GAAAAAGAAGGATTTCACCGTTGGGTAACGAGGATCCGGCAAGGTGGGAGGGATTTAGTA

C.4.6 ------------------------------------------------------------

C.4.8 ------------------------------------------------------------

C.4.10 ------------------------------------------------------------

A.2.9 ------------------------------------------------------------

A.1.1 ------------------------------------------------------------

A.2.2 ------------------------------------------------------------

APHIS-S ------------------------------------------------------------

C.3.4 ------------------------------------------------------------

J.5.2 -------------------------------------------GGTGGACCACTACTCTG

J.5.6 -------------------------------------------GGTGGACCACTACTCTG

J.5.3 -------------------------------------------GGTGGACCACTACTCTG

J.6.4 -------------------------------------------GGTGGACCACTACTCTG

C.3.6 TTCAATTCGTGAAAGGCAGTTTAGATAGTTTCTTCTATGTGCAGGTGGACCACTACTCTG

C.4.6 -------------------------------------------GGTGGACCACTACTCTG

C.4.8 -------------------------------------------GGTGGACCACTACTCTG

C.4.10 -------------------------------------------GGTGGACCACTACTCTG

A.2.9 -------------------------------------------GGTGGACCACTACTCTG

A.1.1 -------------------------------------------GGTGGACCACTACTCTG

A.2.2 -------------------------------------------GGTGGACCACTACTCTG

APHIS-S -------------------------------------------GGTGGACCACTACTCTG

C.3.4 -------------------------------------------GGTGGACCACTACTCTG

*****************

J.5.2 GTGGCAACAAGCGCAAGTTGAGCACGGCTGTGGCGTTGCTGGGGCGCACGCGGCTTATAT

J.5.6 GTGGCAACAAGCGCAAGTTGAGCACGGCTGTGGCGTTGCTGGGGCGCACGCGGCTTATAT

J.5.3 GTGGCAACAAGCGCAAGTTGAGCACGGCTGTGGCGTTGCTGGGGCGCACGCGGCTTATAT

J.6.4 GTGGCAACAAGCGCAAGTTGAGCACGGCTGTGGCGTTGCTGGGGCGCACGCGGCTTATAT

C.3.6 GTGGCAACAAGCGCAAGTTGAGCACGGCTGTGGCGTTGCTGGGGCGCACGCGGCTTATAT

C.4.6 GTGGCAACAAGCGCAAGTTGAGCACGGCTGTGGCGTTGCTGGGGCGCACGCGGCTTATAT

C.4.8 GTGGCAACAAGCGCAAGTTGAGCACGGCTGTGGCGTTGCTGGGGCGCACGCGGCTTATAT

C.4.10 GTGGCAACAAGCGCAAGTTGAGCACGGCTGTGGCGTTGCTGGGGCGCACGCGGCTTATAT

A.2.9 GTGGCAACAAGCGCAAGTTGAGCACGGCTGTGGCGTTGCTGGGGCGCACGCGGCTTATAT

A.1.1 GTGGCAACAAGCGCAAGTTGAGCACGGCTGTGGCGTTGCTGGGGCGCACGCGGCTTATAT

A.2.2 GTGGCAACAAGCGCAAGTTGAGCACGGCTGTGGCGTTGCTGGGGCGCACGCGGCTTATAT

APHIS-S GTGGCAACAAGCGCAAGTTGAGCACGGCTGTGGCGTTGCTGGGGCGCACGCGGCTTATAT

C.3.4 GTGGCAACAAGCGCAAGTTGAGCACGGCTGTGGCGTTGCTGGGGCGCACGCGGCTTATAT

************************************************************

J.5.2 TCGTCGACGAACCCACTACTGGAGTCGATCCTGCTGCTAAGAGACAGATGTGGAACGCGG

J.5.6 TCGTCGACGAACCCACTACTGGAGTCGATCCTGCTGCTAAGAGACAGATGTGGAACGCGG

J.5.3 TCGTCGACGAACCCACTACTGGAGTCGATCCTGCTGCTAAGAGACAGATGTGGAACGCGG

J.6.4 TCGTCGACGAACCCACTACTGGAGTCGATCCTGCTGCTAAGAGACAGATGTGGAACGCGG

C.3.6 TCGTCGACGAACCCACTACTGGAGTCGATCCTGCTGCTAAGAGACAGATGTGGAACGCGG

C.4.6 TCGTCGACGAACCCACTACTGGAGTCGATCCTGCTGCTAAGAGACAGATGTGGAACGCGG

C.4.8 TCGTCGACGAACCCACTACTGGAGTCGATCCTGCTGCTAAGAGACAGATGTGGAACGCGG

C.4.10 TCGTCGACGAACCCACTACTGGAGTCGATCCTGCTGCTAAGAGACAGATGTGGAACGCGG

A.2.9 TCGTCGACGAACCCACTACTGGAGTCGATCCTGCTGCTAAGAGACAGATGTGGAACGCGG

A.1.1 TCGTCGACGAACCCACTACTGGAGTCGATCCTGCTGCTAAGAGACAGATGTGGAACGCGG

A.2.2 TCGTCGACGAACCCACTACTGGAGTCGATCCTGCTGCTAAGAGACAGATGTGGAACGCGG

APHIS-S TCGTCGACGAACCCACTACTGGAGTCGATCCTGCTGCTAAGAGACAGATGTGGAACGCGG

C.3.4 TCGTCGACGAACCCACTACTGGAGTCGATCCTGCTGCTAAGAGACAGATGTGGAACGCGG

************************************************************

J.5.2 TTCGAGAAGCTCGCCGGTCGGGTCGTGGTGTGGTGCTGACATCACACAGCATGGAGGAGT

J.5.6 TTCGAGAAGCTCGCCGGTCGGGTCGTGGTGTGGTGCTGACATCACACAGCATGGAGGAGT

J.5.3 TTCGAGAAGCTCGCCGGTCGGGTCGTGGTGTGGTGCTGACATCACACAGCATGGAGGAGT

J.6.4 TTCGAGAAGCTCGCCGGTCGGGTCGTGGTGTGGTGCTGACATCACACAGCATGGAGGAGT

C.3.6 TTCGAGAAGCTCGCCGGTCGGGTCGTGGTGTGGTGCTGACATCACACAGCATGGAGGAGT

C.4.6 TTCGAGAAGCTCGCCGGTCGGGTCGTGGTGTGGTGCTGACATCACACAGCATGGAGGAGT

C.4.8 TTCGAGAAGCTCGCCGGTCGGGTCGTGGTGTGGTGCTGACATCACACAGCATGGAGGAGT

C.4.10 TTCGAGAAGCTCGCCGGTCGGGTCGTGGTGTGGTGCTGACATCACACAGCATGGAGGAGT

A.2.9 TTCGAGAAGCTCGCCGGTCGGGTCGTGGTGTGGTGCTGACATCACACAGCATGGAGGAGT

A.1.1 TTCGAGAAGCTCGCCGGTCGGGTCGTGGTGTGGTGCTGACATCACACAGCATGGAGGAGT

A.2.2 TTCGAGAAGCTCGCCGGTCGGGTCGTGGTGTGGTGCTGACATCACACAGCATGGAGGAGT

APHIS-S TTCGAGAAGCTCGCCGGTCGGGTCGTGGTGTGGTGCTGACATCACACAGCATGGAGGAGT

C.3.4 TTCGAGAAGCTCGCCGGTCGGGTCGTGGTGTGGTGCTGACATCACACAGCATGGAGGAGT

************************************************************

J.5.2 GTGAGGCTCTGTGCTCGCGGCTCACAATCATGGTCAACGGACAGTTCCAGTGCCTCGGCA

J.5.6 GTGAGGCTCTGTGCTCGCGGCTCACAATCATGGTCAACGGACAGTTCCAGTGCCTCGGCA

J.5.3 GTGAGGCTCTGTGCTCGCGGCTCACAATCATGGTCAACGGACAGTTCCAGTGCCTCGGCA

J.6.4 GTGAGGCTCTGTGCTCGCGGCTCACAATCATGGTCAACGGACAGTTCCAGTGCCTCGGCA

C.3.6 GTGAGGCTCTGTGCTCGCGGCTCACAATCATGGTCAACGGACAGTTCCAGTGCCTCGGCA

C.4.6 GTGAGGCTCTGTGCTCGCGGCTCACAATCATGGTCAACGGACAGTTCCAGTGCCTCGGCA

C.4.8 GTGAGGCTCTGTGCTCGCGGCTCACAATCATGGTCAACGGACAGTTCCAGTGCCTCGGCA

C.4.10 GTGAGGCTCTGTGCTCGCGGCTCACAATCATGGTCAACGGACAGTTCCAGTGCCTCGGCA

A.2.9 GTGAGGCTCTGTGCTCGCGGCTCACAATCATGGTCAACGGACAGTTCCAGTGCCTCGGCA

A.1.1 GTGAGGCTCTGTGCTCGCGGCTCACAATCATGGTCAACGGACAGTTCCAGTGCCTCGGCA

A.2.2 GTGAGGCTCTGTGCTCGCGGCTCACAATCATGGTCAACGGACAGTTCCAGTGCCTCGGCA

APHIS-S GTGAGGCTCTGTGCTCGCGGCTCACAATCATGGTCAACGGACAGTTCCAGTGCCTCGGCA

C.3.4 GTGAGGCTCTGTGCTCGCGGCTCACAATCATGGTCAACGGACAGTTCCAGTGCCTCGGCA

************************************************************

J.5.2 CGCCGCAACATTTAAAGAATAAGTTCTCTGAAGGTTTCACATTGACAATTAAAATTAAAG

J.5.6 CGCCGCAACATTTAAAGAATAAGTTCTCTGAAGGTTTCACATTGACAATTAAAATTAAAG

J.5.3 CGCCGCAACATTTAAAGAATAAGTTCTCTGAAGGTTTCACATTGACAATTAAAATTAAAG

J.6.4 CGCCGCAACATTTAAAGAATAAGTTCTCTGAAGGTTTCACATTGACAATTAAAATTAAAG

C.3.6 CGCCGCAACATTTAAAGAATAAGTTCTCTGAAGGTTTCACATTGACAATTAAAATTAAAG

C.4.6 CGCCGCAACATTTAAAGAATAAGTTCTCTGAAGGTTTCACATTGACAATTAAAATTAAAG

C.4.8 CGCCGCAACATTTAAAGAATAAGTTCTCTGAAGGTTTCACATTGACAATTAAAATTAAAG

C.4.10 CGCCGCAACATTTAAAGAATAAGTTCTCTGAAGGTTTCACATTGACAATTAAAATTAAAG

A.2.9 CGCCGCAACATTTAAAGAATAAGTTCTCTGAAGGTTTCACATTGACAATTAAAATTAAAG

A.1.1 CGCCGCAACATTTAAAGAATAAGTTCTCTGAAGGTTTCACATTGACAATTAAAATTAAAG

A.2.2 CGCCGCAACATTTAAAGAATAAGTTCTCTGAAGGTTTCACATTGACAATTAAAATTAAAG

APHIS-S CGCCGCAACATTTAAAGAATAAGTTCTCTGAAGGTTTCACATTGACAATTAAAATTAAAG

C.3.4 CGCCGCAACATTTAAAGAATAAGTTCTCTGAAGGTTTCACATTGACAATTAAAATTAAAG

************************************************************

J.5.2 TGGACGACGAGACGAAGACTGTACGGCCTGAAGTCTGCGATGCTGTGAAGCATTACGTCA

J.5.6 TGGACGACGAGACGAAGACTGTACGGCCTGAAGTCTGCGATGCTGTGAAGCATTACGTCA

J.5.3 TGGACGACGAGACGAAGACTGTACGGCCTGAAGTCTGCGATGCTGTGAAGCATTACGTCA

J.6.4 TGGACGACGAGACGAAGACTGTACGGCCTGAAGTCTGCGATGCTGTGAAGCATTACGTCA

C.3.6 TGGACGACGAGACGAAGACTGTACGGCCTGAAGTCTGCGATGCTGTGAAGCATTACGTCA

C.4.6 TGGACGACGAGACGAAGACTGTACGGCCTGAAGTCTGCGATGCTGTGAAGCATTACGTCA

C.4.8 TGGACGACGAGACGAAGACTGTACGGCCTGAAGTCTGCGATGCTGTGAAGCATTACGTCA

C.4.10 TGGACGACGAGACGAAGACTGTACGGCCTGAAGTCTGCGATGCTGTGAAGCATTACGTCA

A.2.9 TGGACGACGAGACGAAGACTGTACGGCCTGAAGTCTGCGATGCTGTGAAGCATTACGTCA

A.1.1 TGGACGACGAGACGAAGACTGTACGGCCTGAAGTCTGCGATGCTGTGAAGCATTACGTCA

A.2.2 TGGACGACGAGACGAAGACTGTACGGCCTGAAGTCTGCGATGCTGTGAAGCATTACGTCA

APHIS-S TGGACGACGAGACGAAGACTGTACGGCCTGAAGTCTGCGATGCTGTGAAGCATTACGTCA

C.3.4 TGGACGACGAGACGAAGACTGTACGGCCTGAAGTCTGCGATGCTGTGAAGCATTACGTCA

************************************************************

J.5.2 GTACCAACTTCAGAGAGCCGAAGATTATGGAGGAGTACCAGGGTCTGTTAACATACTATT
[truncated: 29,686 more chars]
